# Supplementary material for: Poly-β-D-(1,6)-N-acetyl-glucosamine (PNAG) glycan vaccines with broad spectrum neutralizing activities
Source: Nat Commun. 2025 Jul 4;16:6179. doi: 10.1038/s41467-025-61559-7 (PMC12227562; doi:10.1038/s41467-025-61559-7)
Supplement: Supplementary file 1 — Supplementary Information [file 41467_2025_61559_MOESM1_ESM.pdf]

Supporting Information

Poly-β-D-(1,6)-*N*-Acetyl-Glucosamine (PNAG) Glycan Vaccines with Broad Spectrum Neutralizing Activities

Kuo-Shiang Liao[1,2], Mu-Rong Kao[1,2,3], Tzu-Hsuan Ma[2,3], Mei-Hua Hsu[4], Tzu-Yin Chen[2], Balázs Imre[1,2,3], Philip J. Harris[5], Jiun-Jie Shie[6], Cheng-Hsun Chiu[4], Chung-Yi Wu[1], Yves S. Y. Hsieh[1,2,3]*

Author Information

Affiliations

[1]Genomics Research Center, Academia Sinica, 128 Academia Road, Section 2, Nankang, Taipei 115, Taiwan

[2]School of Pharmacy, College of Pharmacy, Taipei Medical University, 250 Wuxing Street, Taipei 11031, Taiwan

[3]Division of Glycoscience, Department of Chemistry, School of Engineering Sciences in Chemistry, Biotechnology and Health, KTH Royal Institute of Technology, AlbaNova University Centre, Stockholm, SE10691, Sweden

[4]Molecular Infectious Disease Research Center, Chang Gung Memorial Hospital, Chang Gung University College of Medicine, 259, Wenhua 1st Rd., Guishan Dist., Taoyuan 333, Taiwan

[5]School of Biological Sciences, The University of Auckland, Auckland Mail Centre, Private Bag 92019, Auckland 1142, New Zealand

[6]Institute of Chemistry, Academia Sinica, 128 Academia Road, Section 2, Nankang, Taipei 115, Taiwan

TABLE OF CONTENT

General Information-- S3

Figure S1. Synthesis of building blocks **6**, **7**, **8**, **9**-- S4

Figure S2. Synthesis of disaccharide building blocks **10** and **11**---------------------------- S8

Figure S3. Stepwise synthesis of oligoglucosamines -- S10

Figure S4. Synthesis of thiol linker and global deprotection--------------------------------- S19

Figure S5. Synthesis of dPNAG glycan antigens with random *N*-acetylation------------- S25

Figure S6. Synthesis of dPNAG tetra, octa, dodeca, octadecasaccharide-CRM197 as vaccine candidates--- S30

Table S1. dPNAG Glycan antigens with different chain lengths conjugated to the CRM197 carrier protein -- S30

MALDI-TOF spectra of PNAG–CRM197 glycan conjugates------------------------------- S31

^{1}H and ^{13}C NMR Spectra (Figure S7 - S85) -- S40

Table S2. Statistical significance and P values for dPNAG glycan binding assays with serum antibodies from infected patients-- S120

Table S3. Statistical significance and P values for dPNAG glycan binding assays with sera from mice immunized with dPNAG-CRM197 conjugates----------------------------- S128

Table S4. Statistical significance and P values for the opsonophagocytic killing assay with *Staphylococcus aureus* (Newman)-- S140

Table S5. Statistical significance and P values for the opsonophagocytic killing assay with *Strepcoccus pneumoniaea* (19A)--- S142

Table S6. Statistical significance and P values for the opsonophagocytic killing assay with *Acinetobacter baumannii* (17978)-- S143

Figure S86. Glycan array analysis of antibodies that bind to dPNAG glycans in the serum from a patient without bacterial infection--- S145

Figure S87. Immunization of mice and evaluation of the immunogenicity of the acetylated and non-acetylated dPNAG-CRM197 conjugates as vaccine candidates------- S146

Figure S88. Evaluating in vivo efficacy of vaccine candidates for protection against *Staphylococcus aureus* infection-- S147

General Information. All reactions were carried out in an inert atmosphere unless mentioned otherwise, and standard syringe–septa techniques were followed. Solvents were purchased from Acros, Echo chemical, Merck, J. T. Baker, Sigma-Aldrich and Fluka and used without further purification. Pulverized molecule sieve 4Å (Acros) was dried in 200°C in a high vacuum. The progress of all the reactions was monitored by TLC, using TLC glass plates precoated with silica gel 60 F254 (Merck). Reaction product was detected by UV light (254 nm), *p*-anisaldehyde, or ceric ammonium molybdate. Column chromatography was performed on silica gel Geduran® Si 60 (40-63 μm, Merck). ^{1}H and ^{13}C NMR spectra were recorded with a Bruker AVANCE 600 (600 MHz) spectrometer at 25 ^{o}C and chemical shifts were measured in δ (ppm) with residual solvent peaks as internal standards ($CDCl_3$, δ 7.24 ppm D_2O δ 4.80, DMSO-d_6, δ 2.50 ppm in ^{1}H NMR and $CDCl_3$, δ 77 ppm, DMSO-d6, δ 39.5 ppm in ^{13}C NMR). Coupling constants *J*, were measured in Hz. Data are represented as follows: chemical shifts, and multiplicity (s = singlet, d= doublet, t = triplet, q = quartet, m= multiplet, br = broad). HR MALDI-TOF (LR MALDI-TOF) mass spectra were recorded on a Bruker Ultraflex II TOF/TOF200 sepctrameter using sinapinic acid as the matrix. HR ESI mass spectra were recorded on an APEX-ultra 9.4 T FTICR mass spectrometer (Bruker Daltonics).

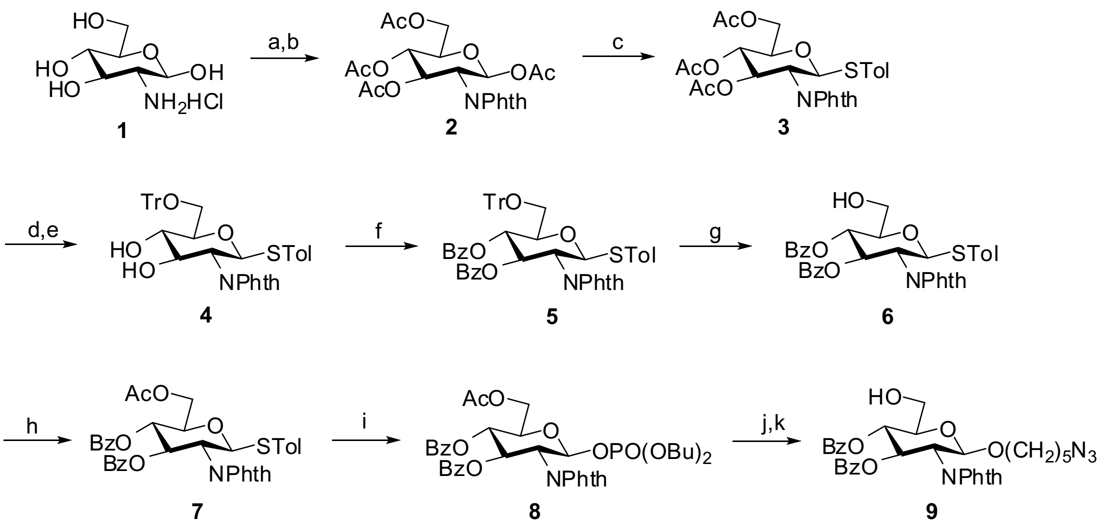

Figure S1. Synthesis of Building Blocks **6**, **7**, **8**, **9**

Reagents and conditions: (a) NaOMe, phthalic anhydride, MeOH, 65 ºC, 2 h; (b) Ac_2O, pyridine, rt, 16 h, 73% (two steps); (c) TolSH, $BF_3O{\cdot}Et_2O$, CH_2Cl_2, rt, 24 h, 65%; (d) NaOMe, MeOH, rt, 2 h then TrCl, cat. DMAP, pyridine, CH_2Cl_2, rt, 24 h, 94%; (e) BzCl, cat. DMAP, pyridine, CH_2Cl_2, rt, 16 h, 89%; (f) *p*TSA, MeOH/CH_2Cl_2, rt, 8 h, 91%; (g) Ac_2O, cat. DMAP, pyridine, CH_2Cl_2, rt, 2 h, 99%; (h) $HOPO(OBu)_2$, NIS/TfOH, CH_2Cl_2, MS 4Å, 0 ºC, 16 h, 95%; (i) $HO(CH_2)_5N_3$, TMSOTf, CH_2Cl_2, MS 4Å, -40 to -25 ºC, 2 h, 90%; (j) AcCl, CH_2Cl_2/MeOH, rt, 16 h, 97%. Phth = Phthalimide, Tr = Trityl, DMAP = 4-(Dimethylamino)pyridine, *p*TSA = *p*-Toluenesulfonic acid monohydrate, NIS = N-Iodosuccinimide.

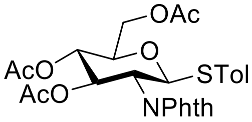

Compound 3. Spectroscopic data and protocol were identical to that reported previously.[1]

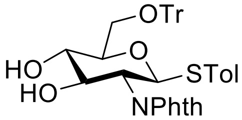

Compound 4. Compound **3** (76.0 g, 140 mmol) was added NaOMe (2.27 g, 42.1 mmol, 0.3 eq.) in methanol (600 mL) at room temperature. After stirring two hours, the solvent was directly removed under reduced pressure and the residue dissolved in CH_2Cl_2 (500 mL) and pyridine (33.9 mL, 421 mmol, 3 eq.) was added at room temperature. Then triphenylmethyl chloride (78.2 g, 281 mmol, 2 eq.) and the cat. DMAP (4-dimethylaminopyridine, 1.71 g, 14.0 mmol, 0.1 eq.) were added to the reaction mixture. The solvents were removed under reduced pressure and the residue purified by normal-phase silica gel column with the eluent EA/hexane = 2: 1 to produce compound **4** as a white powder (87.0 g, 94%). White solid; R_f = 0.44 (silica gel, EtOAc : hexane 1 : 1); ^1H NMR (600 MHz, $CDCl_3$): δ 7.83 (br, 1H, Ar-H), 7.78 (br, 1H, Ar-H), 7.68 (d, J = 3.0 Hz, 2H, Ar-H), 7.48-7.46 (m, 6H, Ar-H), 7.34 (d, J = 7.8 Hz, 2H, Ar-H), 7.31-7.29 (m, 6H, Ar-H), 7.25-7.23 (m, 3H, Ar-H), 7.01 (d, J = 7.8 Hz, 2H, Ar-H), 5.52 (d, J = 10.8 Hz, 1H, C1-H_β), 4.25 (dd, J = 9.6, 9.0 Hz, 1H), 4.17 (dd, J = 10.8, 10.2 Hz, 1H), 3.57-3.51 (m, 2H), 3.47 (dd, J = 10.2, 3.6 Hz, 1H), 3.40 (dd, J = 10.2, 4.8 Hz, 1H), 2.84 (br, 1H, OH), 2.79 (br, 1H, OH), 2.28 (s, 3H, Ar-CH_3); ^{13}C

NMR (150 MHz, $CDCl_3$): δ = 168.3, 167.8, 143.6, 138.1, 134.1, 133.2, 131.6, 129.6, 128.6, 128.1, 127.9, 127.1, 123.7, 87.0, 83.5. 78.1, 73.0, 72.7, 63.7, 55.3; HRMS (ESI-TOF) m/e : Calcd for $C_{40}H_{35}NO_6SNa$ $[M+Na]^+$: 680.2077 Found 680.2110.

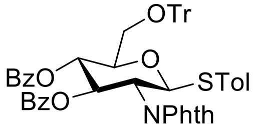

Compound 5. Compound **4** (87.0 g, 13.2 mmol, 1.0 eq.) was dissolved in pyridine (100 mL) and CH_2Cl_2 (200 mL) at room temperature. Benzoyl chloride (61.5 mL, 529 mmol, 4.0 eq.) and the cat. DMAP (1.62 g, 13.2 mmol, 0.10 eq.) were added and stirred continuously overnight at room temperature. The solvents were removed under reduced pressure and the residue purified by normal-phase silica gel column chromatography with the eluent EA/hexane = 1 : 9 ~ 1 : 3 followed by CH_2Cl_2 to give compound **5**, as a white powder (102 g, 89%). White solid; R_f = 0.37 (silica gel, EtOAc : hexane 1 : 2); ^{1}H NMR (600 MHz, $CDCl_3$): δ 7.89 (d, J = 7.2 Hz, 1H, Ar-H), 7.74 (d, J = 7.8 Hz, 3H, Ar-H), 7.70 (d, J = 9.0 Hz, 3H, Ar-H), 7.68-7.64 (m, 1H, Ar-H), 7.51 (d, J = 7.8 Hz, 2H, Ar-H), 7.45 (d, J = 7.8 Hz, 7H, Ar-H), 7.39 (dd, J = 7.2, 7.2 Hz, 1H, Ar-H), 7.29-7.23 (m, 4H, Ar-H), 7.18 (dd, J = 7.8, 7.2 Hz, 6H, Ar-H), 7.14-7.09 (m, 5H, Ar-H), 6.18 (dd, J = 10.2, 9.6 Hz, 1H, CH), 5.86 (d, J = 10.2 Hz, 1H, C1-H_β), 5.63 (dd, J = 10.2, 9.6 Hz, 1H, CH), 4.65 (dd, J = 10.2, 10.2 Hz, 1H), 4.00 (dd, J = 10.2, 4.8 Hz, 1H), 3.39 (d, J = 10.2 Hz, 1H), 3.30 (dd, J = 10.2, 4.8 Hz, 1H), 2.34 (s, 3H, Ar-CH_3); ^{13}C NMR (150 MHz, $CDCl_3$): δ = 168.0, 167.0, 165.7, 164.7, 143.6, 138.5, 134.2, 134.1, 133.9, 133.1, 133.0, 131.6, 131.2, 129.7, 129.7, 129.6, 129.0, 128.6, 128.6, 128.2, 128.1, 127.7, 127.4, 126.8, 123.6, 123.6, 86.6, 83.3, 78.0, 72.5, 69.4, 62.3, 54.0, 21.2; HRMS (ESI-TOF) m/e : Calcd for $C_{54}H_{43}NO_8SNa$ $[M+Na]^+$: 888.2602 Found 888.2613.

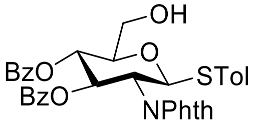

Compound 6. Compound **5** (67.0 g, 77.4 mmol, 1.0 eq.) was dissolved in MeOH/ CH_2Cl_2 = 1/1 (700 mL) at room temperature. *p*TSA (*p*-toluenesulfonic acid, 17.7 g, 92.8 mmol, 1.2 eq.) was added and stirred continuously overnight at room termperature. Et_3N was added to the reaction mixture until the pH reached 7, before the solvent were removed under reduced pressure. Reaction products were fractionated by normal-phase silica gel column chromatography with the eluent EA/hexane = 2:3 to give compound **6** as a white powder (43.9 g, 91%). White solid; R_f = 0.36 (silica gel, EtOAc : hexane 2 : 3); ^{1}H NMR (600 MHz, $CDCl_3$): δ 7.89-7.86 (m, 3H, Ar-H), 7.72-7.64 (m, 5H, Ar-H), 7.49-7.46 (m, 1H, Ar-H), 7.40-7.37 (m, 1H, Ar-H), 7.35-7.32 (m, 4H, Ar-H), 7.24-7.21(m, 2H, Ar-H), 7.09 (d, J = 8.4 Hz, 2H, Ar-H), 6.29 (dd, J = 10.2, 9.6 Hz, 1H, CH), 5.82 (d, J = 10.2 Hz, 1H, C1-H_β), 5.47 (dd, J = 10.2, 10.2 Hz, 1H, CH), 4.55 (dd, J = 10.8, 10.2 Hz, 1H), 3.90 (ddd, J = 9.6, 3.6, 2.4 Hz, 1H), 3.84 (dd, J = 12.0, 2.4 Hz, 1H), 3.71 (dd, J = 12.0, 3.6 Hz, 1H), 2.49 (br, 1H, OH), 2.31 (s, 3H, Ar-CH_3); ^{13}C NMR (150 MHz, $CDCl_3$): δ = 168.0, 166.9, 165.9, 165.6, 138.8, 134.3, 134.2, 133.8, 133.2, 131.6, 131.2, 129.9, 129.8, 129.7, 128.5, 128.5, 128.4, 128.3, 127.0, 123.6, 83.6, 71.8, 69.7, 61.6, 53.9, 21.2; HRMS (ESI-TOF) m/e : Calcd for $C_{35}H_{29}NO_8SNa$ $[M+Na]^+$: 646.1506 Found 646.1533.

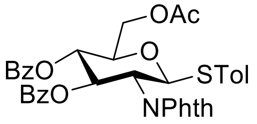

Compound 7. Compound **6** (30.0 g, 48.1 mmol, 1.0 eq.) was dissolved in CH_2Cl_2 (250 mL) and pyridine (7.75 mL, 96.2 mmol, 2 eq.) was added at room temperature. To the mixture Ac_2O (acetic

anhydride, 9.08 mL, 96.2 mmol, 2 eq.) and the cat. DMAP (587 mg, 4.81 mmol, 0.10 eq.) was added and stirred at room temperature for 2 h. The solvent were removed under reduced pressure and the residue purified by normal phase silica gel column chromatography with the eluent EA/hexane = 1:2 to give compound **7** as a white powder (31.7 g, 99%). White solid; R_f = 0.50 (silica gel, EtOAc : hexane 2 : 3); ^{1}H NMR (600 MHz, $CDCl_3$): δ 7.87-7.85 (m, 3H, Ar-H), 7.72-7.65 (m, 5H, Ar-H), 7.47-7.44 (m, 1H, Ar-H), 7.39-7.36 (m, 1H, Ar-H), 7.34-7.30 (m, 4H, Ar-H), 7.23-7.21(m, 2H, Ar-H), 7.08 (d, *J* = 7.8 Hz, 2H, Ar-H), 6.22 (dd, *J* = 10.2, 9.6 Hz, 1H, CH), 5.80 (d, *J* = 10.2 Hz, 1H, C1-H_β), 5.55 (dd, *J* = 9.6, 9.6 Hz, 1H, CH), 4.55 (dd, *J* = 10.2, 9.6 Hz, 1H), 4.31-4.30 (m, 2H), 4.12 (ddd, *J* = 10.2, 0.8, 0.8 Hz, 1H), 2.32 (s, 3H, Ar-CH_3), 2.04 (s, 3H, OAc); ^{13}C NMR (150 MHz, $CDCl_3$): δ = 170.5, 168.0, 166.9, 165.6, 165.1, 138.7, 134.3, 134.2, 133.8, 133.4, 133.2, 131.6, 131.1, 129.7, 129.7, 129.6, 128.7, 128.4, 128.4, 128.2, 127.1, 123.6, 83.4, 76.0, 72.0, 69.6, 62.6, 53.8, 21.2, 20.7; HRMS (ESI-TOF) m/e : Calcd for $C_{37}H_{31}NO_9SNa$ $[M+Na]^+$: 688.1612 Found 688.1645.

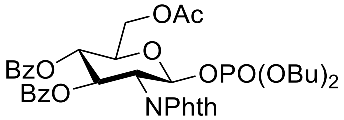

.

Compound 8. Compound **7** (29 g, 43.6 mmol, 1.0 eq.), dibutyl phosphate (34.6 mL, 174 mmol, 4 eq.) and activated pulverized MS-4Å in dry CH_2Cl_2 (600 mL) were stirred at room temperature for 2 h. The solution was cooled to 0°C and *N*-iodosuccinimide (29.4 g, 131 mmol, 3 eq.) and 0.5 M trifluoromethanesulfonic acid were sequentially added in ether (26.1 mL, 13.1 mmol, 0.3 eq.). The reaction mixture was stirred at 0°C overnight. After the reaction was complete, the solution was filtered through celite. The filtrate was then quenched with 20% $Na_2S_2O_3$ (aq.), and washed with saturated $NaHCO_3$ (aq.) and brine. The organic layer was dried over $MgSO_4$ and concentrated. The residue was purified by normal-phase silica gel column chromatography with the eluent EA/hexane = 2 :3 to give compound **8** as a white foam (31.1 g, 95%). White solid; R_f = 0.52 (silica gel, EtOAc : hexane 1 : 1); ^{1}H NMR (600 MHz, $CDCl_3$): δ 7.87 (d, *J* = 7.8 Hz, 2H, Ar-H), 7.84-7.79 (m, 2H, Ar-H), 7.72 (d, *J* = 7.8 Hz, 2H, Ar-H), 7.66 (br, 2H, Ar-H), 7.46 (dd, *J* = 7.8, 7.2 Hz, 1H, Ar-H), 7.38 (dd, *J* = 7.8, 7.2 Hz, 1H, Ar-H), 7.32 (dd, *J* = 7.8, 7.2 Hz, 2H, Ar-H), 7.23 (dd, *J* = 7.8, 7.2 Hz, 2H, Ar-H), 6.30 (dd, *J* = 10.2, 9.6 Hz, 1H, CH), 6.18 (dd, *J* = 8.4, 7.8 Hz, 1H, C1-H_β), 5.62 (dd, *J* = 10.2, 9.6 Hz, 1H, CH), 4.62 (dd, *J* = 9.6, 9.6 Hz, 1H), 4.32 (dd, *J* = 12.0, 4.8 Hz, 1H), 4.27 (d, *J* = 9.6 Hz, 1H), 4.22-4.20 (m, 1H), 4.02-3.95 (m, 2H), 3.80-3.71 (m, 2H), 2.03 (s, 3H, OAc), 1.58-1.54 (m, 2H, CH_{2Bu}), 1.34-1.28 (m, 4H, CH_{2Bu}), 1.09-1.03 (m, 2H, CH_{2Bu}), 0.86 (t, *J* = 7.8 Hz, 3H, CH_{3Bu}), 0.70 (t, *J* = 7.8 Hz, 3H, CH_{3Bu}); ^{13}C NMR (150 MHz, $CDCl_3$): δ = 170.4, 167.7, 167.4, 165.4, 165.1, 134.3, 133.5, 133.3, 131.3, 129.8, 129.7, 128.6, 128.4, 128.4, 128.3, 123.6, 93.8, 93.8, 72.6, 70.4, 69.2, 68.1, 68.1, 67.9, 67.9, 62.0, 55.1, 55.1, 32.0, 31.9, 31.8, 31.8, 20.6, 18.5, 18.3, 13.5, 13.3; HRMS (ESI-TOF) m/e : Calcd for $C_{38}H_{42}NO_{13}PNa$ $[M+Na]^+$: 774.2286 Found 774.2318.

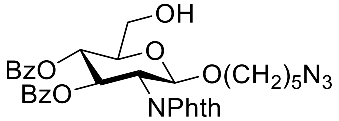

Compound 9. Compound **8** (16.3 g, 21.7 mmol, 1.0 eq.), 5-azidopentan-1-ol (5.60 g, 43.4 mmol, 2 eq.) and activated pulverized MS-4Å in dry CH_2Cl_2 (360 mL). The reaction mixture was stirred at room temperature for 2 h. The solution was cooled to -40 ºC and trimethylsilyl trifluoromethanesulfonate (6.69 mL, 36.9 mmol, 1.7 eq.) added. After stirring at -40 ºC for 1 h, the reaction mixture was warmed to -25 ºC, with an additional reaction occurring for 1 h. After this

reaction had completed, the solution was filtered through celite. The filtrate was then quenched with saturated $NaHCO_3$ (aq.) and brine. The organic layer was collected and dried over $MgSO_4$, before being concentrated under reduced presssure. The residue was purified on a normal-phase silica gel chromatography column with the eluent EA/CH_2Cl_2/hexane = 1 : 1 : 3 to give the azido compound as a white powder (13.1 g, 90%). White solid; R_f = 0.54 (silica gel, EtOAc : CH_2Cl_2 : hexane 1 : 2 : 1); ^{1}H NMR (600 MHz, $CDCl_3$): δ 7.88 (d, J = 7.2 Hz, 2H, Ar-H), 7.86-7.76 (m, 2H, Ar-H), 7.73 (d, J = 7.2 Hz, 2H, Ar-H), 7.66 (br, 2H, Ar-H), 7.45 (dd, J = 7.8, 7.2 Hz, 1H, Ar-H), 7.37 (dd, J = 7.8, 7.2 Hz, 1H, Ar-H), 7.31 (dd, J = 7.8, 7.2 Hz, 2H, Ar-H), 7.22 (dd, J = 7.8, 7.2 Hz, 2H, Ar-H), 6.21 (dd, J = 9.6, 9.0 Hz, 1H, CH), 5.60 (dd, J = 9.6, 9.6 Hz, 1H, CH), 5.51 (d, J = 8.4 Hz, 1H, C1-H_β), 4.53 (dd, J = 10.8, 8.4 Hz, 1H), 4.33 (dd, J = 6.6, 4.8 Hz, 1H), 4.26 (dd, J = 12.0, 3.0 Hz, 1H), 4.11-4.08 (m, 1H), 3.89-3.87 (m, 1H), 3.50-3.48 (m, 1H), 2.97-2.91 (m, 2H, $CH_{2linker}$), 2.02 (s, 3H, OAc), 1.52-1.41 (m, 2H, $CH_{2linker}$), 1.35-1.34 (m, 2H, $CH_{2linker}$), 1.20-1.14 (m, 2H, $CH_{2linker}$); ^{13}C NMR (150 MHz, $CDCl_3$): δ = 170.5, 165.6, 165.1, 134.2, 133.3, 131.3, 129.7, 129.6, 128.7, 128.4, 128.3, 128.2, 123.5, 98.2, 71.9, 71.0, 69.8. 69.6, 62.4, 54.7, 51.0, 28.6, 28.2, 22.9, 20.6; HRMS (ESI-TOF) m/e : Calcd for $C_{35}H_{34}N_4O_{10}Na$ $[M+Na]^+$: 693.2167 Found 693.2202.

The azido compound (11.8 g, 17.6 mmol, 1.0 eq.) was dissolved in CH_2Cl_2 (60 mL) and was further diluted with MeOH (180 mL). AcCl (3.51 mL) was then added in an ice-bath. The mixture was kept for 16 h at room temperature and then concentrated. The residue was purified on a normal-phase silica gel chromatography column with the eluent EA/hexane = 2 : 3 to give compound **9** as a white powder (10.7 g, 97%). White solid; R_f = 0.24 (silica gel, EtOAc : hexane 2 : 3); ^{1}H NMR (600 MHz, $CDCl_3$): δ 7.92 (d, J = 7.8 Hz, 1H, Ar-H), 7.90-7.68 (m, 6H, Ar-H), 7.49 (dd, J = 7.8, 7.2 Hz, 1H, Ar-H), 7.39 (dd, J = 7.2, 7.2 Hz, 1H, Ar-H), 7.35 (dd, J = 7.8, 7.8 Hz, 2H, Ar-H), 7.24 (dd, J = 7.8, 7.2 Hz, 2H, Ar-H), 6.30 (dd, J = 10.2, 9.0 Hz, 1H, CH), 5.52-5.48 (m, 2H, CH, C1-H_β), 4.53 (dd, J = 10.2, 9.0 Hz, 1H), 3.91-3.84 (m, 3H), 3.75-3.71 (m, 1H), 3.51-3.47 (m, 1H), 2.99-2.92 (m, 2H, $CH_{2\text{-linker}}$), 2.59 (t, J = 7.8 Hz, 1H, OH), 1.50-1.42 (m, 2H, $CH_{2\text{-linker}}$), 1.40-1.35 (m, 2H, $CH_{2\text{-linker}}$), 1.21-1.14 (m, 2H, $CH_{2\text{-linker}}$); ^{13}C NMR (150 MHz, $CDCl_3$): δ = 166.1, 165.6, 134.2, 133.6, 133.2, 131.3, 129.9, 129.7, 128.6, 128.6, 128.4, 128.3, 123.5, 98.2, 74.4, 70.9, 70.1, 69.6, 61.3, 54.8, 51.1, 28.7, 28.3, 23.0; HRMS (ESI-TOF) m/e : Calcd for $C_{33}H_{32}N_4O_9Na$ $[M+Na]^+$: 651.2061 Found 651.2093.

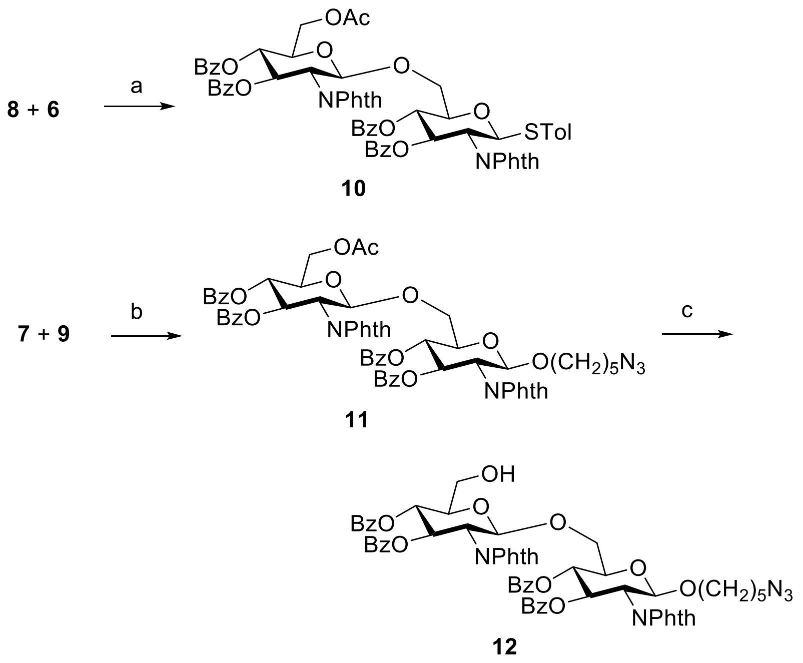

Figure S2. Synthesis of Disaccharide Building Blocks **10** and **11**

Reagents and conditions: (a) TMSOTf, CH_2Cl_2, MS 4Å, -30 to 0 ºC, 2 h, 99%; (b) NIS/TfOH, CH_2Cl_2, MS 4Å, -20 ºC, 95%; (c) AcCl, CH_2Cl_2/MeOH, rt, 16 h, 96%.

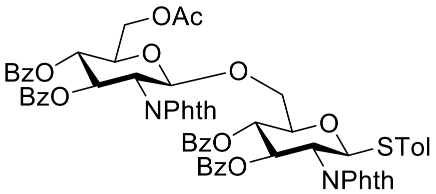

Compound 10. Compound **8** (18.0 g, 24.0 mmol, 1.15 eq.), acceptor **6** (13.0 g, 20.8 mmol, 1.0 eq.) and activated pulverized MS-4Å in dry CH_2Cl_2 (500 mL) were stirred at room temperature for 2 h. The solution was cooled to -30 ºC and trimethylsilyl trifluoromethanesulfonate (4.54 mL, 25.0 mmol, 1.2 eq.) added. After stirring at -30 °C for 1.5 h, the reaction mixture was heated to 0 °C and stirred for an additional 0.5 h to ensure the reaction was complete. The reaction mixture was then filtered through celite, and the filtrate quenched with saturated $NaHCO_{3\,(aq.)}$ and brine. The organic layer was then collected and dried over $MgSO_4$, before being concentrated at a reduced pressure. The residue was purified on a normal-phase silica gel chromatography column with the eluent EA/hexane = 2:3 and on a second column with the eluent EA/CH_2Cl_2/hexane = 1 : 1 : 3 to give compound **10** as a white powder (19.9 g, 99%). White solid; R_f = 0.61 (silica gel, EtOAc : hexane 1 : 1); ^{1}H NMR (600 MHz, $CDCl_3$): δ 7.88 (d, J = 8.4 Hz, 2H, Ar-H), 7.79-7.73 (m, 6H, Ar-H), 7.66-7.56 (m, 6H, Ar-H), 7.52 (br, 2H, Ar-H), 7.44 (dd, J = 7.8, 7.2 Hz, 2H, Ar-H), 7.36 (dd, J = 7.2, 7.2 Hz, 1H, Ar-H), 7.33-7.28 (m, 5H, Ar-H), 7.24-7.20 (m, 4H, Ar-H), 7.16-7.13 (m, 1H, Ar-H), 6.21 (dd, J = 10.2, 9.6 Hz, 1H, CH), 6.17 (dd, J = 10.2, 10.2 Hz, 1H, CH), 5.68-5.67 (m, 2H, 2xC1-$H_β$), 5.24 (dd, J = 9.6, 9.6 Hz, 1H, CH), 5.32 (dd, J = 10.2, 9.6 Hz, 1H, CH), 4.57 (dd, J = 10.2, 9.6 Hz, 1H), 4.43 (dd, J = 10.2, 10.2 Hz, 1H), 4.27 (dd, J = 12.0, 4.8 Hz, 1H), 4.22 (dd, J = 12.0, 2.4 Hz, 1H), 4.13-4.06 (m, 3H), 3.83 (dd, J = 11.4, 5.4 Hz, 1H), 2.36 (s, 3H, Ar-CH_3), 2.00 (s, 3H, OAc); ^{13}C NMR (150 MHz, $CDCl_3$): δ = 170.5, 167.7, 166.7, 165.5, 165.4,

165.0, 165.0, 138.4, 134.1, 134.0, 133.8, 133.5, 133.3, 133.0, 133.0, 131.4, 131.0, 129.6, 129.5, 128.7, 128.6, 128.4, 128.3, 128.2, 128.1, 128.0, 126.8, 123.4, 82.6, 77.2, 71.9, 71.8, 71.0, 69.9, 69.6, 68.9, 62.3, 54.5, 53.6, 21.1, 20.5; HRMS (ESI-TOF) m/e : Calcd for $C_{65}H_{52}N_2O_{17}SNa$ $[M+Na]^+$: 1187.2879 Found 1187.2908.

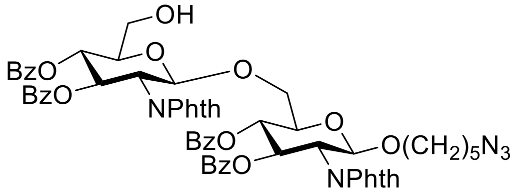

Compound 12. Compound **7** (13.3 g, 20.0 mmol, 1.2 eq.), acceptor **9** (10.5 g, 16.7 mmol, 1.0 eq.) and activated pulverized MS-4Å in dry CH_2Cl_2 (360 mL) were stirred at room temperature for 2 h. The solution was cooled to -20 ºC and *N*-iodosuccinimide (7.52 g, 33.4 mmol, 2 eq.) and 0.5 M trifluoromethanesulfonic acid in ether (6.68 mL, 3.34 mmol, 0.2 eq.) were added sequentially. The reaction mixture was stirred at -20 ºC for 1 h. After the reaction was complete, the solution was filtered through celite. The filtrate was then quenched with 20% $Na_2S_2O_3$ (aq.), and washed with saturated $NaHCO_3$ (aq.) and brine. The organic layer was dried over $MgSO_4$ and concentrated. The residue was fractionated on a normal-phase silica gel chromatography column with the eluent EA/CH_2Cl_2/hexane = 1 : 1 : 2 and on a second column with the eluent EA/hexane = 1 : 1 to give the acetyl disaccharide compound **11** as a white powder (18.5 g, 95%). White solid; R_f = 0.18 (silica gel, EtOAc:hexane 2:3); ^1H NMR (600 MHz, $CDCl_3$): δ 7.87-7.64 (m, 16H, Ar-H), 7.48-7.45 (m, 2H, Ar-H), 7.39-7.30 (m, 6H, Ar-H), 7.23-7.18 (m, 4H, Ar-H), 6.20 (dd, *J* = 10.2, 9.6 Hz, 1H, CH), 6.15 (dd, *J* = 10.2, 9.6 Hz, 1H, CH), 5.61 (d, *J* = 8.4 Hz, 1H, C1-H_β), 5.52 (dd, *J* = 9.6, 9.6 Hz, 1H, CH), 5.37 (d, *J* = 8.4 Hz, 1H, C1-H_β), 5.34 (dd, *J* = 9.6, 9.6 Hz, 1H, CH), 4.53 (dd, *J* = 10.8, 8.4 Hz, 1H), 4.42 (dd, *J* = 10.8, 8.4 Hz, 1H), 4.29 (dd, *J* = 12.0, 5.4 Hz, 1H), 4.19 (dd, *J* = 12.0, 2.4 Hz, 1H), 4.08-4.05 (m, 1H), 4.03-4.00 (m, 2H), 3.82 (dd, *J* = 11.4, 4.8 Hz, 1H), 3.60-3.56 (m, 1H), 3.31-3.27 (m, 1H), 2.95-2.89 (m, 2H, $CH_{2\text{-linker}}$), 1.97 (s, 3H, OAc), 1.36-1.26 (m, 4H, 2x$CH_{2\text{-linker}}$), 1.11-1.06 (m, 2H, $CH_{2\text{-linker}}$); ^{13}C NMR (150 MHz, $CDCl_3$): δ = 170.5, 168.0, 167.1, 165.5, 165.2, 165.1, 134.1, 134.0, 133.4, 133.2, 133.1, 131.5, 131.3, 129.7, 129.7, 129.6, 128.7, 128.7, 128.6, 128.5, 128.3, 128.3, 128.2, 128.1, 123.4, 98.1, 97.8, 71.9, 71.0, 70.9, 70.2, 69.8, 69.1, 68.2, 62.4, 54.8, 54.7, 51.0, 28.6, 28.2, 23.0, 20.5; HRMS (ESI-TOF) m/e : Calcd for $C_{63}H_{55}N_5O_{18}Na$ $[M+Na]^+$: 1192.3434 Found 1192.3489.

Compound **11** (13.4 g, 11.5 mmol, 1.0 eq.) was dissolved in CH_2Cl_2 (70 mL) and diluted with MeOH (210 mL) and then AcCl (2.29 mL) was added with cooling in an ice-bath. The mixture was kept for 16 h at room temperature and then concentrated. The residue was purified on a normal phase silica gel chromatography column with the eluent EA/hexane= 1 : 1 to give compound **12** as a white powder (12.4 g, 96%). White solid; R_f = 0.55 (silica gel, EtOAc : hexane 1 : 1); ^1H NMR (600 MHz, $CDCl_3$): δ 7.89-7.86 (m, 4H, Ar-H), 7.82-7.66 (m, 12H, Ar-H), 7.53-7.50 (m, 1H, Ar-H), 7.49-7.46 (m, 1H, Ar-H), 7.39-7.37 (m, 4H, Ar-H), 7.34-7.32 (m, 2H, Ar-H), 7.25-7.21 (m, 4H, Ar-H), 6.22 (dd, *J* = 10.8, 9.0 Hz, 1H, CH), 6.13 (dd, *J* = 10.8, 9.0 Hz, 1H, CH), 5.58 (d, *J* = 8.4 Hz, 1H, C1-H_β), 5.43 (dd, *J* = 9.6, 9.6 Hz, 1H, CH), 5.38 (d, *J* = 8.4 Hz, 1H, C1-H_β), 5.30 (dd, *J* = 9.6, 9.6 Hz, 1H, CH), 4.44-4.38 (m, 2H), 4.05 (dd, *J* = 10.8, 3.6 Hz, 1H), 4.00 (ddd, *J* = 13.8, 6.0, 3.6 Hz, 1H), 3.85-3.77 (m, 3H), 3.63-3.59 (m, 2H), 3.29 (ddd, *J* = 10.2, 7.2, 5.4 Hz, 1H), 2.95-2.87 (m, 2H, $CH_{2\text{-linker}}$), 2.83 (t, *J* = 7.2 Hz, 1H, OH), 1.35-1.28 (m, 4H, 2x$CH_{2\text{-linker}}$), 1.11-1.05 (m, 2H, $CH_{2\text{-linker}}$); ^{13}C NMR (150 MHz, $CDCl_3$): δ = 168.1, 167.2, 165.8, 165.6, 165.6, 165.6, 134.2, 134.1, 133.5, 133.4, 133.2, 133.2, 131.3, 129.9, 129.9, 129.7, 129.7, 128.9, 128.6, 128.6, 128.5, 128.4, 128.3, 128.2, 123.5, 98.0, 97.6, 74.3, 73.0, 71.1, 70.8, 70.8, 69.8, 69.2, 68.2, 54.8, 54.5,

51.1, 28.7, 28.3, 23.0; HRMS (ESI-TOF) m/e : Calcd for $C_{61}H_{53}N_5O_{17}Na$ $[M+Na]^+$: 1150.3329 Found 1150.3400.

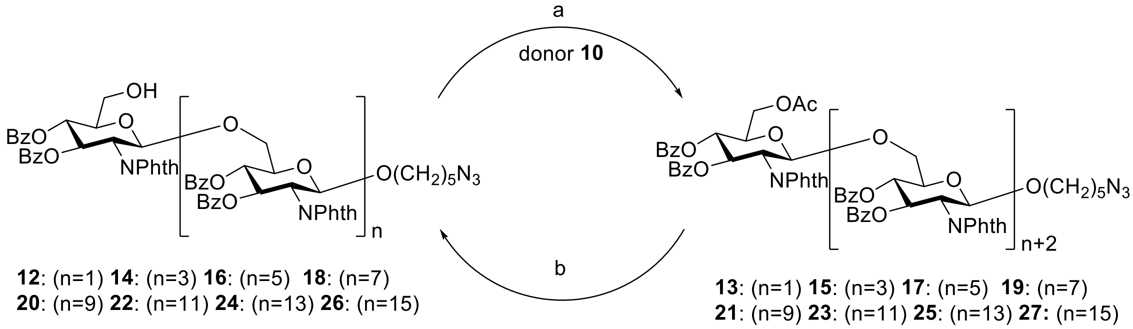

Figure S3. Stepwise Synthesis of Oligoglucosamines

Reagents and conditions: (a) NIS/TfOH, CH_2Cl_2, -30 to -20 ºC, 2 h **13**: (n=1) 84%, **15**: (n=3) 57%, **17**: (n=5) 59%, **19**: (n=7) 56%, **21**: (n=9) 48%, **23**: (n=11) 50%, **25**: (n=13) 48%, **27**: (n=15) 59%; (b) AcCl, CH_2Cl_2/MeOH, rt, 1-2 d **14**: (n=3) 84%, **16**: (n=5) 94%, **18**: (n=7) 92%, **20**: (n=9) 93%, **22**: (n=11) 94%, **24**: (n=13) 90%, **26**: (n=15) 88%.

General Method A. General procedure for NIS–TfOH-catalyzed glycosylation

Thioglycoside donor and acceptor (1 eq.) and activated pulverized MS-4Å in dry CH_2Cl_2 were stirred at room temperature for 2 h. The solution was cooled to -30 ºC and *N*-iodosuccinimide and 0.5 M trifluoromethanesulfonic acid in ether were added sequentially. After stirring at -30 ºC for 1 h, the reaction mixture was warmed to -20 ºC for another 1 h. After the reaction was complete, the solution was filtered through celite. The filtrate was then quenched with 20% $Na_2S_2O_{3\,(aq.)}$, and washed with saturated $NaHCO_{3\,(aq.)}$ and brine. The organic layer was dried over $MgSO_4$ and concentrated. The residue was purified by normal phase silica gel column chromatography.

General Method B. General procedure for selective deacetylation in the presence of benzoyl groups

The acetyl compound (1.0 eq.) was dissolved in CH_2Cl_2 and diluted with MeOH and then AcCl was added with cooling in an ice-bath. The mixture was kept for 1-2 days at room temperature and then concentrated. The residue was purified by normal-phase silica gel column chromatography.

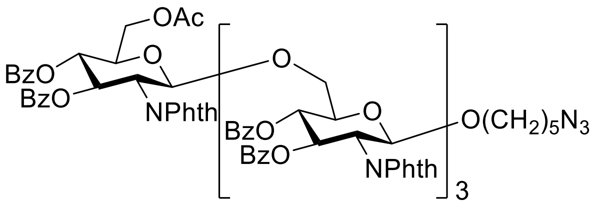

Compound 13. This compound was synthesized according to **General Method A** from donor **10** (12.4 g, 10.6 mmol, 1.2 eq.), acceptor **12** (10.0 g, 8.86 mmol, 1 eq.), *N*-iodosuccinimide (3.99 g, 17.7 mmol, 2 eq.) and 0.5 M trifluoromethanesulfonic acid in ether (5.32 mL, 2.66 mmol, 0.3 eq.) in CH_2Cl_2 (280 mL). The residue was purified on a normal-phase silica gel chromatography column with the eluent EA/hexane = 1:1 and on a second column with the eluent EA/CH_2Cl_2/hexane = 1 : 1 : 2 to give compound **13** as a white powder (16.1 g, 84%). White solid; R_f = 0.15 (silica gel, EtOAc : CH_2Cl_2 : hexane 1 : 1 : 2); ^{1}H NMR (600 MHz, $CDCl_3$): δ 7.88-7.63 (m, 32H, Ar-H), 7.42-7.20 (m, 22H, Ar-H), 7.14 (dd, *J* = 7.8, 7.8 Hz, 2H, Ar-H), 6.28 (dd, *J* = 10.2,

9.0 Hz, 1H, CH), 6.15 (dd, *J* = 10.2, 9.0 Hz, 1H, CH), 6.08-6.04 (m, 2H, 2xCH), 5.66 (d, *J* = 8.4 Hz, 1H, C1-$H_β$), 5.50 (dd, *J* = 9.6, 9.6 Hz, 1H, CH), 5.46 (d, *J* = 8.4 Hz, 1H, C1-$H_β$), 5.38 (d, *J* = 8.4 Hz, 1H, C1-$H_β$), 5.35-5.31 (m, 2H, CH, C1-$H_β$), 5.22 (dd, *J* = 9.6, 9.6 Hz, 1H, CH), 5.00 (dd, *J* = 9.6, 9.6 Hz, 1H, CH), 4.46-4.40 (m, 3H), 4.28-4.22 (m, 2H), 4.19-4.15 (m, 1H), 3.92-3.89 (m, 4H), 3.84 (dd, *J* = 10.2, 4.2 Hz, 1H), 3.78-3.75 (m, 2H), 3.74-3.71 (m, 1H), 3.70-3.66 (m, 1H), 3.52 (dd, *J* = 10.8, 4.2 Hz, 1H), 3.35 (ddd, *J* = 10.2, 7.2, 5.4 Hz, 1H), 2.93-2.85 (m, 2H, $CH_{2\text{-linker}}$), 1.94 (s, 3H, OAc), 1.35-1.29 (m, 4H, 2x$CH_{2\text{-linker}}$), 1.11-1.08 (m, 2H, $CH_{2\text{-linker}}$); ^{13}C NMR (150 MHz, $CDCl_3$): δ = 170.6, 168.0, 167.8, 167.6, 167.2, 165.6, 165.5, 165.4, 165.2, 165.1, 165.0, 164.7, 134.2, 134.1, 133.9, 133.3, 133.1, 133.0, 131.6, 131.5, 131.3, 129.9, 129.8, 129.8, 129.7, 129.7, 129.6, 129.6, 129.2, 128.8, 128.8, 128.6, 128.6, 128.6, 128.6, 128.6, 128.4, 128.3, 128.2, 128.1, 128.1, 128.1, 123.6, 123.5, 123.4, 123.4, 97.9, 97.9, 97.0, 73.5, 73.1, 72.6, 71.7, 71.1, 71.0, 71.0, 70.8, 70.4, 70.1, 70.1, 69.8, 67.4, 67.6, 67.1, 62.5, 54.8, 54.8, 54.7, 54.5, 51.0, 28.6, 28.3, 23.0; HRMS (ESI-TOF) m/e : Calcd for $C_{119}H_{97}N_7O_{34}Na_2$ $[M+2Na]^{2+}$: 1106.7930 Found 1106.7998.

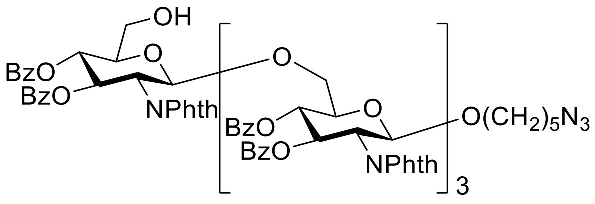

Compound 14. This compound was synthesized according to **General Method B**, from acetyl compound **13** (15.2 g, 7.01 mmol, 1 eq.). To this was added AcCl (1.40 mL) in CH_2Cl_2 (90 mL) and MeOH (120 mL) for 1 day. The residue was purified by normal-phase silica gel column chromatography with the eluent EA/CH_2Cl_2/toluene = 1 : 4 : 4 to give compound **14** as a white powder (12.5 g, 84%). White solid; R_f = 0.36 (silica gel, EtOAc : CH_2Cl_2 : toluene 3 : 1 : 3); ^1H NMR (600 MHz, $CDCl_3$): δ 7.90-7.64 (m, 32H, Ar-H), 7.51-7.43 (m, 4H, Ar-H), 7.42-7.28 (m, 12H, Ar-H), 7.25-7.16 (m, 8H, Ar-H), 6.24 (dd, *J* = 10.2, 9.0 Hz, 1H, CH), 6.12 (dd, *J* = 10.8, 9.0 Hz, 1H, CH), 6.07-6.03 (m, 2H, 2xCH), 5.58 (d, *J* = 8.4 Hz, 1H, C1-$H_β$), 5.45 (d, *J* = 8.4 Hz, 1H, C1-$H_β$), 5.58-5.35 (m, 2H, 2xC1-$H_β$), 5.31-5.21 (m, 3H, 3xCH), 5.16 (dd, *J* = 9.6, 9.6 Hz, 1H, CH), 4.43-4.39 (m, 2H), 4.31 (dd, *J* = 10.8, 8.4 Hz, 1H), 4.23 (dd, *J* = 10.8, 8.4 Hz, 1H), 3.99 (dd, *J* = 10.8, 3.6 Hz, 1H), 3.94 (dd, *J* = 11.4, 2.4 Hz, 1H), 3.92-3.86 (m, 4H), 3.80-3.75 (m, 3H), 3.72-3.66 (m, 2H), 3.62-3.55 (m, 2H), 3.35 (ddd, *J* = 10.2, 7.2, 5.4 Hz, 1H), 3.02 (t, *J* = 7.2 Hz, 1H, OH), 2.93-2.86 (m, 2H, $CH_{2\text{-linker}}$), 1.38-1.30 (m, 4H, 2x$CH_{2\text{-linker}}$), 1.12-1.07 (m, 2H, $CH_{2\text{-linker}}$); ^{13}C NMR (150 MHz, $CDCl_3$): δ = 168.1, 167.2, 165.6, 165.6, 165.5, 165.5, 165.0, 165.0, 134.1, 134.0, 133.9, 133.4, 133.3, 133.2, 133.1, 131.5, 131.4, 129.9, 129.8, 129.7, 129.7, 129.1, 129.0, 128.9, 128.8, 128.7, 128.6, 128.4, 128.3, 128.2, 128.2, 128.1, 123.6, 123.5, 123.4, 74.1, 73.4, 72.6, 72.4, 71.2, 71.0, 70.9, 70.8, 70.5, 70.2, 69.2, 67.9, 67.7, 67.5, 61.2, 54.8, 54.7, 54.6, 51.1, 28.7, 28.3, 23.0; HRMS (ESI-TOF) m/e : Calcd for $C_{117}H_{95}N_7O_{33}Na_2$ $[M+2Na]^{2+}$: 1085.7878 Found 1085.7940.

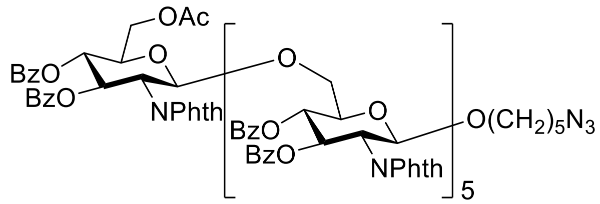

Compound 15. This compound was synthesized according to **General Method A**, from donor **10** (13.3 g, 11.4 mmol, 1.85 eq.), acceptor **14** (13.1 g, 6.16 mmol, 1 eq.), *N*-iodosuccinimide (5.13 g, 22.8 mmol, 3.7 eq.) and 0.5 M trifluoromethanesulfonic acid in ether (6.16 mL, 3.08 mmol, 0.5 eq.) in CH_2Cl_2 (400 mL). The residue was purified by normal-phase silica gel column chromatography with the eluent EA/CH_2Cl_2/toluene = 1 : 4 : 4 and on a second column with the eluent EA/CH_2Cl_2/hexane = 1 : 1 : 1 to give compound **15** as a white powder (11.1 g, 57%). White solid; R_f = 0.56 (silica gel, EtOAc : CH_2Cl_2 : toluene 3 : 1 : 3); ^{1}H NMR (600 MHz, $CDCl_3$): δ 7.88-7.60 (m, 48H, Ar-H), 7.48-7.28 (m, 20H, Ar-H), 7.25-7.20 (m, 10H, Ar-H), 7.16-7.11 (m, 6H, Ar-H), 6.27 (dd, *J* = 10.2, 9.0 Hz, 1H, CH), 6.14-6.06 (m, 5H, 5xCH), 5.63 (d, *J* = 8.4 Hz, 1H, C1-H_β), 5.48 (dd, *J* = 9.6, 9.6 Hz, 1H, CH), 5.44-5.36 (m, 5H, 5xC1-H_β), 5.33 (dd, *J* = 9.6, 9.6 Hz, 1H, CH), 5.23-5.18 (m, 2H, 2xCH), 5.12-5.06 (m, 2H, 2xCH), 4.45-4.40 (m, 3H), 4.34-4.24 (m, 4H), 4.18-4.12 (m, 2H), 3.98-3.92 (m, 3H), 3.86-3.70 (m, 9H), 3.68-3.62 (m, 3H), 3.58-3.56 (m, 1H), 3.33 (ddd, *J* = 10.2, 7.8, 6.0 Hz, 1H), 2.91-2.82 (m, 2H, 2x$CH_{2\text{-linker}}$), 1.93 (s, 3H, OAc), 1.35-1.27 (m, 4H, 4x$CH_{2\text{-linker}}$), 1.09-1.05 (m, 2H, 2x$CH_{2\text{-linker}}$); ^{13}C NMR (150 MHz, $CDCl_3$): δ = 170.6, 168.0, 167.2, 165.6, 165.5, 165.4, 165.2, 165.1, 165.0, 164.9, 164.9, 164.8, 134.1, 133.9, 133.8, 133.2, 133.2, 133.1, 133.1, 133.0, 131.7, 131.3, 129.8, 129.8, 129.7, 129.7, 129.7, 129.6, 129.2, 129.0, 129.0, 128.8, 128.8, 128.7, 128.6, 128.6, 128.5, 128.4, 128.3, 128.3, 128.2, 128.2, 128.1, 128.1, 128.1, 123.5, 123.4, 123.4, 97.8, 97.8, 97.6, 97.3, 97.2, 73.5, 73.0, 73.0, 73.0, 72.5, 71.7, 71.1, 71.0, 71.0, 70.9, 70.9, 70.8, 70.4, 70.2, 70.1, 69.9, 69.8, 69.1, 67.6, 67.2, 67.2, 67.0, 66.7, 62.5, 54.8, 54.7, 54.7, 54.6, 51.0, 28.6, 28.2, 23.0, 20.6; HRMS (ESI-TOF) m/e : Calcd for $C_{175}H_{139}N_9O_{50}Na_2$ $[M+2Na]^{2+}$: 1605.9198 Found 1605.9215.

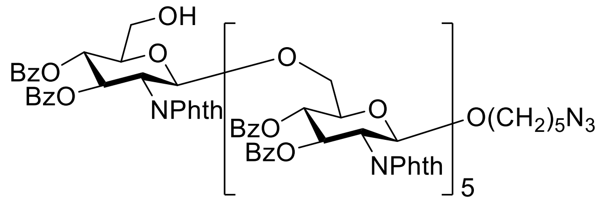

Compound 16. This compound was synthesized according to **General Method B**, from acetyl compound **15** (11.0 g, 3.47 mmol, 1 eq.). Compound **15** was mixed with AcCl (1.41 mL) in CH_2Cl_2 (40 mL) and MeOH (40 mL) for 1 day. The residue was purified by normal-phase silica gel column chromatography with the eluent EA/CH_2Cl_2/toluene = 1 : 3 : 3, to give compound **16** as a white powder (10.2 g, 94%). White solid; R_f = 0.42 (silica gel, EtOAc : CH_2Cl_2 : toluene 3 : 1 : 3); ^{1}H NMR (600 MHz, $CDCl_3$): δ 7.90-7.62 (m, 48H, Ar-H), 7.54-7.47 (m, 6H, Ar-H), 7.43-7.31 (m, 18H, Ar-H), 7.28-7.16 (m, 12H, Ar-H), 6.26 (dd, *J* = 10.8, 9.6 Hz, 1H, CH), 6.18-6.09 (m, 5H, 5xCH), 5.61 (d, *J* = 8.4 Hz, 1H, C1-H_β), 5.49-5.41 (m, 5H, 5xC1-H_β), 5.37 (dd, *J* = 9.6, 9.6 Hz, 1H, CH), 5.31-5.21 (m, 4H, 4xCH), 5.13 (dd, *J* = 9.6, 9.6 Hz, 1H, CH), 4.49-4.45 (m, 2H), 4.36-4.27 (m, 4H), 4.03-3.97 (m, 3H), 3.92-3.69 (m, 12H), 3.66-3.62 (m, 4H), 3.37 (ddd, *J* = 10.2, 7.2, 5.4 Hz, 1H), 3.12 (t, *J* = 7.2 Hz, 1H, OH), 2.96-2.88 (m, 2H, $CH_{2\text{-linker}}$), 1.41-1.31 (m, 4H, 2x$CH_{2\text{-linker}}$), 1.14-1.09 (m, 2H, $CH_{2\text{-linker}}$); ^{13}C NMR (150 MHz, $CDCl_3$): δ = 168.0, 167.1, 165.5, 165.4, 165.4, 165.4, 165.3, 165.0, 164.9, 164.8, 164.7, 134.0, 133.8, 133.3, 133.1, 133.0, 132.9, 131.2,

129.8, 129.7, 129.6, 129.6, 129.5, 129.0, 128.9, 128.9, 128.9, 128.7, 128.7, 128.6, 128.6, 128.5, 128.4, 128.3, 128.2, 128.2, 128.1, 128.1, 128.0, 123.4, 123.3, 123.3, 74.0, 73.3, 72.9, 72.7, 72.4, 72.2, 71.1, 70.9, 70.8, 70.7, 70.4, 70.2, 70.0, 69.8, 69.6, 69.0, 67.7, 67.4, 67.2, 67.1, 66.7, 61.1, 54.7, 54.6, 54.6, 54.5, 54.4, 50.9, 28.5, 28.2, 22.9; HRMS (ESI-TOF) m/e : Calcd for $C_{173}H_{137}N_9O_{49}Na_2$ $[M+2Na]^{2+}$: 1584.9145 Found 1584.9184.

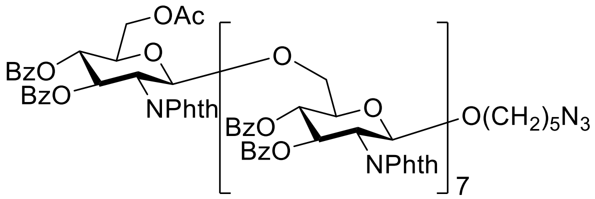

Compound 17. This compound was synthesized according to **General Method A**, from donor **10** (10.4 g, 8.90 mmol, 2.7 eq.), acceptor **16** (10.3 g, 3.29 mmol, 1 eq.), *N*-iodosuccinimide (4.00 g, 17.8 mmol, 5.4 eq.) and 0.5 M trifluoromethanesulfonic acid in ether (3.95 mL, 1.98 mmol, 0.6 eq.) in CH_2Cl_2 (300 mL). The residue was purified on a normal-phase silica gel chromatography column with the eluent EA/CH_2Cl_2/toluene = 1 : 4 : 4 and on a second column with the eluent EA/CH_2Cl_2/hexane = 1 : 1 : 1 to give compound **17** as a white powder (8.10 g, 59%). White solid; R_f = 0.55 (silica gel, EtOAc : CH_2Cl_2 : toluene 3 : 1 : 3); ^{1}H NMR (600 MHz, $CDCl_3$): δ 7.89-7.60 (m, 64H, Ar-H), 7.47-7.41 (m, 8H, Ar-H), 7.38-7.09 (m, 40H, Ar-H), 6.25 (dd, *J* = 10.2, 9.6 Hz, 1H, CH), 6.15-6.05 (m, 7H, 7xCH), 5.61 (d, *J* = 8.4 Hz, 1H, C1-$H_β$), 5.48-5.32 (m, 9H, 2xCH, 7xC1-$H_β$), 5.25 (dd, *J* = 9.6, 9.6 Hz, 1H, CH), 5.18-5.04 (m, 5H, 5xCH), 4.42-4.39 (m, 3H), 4.33-4.21 (m, 6H), 4.13 (dd, *J* = 12.0, 2.4 Hz, 1H), 4.11 (ddd, *J* = 9.6, 5.4, 3.0 Hz, 1H), 3.97-3.92 (m, 3H), 3.86-3.58 (m, 19H), 3.33 (ddd, *J* = 12.6, 10.8, 7.2 Hz, 1H), 2.91-2.82 (m, 2H, $CH_{2\text{-linker}}$), 1.92 (s, 3H, OAc), 1.35-1.25 (m, 4H, 2x$CH_{2\text{-linker}}$), 1.09-1.04 (m, 2H, $CH_{2\text{-linker}}$); ^{13}C NMR (150 MHz, $CDCl_3$): δ = 170.6, 168.0, 167.2, 165.6, 165.5, 165.4, 165.4, 165.4, 165.3, 165.2, 165.1, 165.1, 165.1, 164.9, 164.9, 164.9, 164.8, 164.8, 134.1, 134.0, 133.2, 133.1, 133.0, 133.0, 132.9, 131.8, 131.6, 131.3, 129.8, 129.8, 129.7, 129.7, 129.6, 129.2, 129.0, 129.0, 128.9, 128.8, 128.8, 128.8, 128.7, 128.7, 128.7, 128.6, 128.5, 128.3, 128.2, 128.1, 128.1, 123.5, 123.4, 123.4, 97.9, 97.8, 97.6, 97.6, 97.4, 97.1, 73.5, 73.1, 72.9, 72.9, 72.8, 72.5, 71.7, 71.1, 71.0, 70.9, 70.9, 70.8, 70.4, 70.2, 70.1, 70.0, 69.9, 69.8, 69.1, 67.6, 67.2, 67.0, 66.7, 62.5, 54.8, 54.7, 54.6, 54.5, 51.0, 28.6, 28.2, 23.0, 20.5; HRMS (ESI-TOF) m/e : Calcd for $C_{231}H_{181}N_{11}O_{66}Na_2$ $[M+2Na]^{2+}$: 2105.0465 Found 2105.0547.

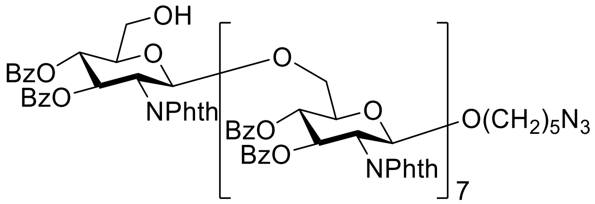

Compound 18. This compound was synthesized according to **General Method B**, from acetyl compound **17** (5.90 g, 1.42 mmol, 1 eq.) to which was added AcCl (0.57 mL) in CH_2Cl_2 (40 mL) and MeOH (40 mL) for 1.5 days. The residue was purified on a normal-phase silica gel chromatography column with the eluent EA/CH_2Cl_2/toluene = 1:3:3 to give compound **18** as a white powder (5.38 g, 92%). White solid; R_f = 0.36 (silica gel, EtOAc : CH_2Cl_2 : toluene 3 : 1 : 3); ^{1}H NMR (600 MHz, $CDCl_3$): δ 7.90-7.60 (m, 64H, Ar-H), 7.49-7.41 (m, 8H, Ar-H), 7.37-7.09 (m, 40H, Ar-H), 6.22 (dd, *J* = 10.2, 9.6 Hz, 1H, CH), 6.14-6.06 (m, 7H, 7xCH), 5.56 (d, *J* = 8.4 Hz, 1H, C1-$H_β$), 5.46-5.33 (m, 8H, CH, 7xC1-$H_β$), 5.26-5.09 (m, 7H, 7xCH), 4.47-4.42 (m, 2H), 4.34-

4.20 (m, 6H), 3.98-3.56 (m, 25H), 3.35 (ddd, J = 10.2, 6.6, 6.6 Hz, 1H), 3.09 (t, J = 7.2 Hz, 1H, OH), 2.90-2.84 (m, 2H, $CH_{2\text{-linker}}$), 1.36-1.26 (m, 4H, 2x$CH_{2\text{-linker}}$), 1.10-1.05 (m, 2H, $CH_{2\text{-linker}}$); ^{13}C NMR (150 MHz, $CDCl_3$): δ = 168.0, 167.1, 165.6, 165.5, 165.5, 165.5, 165.4, 165.4, 165.1, 165.0, 164.9, 164.8, 164.7, 134.0, 133.8, 133.3, 133.1, 133.0, 132.9, 131.3, 129.8, 129.8, 129.7, 129.6, 129.6, 129.1, 129.0, 128.9, 128.9, 128.8, 128.7, 128.7, 128.6, 128.6, 128.5, 128.3, 128.1, 128.1, 128.0, 123.4, 123.3, 97.8, 97.7, 97.5, 97.4, 97.3, 97.2, 74.0, 73.4, 73.0, 72.9, 72.7, 72.4, 72.1, 71.1, 70.9, 70.9, 70.8, 70.8, 70.2, 70.0, 69.8, 69.6, 69.0, 67.8, 67.5, 67.1, 67.0, 66.8, 66.7, 61.1, 54.7, 54.7, 54.5, 54.4, 51.0, 28.6, 28.2, 23.0; HRMS (ESI-TOF) m/e : Calcd for $C_{229}H_{179}N_{11}O_{65}Na_2$ $[M+2Na]^{2+}$: 2084.0412 Found 2084.0453.

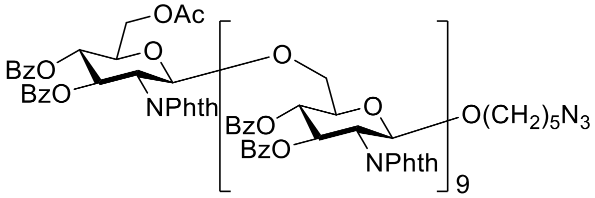

Compound 19. This compound was synthesized according to **General Method A**, from donor **10** (5.36 g, 4.60 mmol, 3.55 eq.), acceptor **18** (5.35 g, 1.30 mmol, 1 eq.), *N*-iodosuccinimide (2.07 g, 9.21 mmol, 7.1 eq.) and 0.5 M trifluoromethanesulfonic acid in ether (1.30 mL, 0.648 mmol, 0.5 eq.) in CH_2Cl_2 (180 mL). The residue was purified on a normal-phase silica gel column chromatography with the eluent EA/CH_2Cl_2/toluene = 1 : 3 : 3 and on a second column with the eluent EA/CH_2Cl_2/hexane = 1 : 1 : 1 to give compound **26** as a white powder (3.75 g, 56%). White solid; R_f = 0.52 (silica gel, EtOAc : CH_2Cl_2 : toluene 3 : 1 : 3); ^1H NMR (600 MHz, $CDCl_3$): δ 7.89-7.09 (m, 140H, Ar-H), 6.25 (dd, J = 10.2, 9.6 Hz, 1H, CH), 6.12-6.06 (m, 9H, 9xCH), 5.61 (d, J = 8.4 Hz, 1H, C1-$H_β$), 5.48-5.32 (m, 11H, 2xCH, 9x C1-$H_β$), 5.25-5.04 (m, 8H, 8xCH), 4.46-4.40 (m, 3H), 4.33-4.13 (m, 10H), 3.98-3.93 (m, 3H), 3.85-3.52 (m, 25H), 3.35 (br, 1H), 2.89-2.85 (m, 2H, $CH_{2\text{-linker}}$), 1.92 (s, 3H, OAc), 1.30-1.25 (m, 4H, 2x$CH_{2\text{-linker}}$), 1.07 (br, 2H, $CH_{2\text{-linker}}$); ^{13}C NMR (150 MHz, $CDCl_3$): δ = 170.6, 168.0, 167.2, 165.6, 165.5, 165.4, 165.4, 165.2, 165.1, 165.1, 165.0, 164.9, 164.8, 164.8, 134.1, 134.0, 133.2, 133.1, 132.9, 131.8, 131.3, 129.9, 129.8, 129.7, 129.7, 129.6, 129.2, 129.0, 128.9, 128.9, 128.9, 128.8, 128.7, 128.7, 128.7, 128.6, 128.5, 128.3, 128.3, 128.2, 128.1, 123.5, 123.4, 123.4, 97.9, 97.8, 97.6, 97.5, 97.5, 97.4, 97.4, 97.1, 73.5, 73.1, 73.0, 72.9, 72.8, 72.7, 72.4, 71.7, 71.1, 71.0, 70.9, 70.8, 69.9, 69.8, 69.1, 67.6, 67.2, 67.1, 67.0, 66.7, 62.5, 54.8, 54.7, 54.6, 54.5, 51.0, 28.6, 28.2, 23.0, 20.5; HRMS (ESI-TOF) m/e : Calcd for $C_{287}H_{223}N_{13}O_{82}Na_3$ $[M+3Na]^{3+}$: 1743.7785 Found 1743.8030.

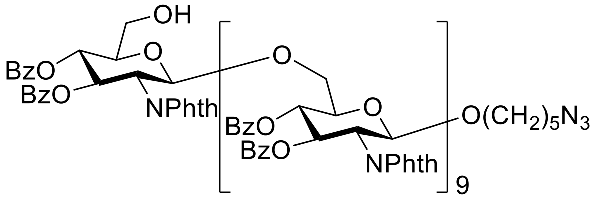

Compound 20. This compound was synthesized according to **General Method B**, from acetyl compound **19** (4.66 g, 0.902 mmol, 1 eq.) to which was added AcCl (0.45 mL) in CH_2Cl_2 (40 mL) and MeOH (40 mL) for 1.5 days. The residue was purified on a normal-phase silica gel chromatography column with the eluent EA/CH_2Cl_2/toluene = 1 : 3 : 3 to give compound **20** as a white powder (4.30 g, 93%). White solid; R_f = 0.35 (silica gel, EtOAc : CH_2Cl_2 : toluene 3 : 1 : 3); ^1H NMR (600 MHz, $CDCl_3$): δ 7.86-7.04 (m, 140H, Ar-H), 6.17 (dd, J = 10.2, 9.6 Hz, 1H, CH), 6.10-6.02 (m, 9H, 9xCH), 5.51 (d, J = 8.4 Hz, 1H, C1-$H_β$), 5.41-5.29 (m, 10H, CH, 9xC1-$H_β$), 5.21-5.05 (m, 9H, 9xCH), 4.41-4.37 (m, 2H), 4.30-4.14 (m, 8H), 3.94-3.49 (m, 31H), 3.30 (ddd, J

= 10.2, 6.6, 6.6 Hz, 1H), 3.05 (t, J = 7.2 Hz, 1H, OH), 2.86-2.78 (m, 2H, $CH_{2\text{-linker}}$), 1.30-1.21 (m, 4H, 2x$CH_{2\text{-linker}}$), 1.05-1.01 (m, 2H, $CH_{2\text{-linker}}$); ^{13}C NMR (150 MHz, $CDCl_3$): δ = 168.0, 167.2, 165.6, 165.5, 165.5, 165.5, 165.1, 165.0, 164.9, 164.9, 164.8, 164.8, 134.0, 133.3, 133.2, 133.1, 133.0, 133.0, 132.9, 131.7, 131.3, 129.9, 129.8, 129.7, 129.6, 129.6, 129.6, 129.1, 129.0, 129.0, 129.0, 128.9, 128.8, 128.7, 128.6, 128.6, 128.5, 128.5, 128.3, 128.1, 128.1, 128.0, 123.5, 123.3, 97.8, 97.8, 97.5, 97.4, 97.4, 97.3, 97.2, 74.1, 73.4, 73.1, 72.9, 72.8, 72.7, 72.6, 72.3, 72.1, 71.1, 70.9, 70.9, 70.6, 70.3, 70.1, 70.0, 69.9, 69.6, 69.0, 67.8, 67.6, 67.1, 66.9, 66.8, 66.7, 61.1, 54.7, 54.7, 54.5, 54.4, 51.0, 28.6, 28.2, 23.0; HRMS (ESI-TOF) m/e : Calcd for $C_{285}H_{221}N_{13}O_{81}Na_2$ $[M+2Na]^{2+}$: 2583.1679 Found 2583.1900.

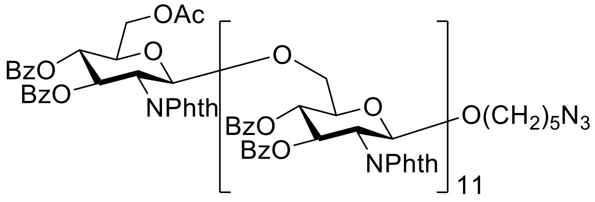

Compound 21. This compound was synthesized according to **General Method A**, from donor **10** (4.24 g, 3.64 mmol, 4.4 eq.), acceptor **20** (4.24 g, 0.828 mmol, 1 eq.), *N*-iodosuccinimide (1.64 g, 4.17 mmol, 8.8 eq.) and 0.5 M trifluoromethanesulfonic acid in ether (0.99 mL, 0.497 mmol, 0.6 eq.) in CH_2Cl_2 (120 mL). The residue was purified on a normal-phase silica gel chromatography column with the eluent EA/CH_2Cl_2/toluene = 1 : 3 : 3 and on a second column with the eluent EA/CH_2Cl_2/hexane = 1 : 1 : 1 to give compound **21** as a white powder (2.44 g, 48%). White solid; R_f = 0.50 (silica gel, EtOAc : CH_2Cl_2 : toluene 3 : 1 : 3); ^{1}H NMR (600 MHz, $CDCl_3$): δ 7.84-7.03 (m, 168H, Ar-H), 6.20 (dd, J = 10.2, 9.6 Hz, 1H, CH), 6.10-6.01 (m, 11H, 11xCH), 5.66 (d, J = 8.4 Hz, 1H, C1-H_β), 5.43-5.28 (m, 13H, 2xCH, 12xC1-H_β), 5.21-4.99 (m, 10H, 10xCH), 4.42-4.35 (m, 3H), 4.30-4.06 (m, 12H), 3.93-3.88 (m, 3H), 3.81-3.47 (m, 31H), 3.29 (ddd, J = 10.2, 6.6, 6.6 Hz, 1H), 2.86-2.77 (m, 2H, $CH_{2\text{-linker}}$), 1.88 (s, 3H, OAc), 1.31-1.22 (m, 4H, 2x$CH_{2\text{-linker}}$), 1.05-1.00 (m, 2H, $CH_{2\text{-linker}}$); ^{13}C NMR (150 MHz, $CDCl_3$): δ = 170.6, 168.0, 167.2, 165.5, 165.5, 165.4, 165.4, 165.2, 165.1, 165.0, 165.0, 164.9, 164.8, 164.8, 134.1, 134.0, 133.2, 132.9, 131.7, 131.3, 129.9, 129.8, 129.7, 129.7, 129.2, 129.0, 128.9, 128.8, 128.8, 128.7, 128.7, 128.6, 128.5, 128.3, 128.3, 128.2, 128.0, 123.5, 123.4, 123.4, 97.9, 97.8, 97.6, 97.5, 97.4, 97.4, 97.1, 73.5, 73.1, 72.9, 72.7, 72.6, 72.4, 71.7, 71.1, 70.9, 70.4, 70.2, 70.1, 69.9, 69.8, 69.1, 67.6, 67.1, 66.8, 66.7, 62.5, 54.7, 54.6, 54.5, 51.0, 28.6, 28.2, 23.0, 20.5; HRMS (ESI-TOF) m/e : Calcd for $C_{343}H_{265}N_{15}O_{98}Na_2$ $[M+2Na]^{2+}$: 3103.2999 Found 3103.3331.

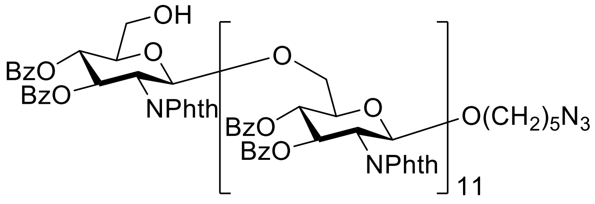

Compound 22. This compound was synthesized according to **General Method B** from acetyl compound **21** (2.40 g, 0.389 mmol, 1 eq.) to which was added AcCl (0.23 mL) in CH_2Cl_2 (35 mL) and MeOH (35 mL) for 2 days. The residue was purified by normal-phase silica gel column chromatography with the eluent EA/CH_2Cl_2/toluene = 1 : 2.5 : 2.5 to give compound **22** as a white powder (2.25 g, 94%). White solid; R_f = 0.29 (silica gel, EtOAc : CH_2Cl_2 : toluene 3 : 1 : 3); ^{1}H NMR (600 MHz, $CDCl_3$): δ 7.91-7.07 (m, 168H, Ar-H), 6.22 (dd, J = 10.2, 9.6 Hz, 1H, CH), 6.16-6.07 (m, 11H, 11xCH), 5.56 (d, J = 8.4 Hz, 1H, C1-H_β), 5.47-5.35 (m, 12H, CH, 11xC1-H_β), 5.28-

5.10 (m, 11H, 11xCH), 4.48-4.43 (m, 2H), 4.36-4.20 (m, 10H), 4.00-3.54 (m, 37H), 3.36 (ddd, *J* = 10.2, 6.6, 6.6 Hz, 1H), 3.08 (br, 1H, OH), 2.91-2.83 (m, 2H, $CH_{2\text{-linker}}$), 1.37-1.27 (m, 4H, $2xCH_{2\text{-linker}}$), 1.11-1.05 (m, 2H, $CH_{2\text{-linker}}$); ^{13}C NMR (150 MHz, $CDCl_3$): δ = 168.0, 167.1, 165.5, 165.5, 165.5, 165.4, 165.4, 165.4, 165.3, 165.1, 165.0, 164.9, 164.9, 164.8, 164.7, 134.0, 133.3, 133.1, 132.9, 131.7, 131.2, 129.8, 129.7, 129.6, 129.1, 129.0, 129.0, 128.9, 128.7, 128.7, 128.6, 128.6, 128.5, 128.4, 128.3, 128.1, 128.1, 128.0, 123.4, 123.3, 97.8, 97.7, 97.5, 97.4, 97.4, 97.3, 91.2, 74.0, 73.4, 73.1, 72.9, 72.9, 72.8, 72.7, 72.7, 72.6, 72.5, 72.3, 72.1, 71.1, 70.9, 70.8, 70.6, 70.2, 70.0, 69.8, 69.6, 69.0, 67.8, 67.5, 67.1, 66.9, 66.7, 61.1, 54.6, 54.6, 54.4, 54.4, 51.0, 28.6, 28.2, 23.0; HRMS (ESI-TOF) m/e : Calcd for $C_{341}H_{263}N_{15}O_{97}Na_2$ $[M+2Na]^{2+}$: 3082.2946 Found 3082.3140.

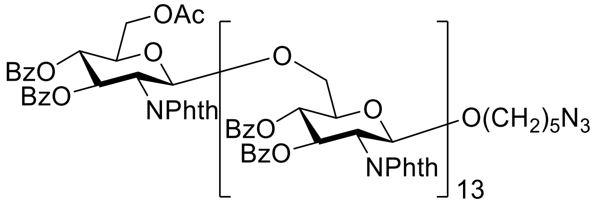

Compound 23. This compound was synthesized according to **General Method A** from donor **10** (1.72 g, 1.48 mmol, 5.2 eq.), acceptor **22** (1.74 g, 0.284 mmol, 1 eq.), *N*-iodosuccinimide (665 mg, 2.96 mmol, 10.4 eq.) and 0.5 M trifluoromethanesulfonic acid in ether (0.34 mL, 0.170 mmol, 0.6 eq.) in CH_2Cl_2 (60 mL). After stirring at -30 °C for 1 h, 0.5 M trifluoromethanesulfonic acid in ether (57 μL, 28.4 μmol, 0.1 eq.) was added to the reaction mixture and warmed to -20 °C for another 1 h. The residue was purified on a normal-phase silica gel chromatography column with the eluent EA/CH_2Cl_2/toluene = 1 : 3 : 3 and on a second chromatography column with the eluent EA/CH_2Cl_2/hexane = 1 : 1 : 1 to give compound **23** as a white powder (1.01 mg, 50%). White solid; R_f = 0.43 (silica gel, EtOAc : CH_2Cl_2 : toluene 3 : 1 : 3); ^1H NMR (600 MH_Z, $CDCl_3$): δ 7.89-7.09 (m, 196H, Ar-H), 6.24 (dd, *J* = 10.2, 9.6 Hz, 1H, CH), 6.13-6.04 (m, 13H, 13xCH), 5.60 (d, *J* = 8.4 Hz, 1H, C1-$H_β$), 5.47-5.30 (m, 15H, 2CH, 13xC1-$H_β$), 5.24-5.03 (m, 12H, 12xCH), 4.46-4.39 (m, 3H), 4.33-4.10 (m, 14H), 3.97-3.89 (m, 3H), 3.85-3.50 (m, 37H), 3.33 (ddd, *J* = 10.2, 6.6, 6.6 Hz, 1H), 2.91-2.82 (m, 2H, $CH_{2\text{-linker}}$), 1.92 (s, 3H, OAc), 1.34-1.25 (m, 4H, $2xCH_{2\text{-linker}}$), 1.08-1.05 (m, 2H, $CH_{2\text{linker}}$); ^{13}C NMR (150 MHz, $CDCl_3$): δ = 170.6, 168.0, 167.2, 165.6, 165.5, 165.4, 165.4, 165.2, 165.1, 165.1, 165.0, 164.9, 164.9, 164.8, 134.1, 133.2, 132.9, 131.8, 131.3, 129.8, 129.7, 129.7, 129.2, 129.0, 129.0, 128.9, 128.8, 128.8, 128.7, 128.6, 128.5, 128.3, 128.3, 128.2, 128.1, 123.5, 123.5, 123.4, 97.9, 97.8, 97.6, 97.5, 97.4, 97.1, 73.5, 73.1, 72.9, 72.9, 72.8, 72.7, 72.7, 72.4, 71.7, 71.1, 71.0, 70.5, 70.2, 70.1, 69.9, 69.8, 69.1, 67.6, 67.2, 67.0, 66.8, 62.5, 54.6, 54.5, 51.0, 28.6, 28.3, 23.0, 20.6; MS (MADIL-TOF) m/e: Calcd for $C_{399}H_{307}N_{17}O_{114}$: 7158.9 Found 7149.8.

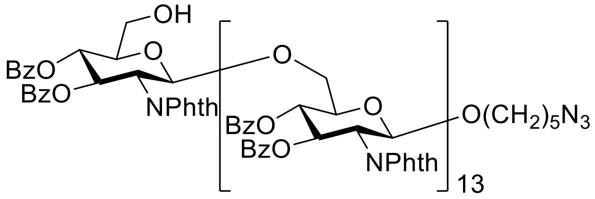

Compound 24. This compound was synthesized according to **General Method B** from acetyl compound **23** (900 mg, 0.126 mmol, 1 eq.) to which was added AcCl (87.9 μmL) in CH_2Cl_2 (15 mL) and MeOH (15 mL) for 2 days. The residue was purified by normal-phase silica gel column chromatography with the eluent EA/CH_2Cl_2/toluene = 1:2.5:2.5 to give compound **24** as a white powder (804 mg, 90%). White solid; R_f = 0.27 (silica gel, EtOAc:CH_2Cl_2:toluene 3:1:3); ^1H NMR

(600 MHz, $CDCl_3$): δ 7.90-7.08 (m, 196H, Ar-H), 6.20 (dd, *J* = 10.2, 9.6 Hz, 1H, CH), 6.14-6.04 (m, 13H, 13xCH), 5.54 (d, *J* = 8.4 Hz, 1H, C1-$H_β$), 5.45-5.32 (m, 14H, CH, 13xC1-$H_β$), 5.25-5.07 (m, 13H, 13xCH), 4.46-4.41 (m, 2H), 4.34-4.17 (m, 12H), 3.97-3.51 (m, 43H), 3.34 (ddd, *J* = 10.2, 6.6, 6.6 Hz, 1H), 3.08 (t, *J* = 7.2 Hz, 1H, OH), 2.91-2.82 (m, 2H, $CH_{2\text{-linker}}$), 1.34-1.25 (m, 4H, 2x$CH_{2\text{-linker}}$), 1.09-1.05 (m, 2H, $CH_{2\text{-linker}}$); ^{13}C NMR (150 MHz, $CDCl_3$): δ = 168.0, 167.1, 165.6, 165.5, 165.5, 165.5, 165.4, 165.4, 165.1, 165.0, 164.9, 164.9, 164.8, 164.8, 134.0, 133.3, 133.1, 132.9, 131.7, 131.2, 129.8, 129.8, 129.6, 129.1, 129.0, 129.0, 128.9, 128.8, 128.7, 128.5, 128.5, 128.3, 128.1, 128.0, 123.5, 123.4, 97.9, 97.8, 97.4, 97.3, 97.2, 74.1, 73.4, 73.1, 72.9, 72.9, 72.8, 72.6, 72.5, 72.3, 72.1, 71.1, 70.9, 70.9, 70.6, 70.3, 70.1, 69.9, 69.6, 69.1, 67.8, 67.6, 67.1, 66.9, 61.1, 54.6, 54.5, 54.4, 51.0, 28.6, 28.2, 23.0; MS (MALDI-TOF) m/e : Calcd for $C_{397}H_{305}N_{17}O_{113}$: 7116.9 Found 7106.5.

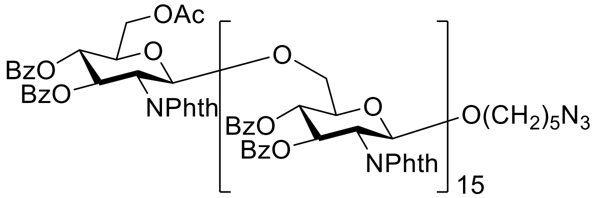

Compound 25. This compound was synthesized according to **General Method A** from donor **10** (798 mg, 0.685 mmol, 6.1 eq.), acceptor **24** (800 mg, 0.112 mmol, 1 eq.), *N*-iodosuccinimide (308 mg, 1.37 mmol, 12.2 eq.) and 0.5 M trifluoromethanesulfonic acid in ether (0.135 mL, 67.4 μmol 0.6 eq.) in CH_2Cl_2 (30 mL). After stirring at -30 °C for 1 h, 0.5 M trifluoromethanesulfonic acid in ether (45 μL, 22.5 μmol, 0.2 eq.) was added to the reaction mixture, which was then warmed to -20 °C for another 1 h. The residue was purified on a normal-phase silica gel chromatography column with the eluent EA/CH_2Cl_2/toluene = 1 : 3 : 3 and on a second chromatography column with the eluent EA/CH_2Cl_2/hexane = 1 : 1 : 1 to give compound **25** as a white powder (476 mg, 52%). White solid; R_f = 0.42 (silica gel, EtOAc : CH_2Cl_2 : toluene 3 : 1 : 3); ^1H NMR (600 MHz, $CDCl_3$): δ 7.89-7.08 (m, 224H, Ar-H), 6.25 (dd, *J* = 10.2, 9.6 Hz, 1H, CH), 6.13-6.05 (m, 15H, 15xCH), 5.61 (d, *J* = 8.4 Hz, 1H, C1-$H_β$), 5.48-5.32 (m, 17H, 2xCH, 15xC1-$H_β$), 5.25-5.03 (m, 14H, 14xCH), 4.49-4.39 (m, 3H), 4.34-4.11 (m, 16H), 3.97-3.52 (m, 46H), 3.34 (ddd, *J* = 10.2, 6.6, 6.6 Hz, 1H), 2.91-2.82 (m, 2H, $CH_{2\text{-linker}}$), 1.93 (s, 3H, OAc), 1.36-1.27 (m, 4H, 2x$CH_{2\text{-linker}}$), 1.09-1.05 (m, 2H, $CH_{2\text{-linker}}$); ^{13}C NMR (150 MHz, $CDCl_3$): δ = 170.6, 168.0, 167.2, 165.6, 165.5, 165.4, 165.2, 165.1, 165.1, 165.0, 164.9, 164.9, 164.8, 134.1, 133.2, 132.9, 131.8, 131.3, 129.8, 129.7, 129.7, 129.2, 129.0, 129.0, 128.9, 128.8, 128.8, 128.7, 128.6, 128.5, 128.3, 128.2, 128.1, 123.5, 123.4, 123.4, 97.9, 97.8, 97.5, 97.4, 97.1, 73.5, 73.1, 72.9, 72.7, 72.4, 71.7, 71.1, 70.9, 70.5, 70.2, 70.1, 69.9, 69.8, 69.1, 67.6, 67.2, 66.9, 66.9, 62.5, 54.8, 54.6, 54.5, 51.0, 28.6, 28.3, 23.0, 20.6; MS (MADIL-TOF) m/e: Calcd for $C_{455}H_{349}N_{19}O_{130}$: 8157.1 Found 8145.6.

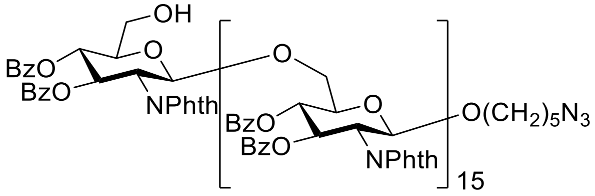

Compound 26. This compound was synthesized according to **General Method B** from acetyl compound **25** (470 mg, 57.6 μmol, 1 eq.) to which was added AcCl (46.1 μmL) in CH_2Cl_2 (7 mL) and MeOH (7 mL) for 2 days. The residue was purified by normal-phase silica gel column chromatography with the eluent EA/CH_2Cl_2/toluene = 1:2.5:2.5 to give compound **26** as a white powder (412 mg, 88%). White solid; R_f = 0.28 (silica gel, EtOAc:CH_2Cl_2:toluene 3:1:3); ^1H NMR

(600 MHz, $CDCl_3$): δ 7.90-7.09 (m, 224H, Ar-H), 6.19 (dd, *J* = 10.2, 9.6 Hz, 1H, CH), 6.12-6.03 (m, 15H, 15xCH), 5.53 (d, *J* = 7.8 Hz, 1H, C1-$H_β$), 5.44-5.31 (m, 16H, CH, 15xC1-$H_β$), 5.23-5.06 (m, 15H, 15xCH), 4.45-4.40 (m, 2H), 4.33-4.16 (m, 14H), 3.96-3.50 (m, 49H), 3.33 (ddd, *J* = 10.2, 6.6, 6.6 Hz, 1H), 3.06 (br, 1H, OH), 2.91-2.82 (m, 2H, $CH_{2\text{-linker}}$), 1.35-1.27 (m, 4H, 2x$CH_{2\text{-linker}}$), 1.09-1.06 (m, 2H, $CH_{2\text{-linker}}$); ^{13}C NMR (150 MHz, $CDCl_3$): δ = 168.0, 167.2, 165.6, 165.6, 165.5, 165.4, 165.2, 165.0, 164.9, 164.8, 134.1, 133.3, 133.2, 132.0, 131.8, 131.3, 129.9, 129.8, 129.7, 129.2, 129.1, 129.0, 128.8, 128.7, 128.6, 128.6, 128.3, 128.2, 128.1, 123.5, 97.9, 97.8, 97.5, 97.5, 97.2, 74.1, 73.5, 73.1, 72.7, 72.4, 72.2, 71.2, 71.0, 70.9, 70.7, 70.3, 70.1, 69.9, 69.7, 69.1, 67.9, 67.6, 67.2, 67.0, 54.7, 54.5, 54.5, 51.1, 28.7, 28.3, 23.0; MS (MALDI-TOF) m/e : Calcd for $C_{453}H_{347}N_{19}O_{129}$: 8115.1 Found 8100.3.

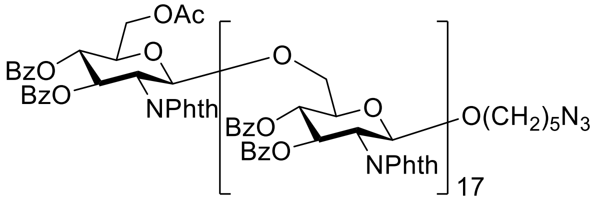

Compound 27. This compound was synthesized according to **General Method A** from donor **10** (197 mg, 0.169 mmol, 7 eq.), acceptor **26** (196 mg, 24.1 µmol, 1 eq.), *N*-iodosuccinimide (76.0 mg, 0.338 mmol, 14 eq.) and 0.5 M trifluoromethanesulfonic acid in ether (57.9 µL, 29.0 µmol 1.2 eq.) in CH_2Cl_2 (7 mL). The residue was purified on a normal-phase silica gel chromatography column with the eluent EA/CH_2Cl_2/toluene = 1 : 3 : 3 and on a second chromatography column with the eluent EA/CH_2Cl_2/hexane = 1 : 1 : 1 to give compound **27** as a white powder (130 mg, 59%). White solid; R_f = 0.35 (silica gel, EtOAc : CH_2Cl_2 : toluene 3:1:3); ^1H NMR (600 MHz, $CDCl_3$): δ 7.89-7.04 (m, 252H, Ar-H), 6.24 (dd, *J* = 9.6, 9.0 Hz, 1H, CH), 6.12-6.04 (m, 17H, 17xCH), 5.59 (d, *J* = 8.4 Hz, 1H, C1-$H_β$), 5.46-5.31 (m, 19H, 2xCH, 17xC1-$H_β$), 5.23-5.02 (m, 16H, 16xCH), 4.45-4.38 (m, 3H), 4.33-4.10 (m, 18H), 3.96-3.51 (m, 52H), 3.33 (ddd, *J* = 9.6, 6.6, 6.6 Hz, 1H), 2.91-2.82 (m, 2H, $CH_{2\text{-linker}}$), 1.92 (s, 3H, OAc), 1.35-1.27 (m, 4H, 2x$CH_{2\text{-linker}}$), 1.09-1.03 (m, 2H, $CH_{2\text{-linker}}$); ^{13}C NMR (150 MHz, $CDCl_3$): δ = 170.6, 168.1, 167.2, 165.6, 165.6, 165.4, 165.2, 165.1, 165.1, 165.0, 165.0, 164.9, 164.8, 134.1, 133.2, 133.0, 131.8, 131.3, 129.9, 129.8, 129.7, 129.3, 129.1, 129.0, 128.9, 128.9, 128.8, 128.7, 128.7, 128.6, 128.4, 128.2, 128.1, 123.5, 123.5, 123.4, 97.9, 97.8, 97.5, 97.4, 97.2, 73.5, 73.1, 72.9, 72.9, 72.7, 72.4, 71.7, 71.2, 71.0, 70.5, 70.3, 70.1, 69.9, 69.9, 69.1, 67.2, 66.9, 66.6, 54.8, 54.7, 54.6, 51.1, 28.7, 28.3, 23.0, 20.6; MS (MADIL-TOF) m/e: Calcd for $C_{511}H_{391}N_{21}O_{146}$: 9155.3817 Found 9139.6.

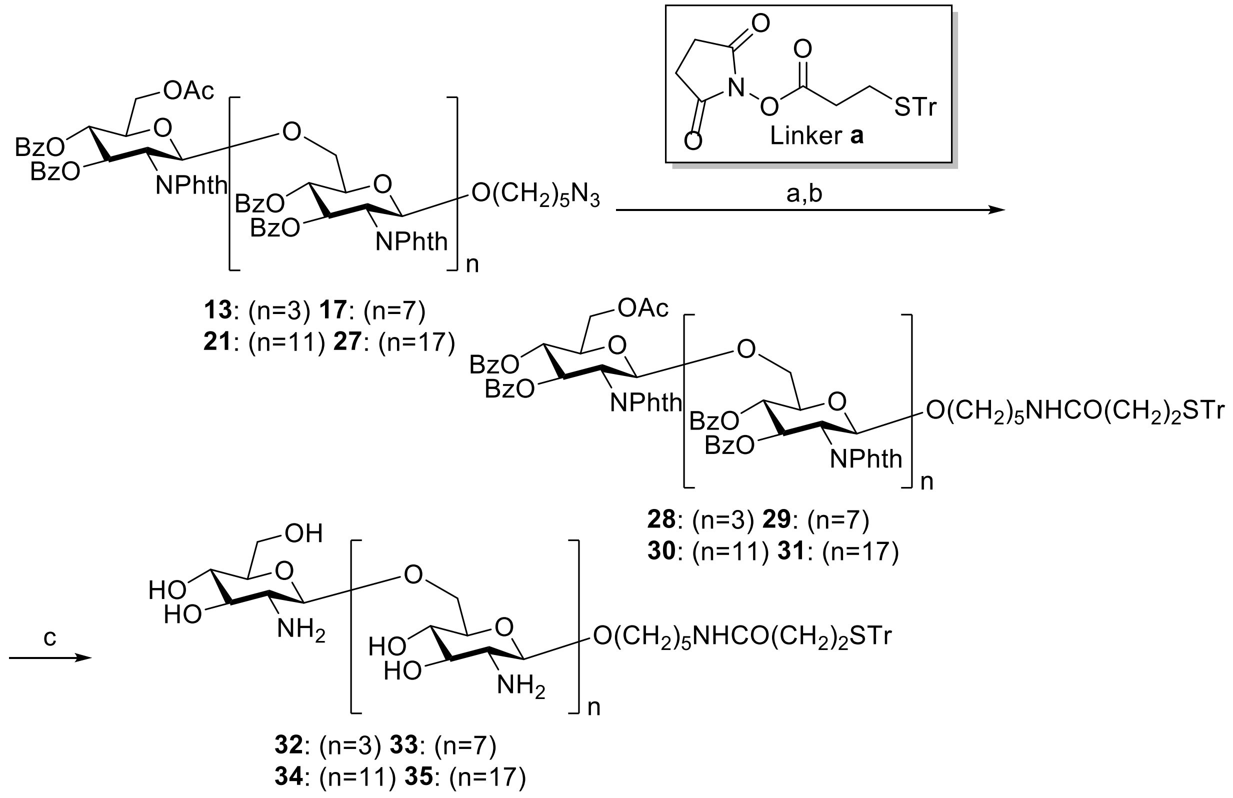

Figure S4. Installation of the Thiol Linker and Global Deprotection

Reagents and conditions: (a) H_2, $Pd(OH)_2/C$, cat. AcOH, $MeOH/CH_2Cl_2$, rt, 16 h; (b) Linker **a**, Et_3N, rt, o/n **28**: (n = 3) 62%, **29**: (n = 7) 75% **30**: (n = 11) 53%, **31**: (n = 17) 51% (two steps); (c) $N_2H_4{\cdot}H_2O$, EtOH, reflux, 3h **32**: (n = 3) 93%, **33**: (n = 7) 91% **34**: (n = 11) 96%, **35**: (n = 17) 94%.

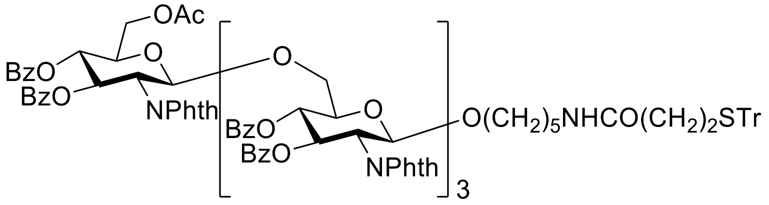

Compound 28. Compound **13** (1.20 g, 0.553 mol, 1.0 eq.) was dissolved in CH_2Cl_2 (20 mL) and MeOH (20 mL) at room temperature. Palladium hydroxide on carbon (20 wt. %) (120 mg) and cat. acetic acid (2 drops) were added and stirred overnight at room temperature under hydrogen gas at normal pressure. After the reaction was complete, the solution was filtered through celite. The filtrate was concentrated and vacuumed to give a residual amine compound (1.19 g). This amine compound was dissolved in CH_2Cl_2 (45 mL) at 0 ºC. 2,5-Dioxopyrrolidin-1-yl 3-(tritylthio)propanoate (739 mg, 1.66 mol, 3 eq.) and Et_3N (0.46 mL, 3.32 mmol, 6 eq.) were added and continuously stirred overnight at room temperature. After the reaction was complete, the solution was quenched with saturated $NaHCO_3$ $_{(aq.)}$ and brine. The organic layer was dried over $MgSO_4$ and concentrated. The residue was purified by normal-phase silica gel column chromatography with the eluent EA/CH_2Cl_2/toluene = 1 : 1.5 : 1.5, to give compound **28** as a white

powder (842 mg, 62%). White solid; R_f = 0.39 (silica gel, EtOAc : CH_2Cl_2 : toluene 2 : 1 : 2); ^1H NMR (600 MHz, $CDCl_3$): δ 7.86-7.63 (m, 32H, Ar-H), 7.53-7.31 (m, 22H, Ar-H), 7.27-7.12 (m, 17H, Ar-H), 6.28 (dd, *J* = 10.2, 9.6 Hz, 1H, CH), 6.16 (dd, *J* = 10.8, 9.6 Hz, 1H, CH), 6.07-6.02 (m, 2H, 2xCH), 5.67 (d, *J* = 8.4 Hz, 1H, C1-$H_β$), 5.52-5.49 (m, 2H, CH, C1-$H_β$), 5.44 (dd, *J* = 5.4, 5.4 Hz, 1H, NHCO), 5.38-5.30 (m, 3H, CH, 2xC1-$H_β$), 5.23 (dd, *J* = 10.2, 9.6 Hz, 1H, CH), 4.98 (dd, *J* = 9.6, 9.6 Hz, 1H, CH), 4.47-4.39 (m, 3H), 4.28-4.21 (m, 2H), 4.18-4.16 (m, 2H), 3.95-3.89 (m, 4H), 3.85 (dd, *J* = 10.8, 8.4 Hz, 1H), 3.82-3.77 (m, 2H), 3.71 (dd, *J* = 9.0, 4.2, 4.2 Hz, 1H), 3.66 (ddd, *J* = 10.2, 6.0, 6.0 Hz, 1H), 3.49 (dd, *J* = 10.8, 4.2 Hz, 1H), 3.34 (ddd, *J* = 10.2, 6.6, 6.6 Hz, 1H), 2.84 (dt, *J* = 6.6, 6.6 Hz, 2H, $CH_{2\text{-linker}}$), 2.42 (t, *J* = 7.2 Hz, 2H, $CH_{2\text{-linker}}$), 1.97 (t, *J* = 7.2 Hz, 2H, $CH_{2\text{-linker}}$), 1.93 (s, 3H, OAc), 1.37-1.20 (m, 4H, 2x$CH_{2\text{-linker}}$), 1.07-1.02 (m, 2H, $CH_{2\text{-linker}}$); ^{13}C NMR (150 MHz, $CDCl_3$): δ = 170.6, 170.5, 168.1, 168.1, 167.3, 165.6, 165.6, 165.5, 165.4, 165.2, 165.1, 164.7, 144.7, 134.2, 134.0, 133.3, 133.3, 133.1, 133.1, 131.6, 131.3, 129.9, 129.8, 129.7, 129.7, 129.6, 129.5, 129.2, 128.8, 128.8, 128.6, 128.4, 128.4, 128.3, 128.2, 128.2, 128.1, 127.9, 126.6, 123.7, 123.5, 123.5, 98.0, 97.9, 97.9, 97.0. 73.6, 73.1, 72.6, 71.7, 71.1, 70.8, 70.4, 70.2, 70.1, 69.8, 69.4, 67.8, 67.7, 67.0, 66.7, 62.5, 54.9, 54.8, 54.7, 54.5, 39.3, 35.4, 28.9, 28.7, 27.7, 23.2, 20.6; HRMS (MADIL-TOF) m/e: Calcd for $C_{141}H_{117}N_5O_{35}SNa$ $[M+Na]^+$: 2494.7142 Found 2494.7323.

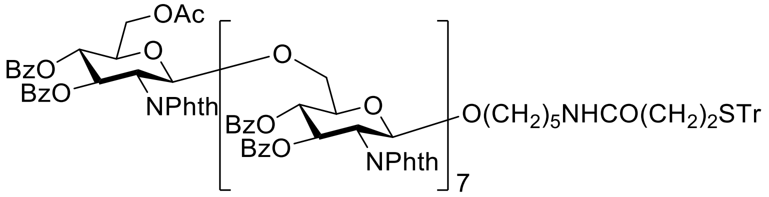

Compound 29. Compound **17** (810 mg, 0.194 mol, 1.0 eq.) was dissolved in CH_2Cl_2 (15 mL) and MeOH (15 mL) at room temperature. Palladium hydroxide on carbon (20 wt. %) (81 mg) and cat. acetic acid (2 drops) were added and stirred continuously at room temperature overnight under hydrogen gas at normal pressure. After the reaction was complete, the solution was filtered through celite. The filtrate was concentrated and vacuumed to give an amine compound (799 mg) as a residue. This amine compound was dissolved in CH_2Cl_2 (30 mL) at 0 ºC, and 2,5-dioxopyrrolidin-1-yl 3-(tritylthio)propanoate (258 mg, 579 mmol, 3 eq.), and Et_3N (0.161 mL, 1.16 mol, 6 eq.) were added and stirred continuously overnight at room temperature. After ther reaction was complete, the solution was quenched with saturated $NaHCO_{3\,(aq.)}$ and brine. The organic layer was dried over $MgSO_4$ and concentrated. The residue was purified by normal-phase silica gel column chromatography with the eluent EA/CH_2Cl_2/toluene = 1 : 1.5 : 1.5 to give compound **36** as a white powder (652 mg, 75%). White solid; R_f = 0.33 (silica gel, EtOAc : CH_2Cl_2 : toluene 2 : 1 : 2); ^1H NMR (600 MHz, $CDCl_3$): δ 7.91-7.60 (m, 64H, Ar-H), 7.47-7.08 (m, 63H, Ar-H), 6.26 (dd, *J* = 10.2, 9.6 Hz, 1H, CH), 6.16-6.06 (m, 7H, 7xCH), 5.62 (d, *J* = 7.8 Hz, 1H, C1-$H_β$), 5.49-5.33 (m, 10H, NHCO, 2xCH, 7xC1-$H_β$), 5.24 (dd, *J* = 9.6, 9.0 Hz, 1H, CH), 5.20-5.06 (m, 5H, 5xCH), 4.47-4.40 (m, 3H), 4.35-4.22 (m, 6H), 4.17-4.11 (m, 2H), 3.95-3.93 (m, 3H), 3.87-3.55 (m, 19H), 3.32 (ddd, *J* = 9.6, 6.0, 6.0 Hz, 1H), 2.89-2.81 (m, 2H, $CH_{2\text{-linker}}$), 2.44 (t, *J* = 7.8 Hz, 2H, $CH_{2\text{-linker}}$), 1.99 (t, *J* = 7.8 Hz, 2H, $CH_{2\text{-linker}}$), 1.93 (s, 3H, OAc), 1.35-1.19 (m, 4H, 2x$CH_{2\text{linker}}$), 1.05-1.00 (m, 2H, $CH_{2\text{-linker}}$); ^{13}C NMR (150 MHz, $CDCl_3$): δ = 170.6, 170.5, 168.0, 167.2, 165.5, 165.5, 165.4, 165.4, 165.3, 164.9, 164.9, 164.9, 164.7, 144.6, 134.2, 134.1, 133.9, 133.2, 133.1, 133.0, 133.0, 132.9, 131.2, 129.8, 129.7, 129.7, 129.6, 129.5, 129.2, 128.9, 128.9, 128.9, 128.8, 128.8, 128.8, 128.7, 128.7, 128.6, 128.6, 128.6, 128.5, 128.3, 128.2, 128.2, 128.0, 127.8, 126.6, 123.4, 123.4, 97.9, 97.8, 97.6, 97.5, 97.3, 97.1, 73.6, 73.0, 73.0, 72.9, 72.9, 72.8, 72.8, 71.6, 71.0, 71.0, 70.9,

70.8, 70.4, 70.2, 70.1, 70.0, 69.9, 69.8, 69.3, 67.6, 67.2, 67.0, 66.6, 62.5, 54.8, 54.7, 54.7, 54.6, 54.5, 39.2, 35.3, 28.8, 28.7, 27.6, 23.1, 20.5; HRMS (ESI-TOF) m/e: Calcd for $C_{253}H_{200}N_9O_{67}SNa_2$ $[M+2Na]^{2+}$: 2257.1051 Found 2257.1000.

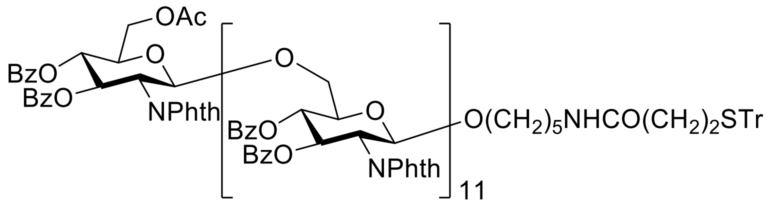

Compound 30. Compound **21** (890 mg, 0.144 mol, 1.0 eq.) was dissolved in CH_2Cl_2 (15 mL) and MeOH (15 mL) at room temperature. Palladium hydroxide on carbon (20 wt. %) (89 mg) and cat. acetic acid (2 drops) were added and stirred continuously at room temperature overnight under normal pressure hydrogen gas. After the reaction was complete, the solution was filtered through celite. The filtrate was concentrated and vacuumed to give a residual amine compound (875 mg). This amine compound was dissolved in CH_2Cl_2 (25 mL) at 0 ºC, and 2,5-dioxopyrrolidin-1-yl 3-(tritylthio)propanoate (193 mg, 433 mmol, 3 eq.), and Et_3N (0.121 mL, 866 mmol, 6 eq.) were added and stirred continuously overnight at room temperature. After the reaction was complete, the solution was quenched with saturated $NaHCO_{3\,(aq.)}$ and brine. The organic layer was dried over $MgSO_4$ and concentrated. The residue was purified by normal-phase silica gel column chromatography with the eluent EA/CH_2Cl_2/toluene = 1:1.5:1.5 to give compound **30** as a white powder (492 mg, 53%). White solid; R_f = 0.35 (silica gel, EtOAc:CH_2Cl_2:toluene 2:1:2); ^1H NMR (600 MHz, $CDCl_3$): δ 7.89-7.09 (m, 183H, Ar-H), 6.25 (dd, J = 10.2, 9.6 Hz, 1H, CH), 6.14-6.05 (m, 11H, 11xCH), 5.60 (d, J = 8.4 Hz, 1H, C1-$H_β$), 5.48-5.32 (m, 14H, NHCO, 2xCH, 11xC1-$H_β$), 5.25-5.03 (m, 10H, 10xCH), 4.46-4.39 (m, 3H), 4.33-4.11 (m, 12H), 3.95-3.92 (m, 3H), 3.85-3.50 (m, 31H), 3.30 (ddd, J = 10.2, 6.6, 6.0 Hz, 1H), 2.87-2.80 (m, 2H, $CH_{2\text{-linker}}$), 2.44 (t, J = 7.2 Hz, 2H, $CH_{2\text{-linker}}$), 1.98 (t, J = 7.2 Hz, 2H, $CH_{2\text{-linker}}$), 1.92 (s, 3H, OAc), 1.33-1.18 (m, 4H, 2x$CH_{2\text{-linker}}$), 1.03-1.00 (m, 2H, $CH_{2\text{-linker}}$); ^{13}C NMR (150 MHz, $CDCl_3$): δ = 170.6, 170.6, 168.0, 167.2, 165.6, 165.6, 165.2, 165.1, 165.1, 165.0, 164.9, 164.9, 164.7, 144.6, 134.2, 134.1, 134.0, 133.3, 133.2, 133.0, 132.9, 131.8, 131.3, 129.9, 129.7, 129.7, 129.5, 129.2, 129.0, 128.9, 128.9, 128.8, 128.8, 128.8, 128.7, 128.7, 128.6, 128.6, 128.6, 128.5, 128.3, 128.3, 128.2, 128.1, 127.8, 126.6, 123.5, 123.4, 97.9, 97.5, 97.5, 97.4, 97.1, 73.6, 73.0, 72.9, 72.9, 72.8, 72.7, 72.6, 72.4, 71.6, 71.0, 70.9, 70.9, 70.8, 70.4, 70.2, 70.1, 69.9, 69.8, 69.3, 67.6, 67.2, 67.1, 67.0, 66.8, 66.7, 66.6, 62.5, 54.8, 54.6, 54.5, 39.2, 35.4, 28.8, 28.7, 27.6, 23.1, 20.6; HRMS (ESI-TOF) m/e: Calcd for $C_{365}H_{287}N_{13}O_{99}S$ $[M+2H]^{2+}$: 3233.3766 Found 3233.3772.

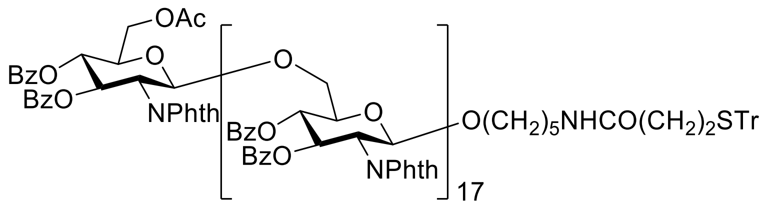

Compound 31. Compound **27** (115 mg, 12.6 μmol, 1.0 eq.) was dissolved in CH_2Cl_2 (6 mL) and MeOH (3 mL) at room temperature. Palladium hydroxide on carbon (20 wt. %) (40 mg) and cat. acetic acid (2 drops) were added and stirred continuously overnight at room termperature under normal pressure hydrogen gas. After the reaction was complete, the solution was filtered through celite. The filtrate was concentrated and vacuumed to give a residual amine compound (114 mg).

This amine compound was dissolved in CH_2Cl_2 (10 mL) at 0° and 2,5-dioxopyrrolidin-1-yl 3-(tritylthio)propanoate (16.6 mg, 37.4 μmol, 3 eq.) and Et_3N (10.4 μL, 74.9 μmol, 6 eq.) were added and stirred continuously overnight at room temperature. After the reaction was complete, the solution was quenched with saturated $NaHCO_3$ (aq.) and brine. The organic layer was dried over $MgSO_4$ and concentrated. The residue was purified by normal-phase silica gel column chromatography with the eluent EA/CH_2Cl_2/toluene = 1 : 1.5 : 1.5, to give compound **30** as a white powder (60.6 mg, 51%). White solid; R_f = 0.41 (silica gel, EtOAc : CH_2Cl_2 : toluene 2:1:2); ^{1}H NMR (600 MHz, $CDCl_3$): δ 7.88-7.09 (m, 252H, Ar-H), 6.23 (dd, J = 10.2, 9.6 Hz, 1H, CH), 6.12-6.03 (m, 17H, 17xCH), 5.59 (d, J = 8.4 Hz, 1H, C1-H_β), 5.46-5.30 (m, 20H, NHCO, 2xCH, 17xC1-H_β), 5.23-5.02 (m, 16H, 16xCH), 4.44-4.38 (m, 3H), 4.32-4.08 (m, 18H), 3.93-3.91 (m, 3H), 3.83-3.50 (m, 49H), 3.29 (ddd, J = 9.6, 6.6, 6.6 Hz, 1H), 2.88-2.78 (m, 2H, $CH_{2\text{-linker}}$), 2.43 (t, J = 7.2 Hz, 2H, $CH_{2\text{-linker}}$), 1.97 (t, J = 7.2 Hz, 2H, $CH_{2\text{-linker}}$), 1.92 (s, 3H, OAc), 1.32-1.19 (m, 4H, 2x$CH_{2\text{-linker}}$), 1.03-0.99 (m, 2H, $CH_{2\text{-linker}}$); ^{13}C NMR (150 MHz, $CDCl_3$): δ = 170.7, 170.6, 168.1, 167.2, 165.6, 165.5, 165.4, 165.2, 165.2, 165.1, 165.0, 164.8, 144.7, 134.1, 133.2, 133.0, 131.8, 131.3, 129.9, 129.8, 129.7, 129.6, 129.3, 129.1, 129.0, 128.9, 128.9, 128.8, 128.8, 128.7, 128.7, 128.6, 128.6, 128.4, 128.2, 128.1, 127.9, 126.7, 123.5, 123.4, 97.9, 97.8, 97.5, 97.2, 73.6, 73.1, 73.0, 72.7, 72.5, 71.7, 71.1, 71.0, 70.5, 70.1, 70.0, 69.9, 69.3, 67.2, 66.9, 66.8, 66.7, 62.6, 54.8, 54.7, 54.6, 39.2, 35.4, 29.7, 28.9, 28.7, 27.7, 23.2, 20.6; MS (MADIL-TOF) m/e: Calcd for $C_{533}H_{411}N_{19}O_{147}S$: 9459.5 Found 9454.2.

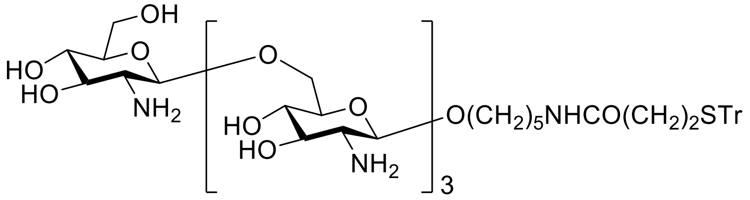

Compound 32. Compound **28** (202 mg, 81.7 μmol) was dissolved in EtOH (7 mL) and hydrazine monohydrate (0.7 mL) was added at room temperature. The mixture was heated to reflux for 3 h. After the reaction was complete, the mixture was concentrated on a rotary evaporator. The residue was fractionated by Biogel P-2 chromatography with H_2O as the eluent to give Compound **32** as a white powder (82.0 mg, 93%). White solid; ^{1}H NMR (600 MHz, DMSO-d6): δ 7.79 (t, J = 5.4 Hz, 1H, NHCO), 7.35-7.23 (m, 15H, Ar-H), 5.11-5.07 (m, 5H, NH_2), 4.99 (d, J = 3.6 Hz, 1H, NH_2), 4.91 (br, 1H, NH_2), 4.52 (t, J = 5.4 Hz, 1H, NH_2), 4.14-4.12 (m, 3H, 3xC1-H_β), 4.05-4.00 (m, 4H, C1-H_β, 3/2xCH_2), 3.72 (ddd, J = 9.6, 6.6, 6.6 Hz, 1H, 1/2x$CH_{2\text{-linker}}$), 3.67 (dd, J = 11.4, 4.8 Hz, 1H, 1/2xCH_2), 3.53-3.49 (m, 3H, 3/2xCH_2), 3.47-3.45 (m, 1H, 1/2x$CH_{2\text{-linker}}$), 3.38-3.36 (m, 1H, 1/2xCH_2), 3.27-3.25 (m, 3H, 3xCH), 3.09-2.97 (m, 11H, 9xCH, $CH_{2\text{-linker}}$), 2.42-2.37 (m, 4H, 4xCH), 2.22 (t, J = 7.2 Hz, 2H, $CH_{2\text{-linker}}$), 2.13 (t, J = 7.2 Hz, 2H, $CH_{2\text{-linker}}$), 1.51-1.46 (m, 2H, $CH_{2\text{-linker}}$), 1.38-1.34 (m, 2H, $CH_{2\text{-linker}}$), 1.30-1.26 (m, 2H, $CH_{2\text{-linker}}$); ^{13}C NMR (150 MHz, DMSO-d6): δ = 169.8, 144.4, 129.1, 128.0, 126.7, 104.2, 104.1, 104.0, 103.6, 77.1, 76.6, 76.5, 76.3, 75.7, 75.7, 75.6, 70.0, 68.8, 68.5, 65.9, 61.0, 57.5, 57.4, 57.3, 38.4, 34.0, 28.9, 28.8, 27.6, 23.0; HRMS (ESI-TOF) m/e: Calcd for $C_{51}H_{76}N_5O_{18}S$ $[M+H]^+$: 1078.4901 Found 1078.4910.

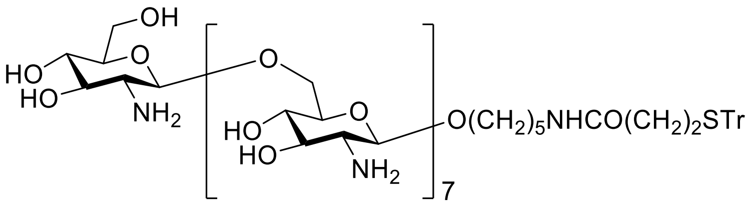

Compound 33. Compound **29** (195 mg, 43.6 μmol) was dissolved in EtOH (8 mL) and hydrazine monohydrate (0.8 mL) was added at room temperature. The mixture was heated to reflux for 3 h. After the reaction was complete, the residue was concentrated on a rotary evaporator, before fractionation on a Toyopearl HW-40 chromatography column, with the eluent 0.1 M AcOH to give Compound **33** as a white powder (68.4 mg, 91%). White solid; ^{1}H NMR (600 MHz, DMSO-d6): δ 7.79 (t, *J* = 5.4 Hz, 1H, NHCO), 7.35-7.24 (m, 15H, Ar-H), 5.26-4.93 (m, 14H, NH_2), 4.89 (br, 1H, NH_2), 4.53 (br, 1H, NH_2), 4.13-4.12 (m, 7H, 7xC1-H_β), 4.06-4.01 (m, 8H, C1-H_β, 7/2xCH_2), 3.74-3.71 (m, 1H, 1/2x$CH_{2\text{-linker}}$), 3.68-3.66 (m, 1H, 1/2xCH_2), 3.52-3.44 (m, 9H, 4xCH_2, 1/2x$CH_{2\text{-linker}}$), 3.28-3.27 (m, 7H, 7xCH), 3.10-2.97 (m, 19H, 17xCH, $CH_{2\text{-linker}}$), 2.43-2.40 (m, 8H, 8xCH), 2.22 (t, *J* = 7.8 Hz, 2H, $CH_{2\text{-linker}}$), 2.13 (t, *J* = 7.8 Hz, 2H, $CH_{2\text{-linker}}$), 1.50-1.47 (m, 2H, $CH_{2\text{-linker}}$), 1.39-1.34 (m, 2H, $CH_{2\text{-linker}}$), 1.30-1.26 (m, 2H, $CH_{2\text{-linker}}$); ^{13}C NMR (150 MHz, DMSO-d6): δ = 169.8, 144.4, 129.1, 128.0, 126.7, 103.2, 102.5, 77.1, 75.6, 70.0, 69.9, 68.8, 68.6, 65.9, 60.9, 57.1, 57.1, 38.4, 34.0, 29.0, 28.8, 27.6, 22.9; HRMS (ESI-TOF) m/e: Calcd for $C_{75}H_{120}N_9O_{34}S$ $[M+H]^+$: 1722.7653 Found 1722.7723.

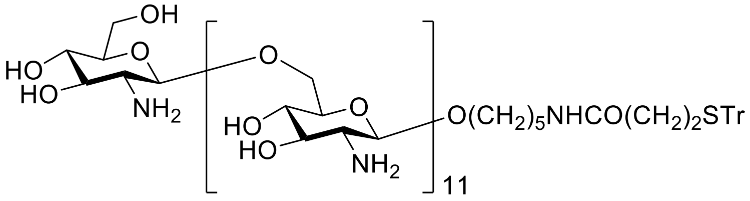

Compound 34. Compound **30** (235 mg, 36.3 μmol) was dissolved in EtOH (7 mL) and hydrazine monohydrate (0.7 mL) was added at room temperature and the mixture was heated to reflux for 3 h. After the reaction was complete, the mixture was concentrated on a rotary evaporator, and the residue was fractionated by Toyopearl HW-40 column chromatography, using the eluent 0.1 M AcOH to give Compound **34** as a white powder (52.6 mg, 96%). White solid; ^{1}H NMR (600 MHz, DMSO-d6): δ 7.79 (t, *J* = 6.0 Hz, 1H, NHCO), 7.35-7.24 (m, 15H, Ar-H), 5.08-4.41 (m, 17H, NH), 4.16-4.15 (m, 11H, 11xC1-H_β), 4.04-4.02 (m, 12H, C1-H_β, 11/2xCH_2), 3.73-3.71 (m, 1H, 1/2x$CH_{2\text{-linker}}$), 3.68-3.67 (m, 1H, 1/2xCH_2), 3.53-3.45 (m, 13H, 6xCH_2, 1/2x$CH_{2\text{-linker}}$), 3.29-3.25 (m, 11H, 11xCH), 3.07-2.97 (m, 27H, 25xCH, $CH_{2\text{-linker}}$), 2.44-2.38 (m, 12H, 12xCH), 2.23 (t, *J* = 7.8 Hz, 2H, $CH_{2\text{-linker}}$), 2.13 (t, *J* = 7.8 Hz, 2H, $CH_{2\text{-linker}}$), 1.50-1.48 (m, 2H, $CH_{2\text{-linker}}$), 1.37-1.34 (m, 2H, $CH_{2\text{-linker}}$), 1.30-1.28 (m, 2H, $CH_{2\text{-linker}}$); ^{13}C NMR (150 MHz, DMSO-d6): δ = 169.8, 144.5, 129.1, 128.0, 126.7, 103.9, 103.8, 103.2, 77.1, 76.3, 76.2, 76.1, 76.0, 75.7, 75.6, 70.0, 69.9, 68.8, 68.5, 65.9, 61.0, 57.3, 57.2, 38.4, 34.0, 28.9, 28.8, 27.6, 23.0; HRMS (ESI-TOF) m/e: Calcd for $C_{99}H_{164}N_{13}O_{50}S$ $[M+H]^+$: 2367.0405 Found 2367.0462.

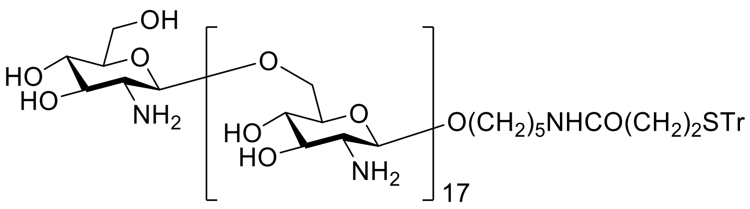

Compound 35. Compound **31** (76 mg, 8.03 μmol) was dissolved in EtOH (4 mL), hydrazine monohydrate (0.4 mL) was added at room temperature, and the mixture was heated to reflux for 3 h. After the reaction was complete, the mixture was concentrated on a rotary evaporator, and the residue was fractionated on a Toyopearl HW-40 chromatography column, using the eluent 0.1 M AcOH to give Compound **35** as a white powder (25.0 mg, 94%). White solid; ^{1}H NMR (600 MHz, DMSO-d6): δ 7.80 (t, *J* = 5.4 Hz, 1H, NHCO), 7.35-7.23 (m, 15H, Ar-H), 4.75 (br, 73H, NH_2, OH), 4.21 (br, 17H, 17xC1-$H_β$), 4.08-4.02 (m, 18H, C1-$H_β$, 17/2xCH_2), 3.72-3.71 (m, 1H, 1/2x$CH_{2\text{-linker}}$), 3.67 (d, *J* = 11.4 Hz, 1H, 1/2xCH_2), 3.55-3.46 (m, 19H, 9xCH_2, 1/2x$CH_{2\text{-linker}}$), 3.29 (br, 17H, 17xCH), 3.11 (br, 37H, 37xCH), 2.99-2.98 (m, 2H, 2x$CH_{2\text{-linker}}$), 2.45 (br, 18H, 18xCH), 2.22 (t, *J* = 7.2 Hz, 2H, $CH_{2\text{-linker}}$), 2.13 (t, *J* = 7.2 Hz, 2H, $CH_{2\text{-linker}}$), 1.49-1.48 (m, 2H, $CH_{2\text{-linker}}$), 1.37-1.35 (m, 2H, $CH_{2\text{-linker}}$), 1.28-1.27 (m, 2H, $CH_{2\text{-linker}}$); ^{13}C NMR (150 MHz, DMSO-d6): δ = 169.8, 144.5, 129.1, 128.1, 126.7, 103.4, 77.2, 75.7, 70.0, 69.9, 68.8, 68.6, 66.0, 60.9, 57.2, 38.4, 34.0, 28.8, 27.6, 23.0; HRMS (ESI-TOF) m/e: Calcd for $C_{135}H_{232}N_{19}O_{74}S$ $[M+3H]^{3+}$: 1111.8226 Found 1111.8236.

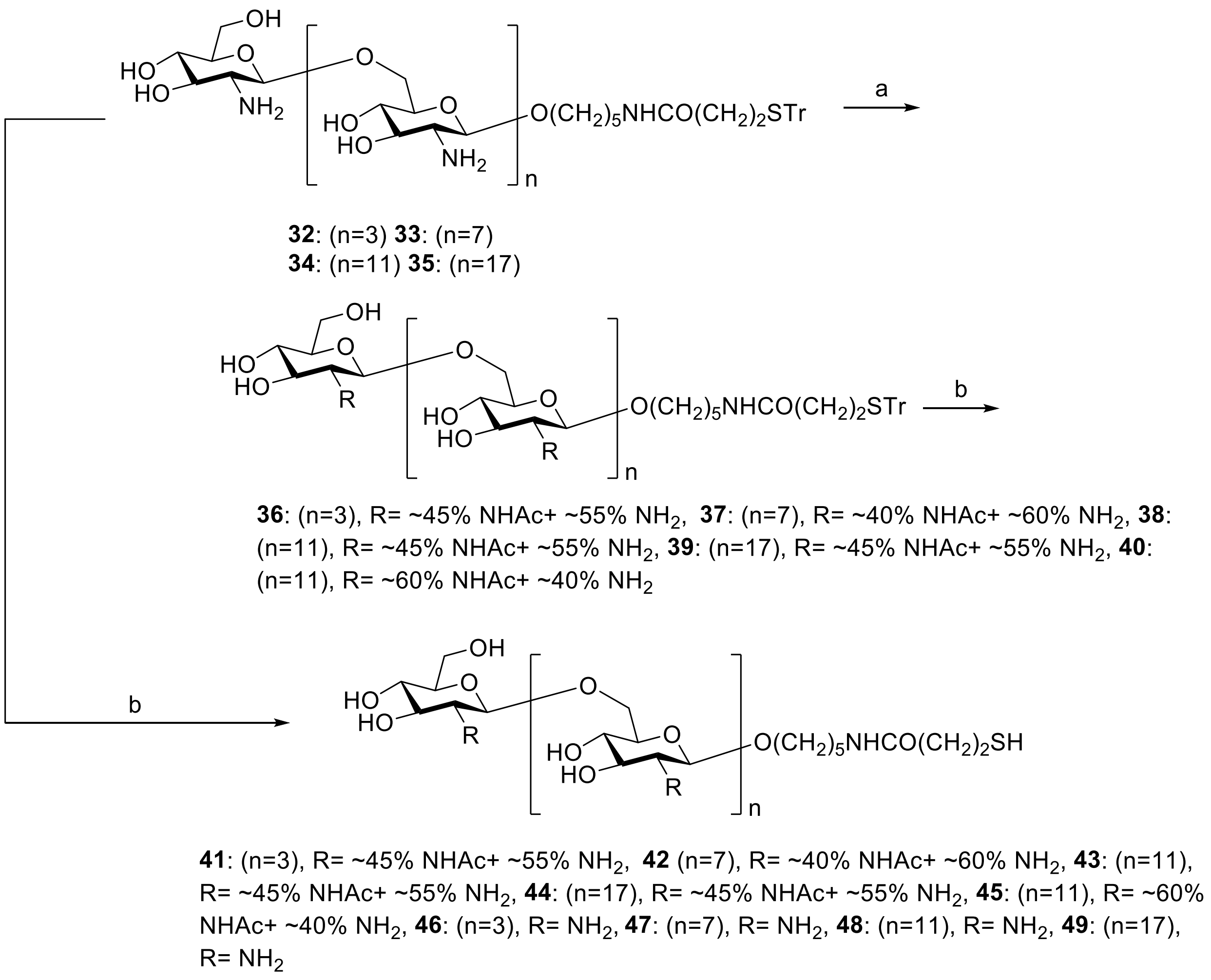

Figure S5. Synthesis of dPNAG Glycan Antigens with *N*-Acetylation

Reagents and conditions: (a) (d) Ac_2O, $NaHCO_3$, H_2O/MeOH, rt, o/n **36**: (n=3), **37**: (n=7) **38**: (n=11), **39**: (n=17), **40**: (n=11); and (e) TFA, Et_3SiH, rt, 1h **41**: (n=3), **42**: (n=7), **43**: (n=11), **44**: (n=17), **45**: (n=11), **46**: (n=3) 95%, **47**: (n=7) 94%, **48**: (n=11) 91%, **49**: (n=17) 86%.

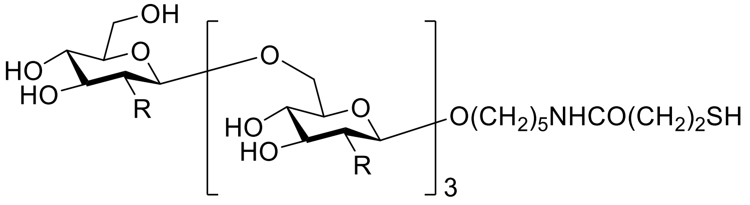

R= ~40% NHAc+ ~60% NH_2

Compound 41. Compound **32** (121 mg, 0.112 mmol) was dissolved in MeOH (5 mL) and water (5 mL) at room temperature. $NaHCO_3$ (28 mg, 0.337 mmol) and acetic anhydride (0.34 mL, 0.168 mmol, 0.5 M in dioxane) were added to the reaction mixture, which was then stirred continuously

overnight at room temperature. After the reaction was complete, the reaction mixutre was concentrated on a rotary evaporator and the residue fractionated by Biogel P-2 column chromatography, with the eluent H_2O to give Compound **36** as a white powder (114 mg, ~40% NHAc) which was used for the next step. The compound (10 mg, 8.72 μmol) was dissolved in triethylsilane (1 mL) and trifluoroacetic acid (1 mL) added at room temperature, after which the mixture was stirred for 1 h at room temperature. After the reaction was complete, the mixture was concentrated on a rotary evaporator and the residue fractionated by Sephadex® LH-20 column chromatography, using water as the eluent, to give Compound **41** as a white powder with a mixture of thiol-disulfide products (7.7 mg). The *N*-acetylation ratio was determined by ^{1}H NMR.

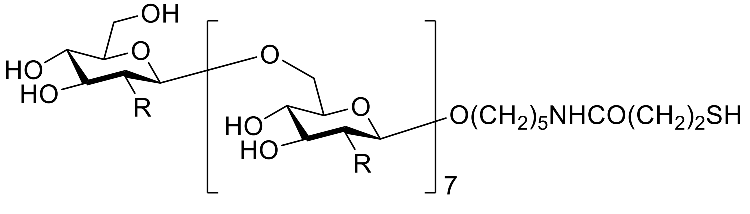

R= ~40% NHAc+ ~60% NH_2

Compound 42. Compound **33** (83 mg, 48.2 μmol) was dissolved in MeOH (3 mL) and water (3 mL) at room temperature. To this was added $NaHCO_3$ (32 mg, 0.385 mmol) and acetic anhydride (0.29 mL, 0.145 mmol, 0.5 M in dioxane) and the mixture stirred continuously overnight at room temperature. After the reaction was complete, the mixture was concentrated on a rotary evaporator and the residue fractionated by Toyopearl HW-40 column chromatography, using 0.1 M AcOH as the eluent to give Compound **37** as a white powder (65 mg, ~40% NHAc) and this compound was used for the next step. The compound (20.0 mg) was dissolved in triethylsilane (1 mL), trifluoroacetic acid (1 mL) was added at room temperature, and the mixture was stirred for 1 h at room temperature. After the reaction was complete, the mixture was concentrated on a rotary evaporator and the residue fractionated by Sephadex® LH-20 column chromatography, using water as the eluent to give Compound **42** as a white powder with a mixture of thiol-disulfide products (9.4 mg). The *N*-acetylation ratio determined by ^{1}H NMR.

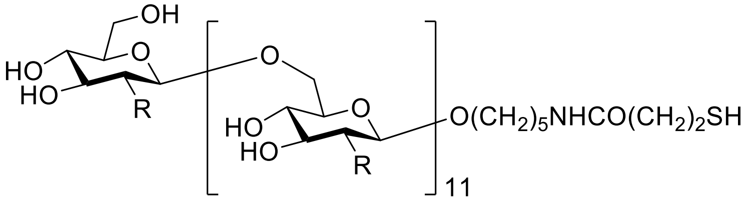

R= ~40% NHAc+ ~60% NH_2

Compound 43. Compound **34** (70 mg, 29.6 μmol) was dissolved in MeOH (3 mL) and water (3 mL) at room temperature. To this mixture $NaHCO_3$ (30 mg, 0.354 mmol) and acetic anhydride (0.27 mL, 0.133 mmol, 0.5 M in dioxane) were added and then stirred continuously overnight at room temperature. After the reaction was complete, the mixture was concentrated on a rotary evaporator and the residue fractionated by Toyopearl HW-40 column chromatography, using 0.1 M AcOH as the eluent to give compound **38** as a white powder (61 mg, ~40% NHAc). This compound (22.0 mg) was then dissolved in triethylsilane (1 mL) and trifluoroacetic acid (1 mL) added at room temperature. The mixture was stirred for 1 h at room temperature. After the reaction was complete, it was concentrated on a rotary evaporator and the residue was fractionated by Sephadex® LH-20 chromatography using water as the eluent to give Compound **43** as a white

powder with a mixture of thiol-disulfide products (8.9 mg). The *N*-acetylation ratio determined by ^{1}H NMR.

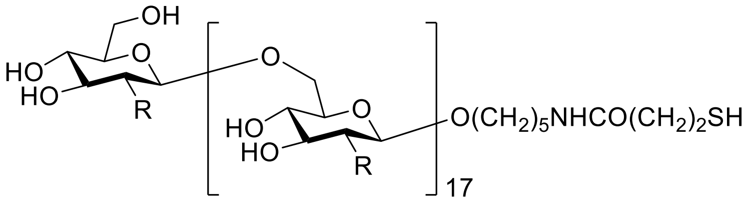

R= ~45% NHAc+ ~55% NH_2

Compound 44. Compound **35** (11 mg, 3.30 μmol) was dissolved in MeOH (1 mL) and water (1 mL) at room temperature. To this was added $NaHCO_3$ (5.6 mg, 59.4 μmol) and acetic anhydride (0.10 mL, 19.8 μmol, 0.2 M in dioxane) and then stirred overnight at room temperature. After the reaction was complete, the mixture was concentrated on a rotary evaporator and the residue fractionated by Toyopearl HW-40 column chromatography using 0.1 M AcOH as the eluent to give Compound **39** as a white powder (9.9 mg, ~40% NHAc) and the compound was used for the next step. The compound (8.2 mg) was dissolved in triethylsilane (1 mL), trifluoroacetic acid (1 mL) was added at room temperature, and the mixture was stirred for 1 h at room temperature. After the reaction was complete, the mixture was concentrated on a rotary evaporator and the residue fractionated by Sephadex® LH-20 column chromatography using water as the eluent to give Compound **44** as awhite powder with a mixture of thiol-disulfide products (3.1 mg). The *N*-acetylation ratio determined by ^{1}H NMR.

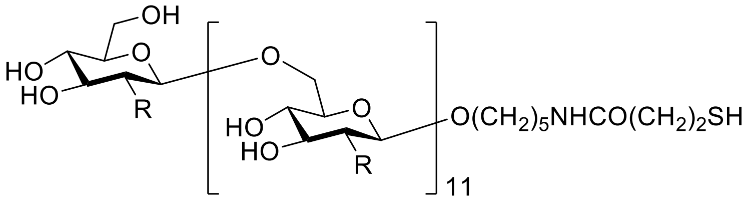

R= ~60% NHAc+ ~40% NH_2

Compound 45. Compound **34** (70 mg, 29.6 μmol) was dissolved in MeOH (3 mL) and water (3 mL) at room temperature. To this was then added $NaHCO_3$ (30 mg, 0.354 mmol) and acetic anhydride (0.27 mL, 0.133 mmol, 0.5 M in dioxane) and the mixture stirred overnight at room temperature. After the reaction was complete, the mixture was concentrated on a rotary evaporator and the residue fractionated by Toyopearl HW-40 column chromatography using 0.1 M AcOH as the eluent to give compound **40** as a white powder (61 mg, ~40% NHAc), which was used for the next step. The compound (20.3 mg) was dissolved in triethylsilane (1 mL) and trifluoroacetic acid (1 mL) was added at room temperature. The mixture was then stirred for 1 h at room temperature. After the reaction was complete, the mixture was concentrated on a rotary evaporator and the residue fractionated by Sephadex® LH-20 column chromagraphy using water as the eluent to give Compound **45** as a white powder with a mixture of thiol-disulfide products (9.4 mg). The *N*-acetylation ratio determined by ^{1}H NMR.

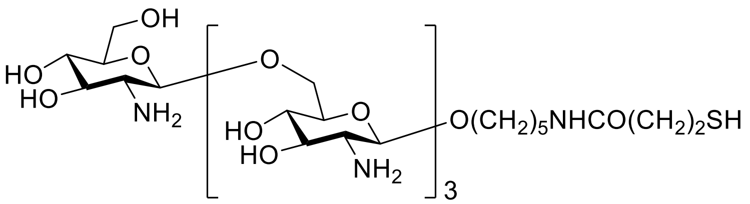

Compound 46. Compound **32** (14.0 mg, 13.0 μmol) was dissolved in triethylsilane (1 mL), trifluoroacetic acid (1 mL) was added at room temperature and the mixture was stirred for 1h at room temperature. After the reaction was complete, the reaction mixture was concentrated on a rotary evaporator and the residue fractionated by Sephadex® LH-20 column chromatography, using water as the eluent to give Compound **46** as a white powder with a mixture of thiol-disulfide products (10.3 mg, 95%). White solid; ^{1}H NMR (600 MHz, D_2O): δ 4.80-4.74 (m, 4H, 4xC1-H_β), 4.28-4.25 (m, 3H, 3/2CH_2), 3.99-3.90 (m, 5H, 2xCH_2, 1/2$CH_{2\text{-linker}}$), 3.78 (dd, *J* =12.6, 4.8 Hz, 1H, 1/2CH_2), 3.72-3.67 (m, 8H, 7xCH, 1/2$CH_{2\text{-linker}}$), 3.60-3.47 (m, 5H, 5/2xCH_2), 3.22 (t, *J* = 6.6 Hz, 2H, $CH_{2\text{-linker}}$), 3.12-3.08 (m, 3H, 3xCH), 3.03 (dd, *J* = 10.2, 8.4 Hz, 1H, CH), 2.78 (t, *J* = 6.6 Hz, 2H, $CH_{2\text{-linker}}$), 2.54 (t, *J* = 6.6 Hz, 2H, $CH_{2\text{-linker}}$), 1.68-1.63 (m, 2H, $CH_{2\text{-linker}}$), 1.57-1.52 (m, 2H, $CH_{2\text{-linker}}$), 1.41-1.36 (m, 2H, $CH_{2\text{-linker}}$); ^{13}C NMR (150 MHz, D_2O): δ = 174.2, 99.4, 99.2, 99.1, 98.9, 76.2, 74.8, 74.7, 72.0, 71.8, 70.7, 69.7, 69.6, 69.6, 69.5, 68.5, 68.4, 60.3, 55.6, 55.5, 55.5, 39.4, 39.1, 28.3, 28.1, 22.4, 20.0; HRMS (ESI-TOF) m/e: Calcd for $C_{32}H_{61}N_5O_{18}S$ $[M+H]^+$: 836.3805 Found 836.3835.

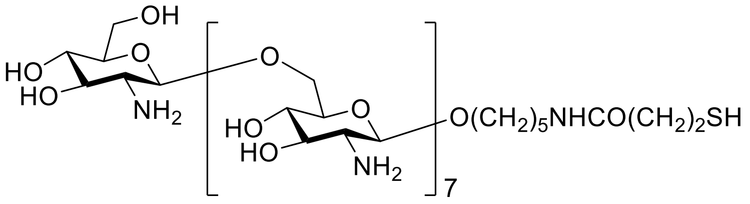

Compound 47. Compound **33** (9.0 mg, 5.22 μmol) was dissolved in triethylsilane (1 mL), trifluoroacetic acid (1 mL) was added at room temperature, and the mixture was stirred for 1 h at room temperature. After the reaction was complete, the mixture was concentrated on a rotary evaporator and the residue fractionated by Sephadex® LH-20 column chromatography using water as the eluent to give Compound **47** as a white powder with a mixture of thiol-disulfide products (7.3 mg, 94%). White solid; ^{1}H NMR (600 MHz, D_2O): 4.80-4.79 (m, 8H, 8xC1-H_β), 4.32-4.25 (m, 7H, 7/2xCH_2), 3.99-3.89 (m, 9H, 4xCH_2, 1/2x$CH_{2\text{lnnker}}$), 3.78 (dd, *J* =12.6, 5.4 Hz, 1H, 1/2xCH_2), 3.73-3.66 (m, 16H, 15xCH, 1/2x$CH_{2\text{linker}}$), 3.60-3.49 (m, 9H, 9/2xCH_2), 3.22 (t, *J* = 7.2 Hz, 2H, $CH_{2\text{linker}}$), 3.15-3.09 (m, 7H, 7xCH), 3.04 (dd, *J* = 10.8, 8.4 Hz, 1H, CH), 2.78 (t, *J* = 6.6 Hz, 2H, $CH_{2\text{linker}}$), 2.54 (t, *J* = 6.6 Hz, 2H, $CH_{2\text{linker}}$), 1.68-1.63 (m, 2H, $CH_{2\text{linker}}$), 1.57-1.52 (m, 2H, $CH_{2\text{linker}}$), 1.41-1.36 (m, 2H, $CH_{2\text{linker}}$); ^{13}C NMR (150 MHz, D_2O): δ = 174.2, 99.6, 99.3, 99.2, 99.1, 98.9, 76.2, 74.9, 74.8, 74.7, 74.6, 71.9, 71.7, 70.7, 69.6, 69.6, 69.5, 68.6, 68.5, 68.3, 60.3, 55.6, 55.5, 55.4, 39.3, 39.1, 28.2, 28.0, 22.4, 20.0; HRMS (ESI-TOF) m/e: Calcd for $C_{56}H_{105}N_9O_{34}S$ $[M+3H]^{3+}$: 494.2234 Found 494.2234.

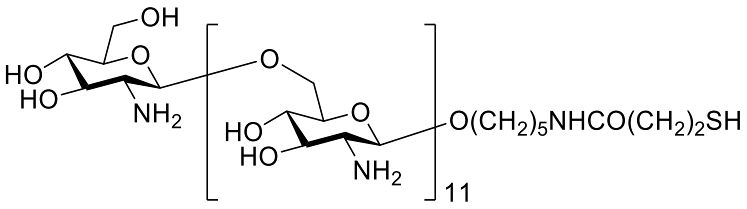

Compound 48. Compound **34** (6.5 mg, 2.75 μmol) was dissolved in triethylsilane (1 mL), trifluoroacetic acid (1 mL) was added at room temperature and the mixture was stirred for 1 h at room temperature. After the reaction was complete, the mixture was concentrated on a rotary evaporator and the residue fractionated by Sephadex® LH-20 column chromatography using water as the eluent to give Compound **48** as a white powder with a mixture of thiol-disulfide products (5.3 mg, 91%). White solid; ^{1}H NMR (600 MHz, D_2O): 4.80-4.77 (m, 12H, 12xC1-$H_β$), 4.34-4.27 (m, 11H, 11/2xCH_2), 4.01-3.90 (m, 13H, 6xCH_2, 1/2x$CH_{2lnnker}$), 3.80 (dd, *J* =12.6, 5.4 Hz, 1H, 1/2xCH_2), 3.78-3.69 (m, 24H, 23xCH, 1/2x$CH_{2linker}$), 3.62-3.51 (m, 13H, 13/2xCH_2), 3.23 (t, *J* = 6.6 Hz, 2H, $CH_{2linker}$), 3.14-3.09 (m, 11H, 11xCH), 3.05 (dd, *J* = 10.2, 8.4 Hz, 1H, CH), 2.80 (t, *J* = 6.6 Hz, 2H, $CH_{2linker}$), 2.56 (t, *J* = 6.6 Hz, 2H, $CH_{2linker}$), 1.70-1.66 (m, 2H, $CH_{2linker}$), 1.59-1.54 (m, 2H, $CH_{2linker}$), 1.43-1.38 (m, 2H, $CH_{2linker}$); ^{13}C NMR (150 MHz, D_2O): δ = 174.2, 99.5, 99.3, 99.2, 98.9, 76.2, 74.9, 74.8, 74.7, 74.6, 72.0, 71.9, 71.8, 70.7, 69.7, 69.5, 68.7, 68.6, 68.4, 60.3, 55.6, 55.5, 39.4, 39.1, 28.3, 28.0, 22.4, 20.0; HRMS (ESI-TOF) m/e: Calcd for $C_{80}H_{149}N_{13}O_{50}S$ $[M+4H]^{4+}$: 531.9882 Found 531.9880.

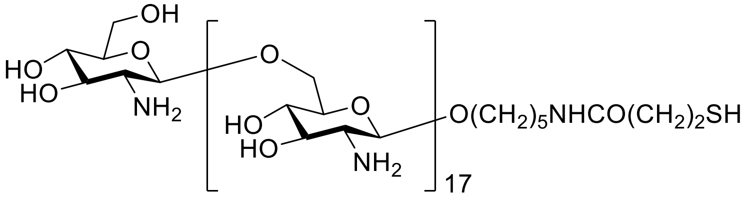

Compound 49. Compound **35** (10.0 mg, 3.0 μmol) was dissolved in triethylsilane (1 mL), trifluoroacetic acid (1 mL) was added at room temperature and the mixture was stirred for 1 h at room temperature. After the reaction was complete, the mixture was concentrated on a rotary evaporator and the residue fractionated by Sephadex® LH-20 column chromatography using water as the eluent to give Compound **49** as a white powder with a mixture of thiol-disulfide products (4.4 mg, 86%). White solid; ^{1}H NMR (600 MHz, D_2O): 4.80-4.75 (m, 18H, 18xC1-$H_β$), 4.32-4.26 (m, 17H, 17/2xCH_2), 4.00-3.89 (m, 19H 9xCH_2, 1/2x$CH_{2\text{-linker}}$), 3.79 (dd, *J* =12.6, 5.4 Hz, 1H, 1/2xCH_2), 3.72-3.69 (m, 36H, 35xCH, 1/2x$CH_{2\text{-linker}}$), 3.61-3.50 (m, 19H, 19/2xCH_2), 3.24-3.21 (m, 2H, $CH_{2\text{-linker}}$), 3.14-3.09 (m, 17H, 17xCH), 3.04 (dd, *J* = 10.2, 8.4 Hz, 1H, CH), 2.79 (t, *J* = 6.6 Hz, 2H, $CH_{2\text{-linker}}$), 2.55 (t, *J* = 6.6 Hz, 2H, $CH_{2\text{-linker}}$), 1.69-1.65 (m, 2H, $CH_{2\text{-linker}}$), 1.58-1.53 (m, 2H, $CH_{2\text{-linker}}$), 1.42-1.37 (m, 2H, $CH_{2\text{-linker}}$); ^{13}C NMR (150 MHz, D_2O): δ = 174.1, 99.7, 99.6, 99.4, 99.3, 99.0, 76.2, 74.9, 74.7, 74.7, 72.1, 71.9, 70.7, 69.6, 69.5, 68.6, 68.4, 60.3, 55.6, 55.5, 39.4, 39.2, 39.1, 28.2, 28.0, 22.4, 22.4, 20.0; HRMS (ESI-TOF) m/e: Calcd for $C_{116}H_{215}N_{19}O_{74}S$ $[M+6H]^{6+}$: 516.0634 Found 516.0635.

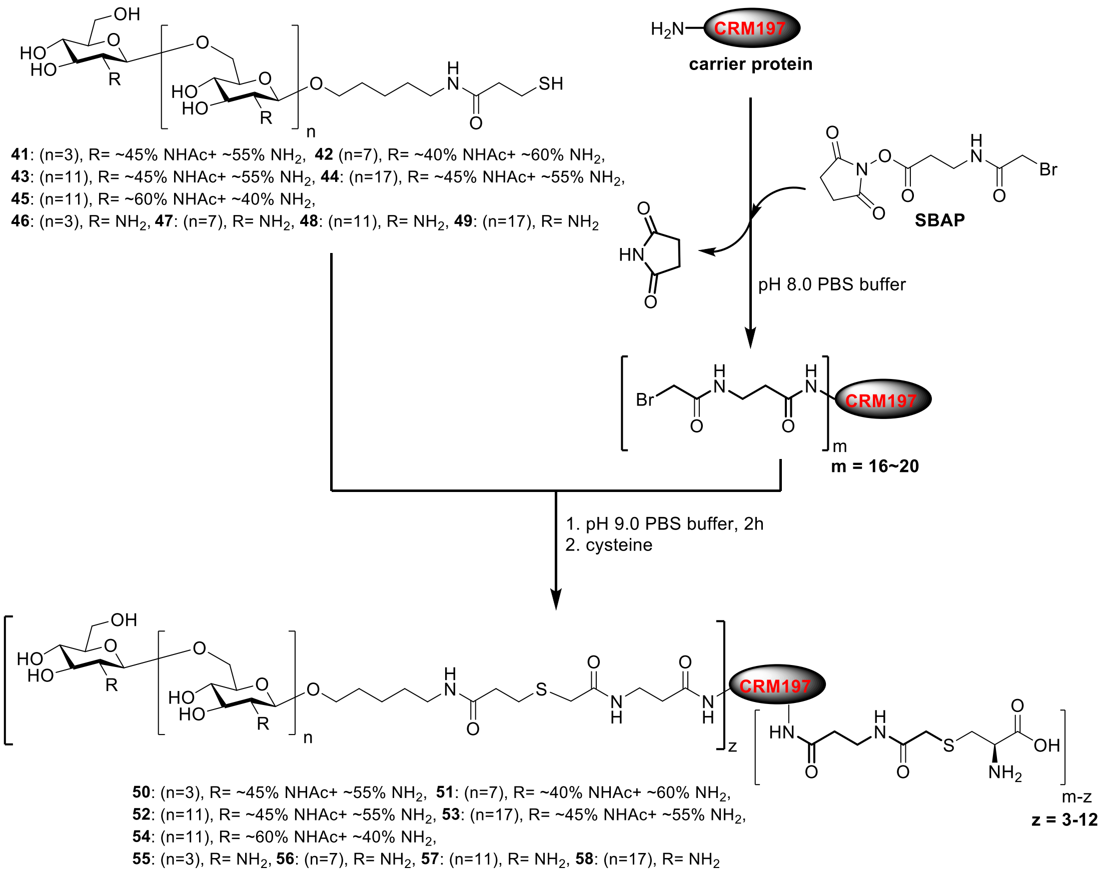

Figure S6. Synthesis of dPNAG tetra, octa, dodeca, octadecasaccharide-CRM197 as vaccine candidates

Table S1. Different lengths of dPNAG antigens on carrier protein CRM197

dPNAG glycan	**50**	**51**	**52**	**53**	**54**	**55**	**56**	**57**	**58**
Amount of protein (μg)	766	743	729	726	741	748	739	679	737
Ratio (Sugar:CRM197)	3.86	4.31	4.31	2.35	2.57	11.5	7.33	4.25	2.24
Amount of sugar (μg)	41.9	87.2	103.6	83.8	68.4	101.5	111.3	87.2	75.0

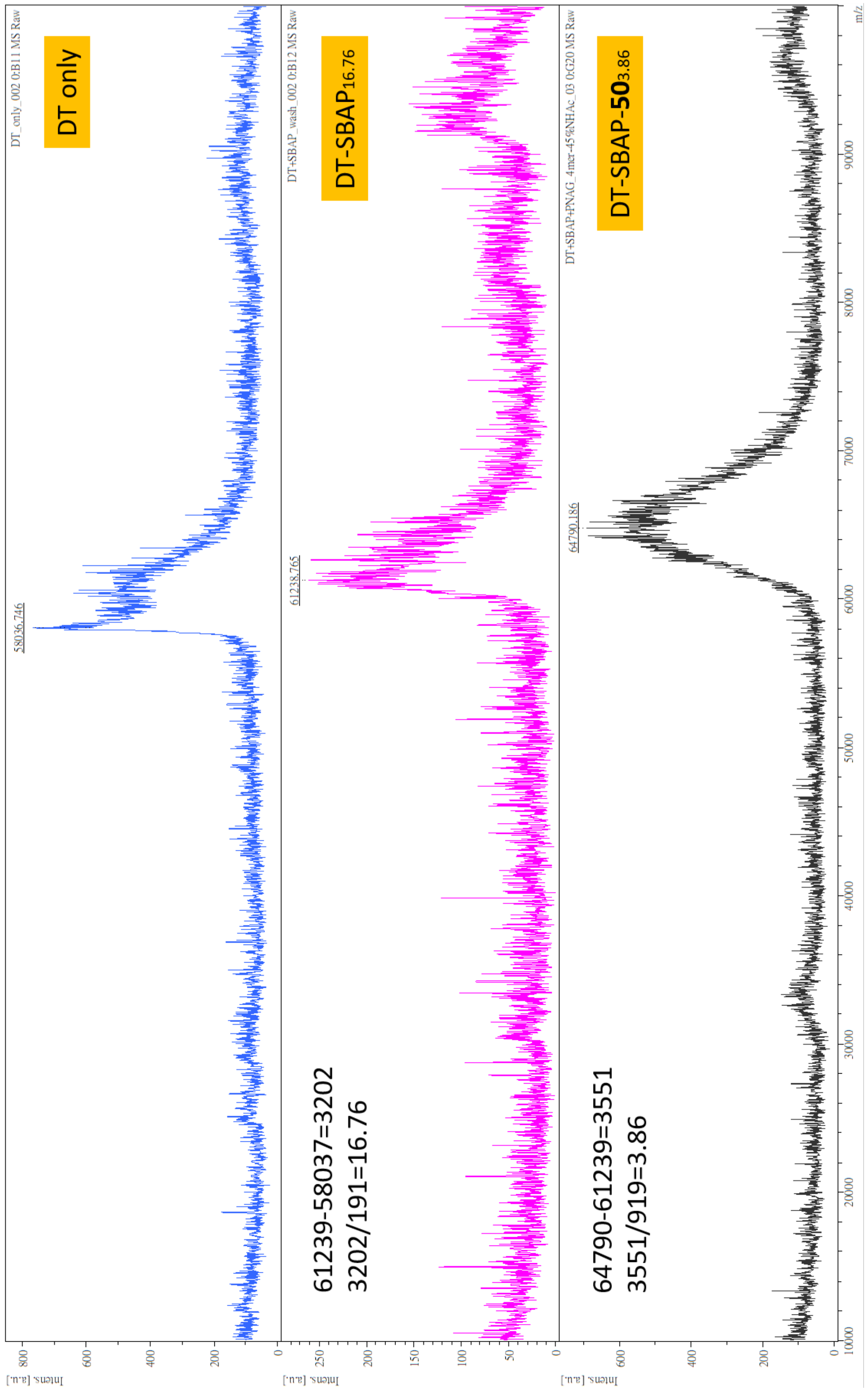
DT only
DT-SBAP16.76
DT-SBAP-50 3.86
DT_only_002 0:B11 MS Raw
DT+SBAP_wash_002 0:B12 MS Raw
DT+SBAP+PNAG_4mer-45%NHAc_03 0:G20 MS Raw
58036.746
61238.765
64790.186
61239-58037=3202
3202/191=16.76
64790-61239=3551
3551/919=3.86
Intens. [a.u.]
m/z

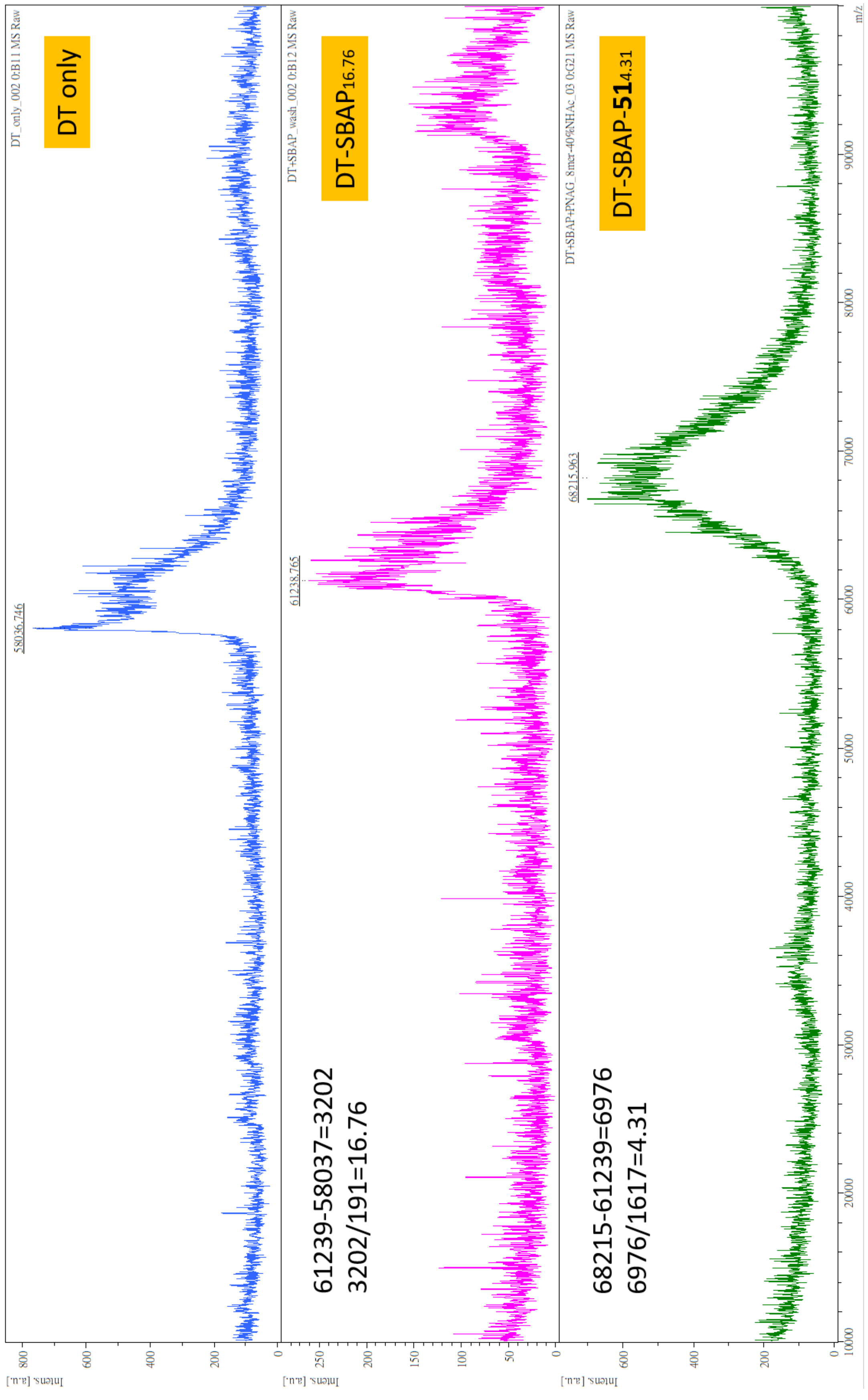
DT only
DT-SBAP16.76
DT-SBAP-514.31
58036.746
61238.765
68215.963
61239-58037=3202
3202/191=16.76
68215-61239=6976
6976/1617=4.31
DT_only_002 0:B11 MS Raw
DT+SBAP_wash_002 0:B12 MS Raw
DT+SBAP+PNAG_8mer-40%NHAc_03 0:G21 MS Raw
Intens. [a.u.]
m/z

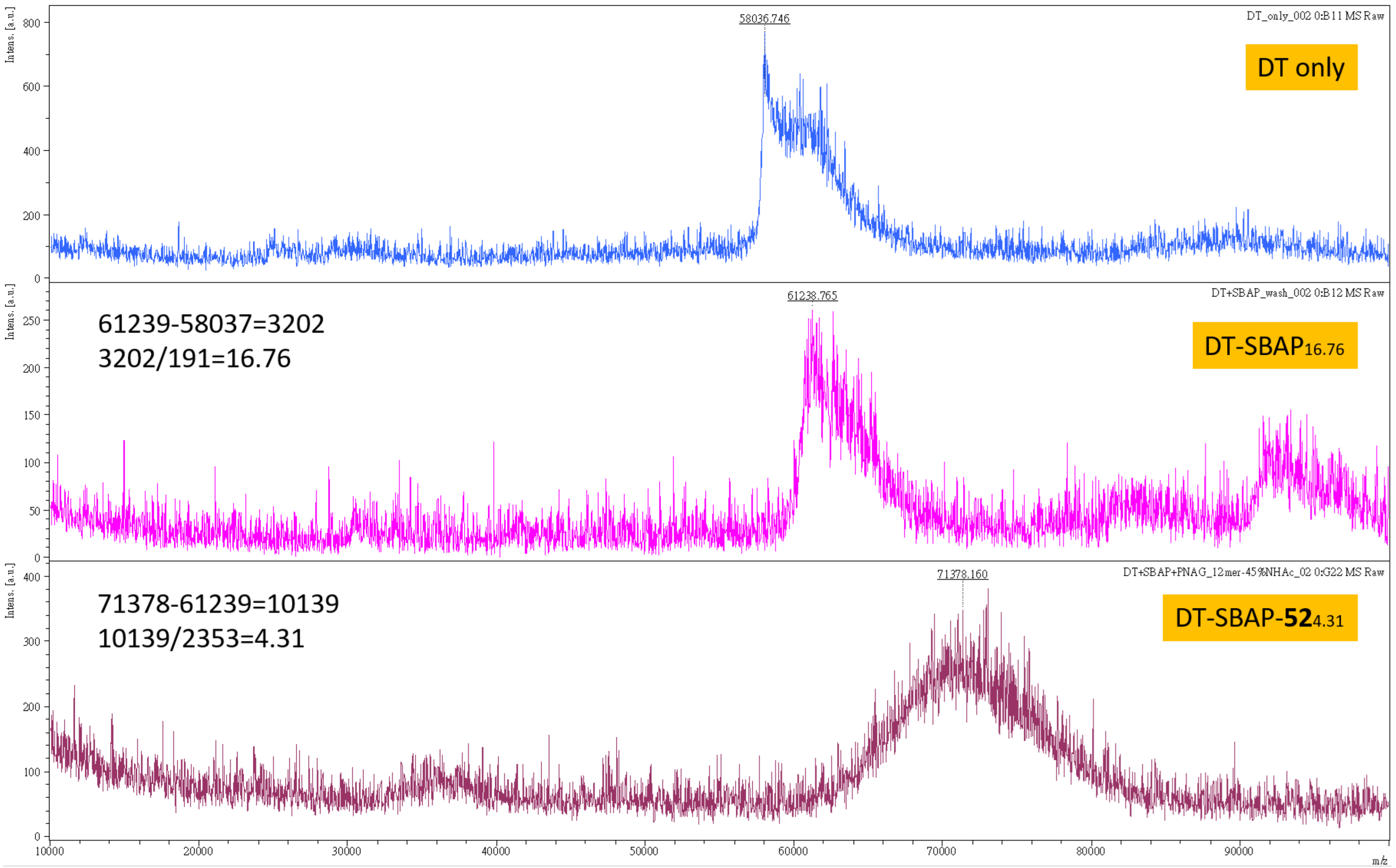
DT_only_002 0:B11 MS Raw
DT only
58036.746
DT+SBAP_wash_002 0:B12 MS Raw
DT-SBAP16.76
61238.765
61239-58037=3202
3202/191=16.76
DT+SBAP+PNAG_12mer-45%NHAc_02 0:G22 MS Raw
DT-SBAP-524.31
71378.160
71378-61239=10139
10139/2353=4.31
Intens. [a.u.]
m/z

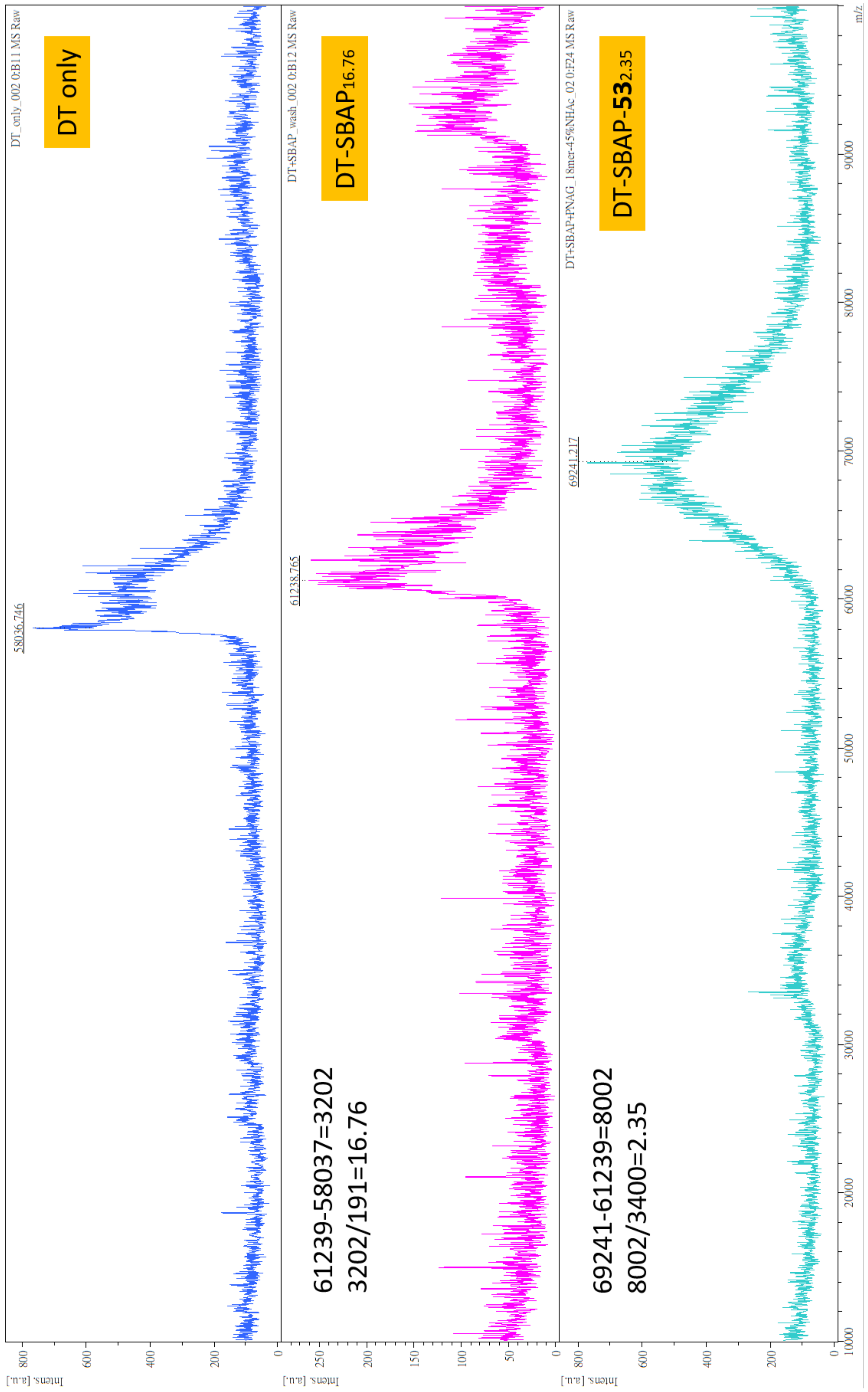
DT_only_002 0:B11 MS Raw
DT only
58036.746
DT+SBAP_wash_002 0:B12 MS Raw
DT-SBAP16.76
61238.765
61239-58037=3202
3202/191=16.76
DT+SBAP+PNAG_18mer-45%NHAc_02 0:F24 MS Raw
DT-SBAP-532.35
69241.217
69241-61239=8002
8002/3400=2.35
Intens. [a.u.]
m/z

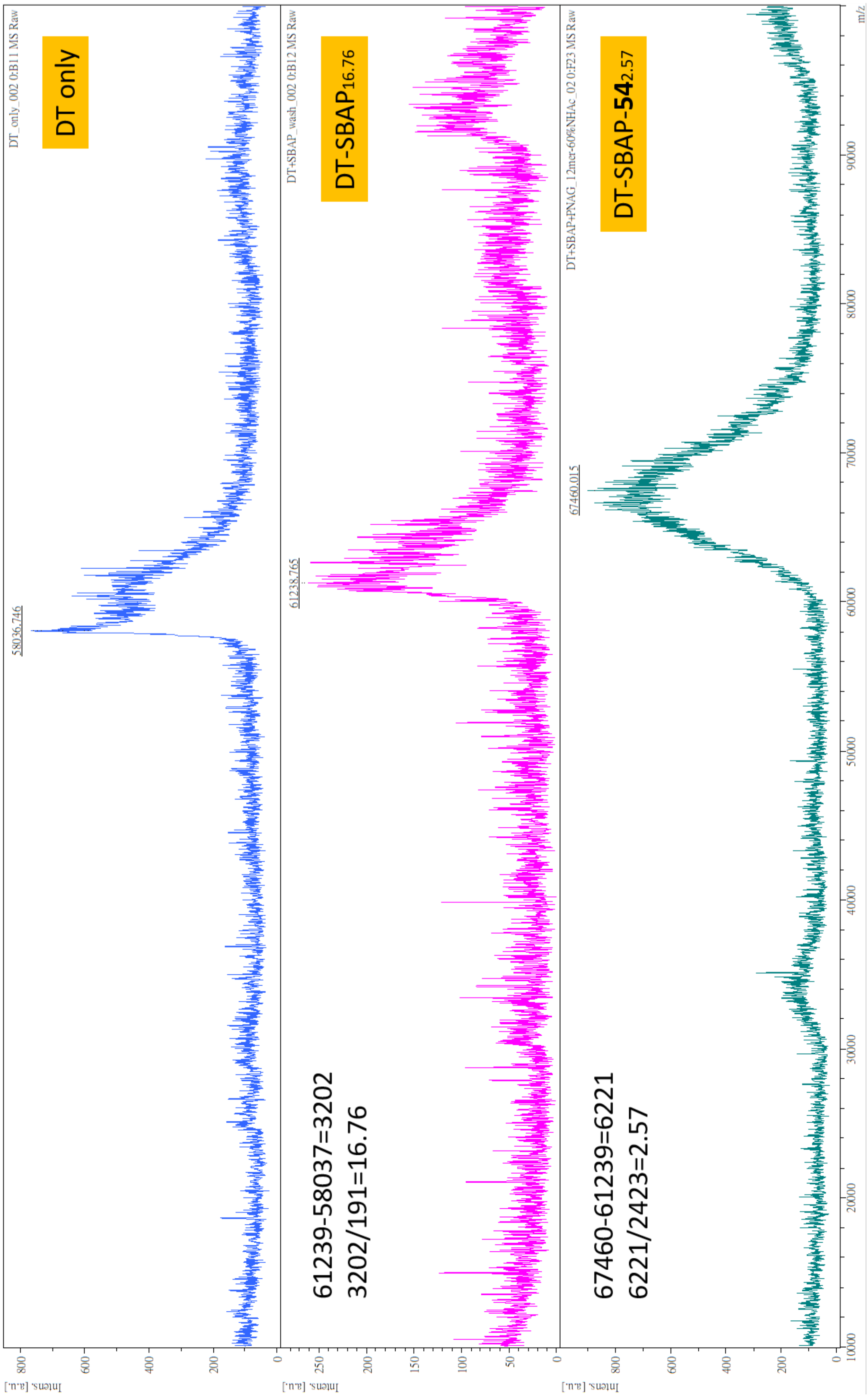

DT only
DT-SBAP16.76
DT-SBAP-542.57
DT_only_002 0:B11 MS Raw
DT+SBAP_wash_002 0:B12 MS Raw
DT+SBAP+PNAG_12mer-60%NHAc_02 0:F23 MS Raw
58036.746
61238.765
67460.015
61239-58037=3202
3202/191=16.76
67460-61239=6221
6221/2423=2.57
Intens. [a.u.]
m/z

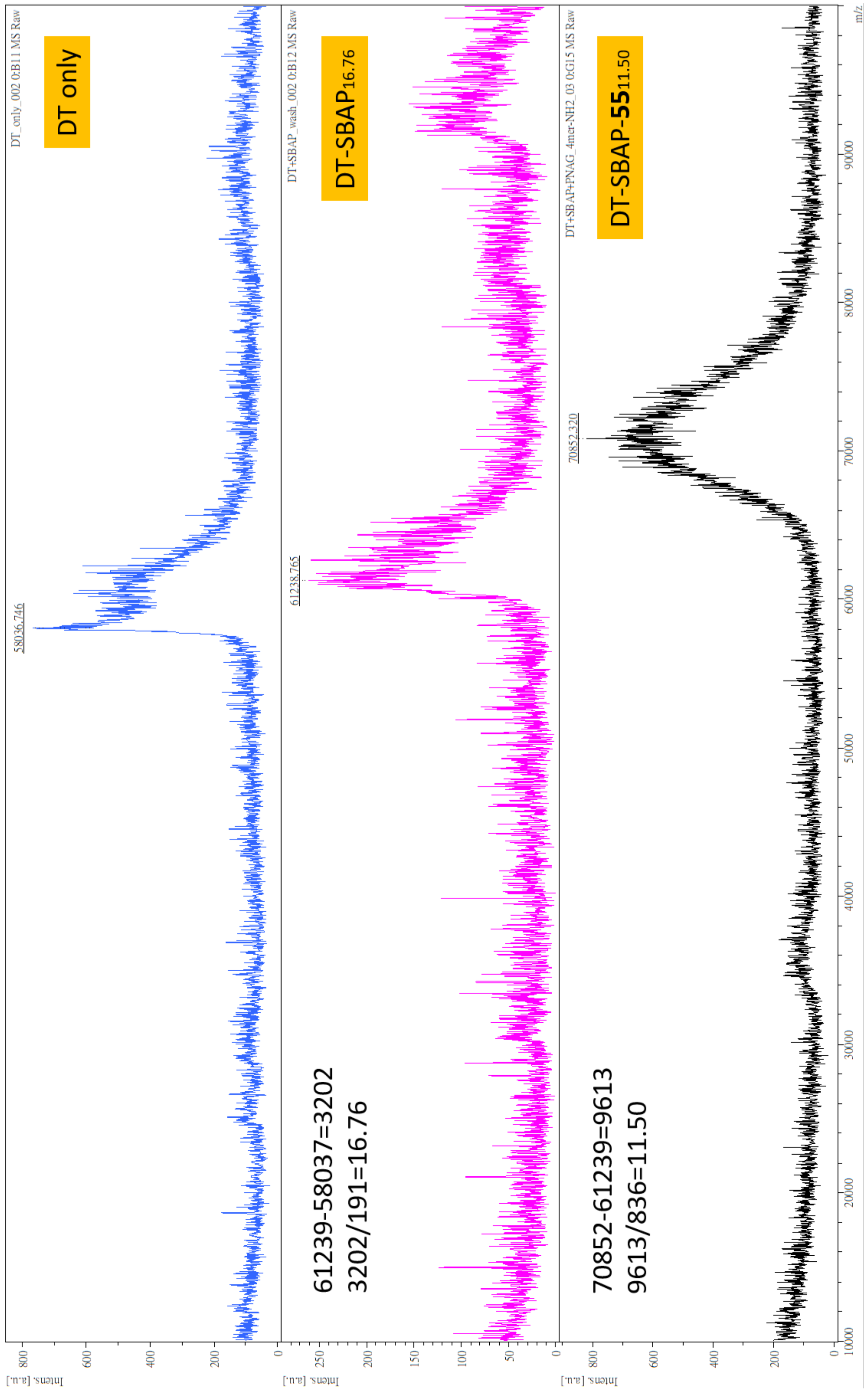

DT only
DT_only_002 0:B11 MS Raw
58036.746
DT-SBAP16.76
DT+SBAP_wash_002 0:B12 MS Raw
61238.765
61239-58037=3202
3202/191=16.76
DT-SBAP-5511.50
DT+SBAP+PNAG_4mer-NH2_03 0:G15 MS Raw
70852.320
70852-61239=9613
9613/836=11.50
Intens. [a.u.]
m/z

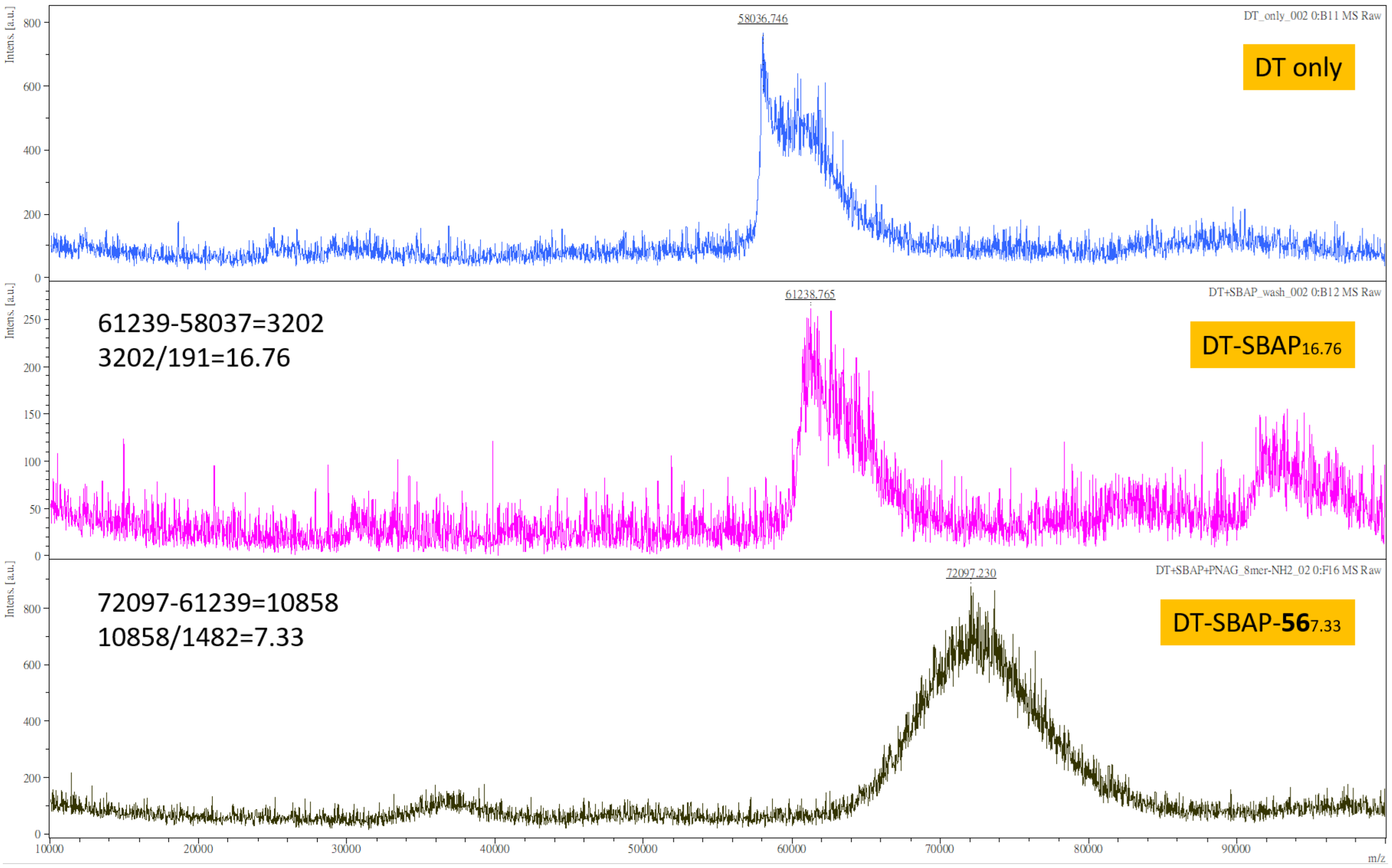
DT_only_002 0:B11 MS Raw
58036.746
DT only
DT+SBAP_wash_002 0:B12 MS Raw
61238.765
61239-58037=3202
3202/191=16.76
DT-SBAP16.76
DT+SBAP+PNAG_8mer-NH2_02 0:F16 MS Raw
72097.230
72097-61239=10858
10858/1482=7.33
DT-SBAP-**56**7.33
Intens. [a.u.]
m/z

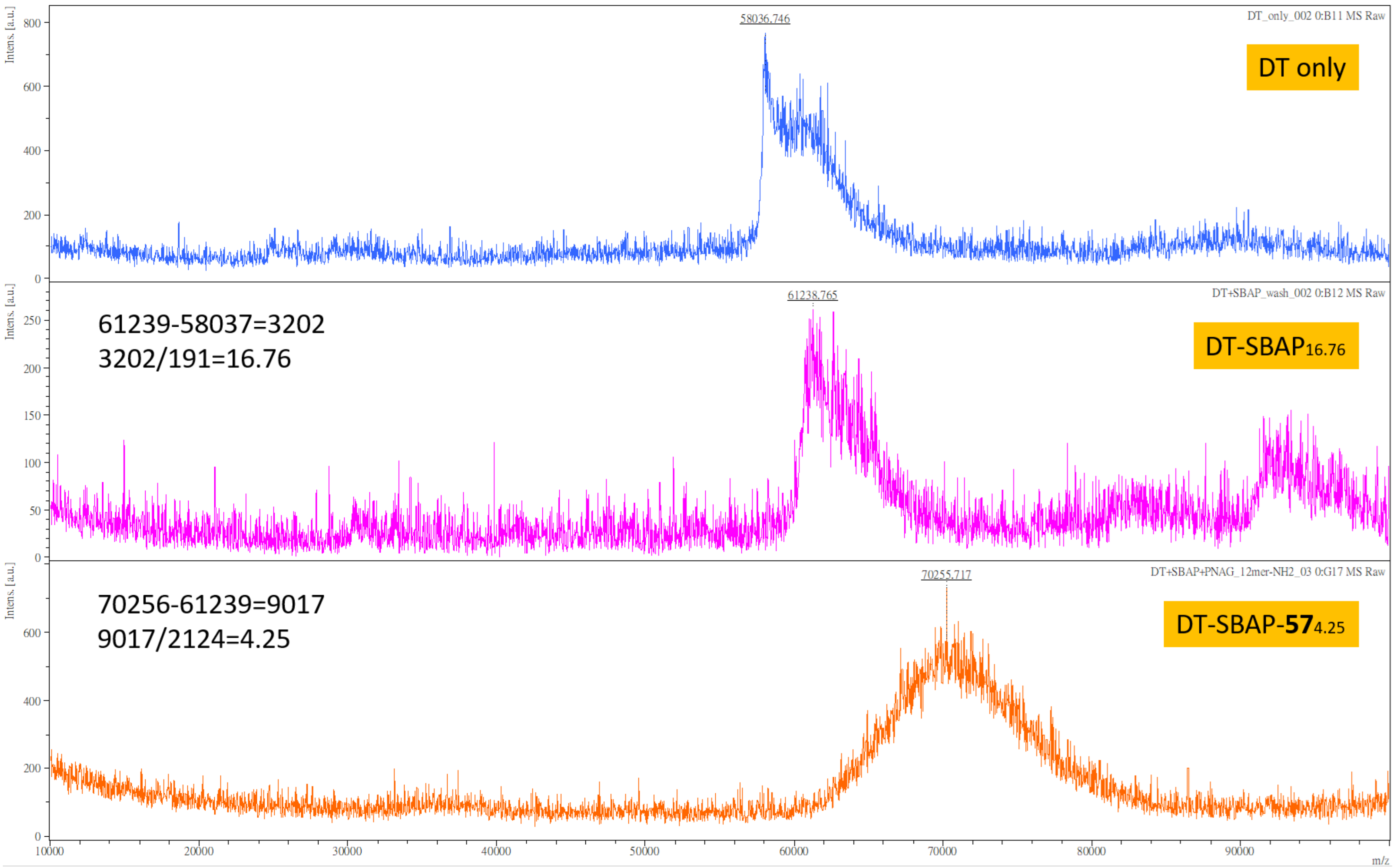

DT_only_002 0:B11 MS Raw
58036.746
DT only
DT+SBAP_wash_002 0:B12 MS Raw
61238.765
61239-58037=3202
3202/191=16.76
DT-SBAP16.76
DT+SBAP+PNAG_12mer-NH2_03 0:G17 MS Raw
70255.717
70256-61239=9017
9017/2124=4.25
DT-SBAP-57 4.25
Intens. [a.u.]
m/z

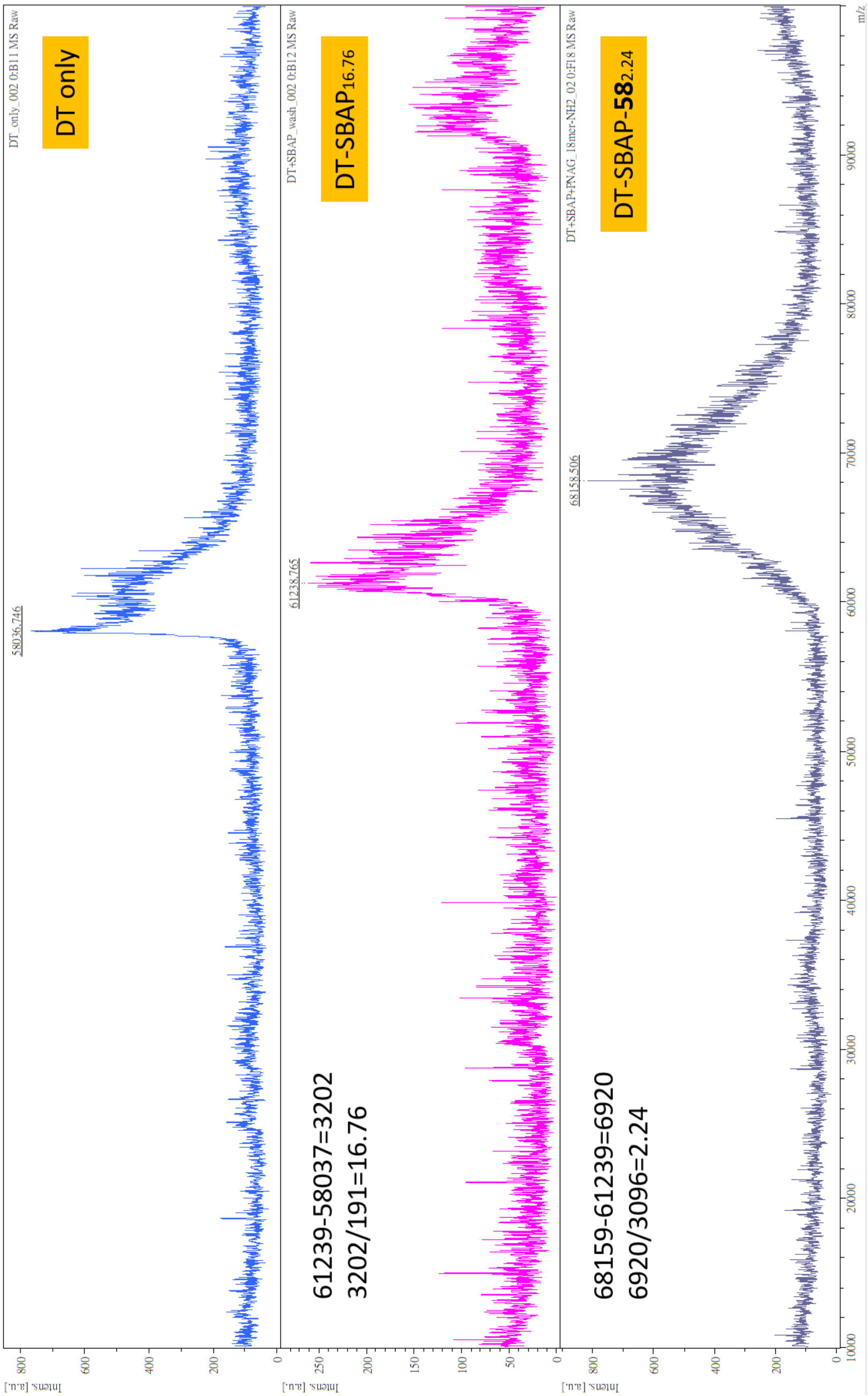

DT only
DT_only_002 0:B11 MS Raw
58036.746
DT-SBAP16.76
DT+SBAP_wash_002 0:B12 MS Raw
61238.765
61239-58037=3202
3202/191=16.76
DT-SBAP-58 2.24
DT+SBAP+PNAG_18mer-NH2_02 0:F18 MS Raw
68158.506
68159-61239=6920
6920/3096=2.24
Intens. [a.u.]
m/z

^{1}H and ^{13}C NMR Spectra (Figure S7- S85)

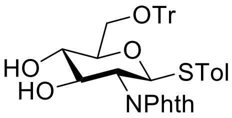

4

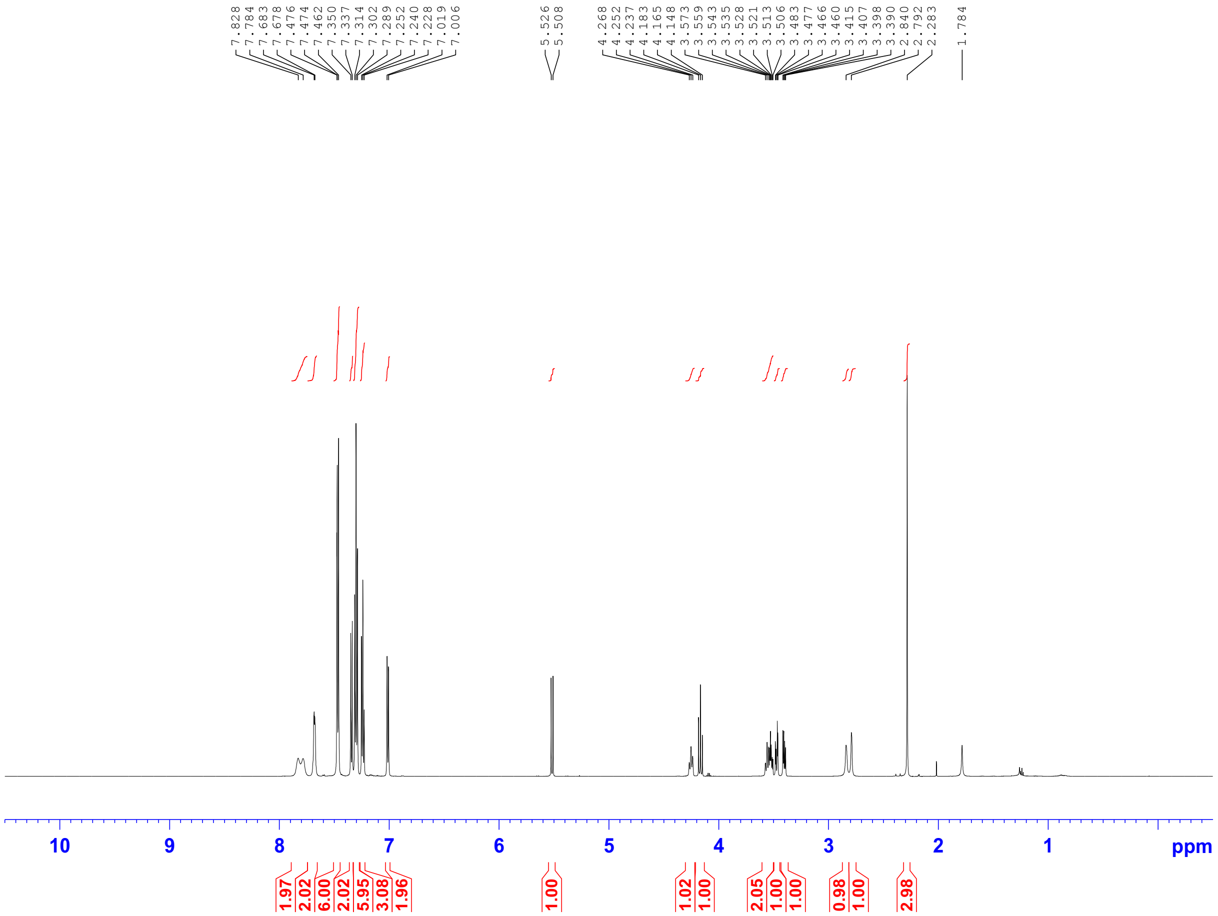

Figure S7. ^{1}H NMR spectrum of Compound 4

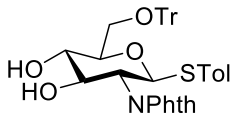

4

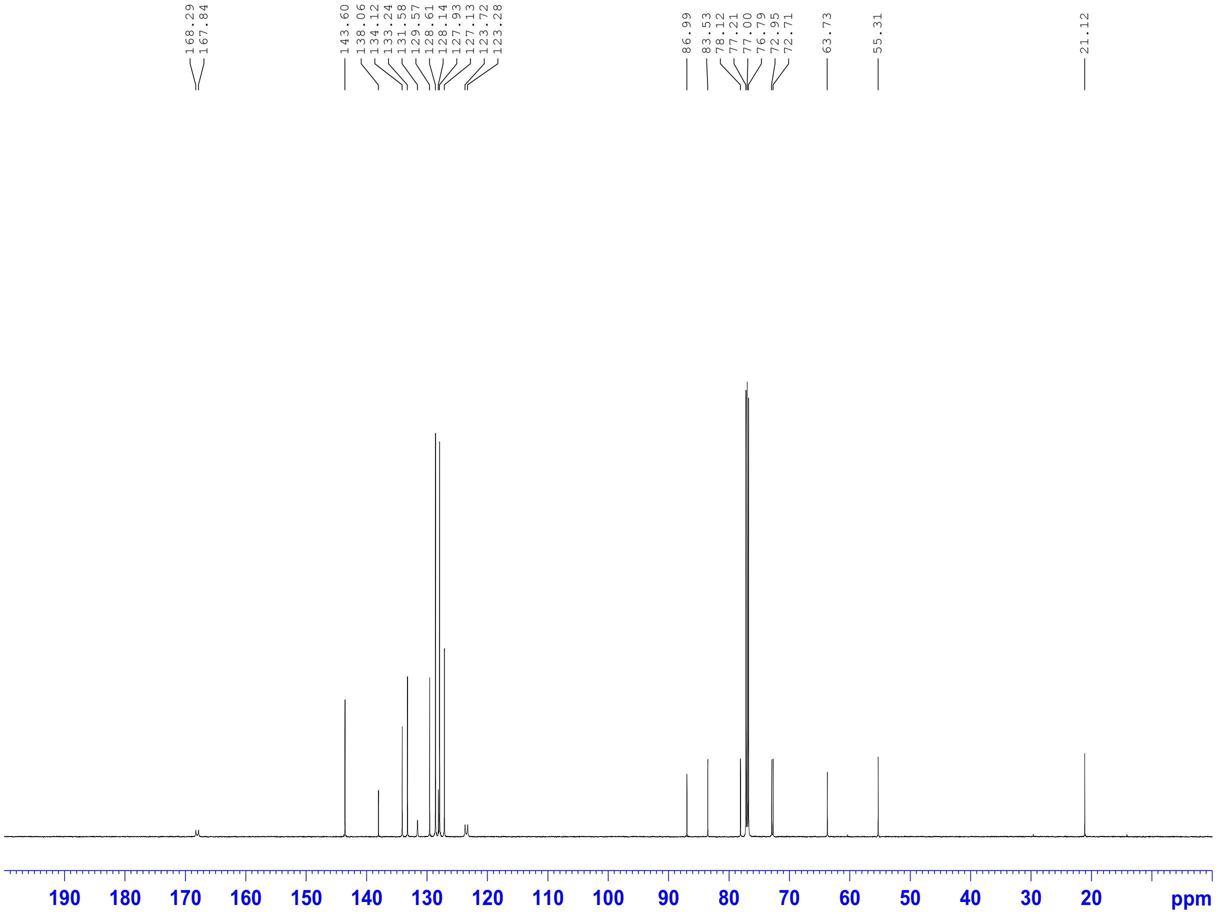

Figure S8. ^{13}**C NMR spectrum of Compound 4**

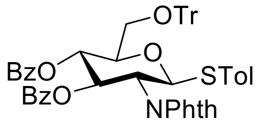

5

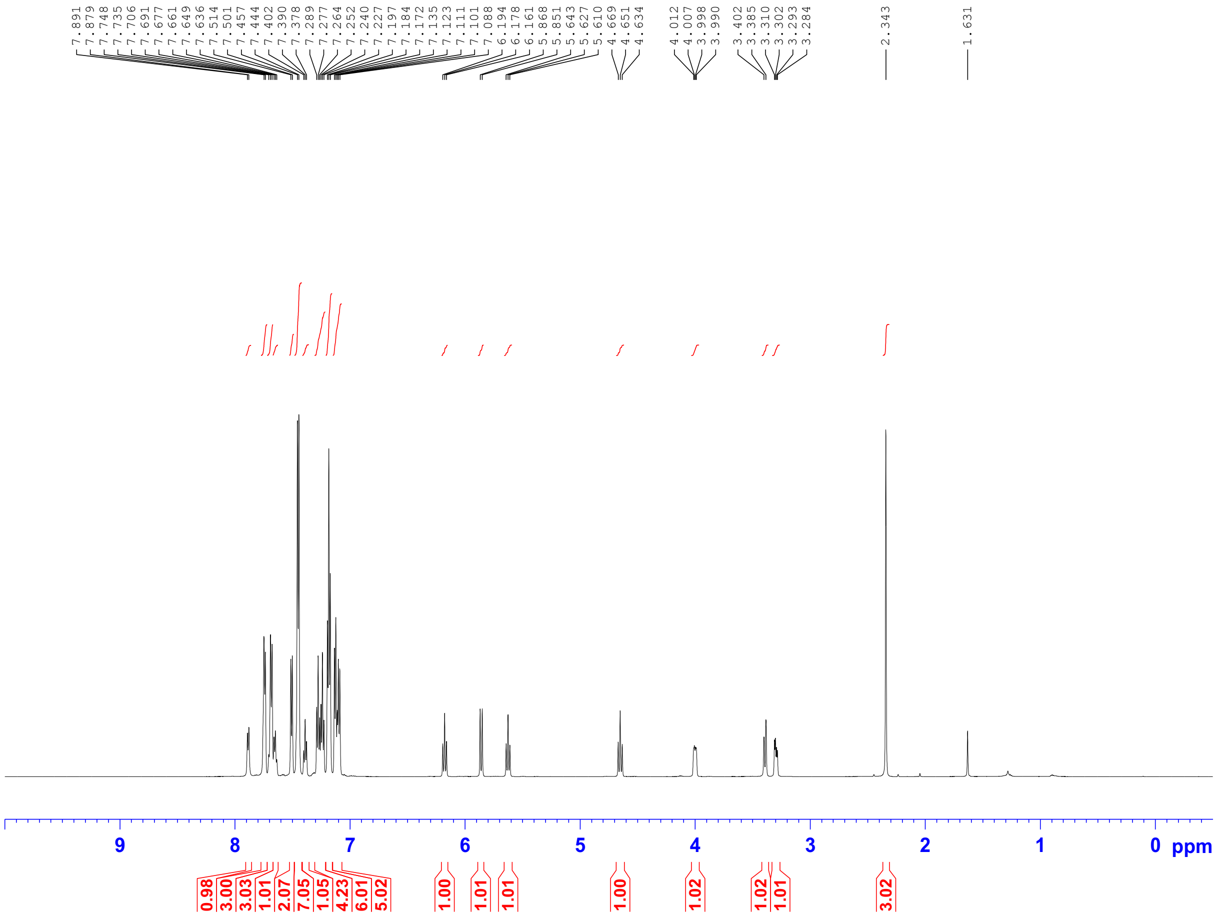

Figure S9. [1]**H NMR spectrum of Compound 5**

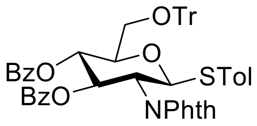

5

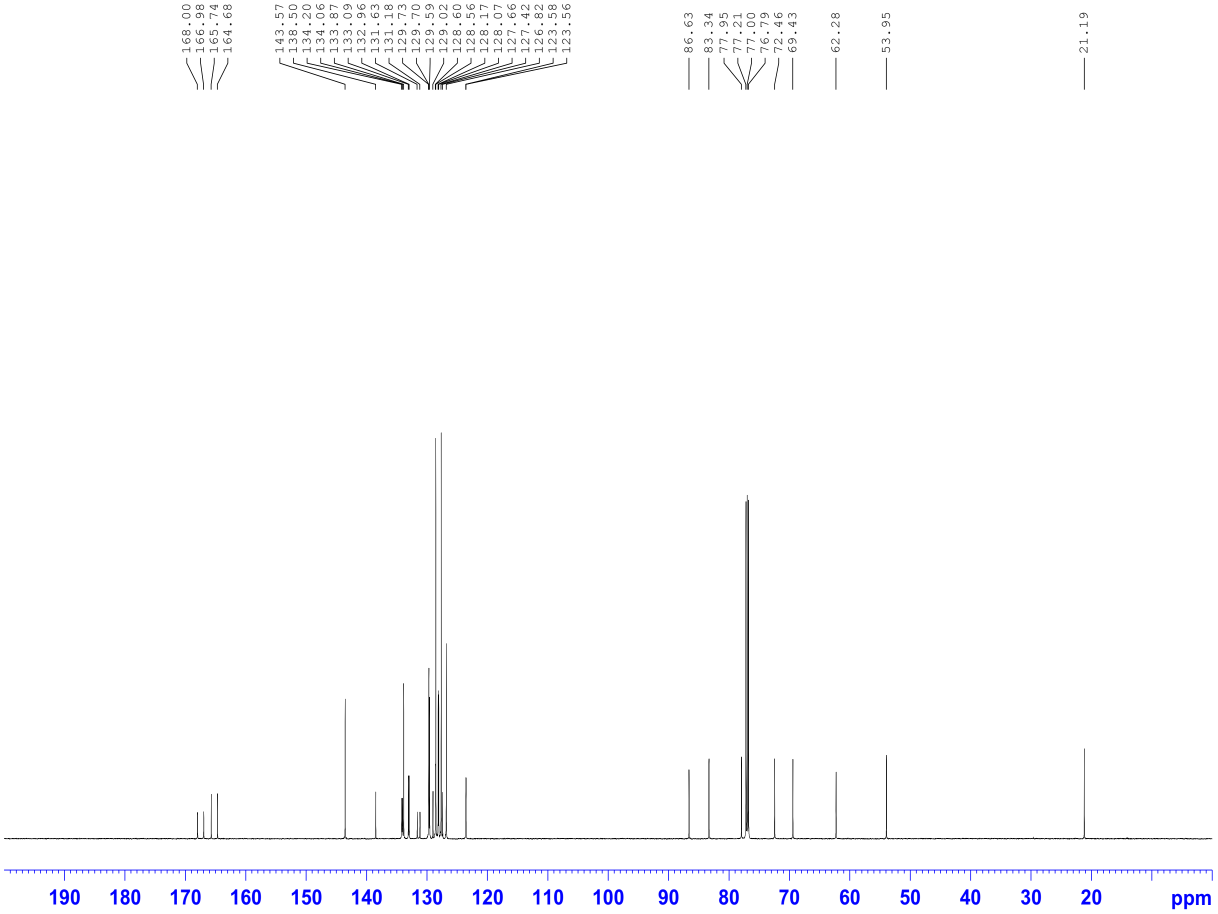

Figure S10. ^{13}**C NMR spectrum of Compound 5**

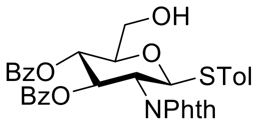

6

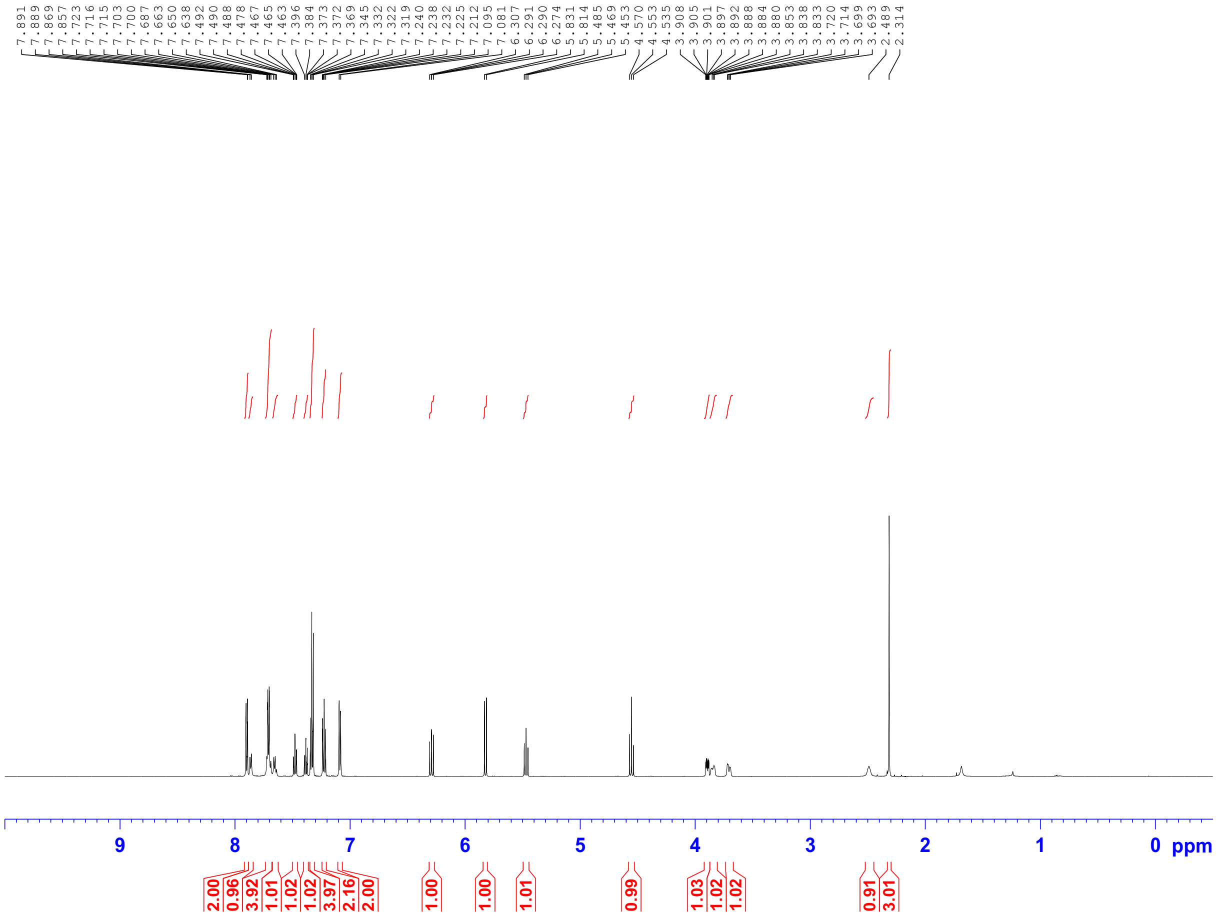

Figure S11. ¹H NMR spectrum of Compound 6

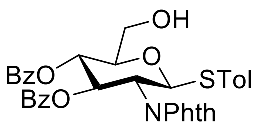

6

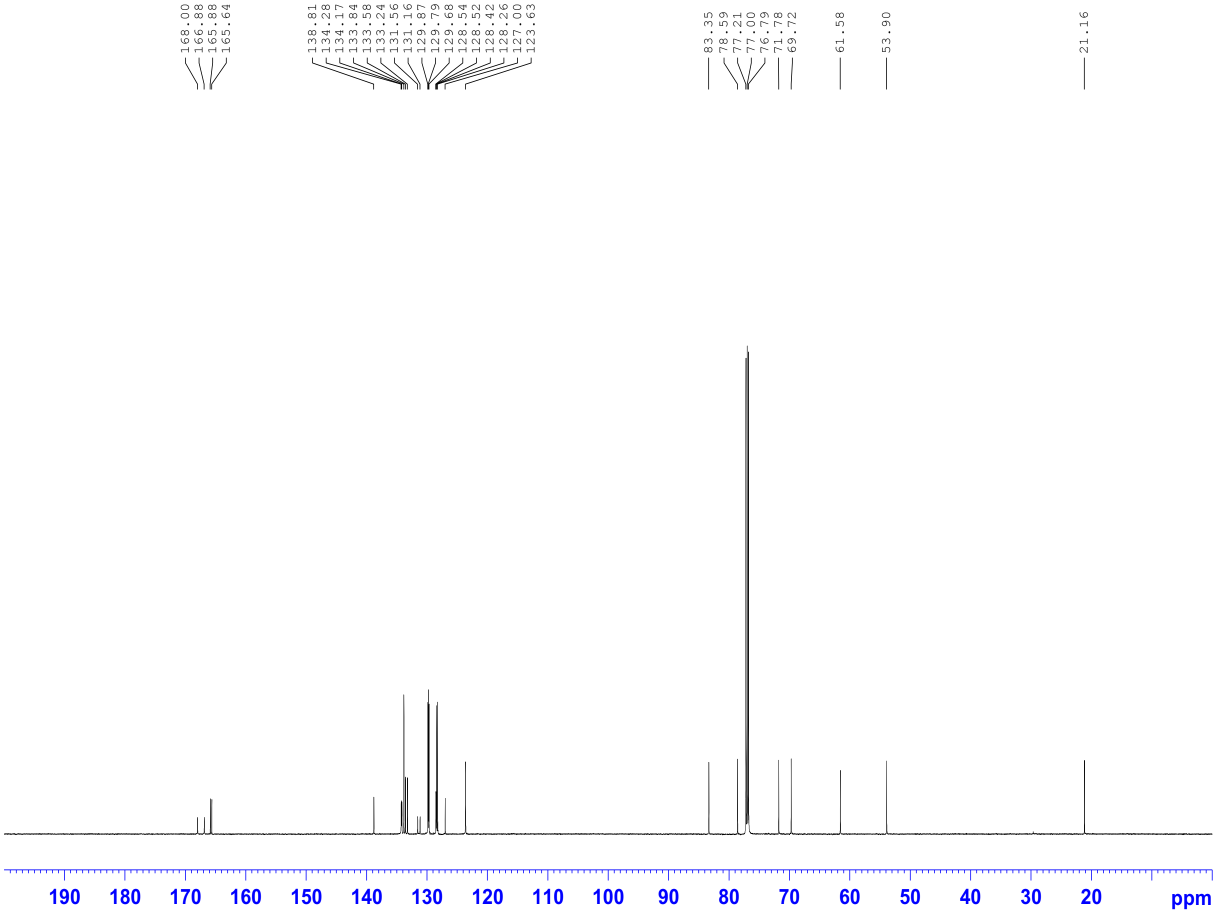

Figure S12. ^{13}C NMR spectrum of Compound 6

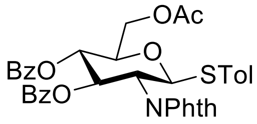

7

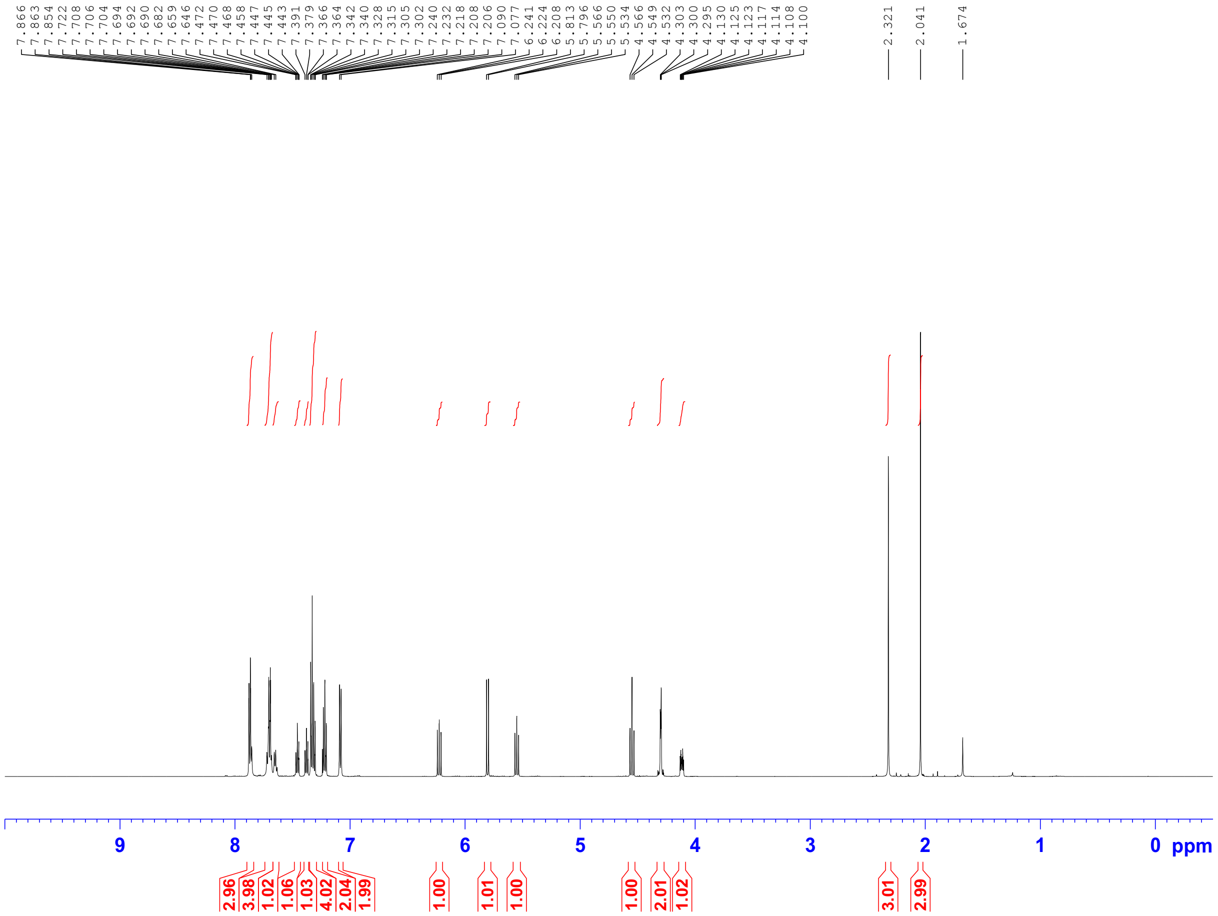

Figure S13. ^{1}H NMR spectrum of Compound 7

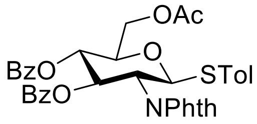

7

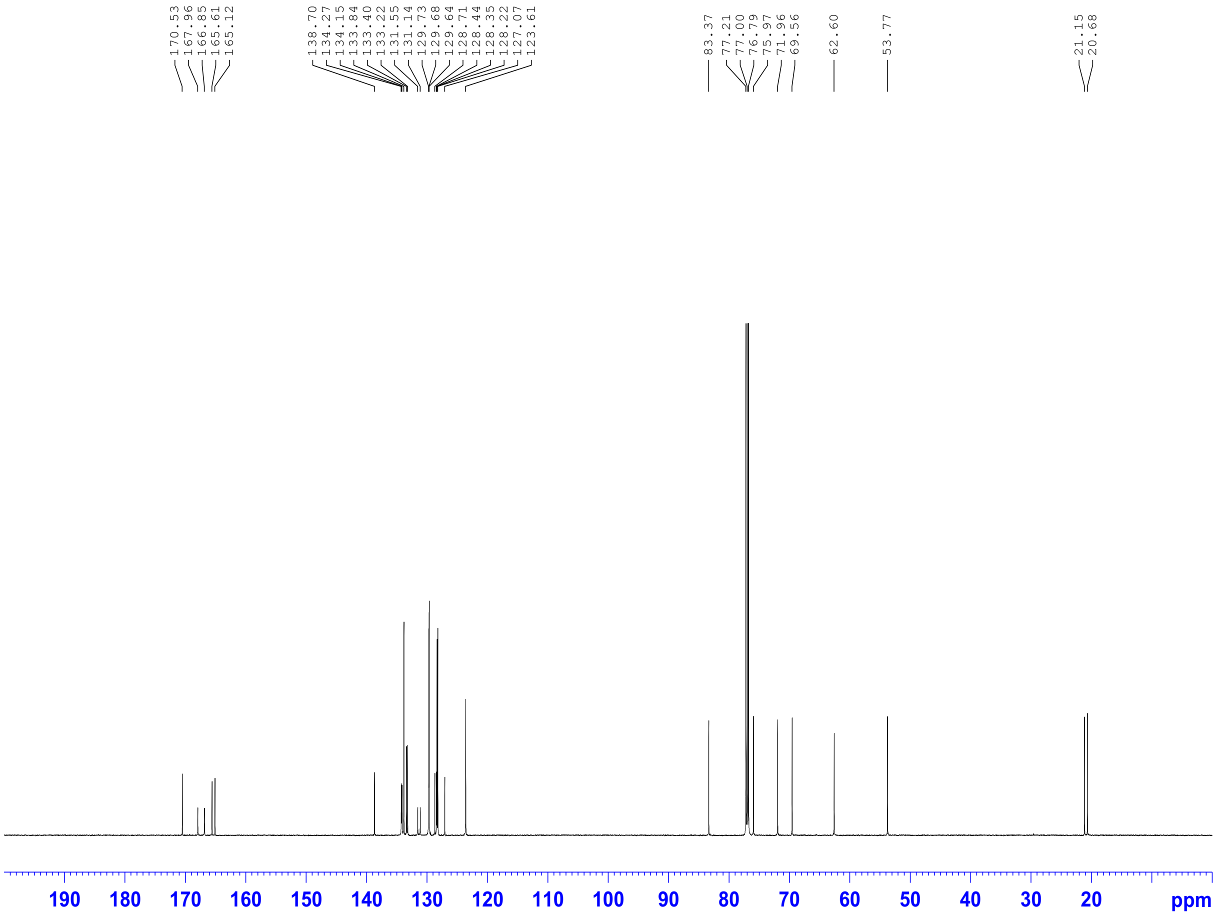

Figure S14. ^{13}C NMR spectrum of Compound 7

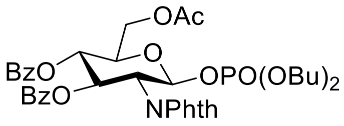

8

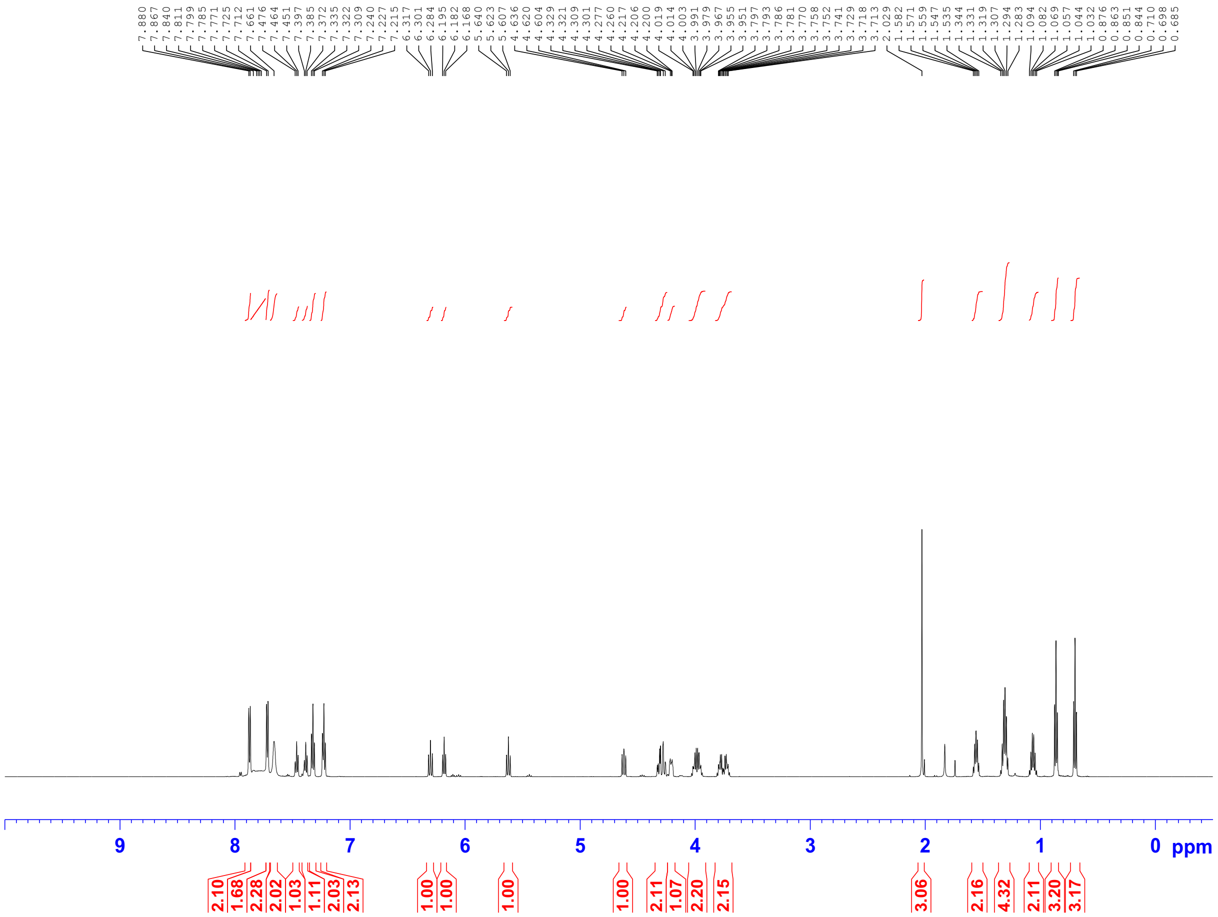

Figure S15. ^{1}H NMR spectrum of Compound 8

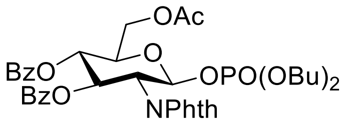

8

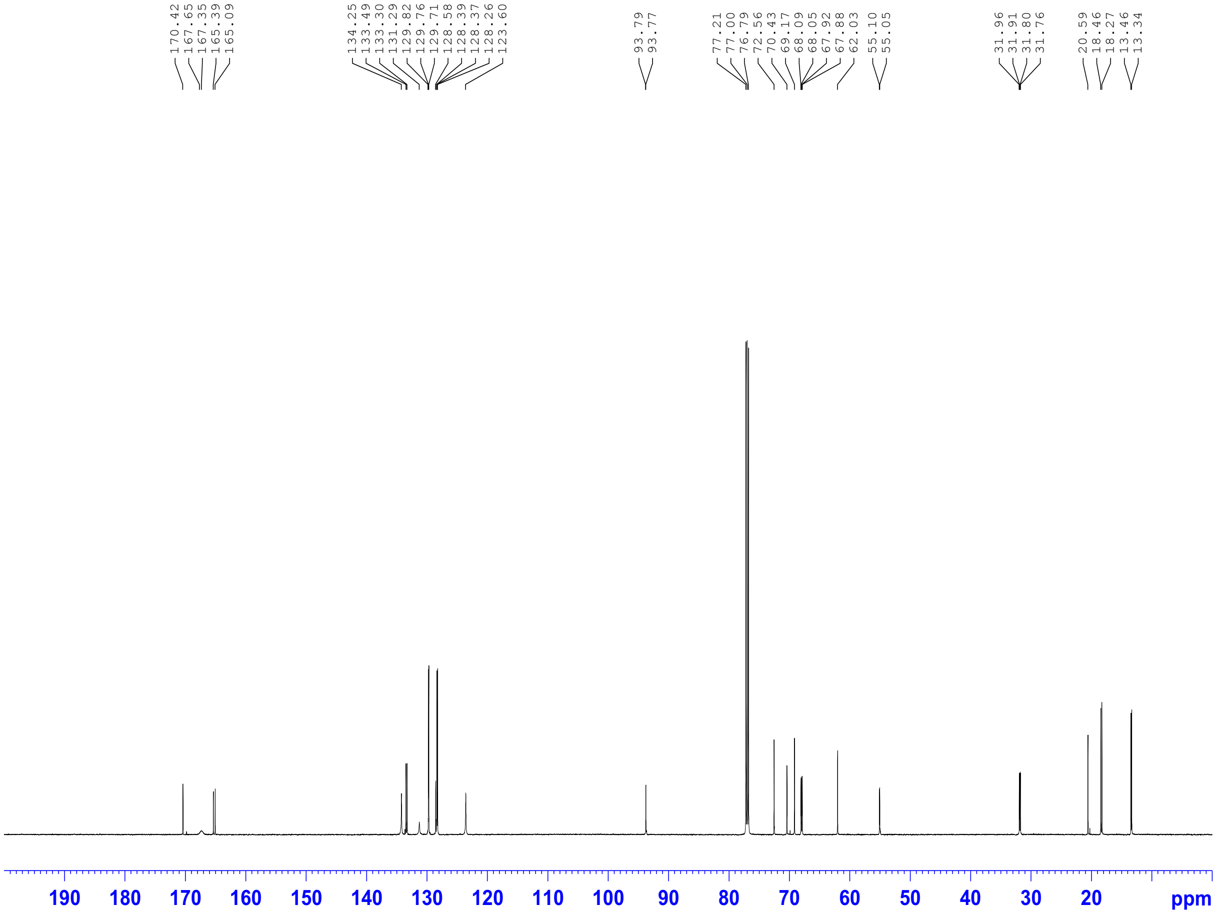

Figure S16. ^{13}C NMR spectrum of Compound 8

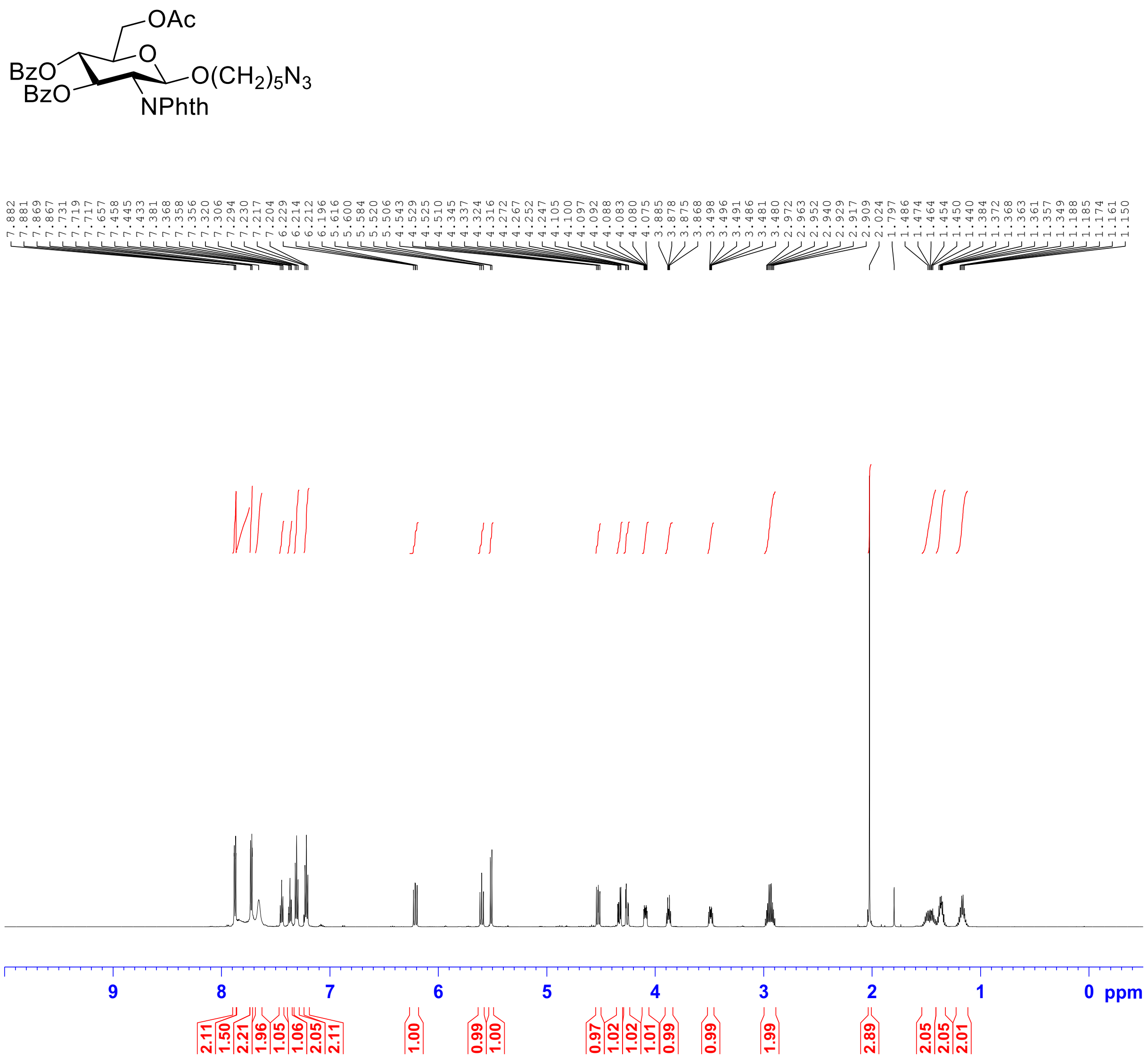

Figure S17. ^{1}H NMR spectrum of compound 8 with azido group

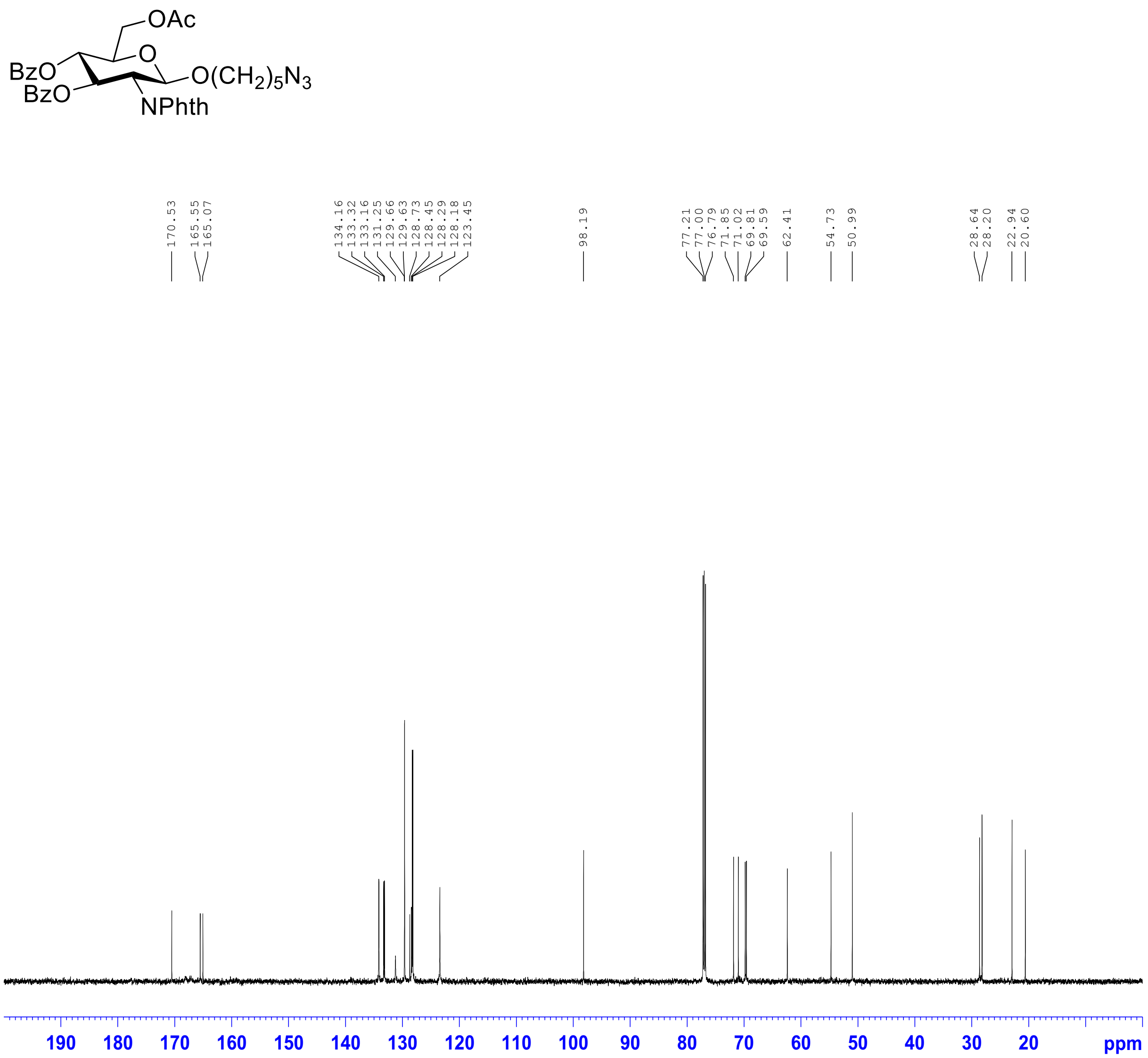

Figure S18. ^{13}C NMR spectrum of compound 8 with azido group

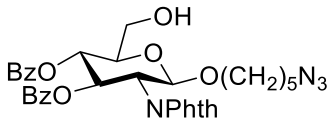

9

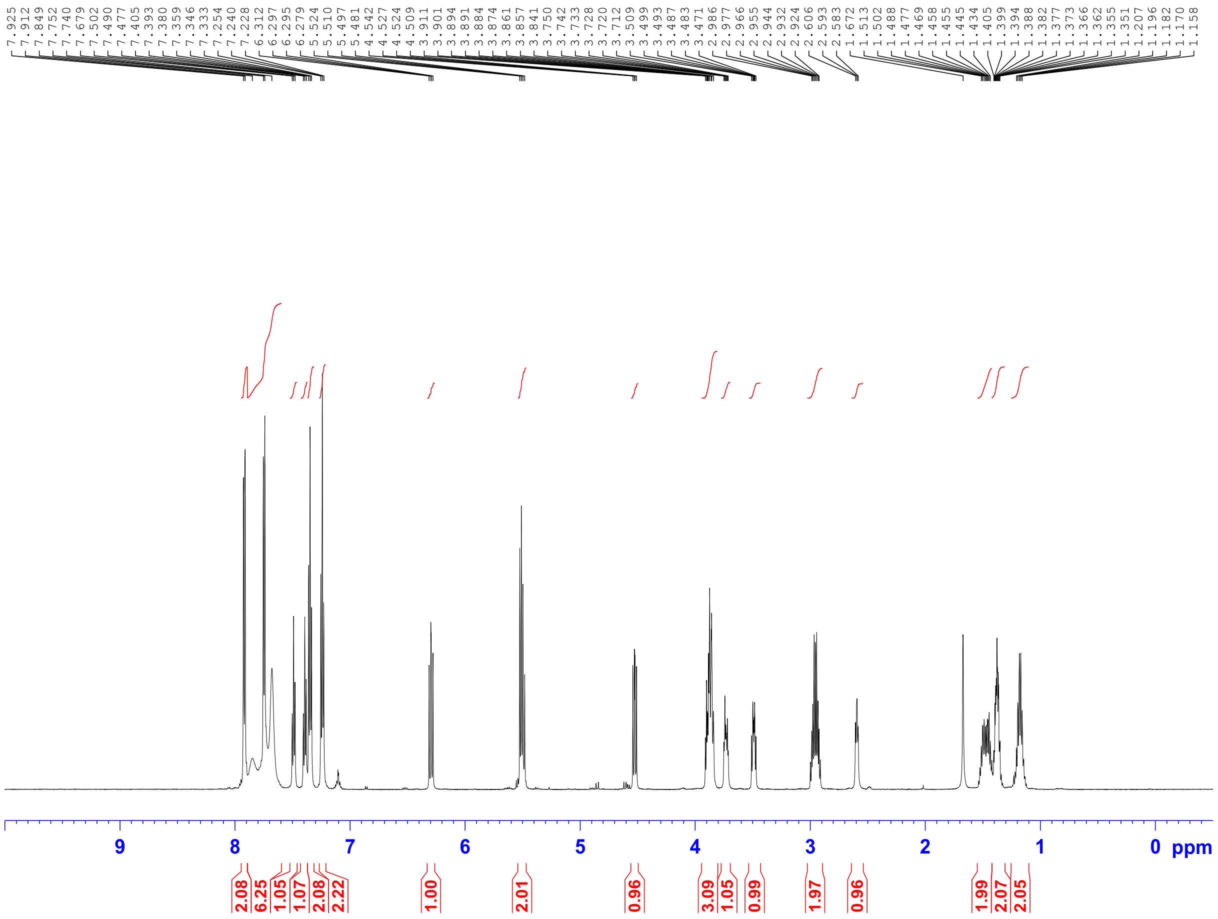

Figure S19. ^{1}**H NMR spectrum of Compound 9**

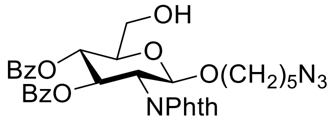

9

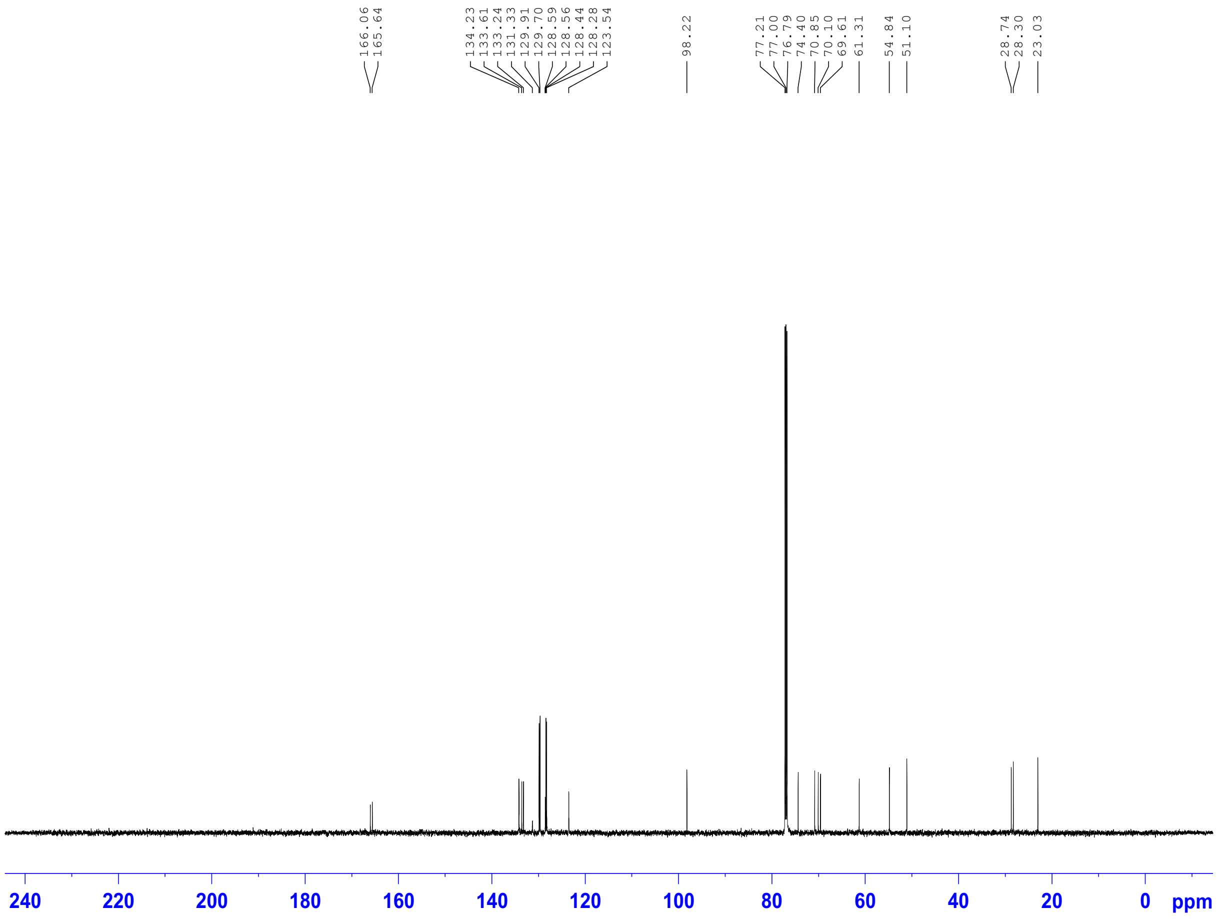

Figure S20. ^{13}C **NMR spectrum of Compound 9**

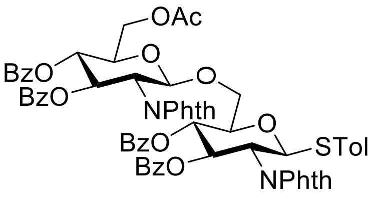

10

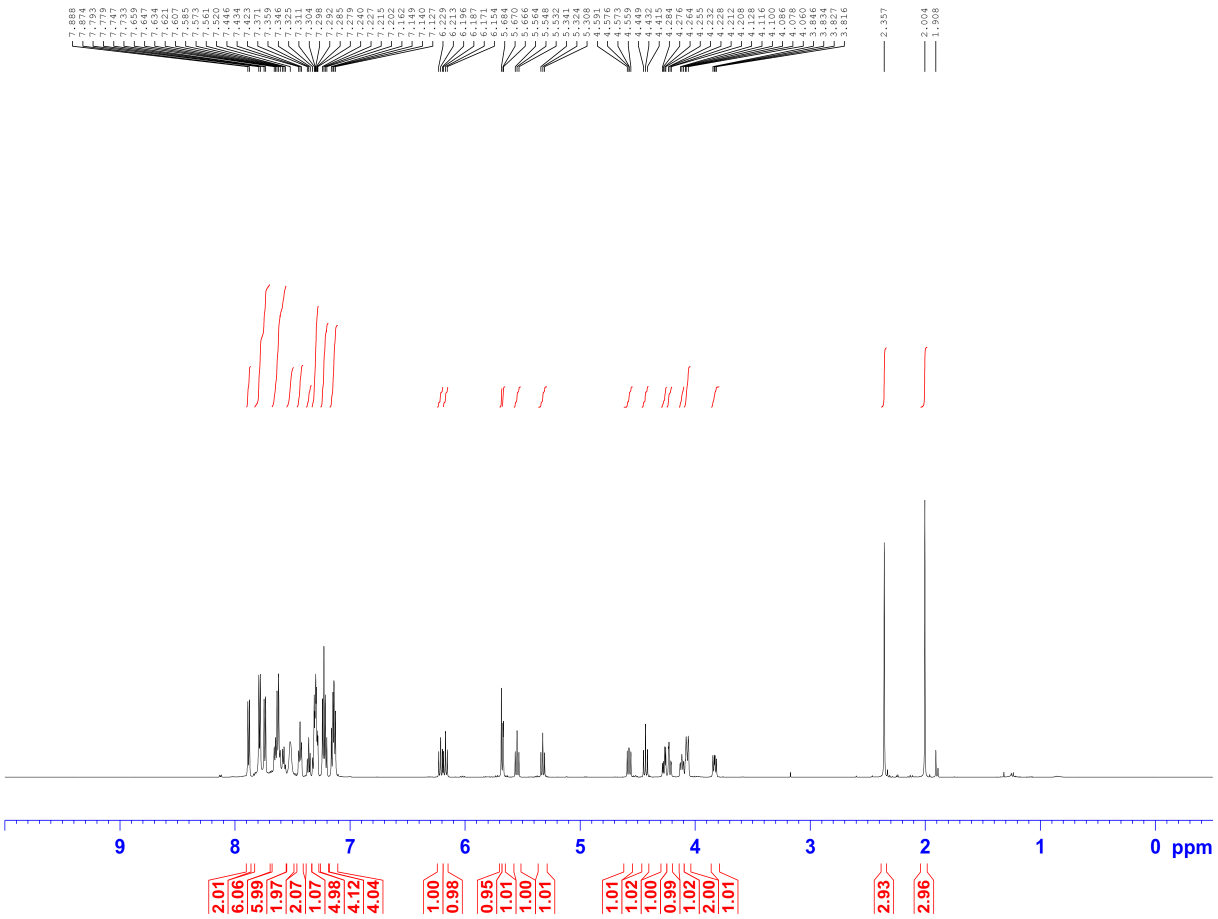

Figure S21. ^{1}H NMR spectrum of Compound 10

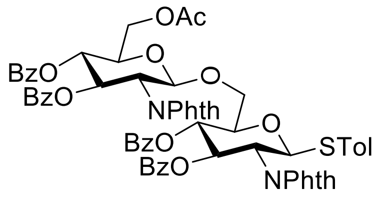

10

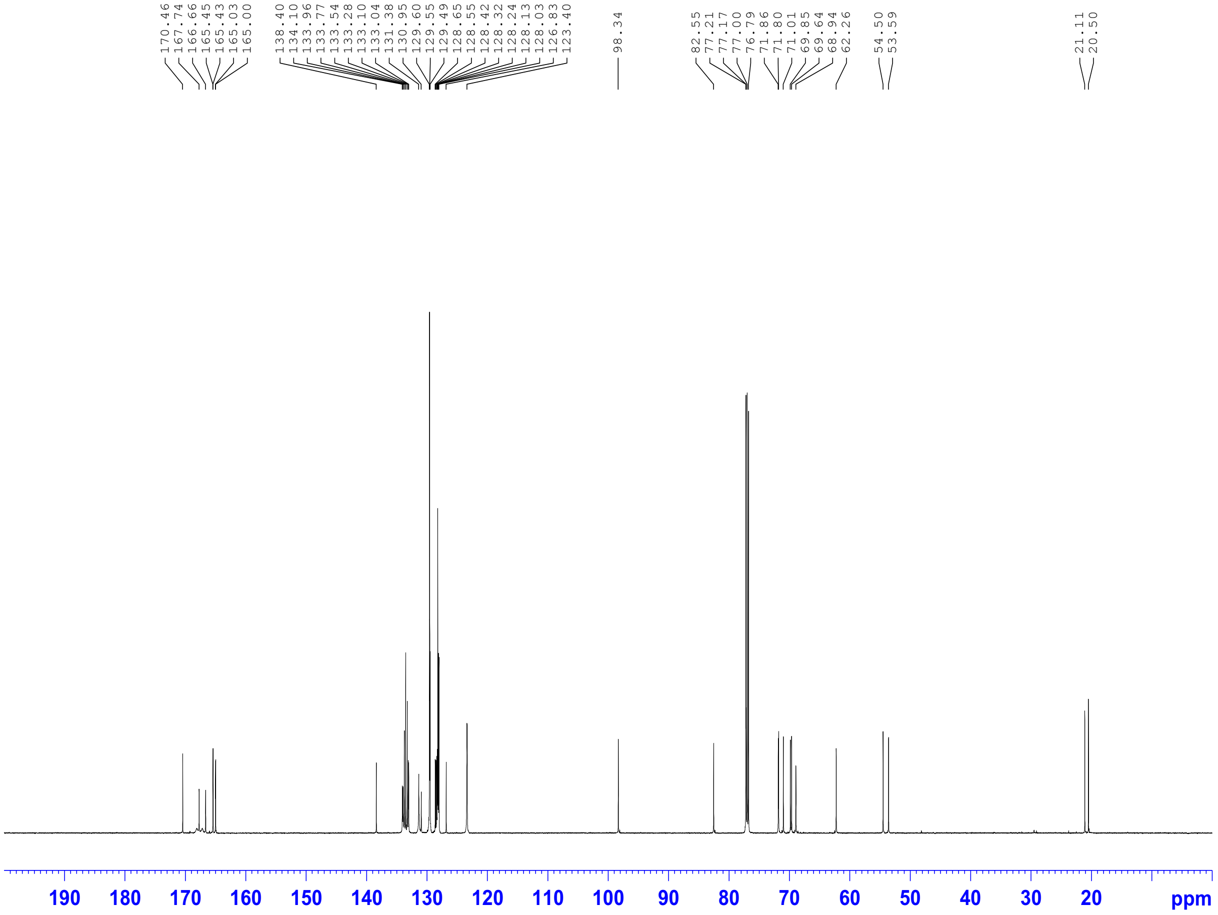

Figure S22. ^{13}C **NMR spectrum of Compound 10**

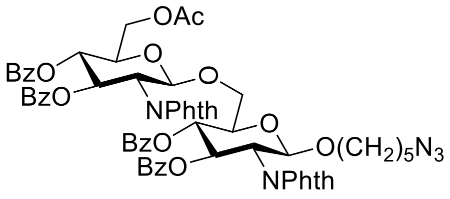

11

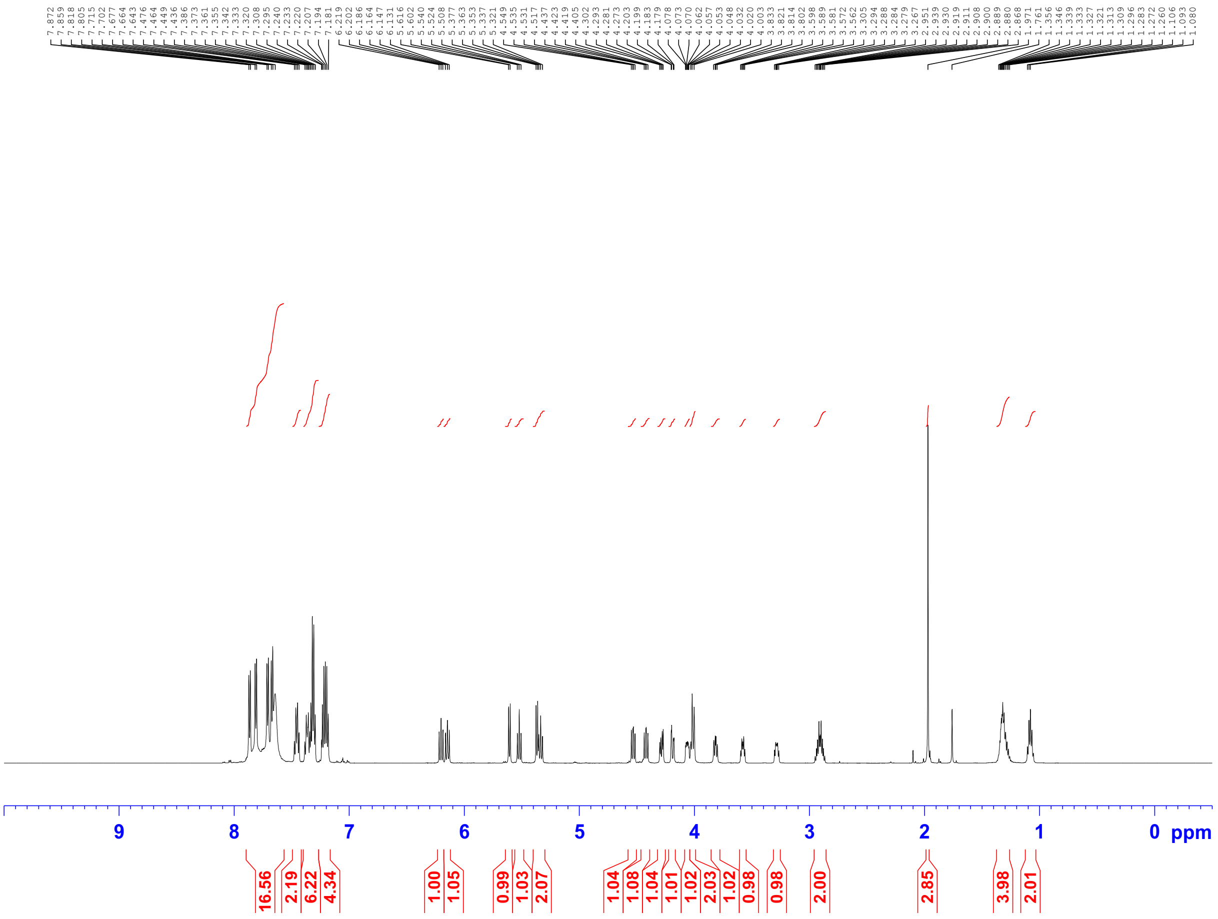

Figure S23. ^{1}H NMR spectrum of Compound 11

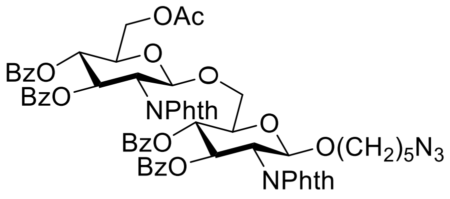

11

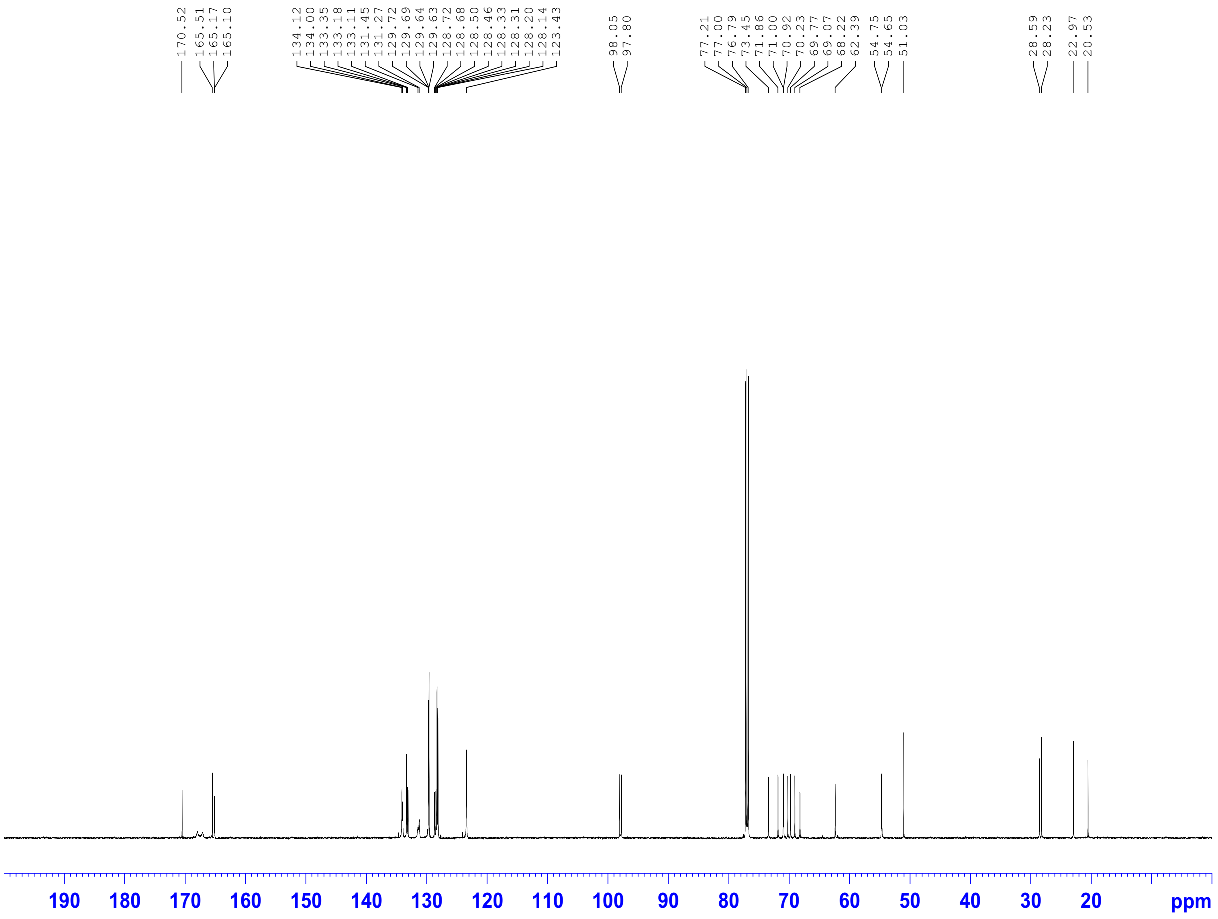

Figure S24. ^{13}C NMR spectrum of Compound 11

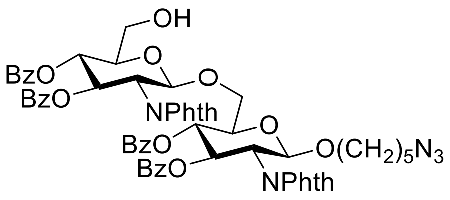

12

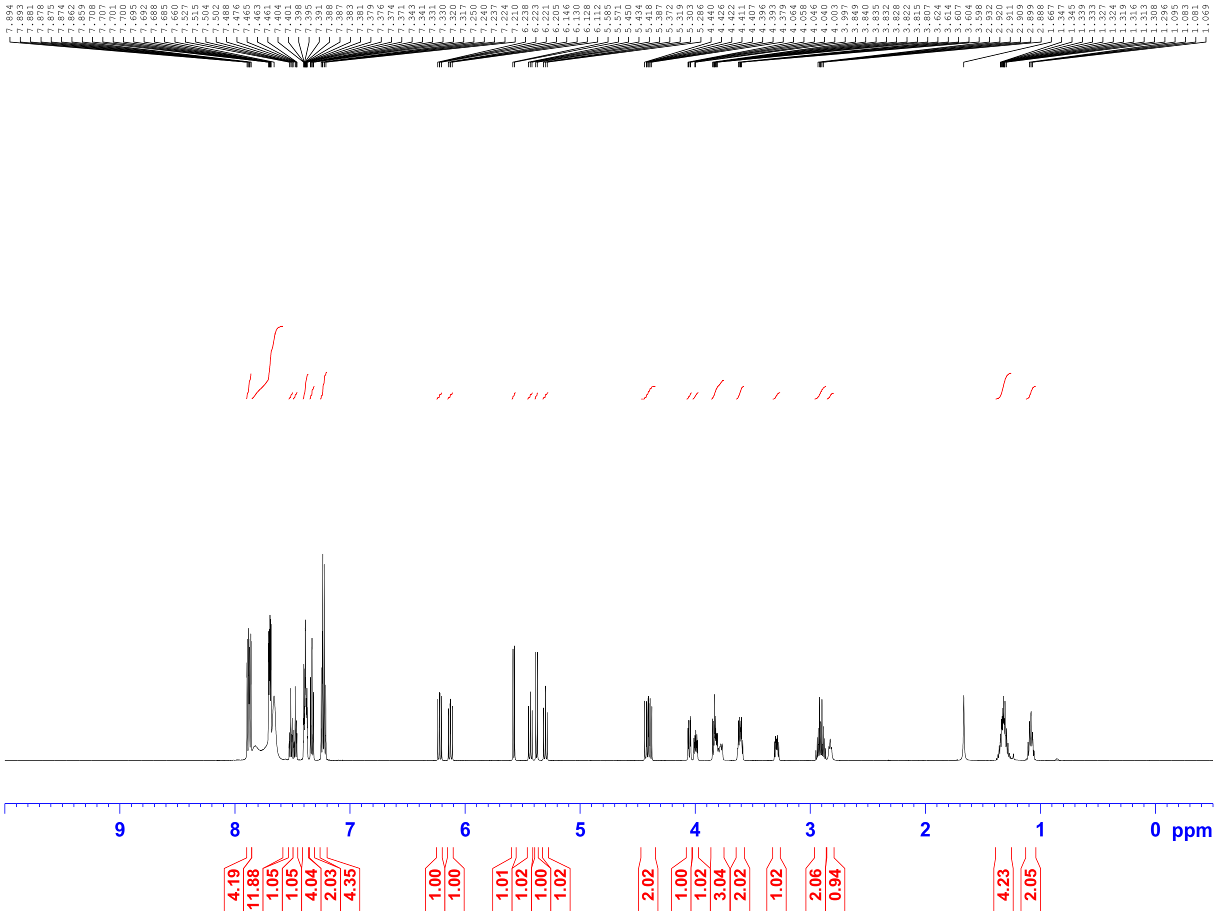

Figure S25. ^{1}H NMR spectrum of Compound 12

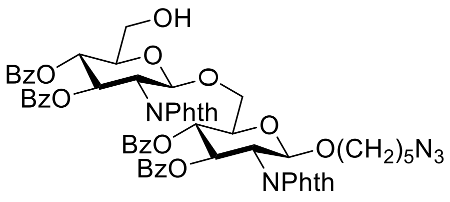

12

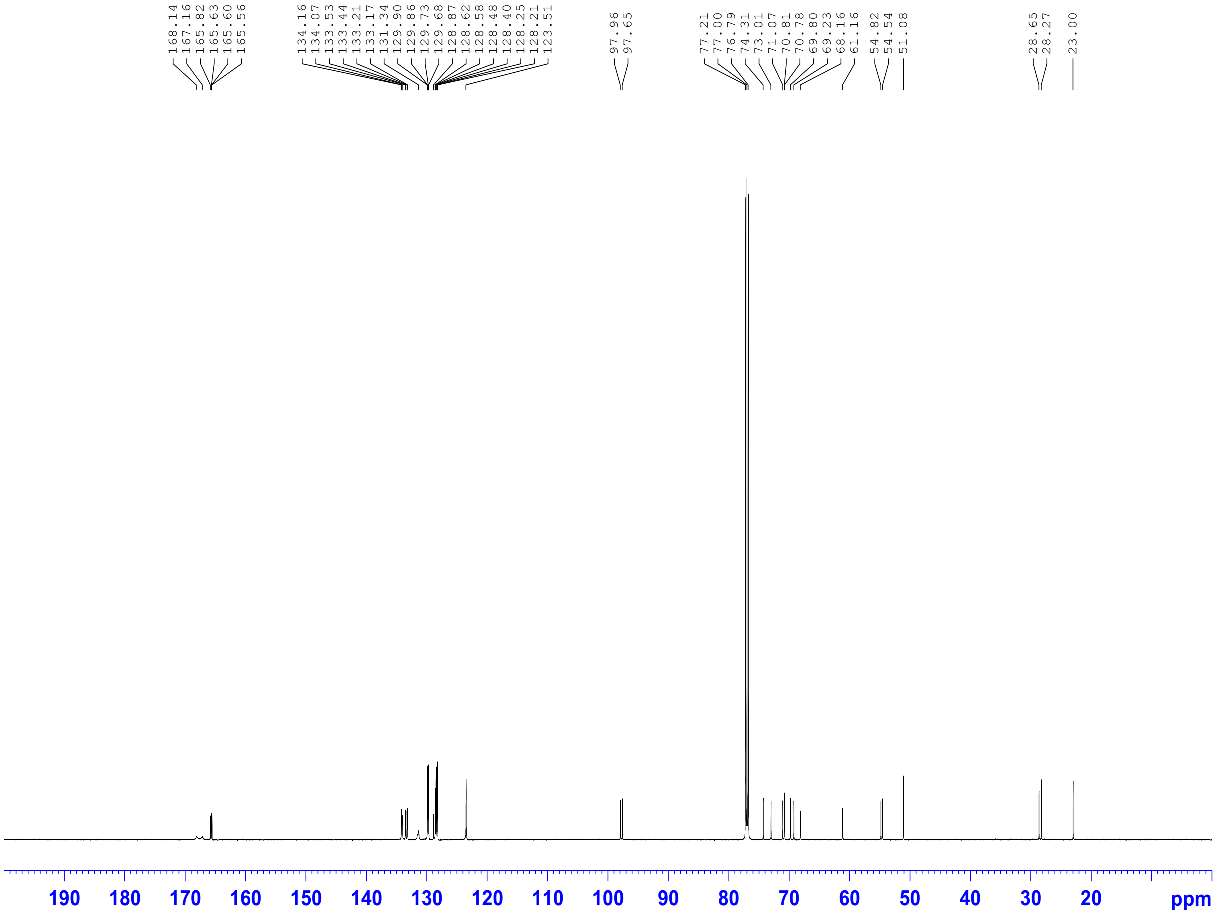

Figure S26. ^{13}**C NMR spectrum of Compound 12**

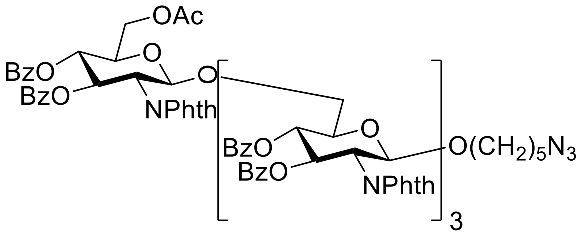

13

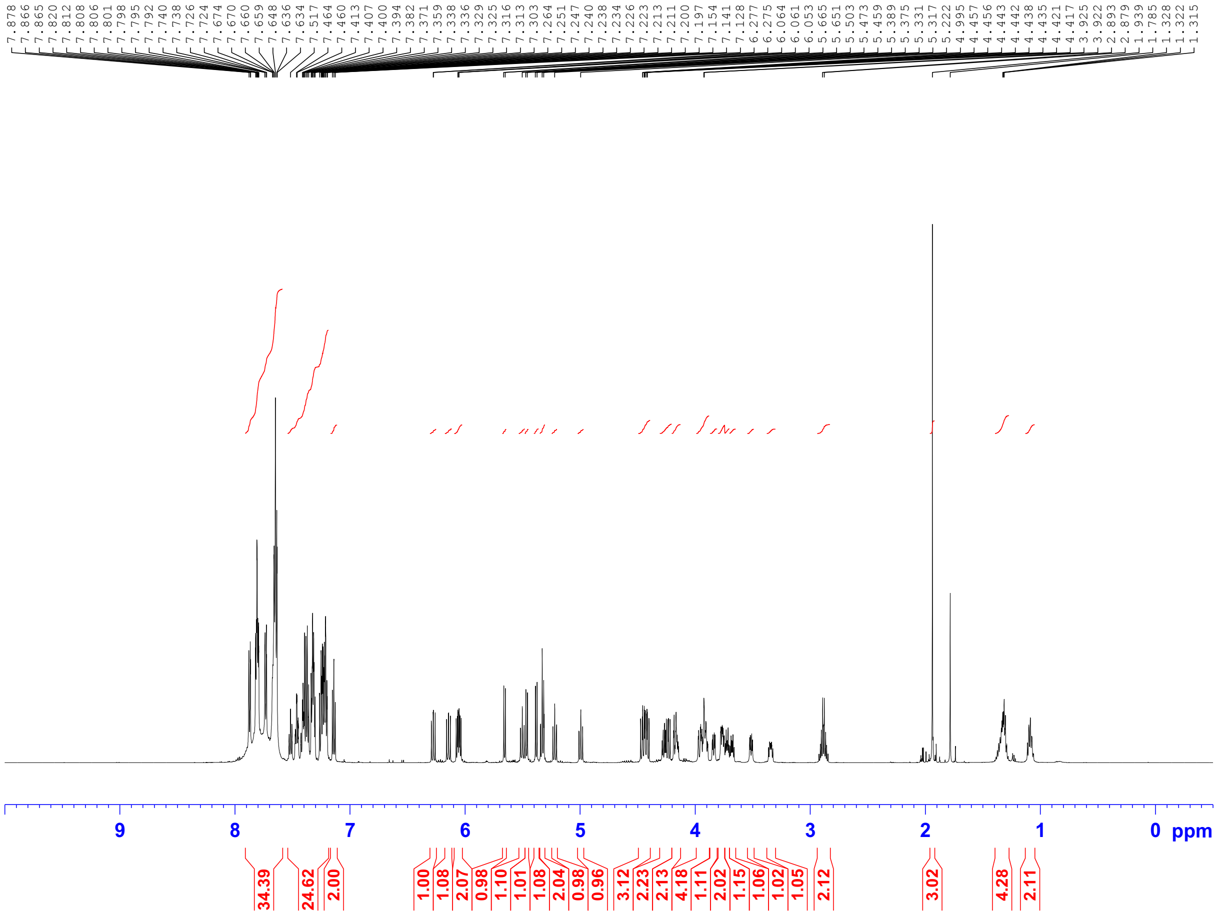

Figure S27. ^{1}H NMR spectrum of Compound 13

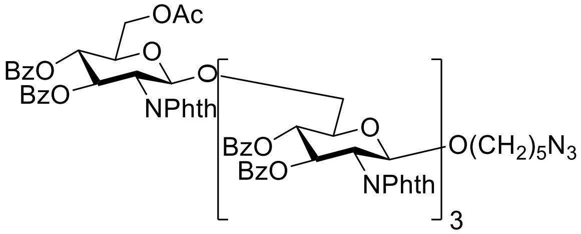

13

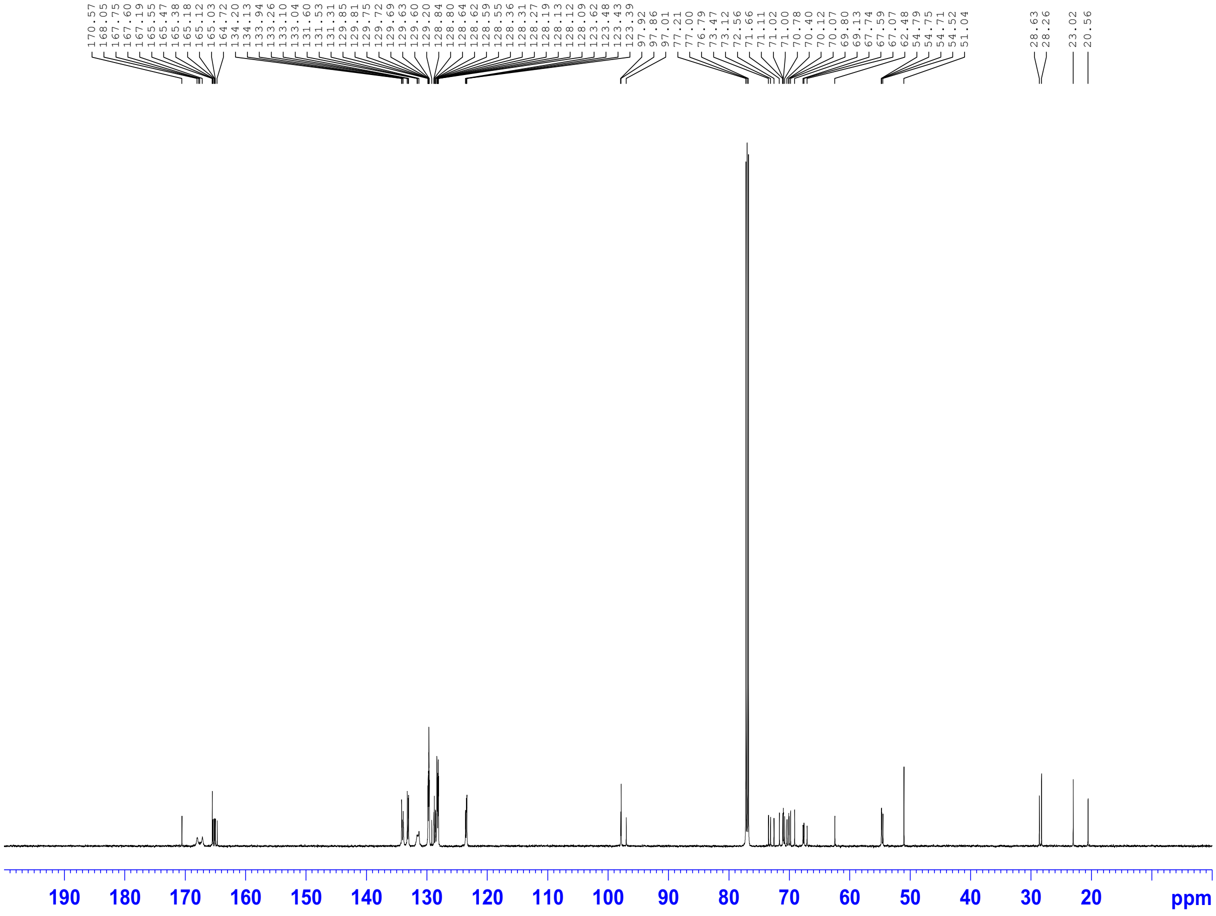

Figure S28. ^{13}**C NMR spectrum of Compound 13**

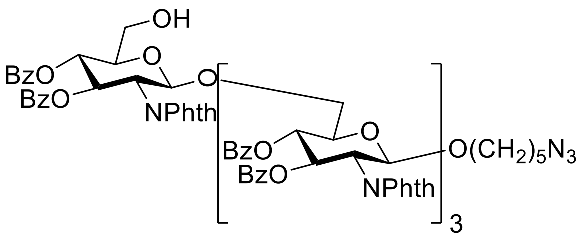

14

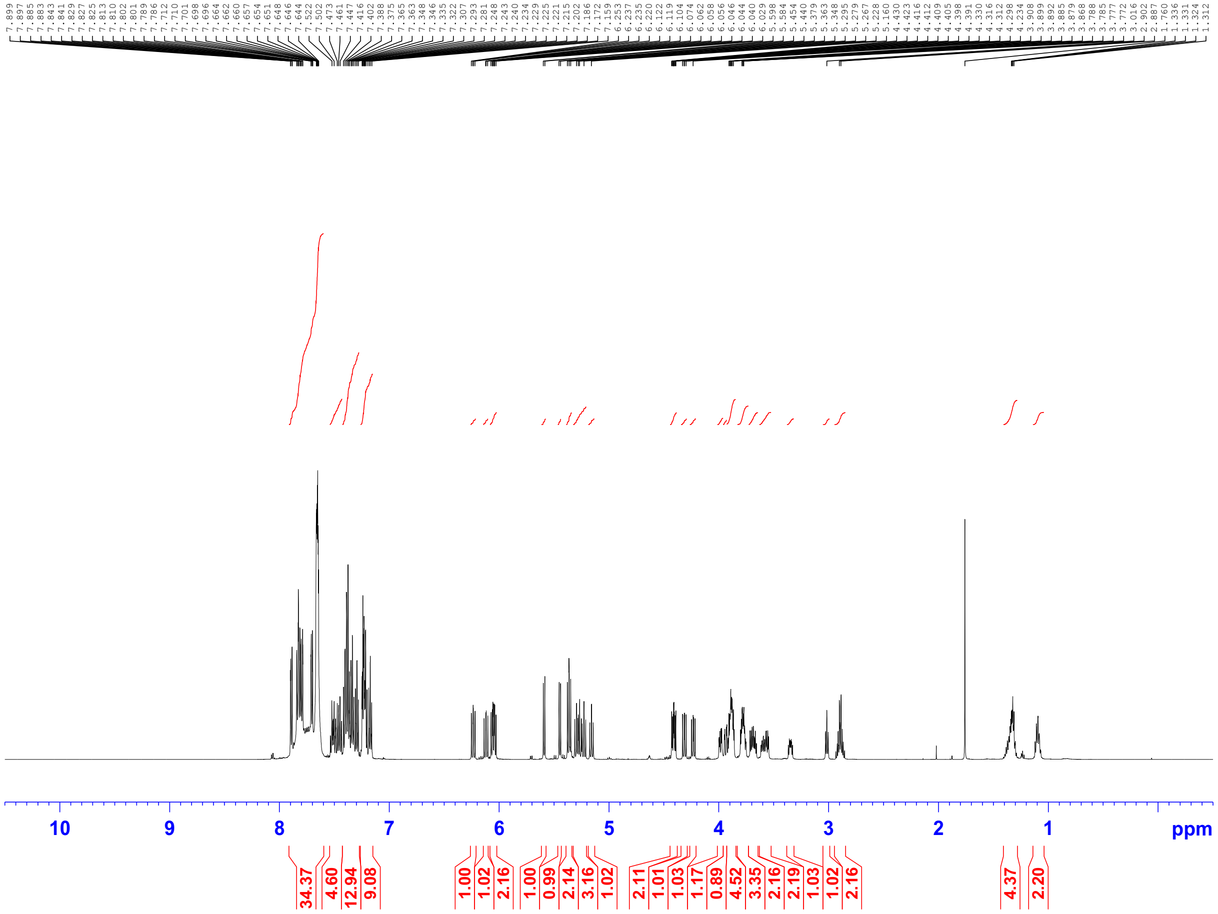

Figure S29. ^{1}H NMR spectrum of Compound 14

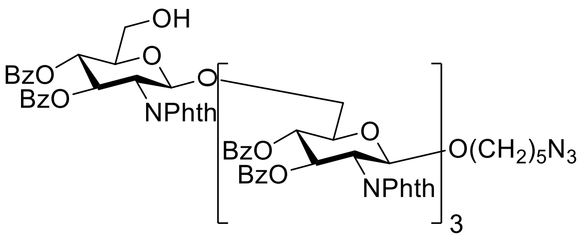

14

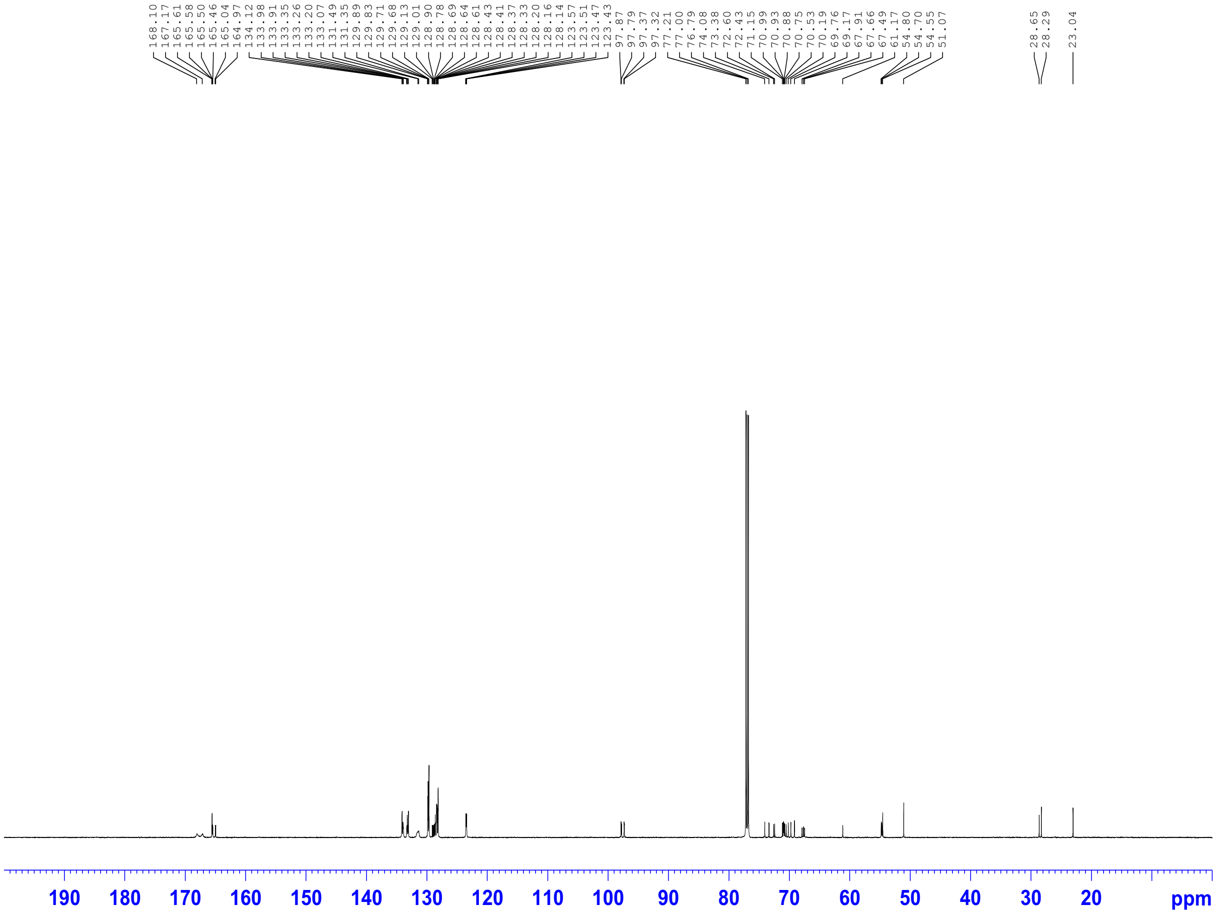

Figure S30. ^{13}C NMR spectrum of Compound 14

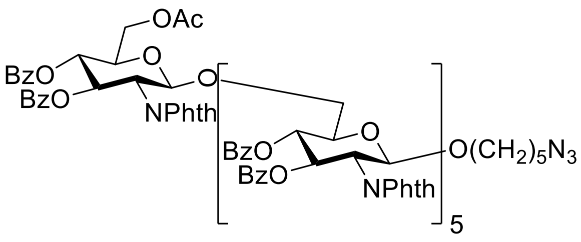

15

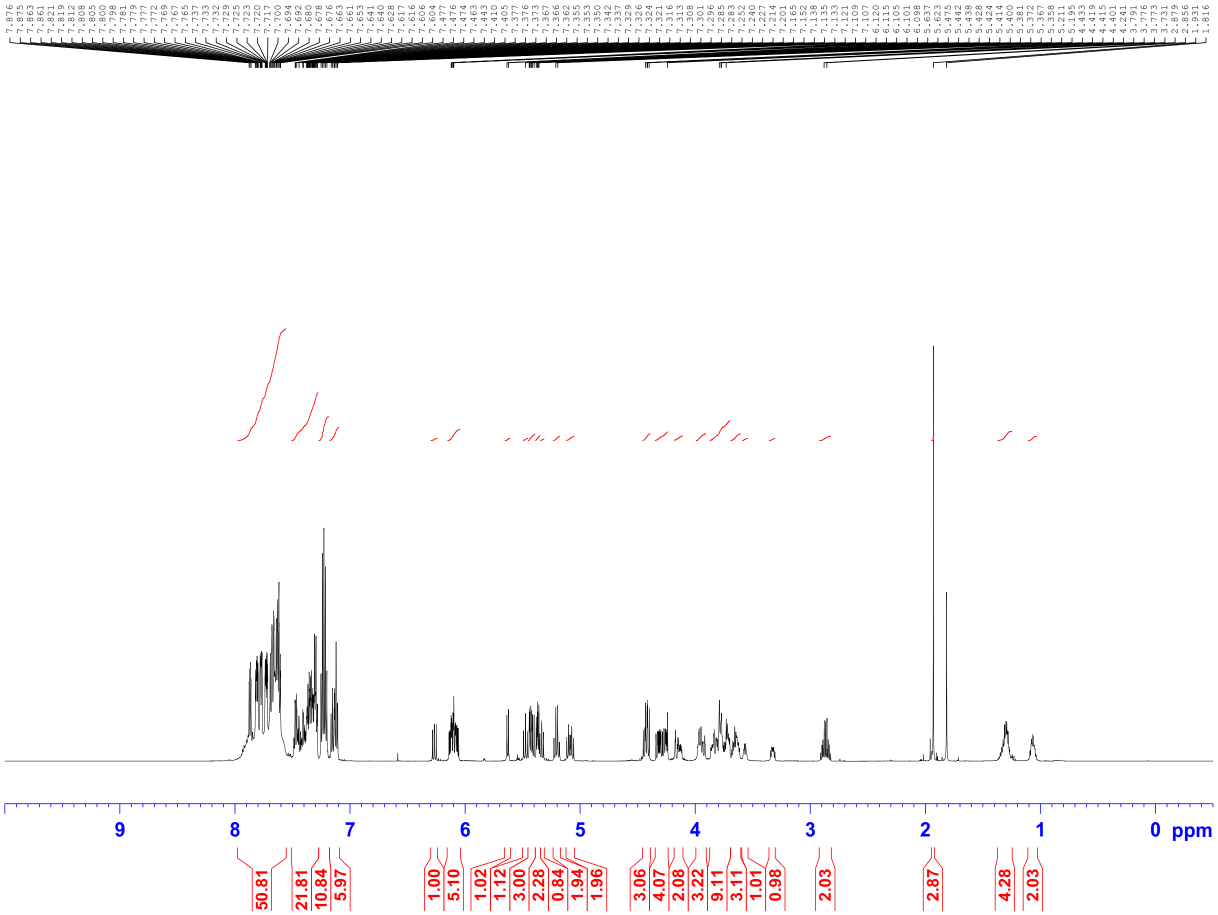

Figure S31. ^{1}H NMR spectrum of Compound 15

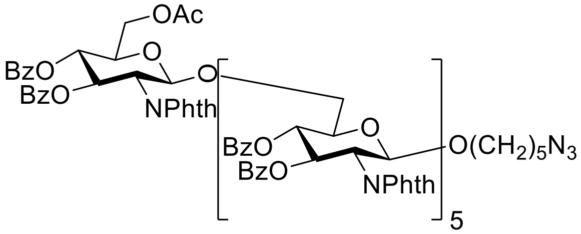

15

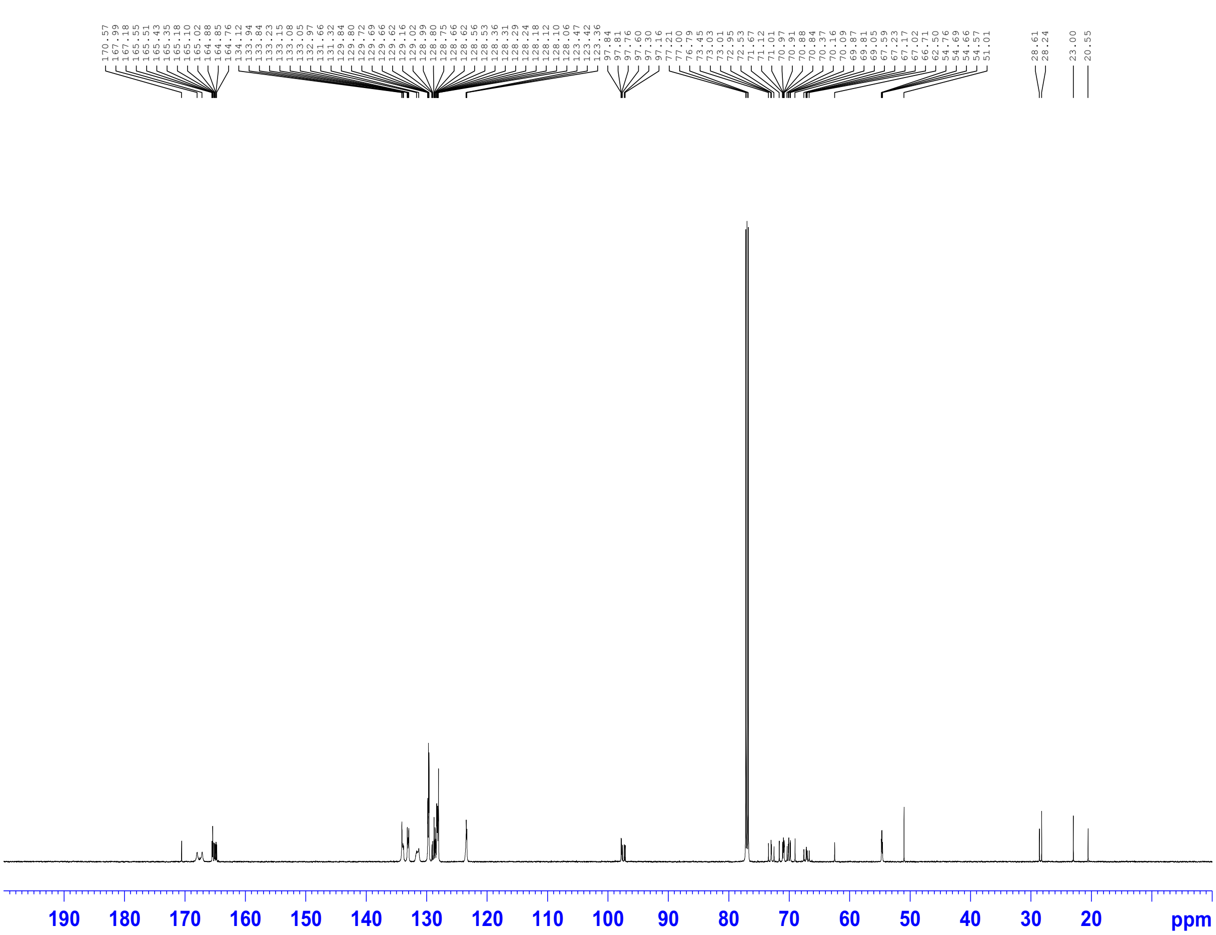

Figure S32. ^{13}C NMR spectrum of Compound 15

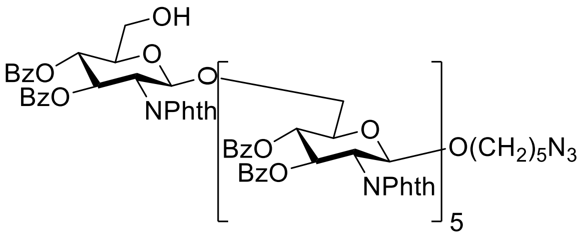

16

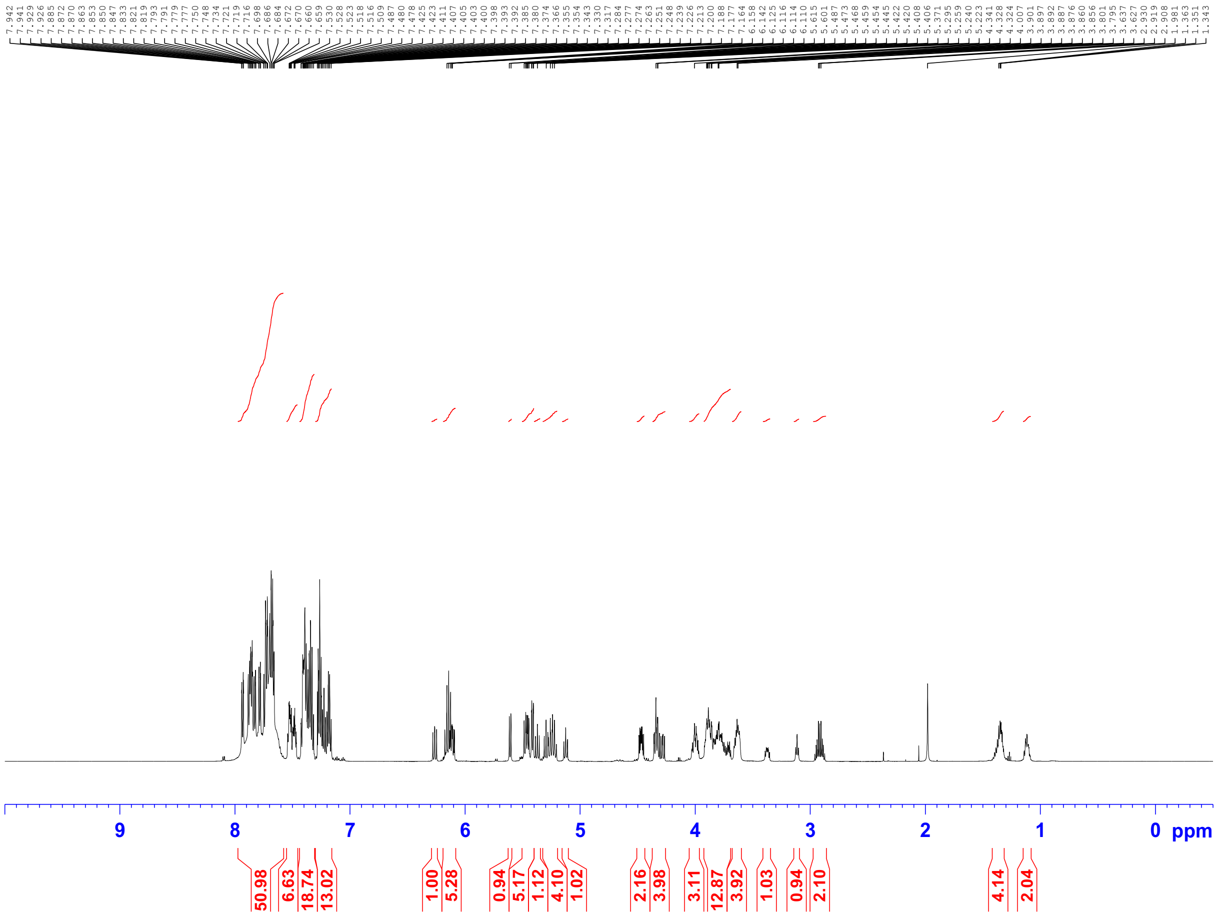

Figure S33. ^{1}H NMR spectrum of Compound 16

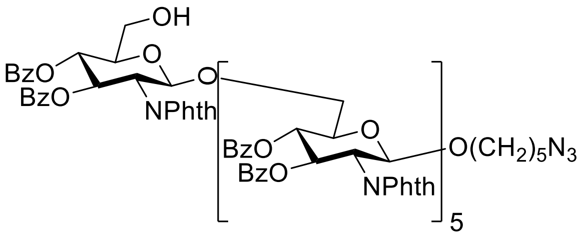

16

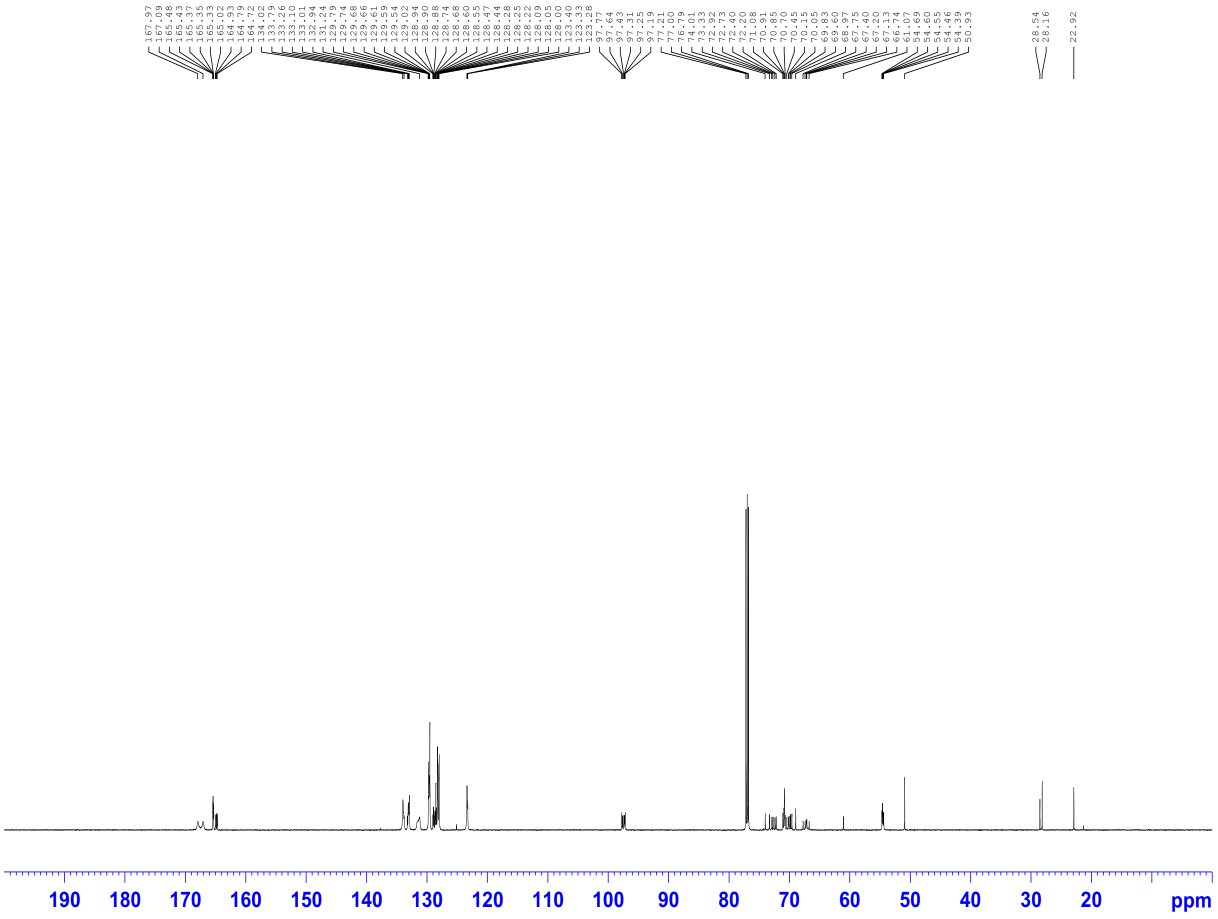

Figure S34. ^{13}C NMR spectrum of Compound 16

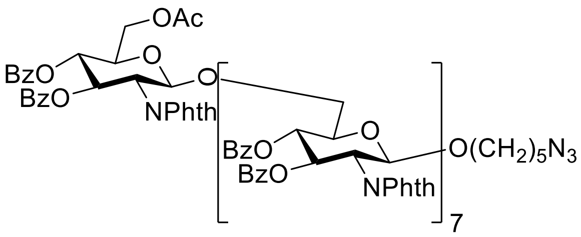

17

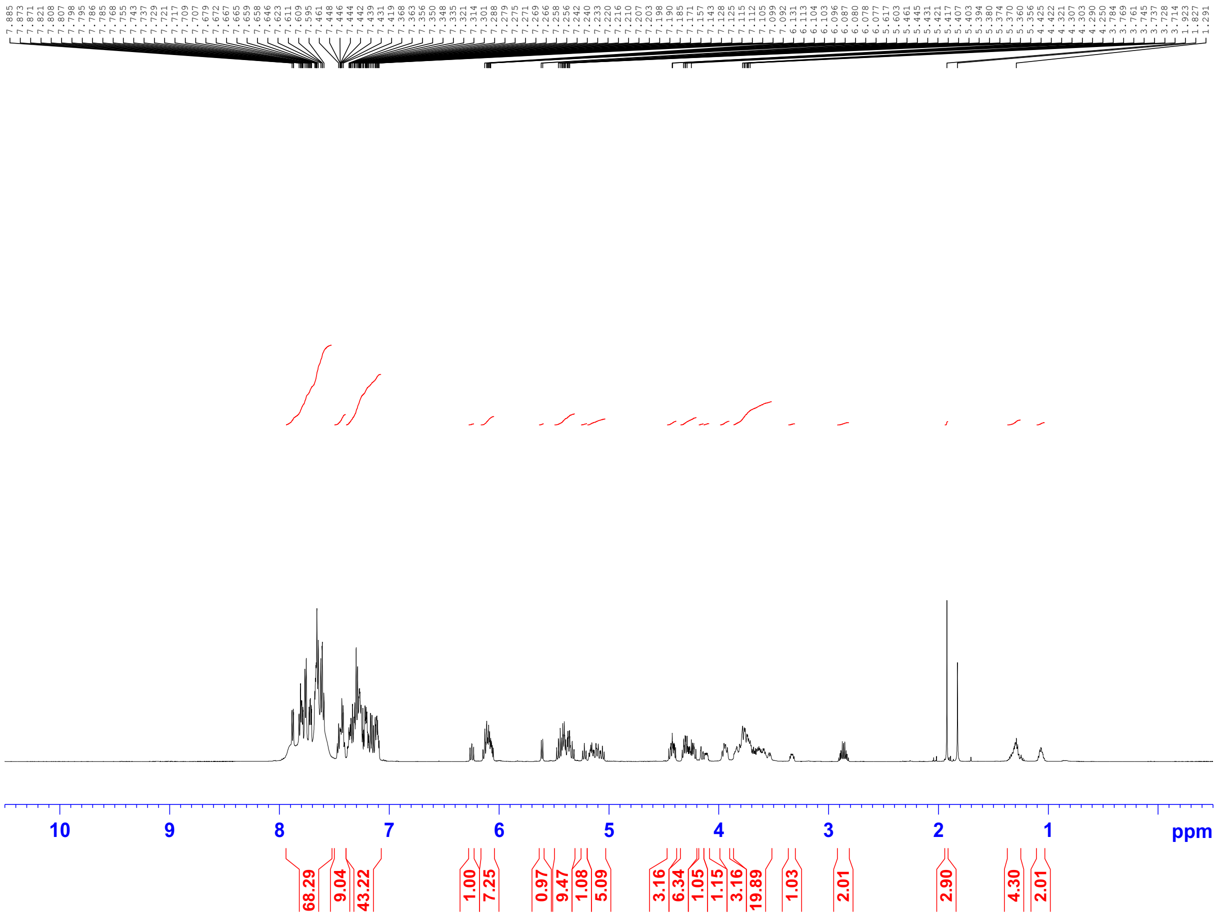

Figure S35. ^{1}H NMR spectrum of Compound 17

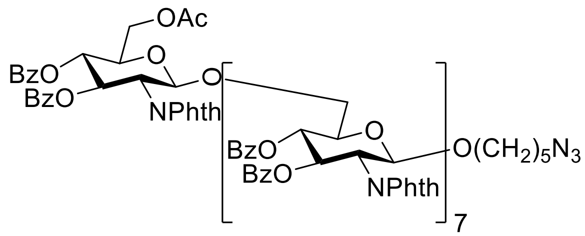

17

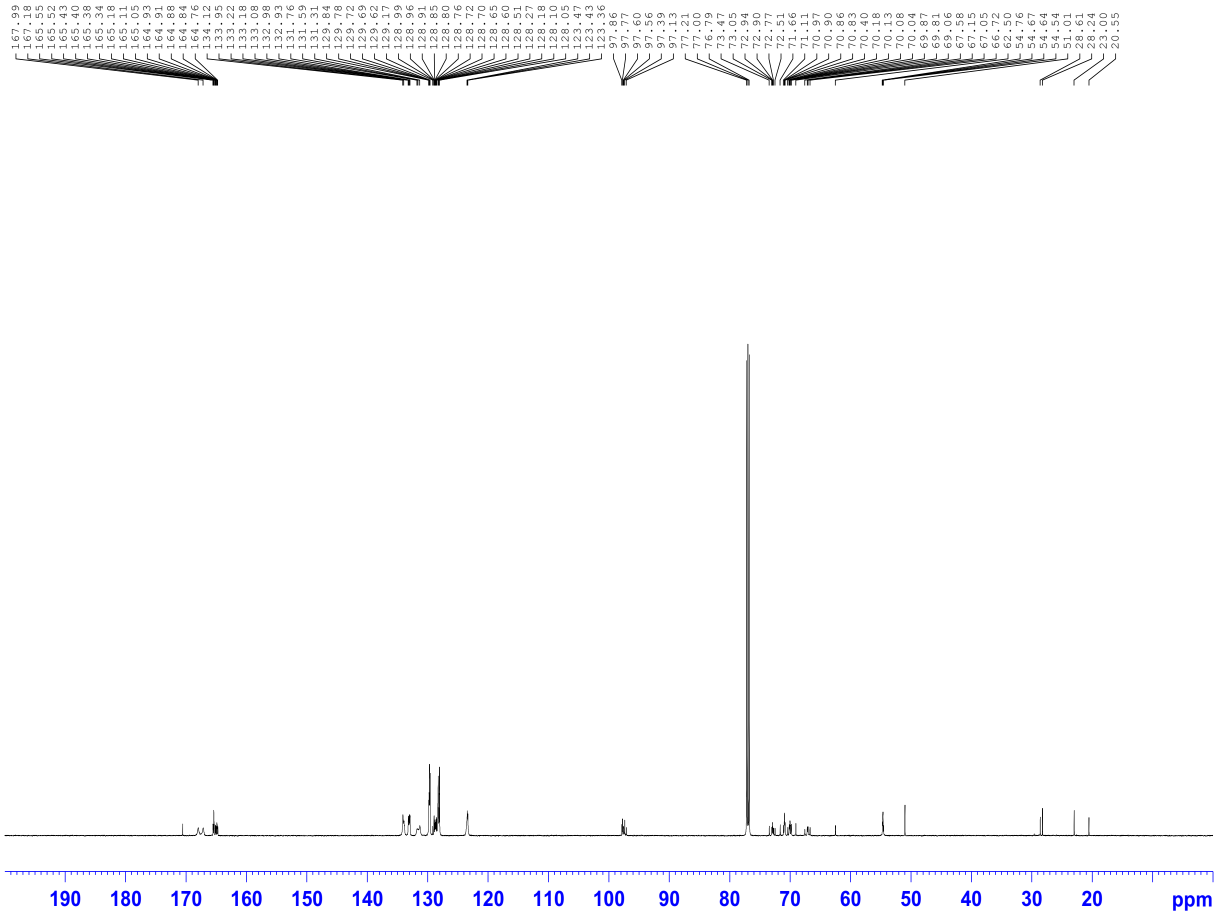

Figure S36. ^{13}C NMR spectrum of Compound 17

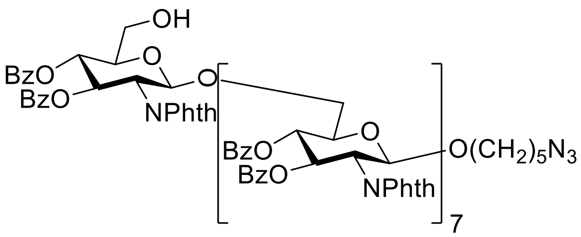

18

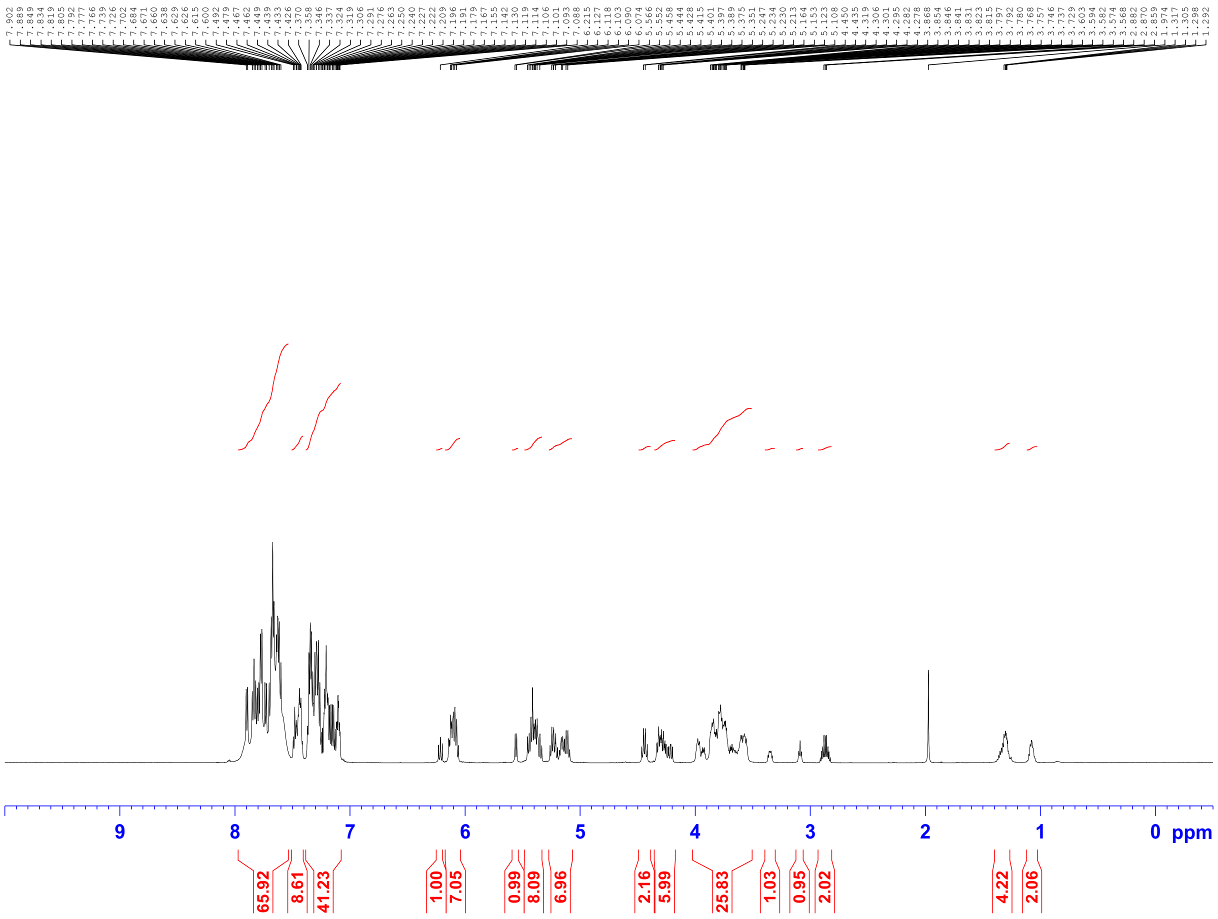

Figure S37. ^{1}H NMR spectrum of Compound 18

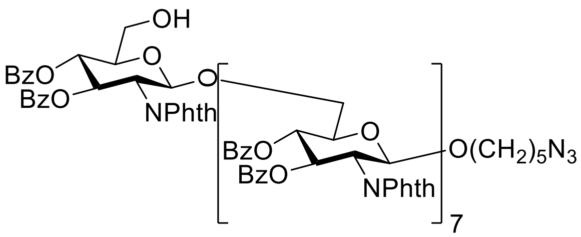

18

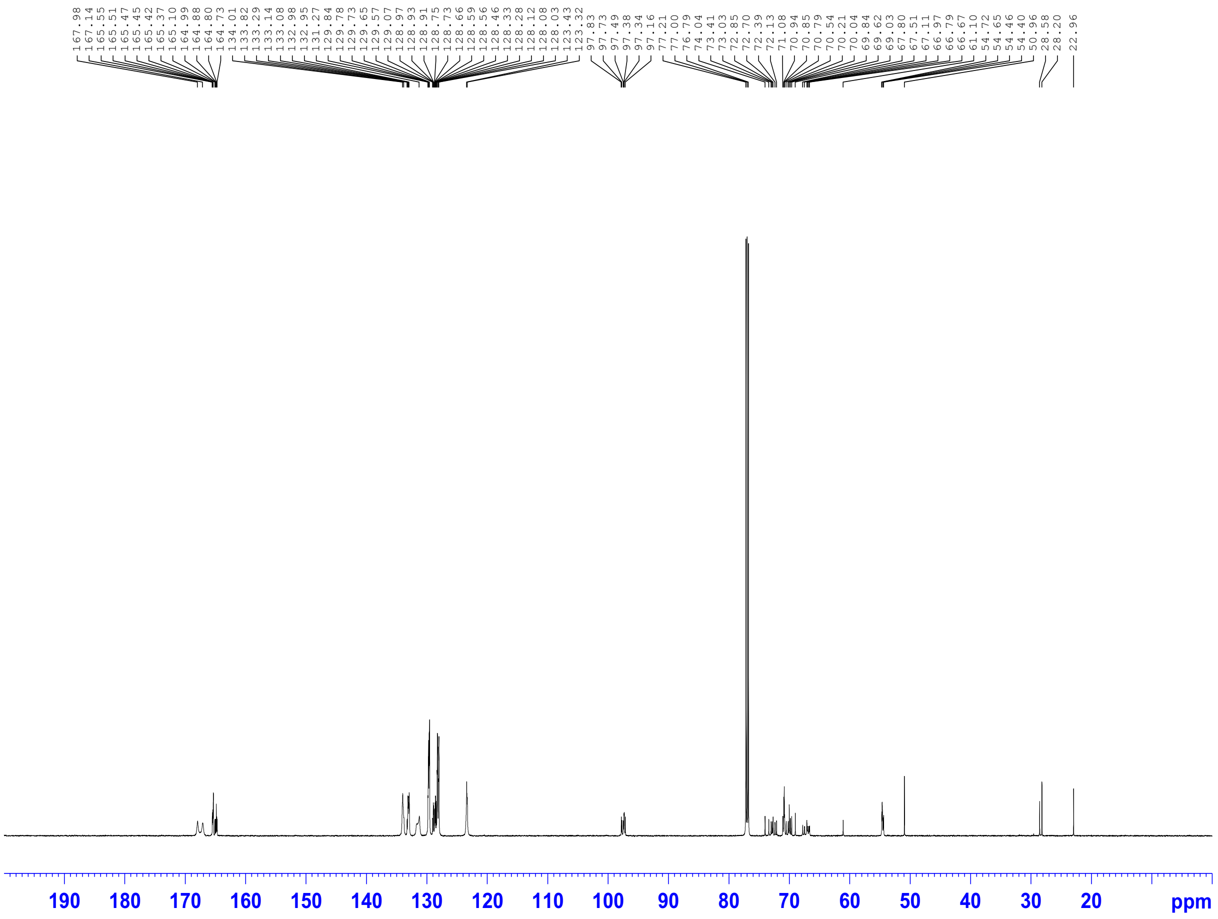

Figure S38. ^{13}C NMR spectrum of Compound 18

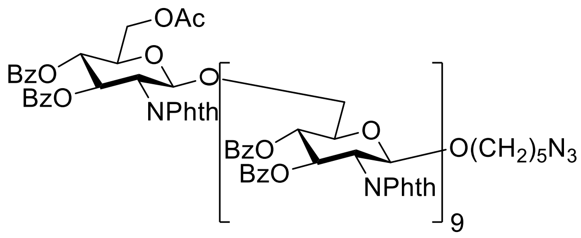

19

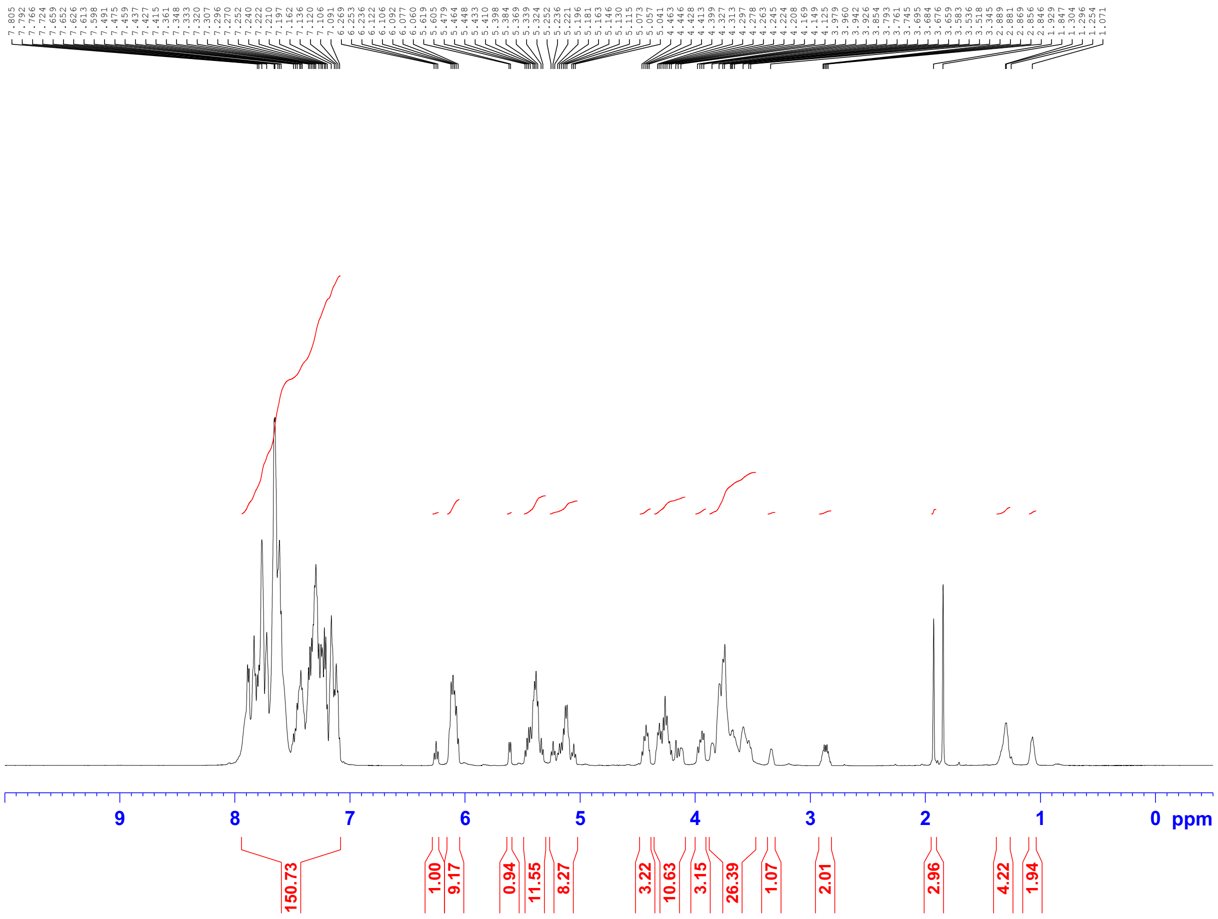

Figure S39. ^{1}H NMR spectrum of Compound 19

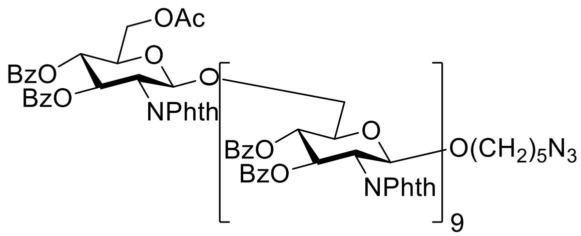

19

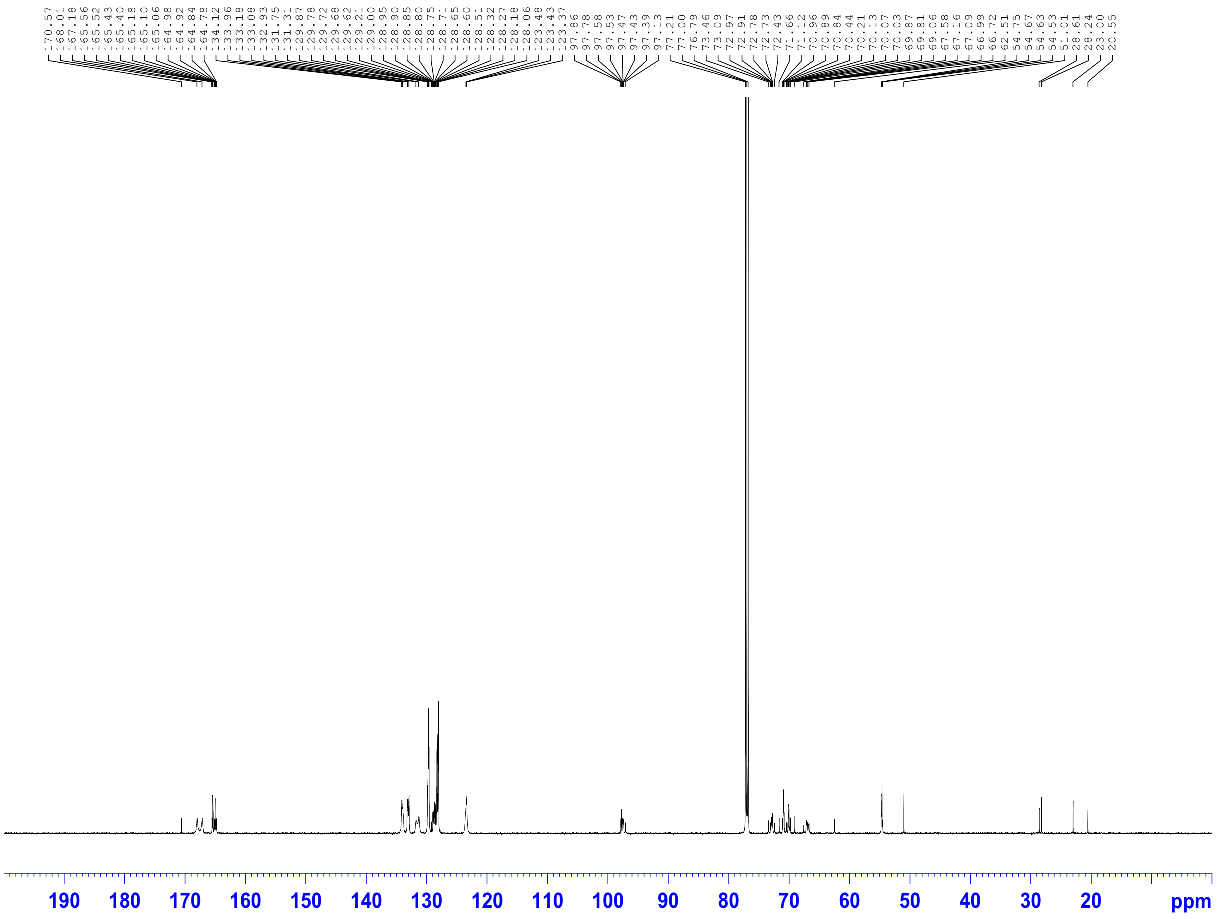

Figure S40. ^{13}C NMR spectrum of Compound 19

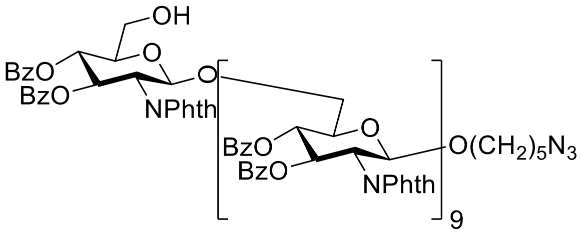

20

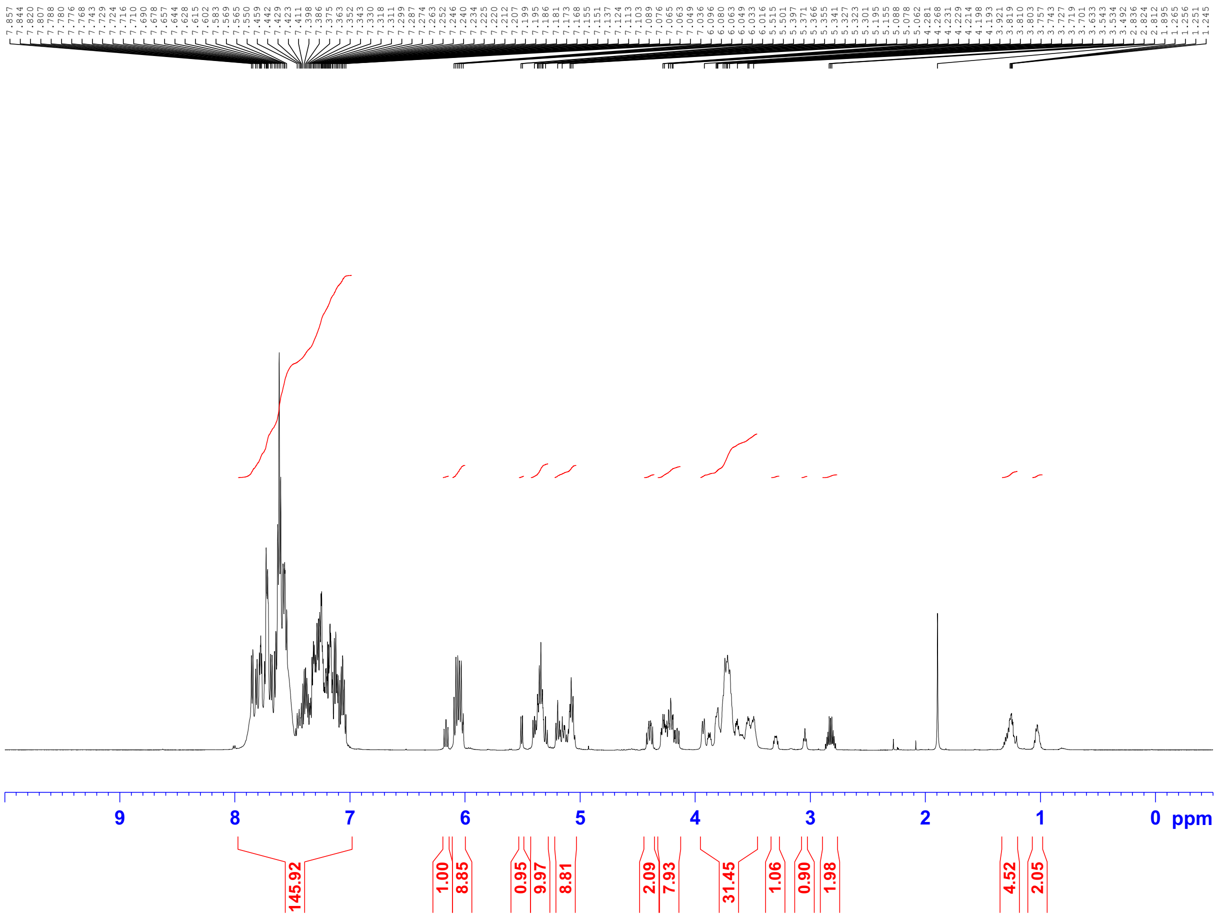

Figure S41. ^{1}**H NMR spectrum of Compound 20**

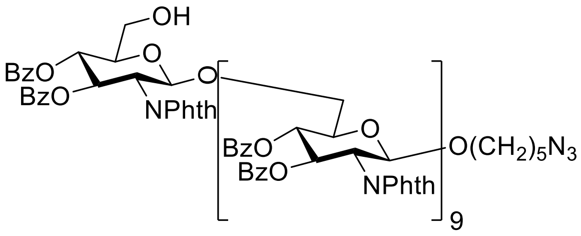

20

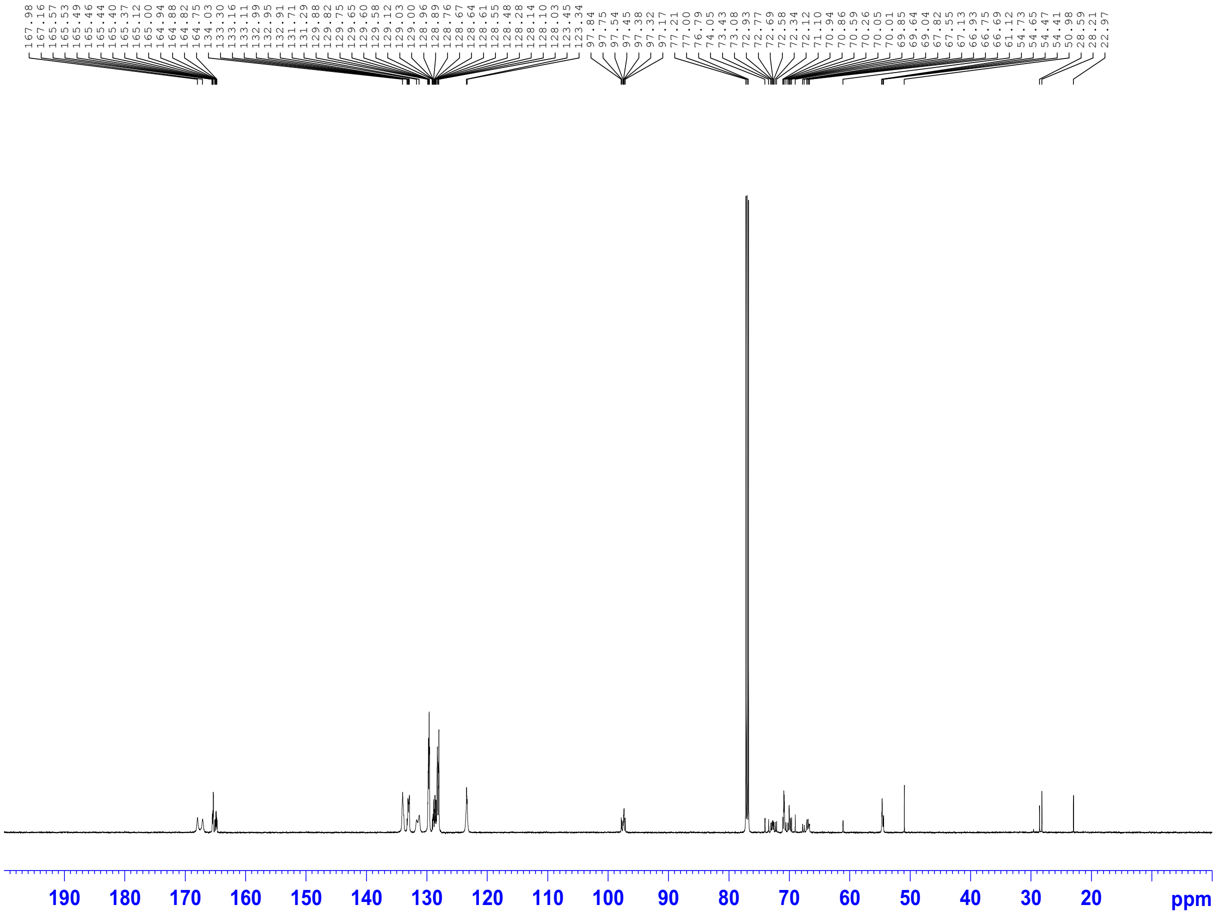

Figure S42. ^{13}C NMR spectrum of Compound 20

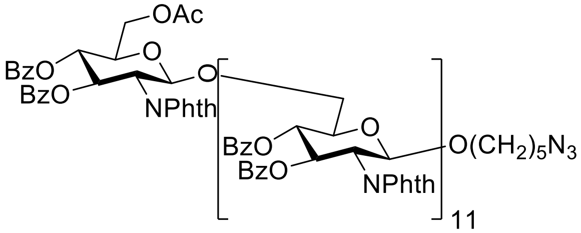

21

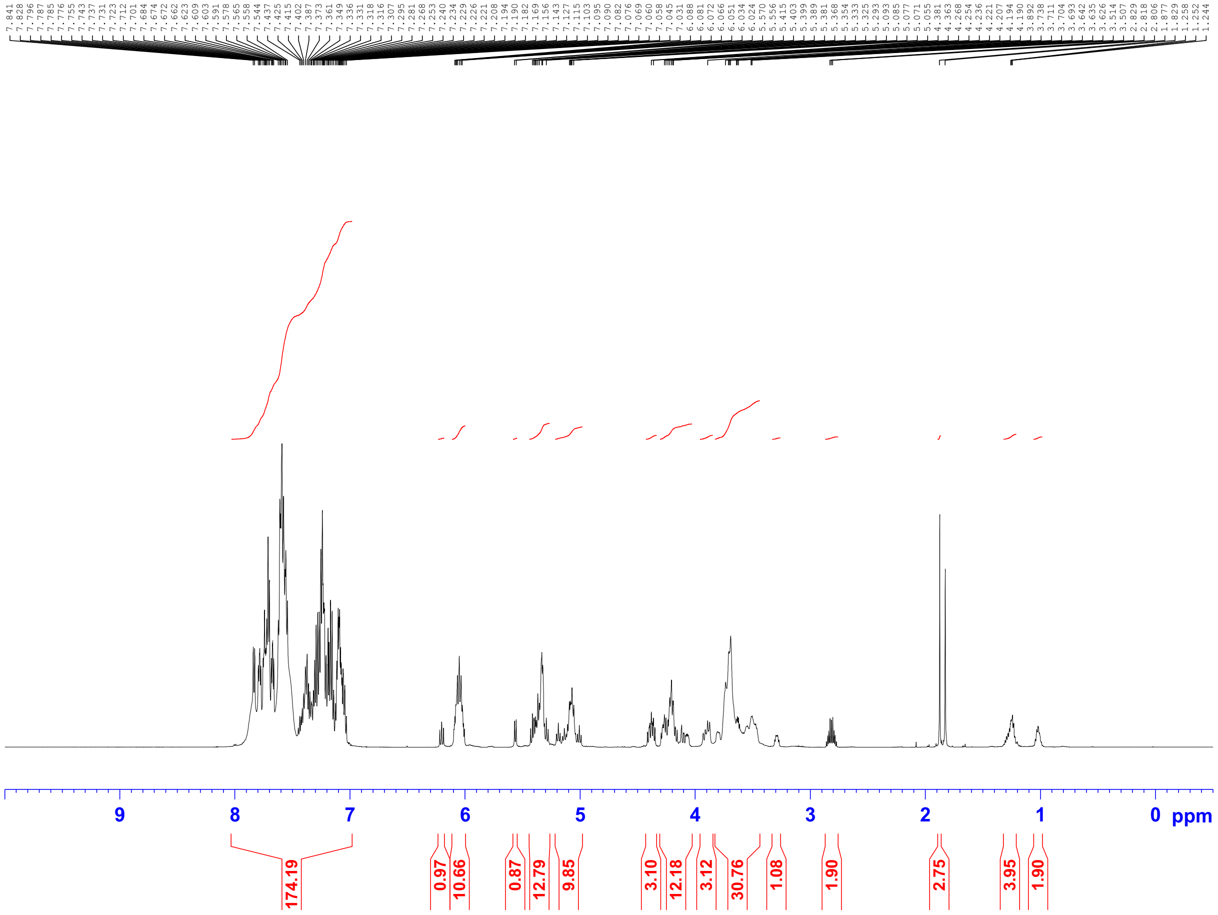

Figure S43. ^{1}H NMR spectrum of Compound 21

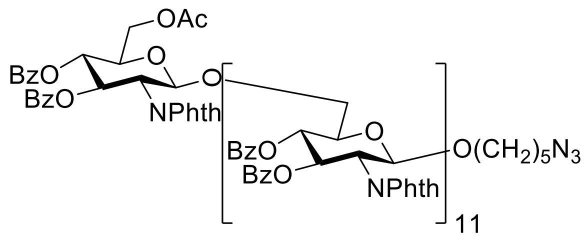

21

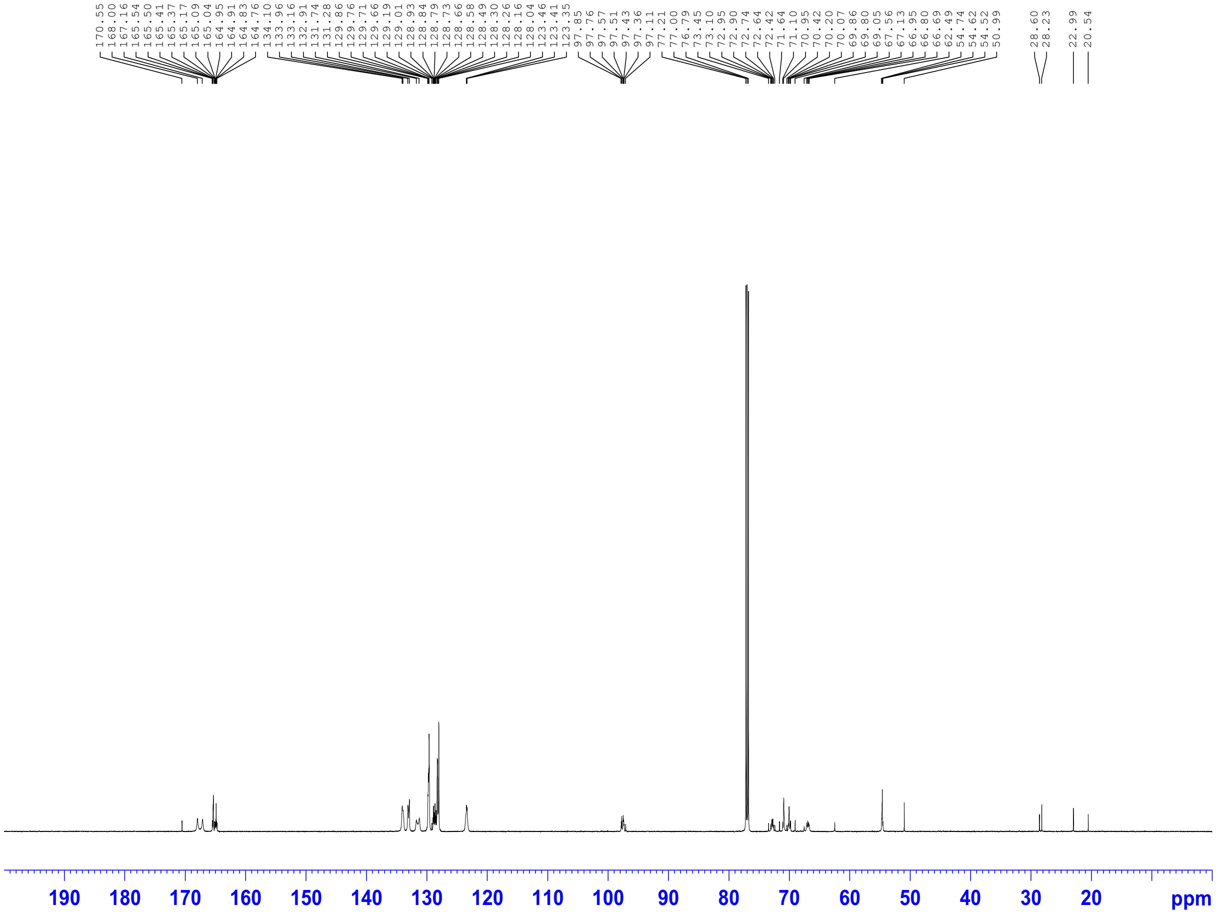

Figure S44. ^{13}C NMR spectrum of Compound 21

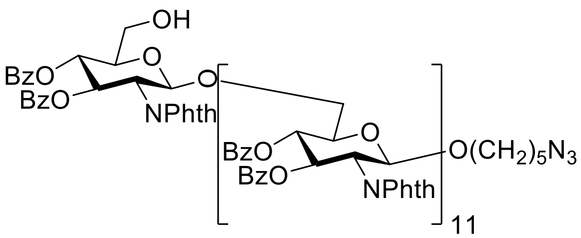

22

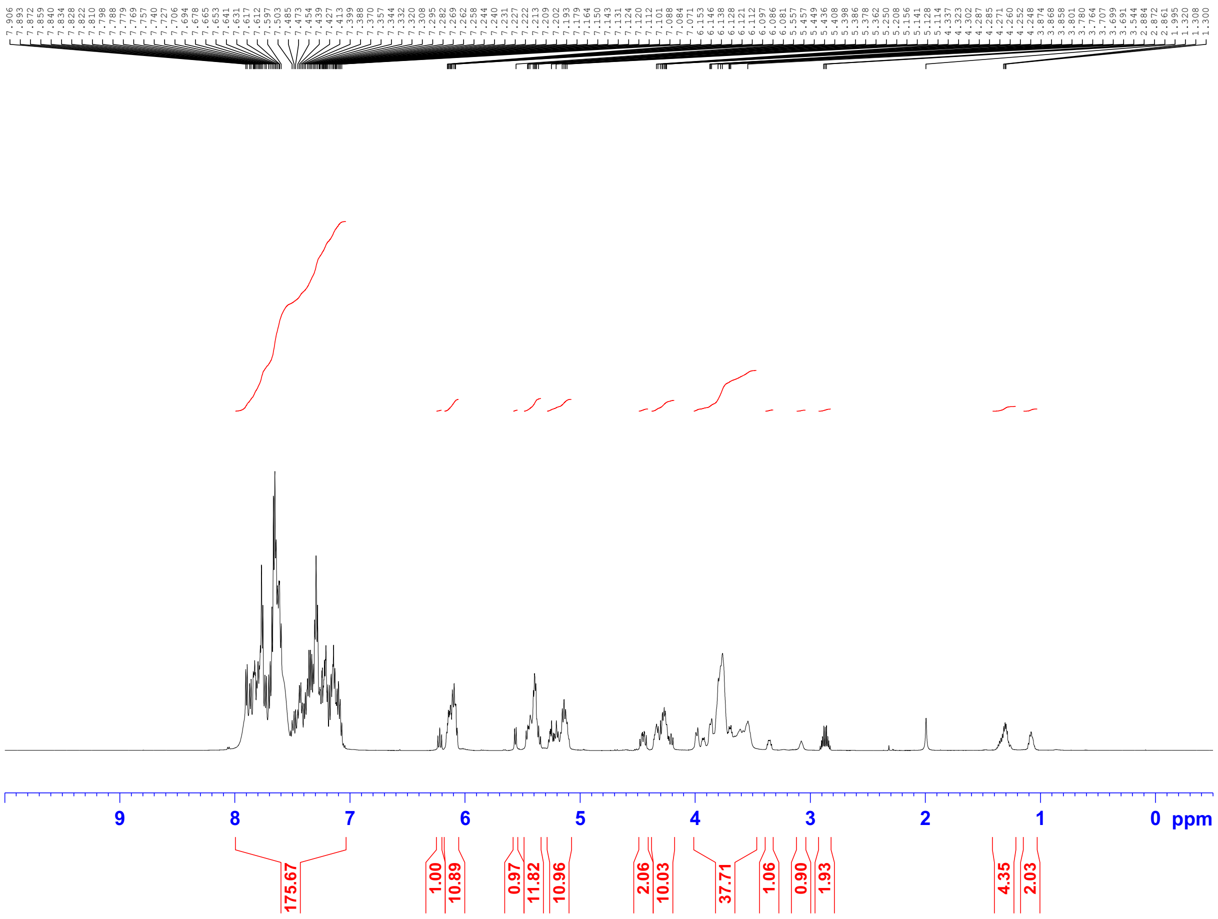

Figure S45. ¹H NMR spectrum of Compound 22

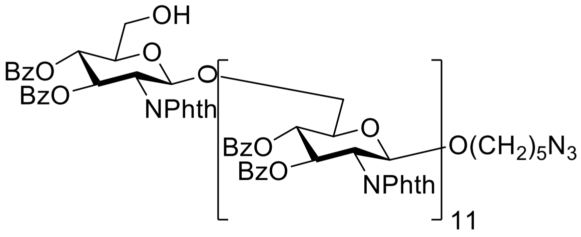

22

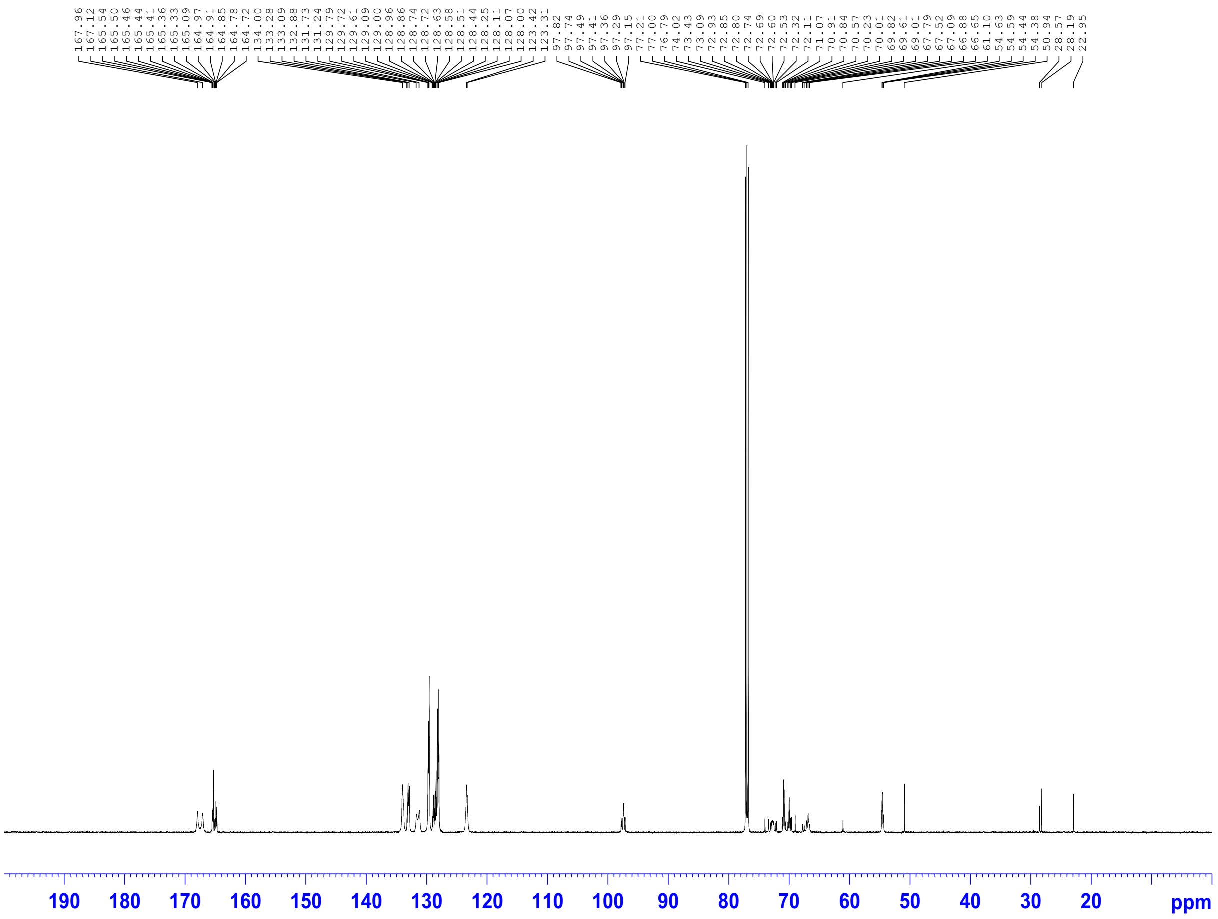

Figure S46. ^{13}C NMR spectrum of Compound 22

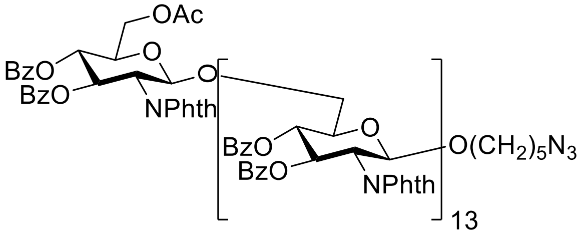

23

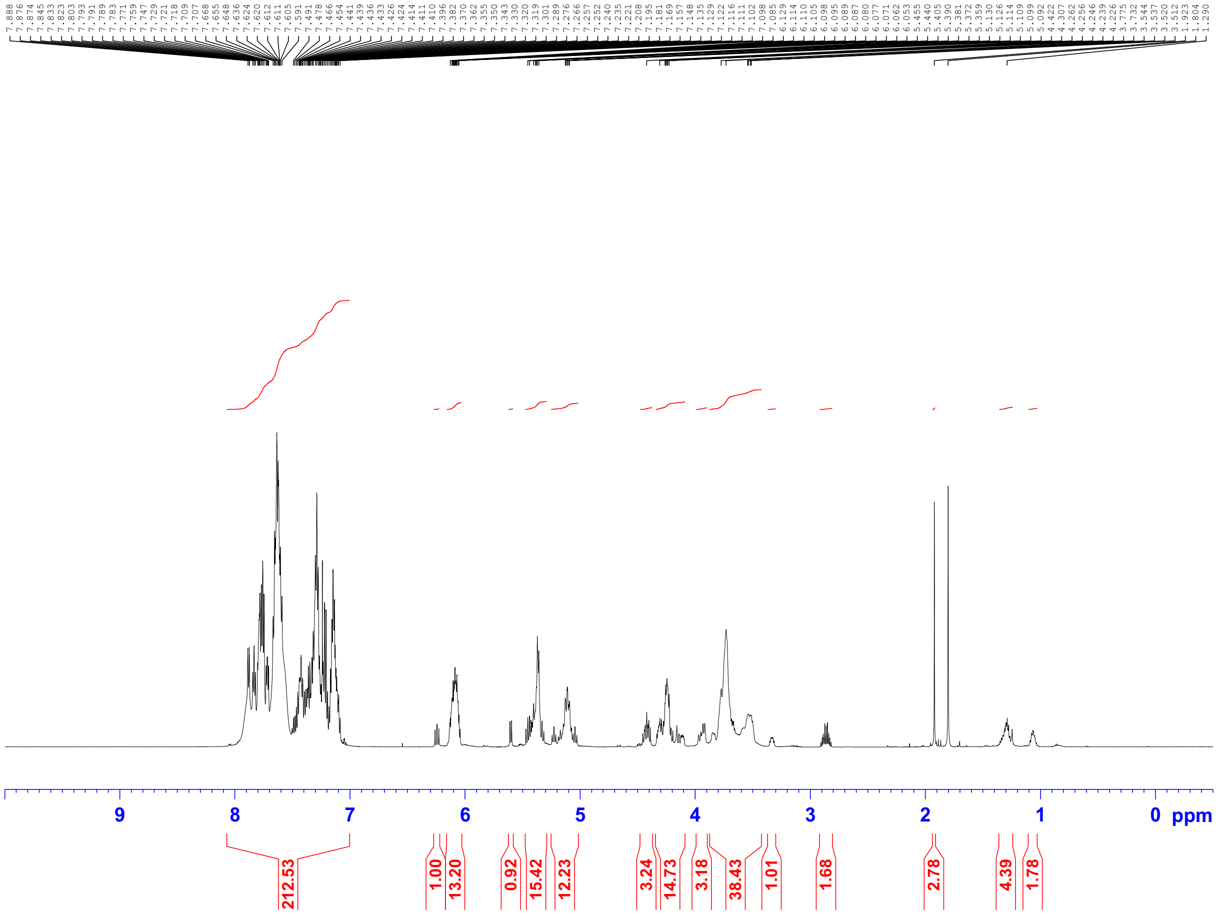

Figure S47. ^{1}H NMR spectrum of Compound 23

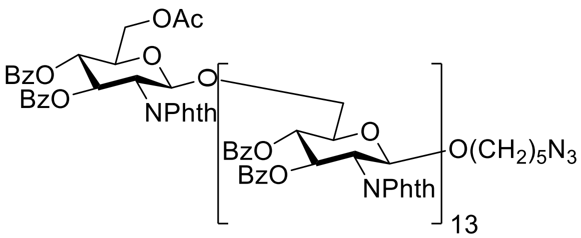

23

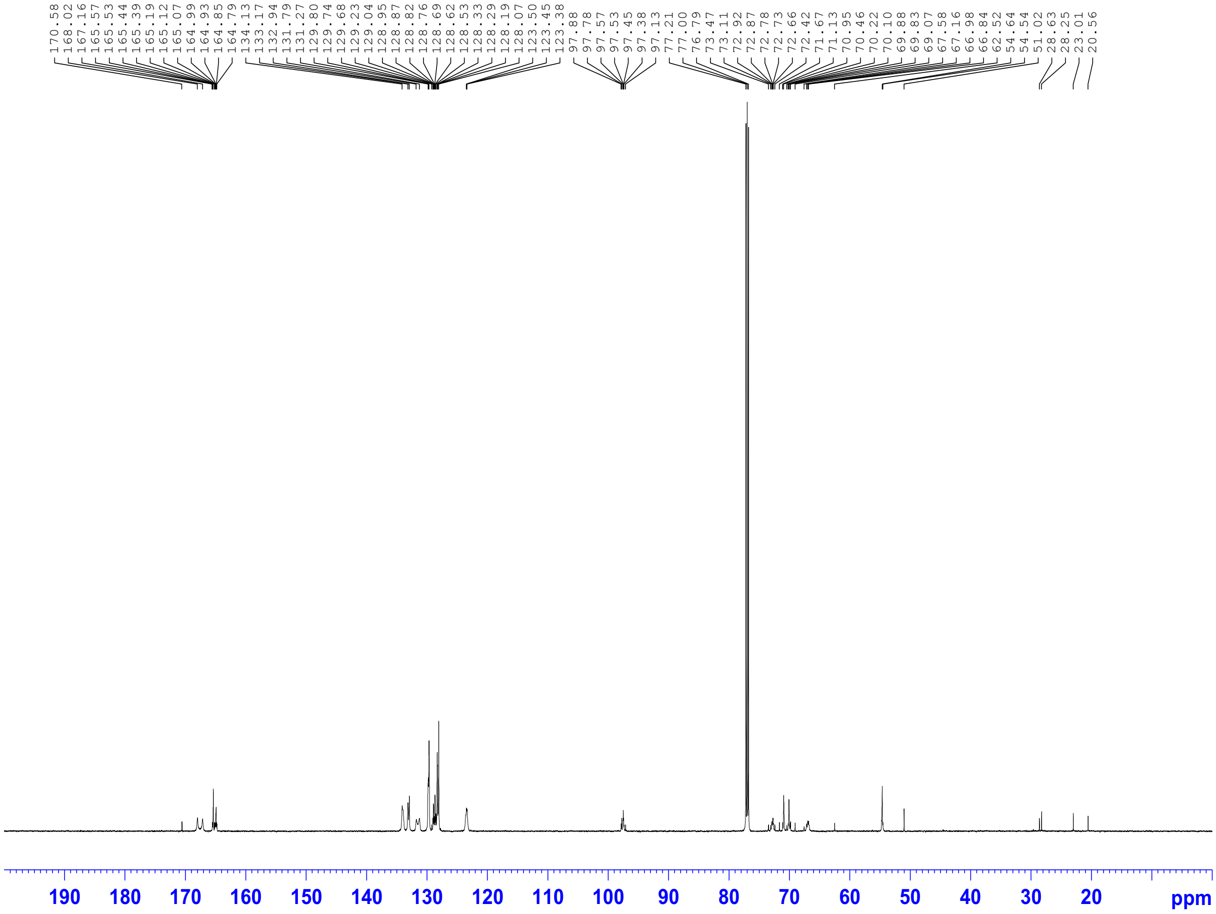

Figure S48. ^{13}C NMR spectrum of Compound 23

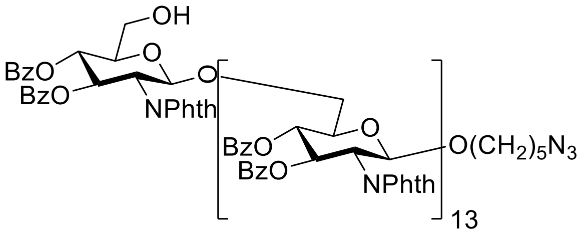

24

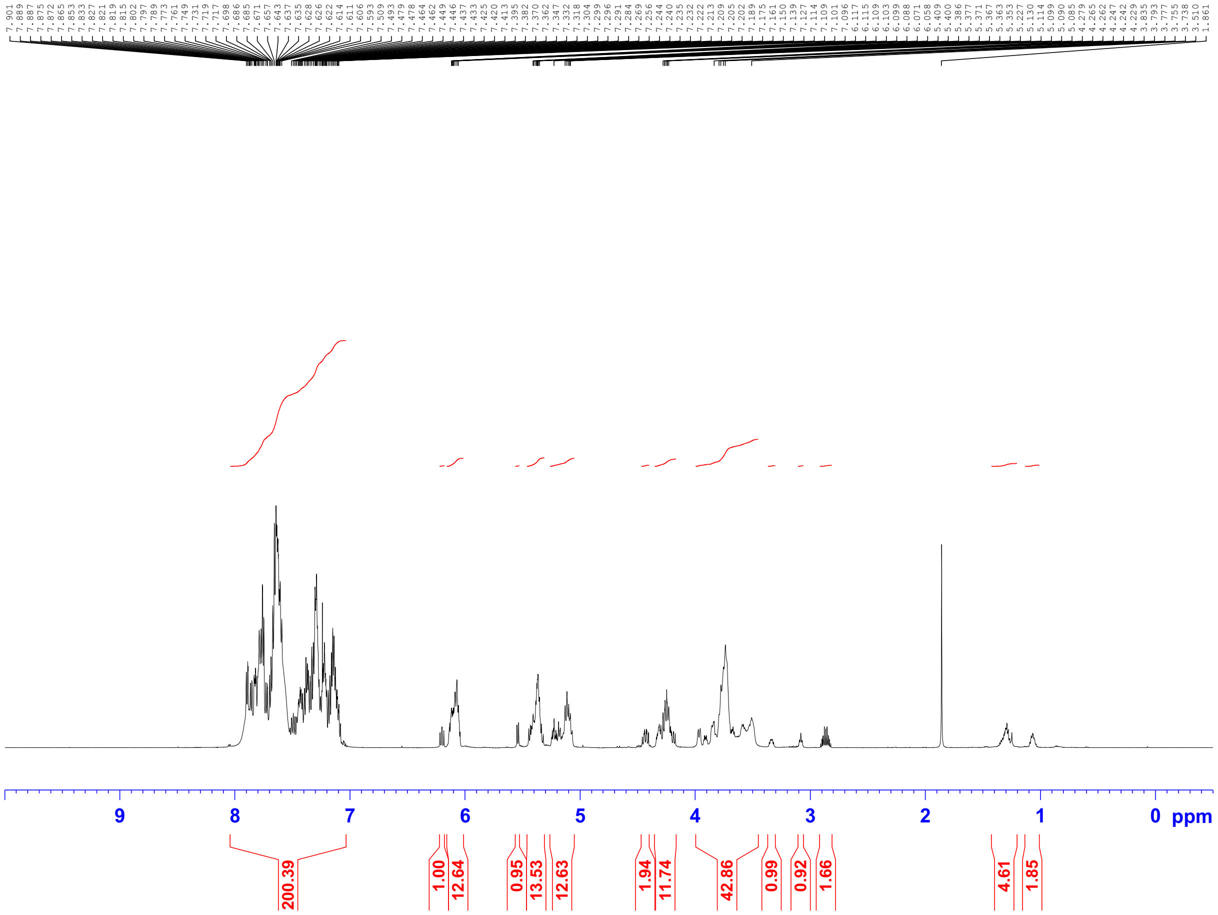

Figure S49. ^{1}H NMR spectrum of Compound 24

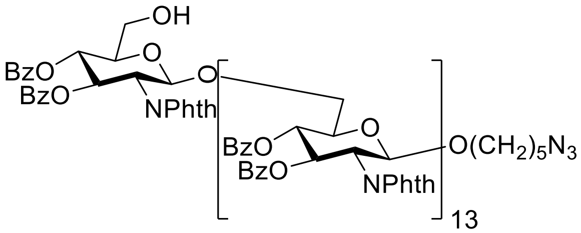

24

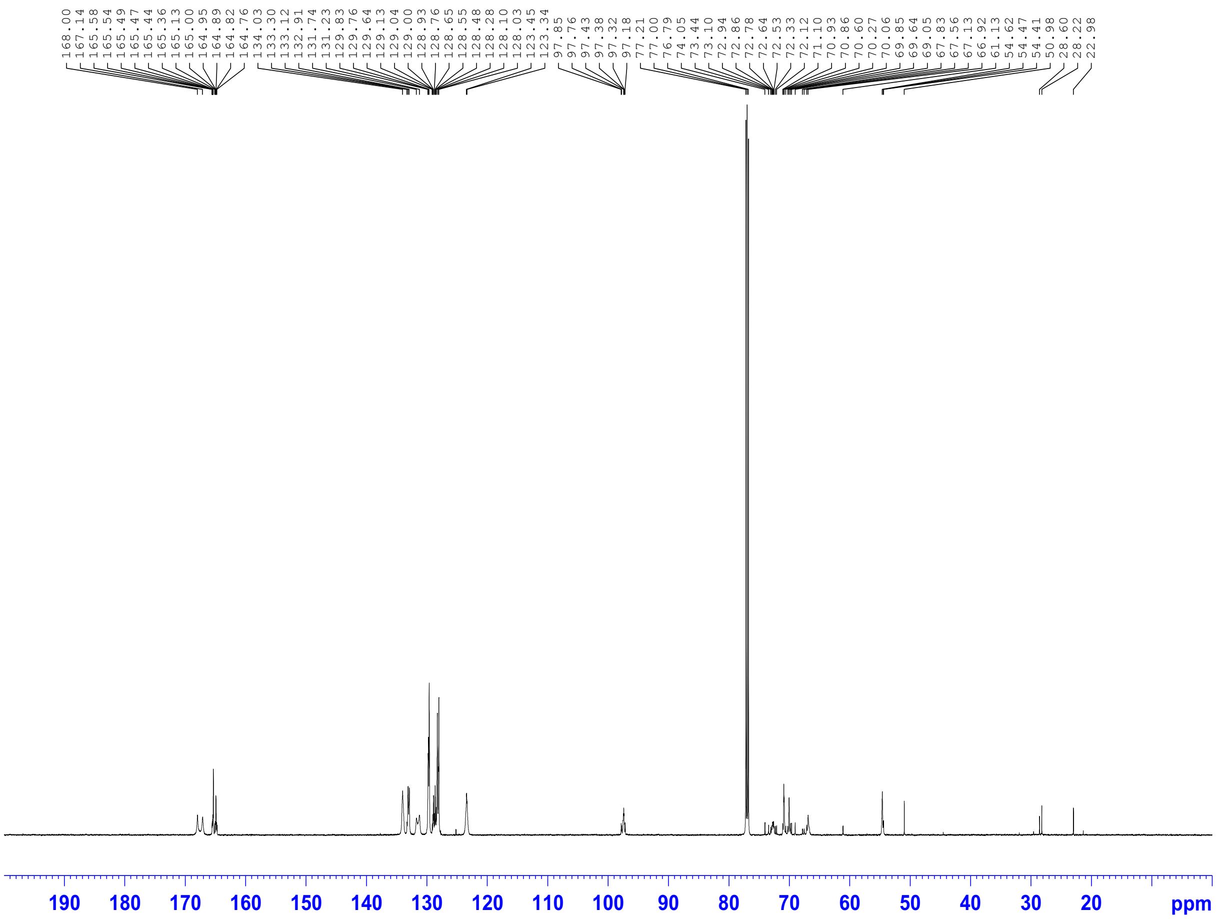

Figure S50. ^{13}**C NMR spectrum of Compound 24**

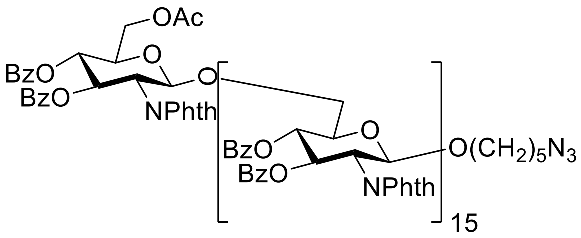

25

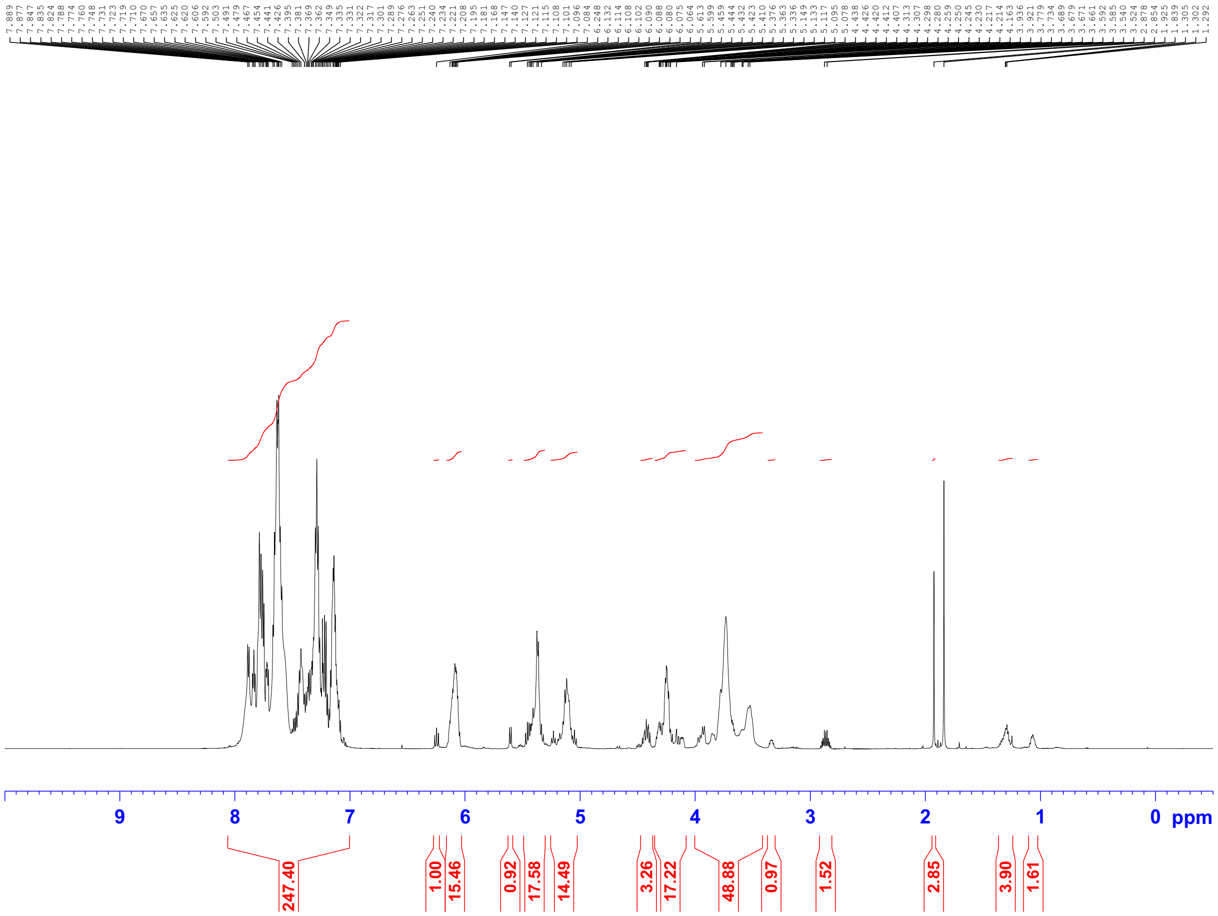

Figure S51. ^{1}**H NMR spectrum of Compound 25**

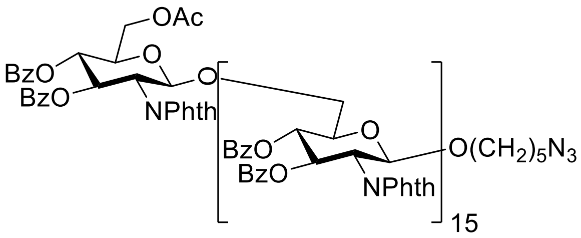

25

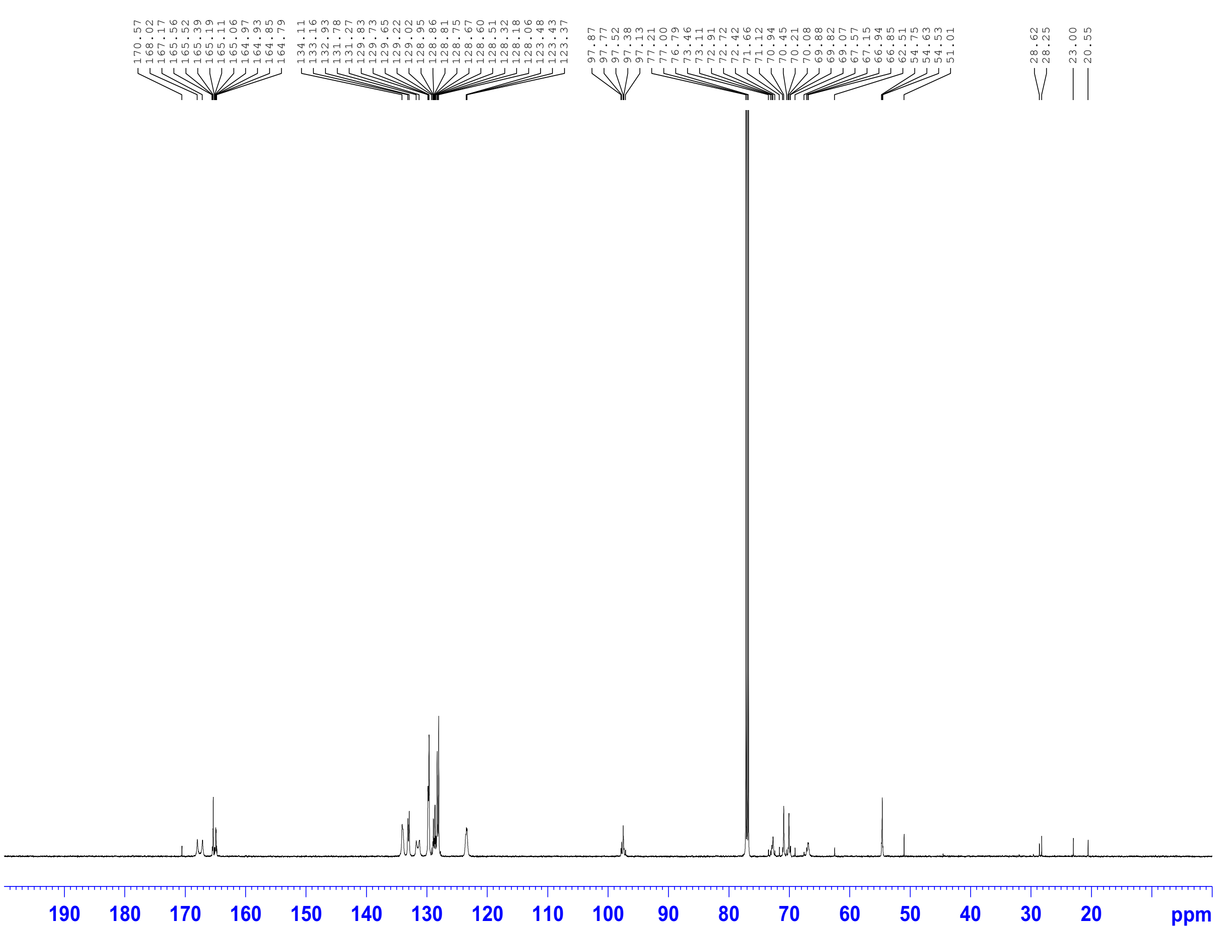

Figure S52. ^{13}C NMR spectrum of Compound 25

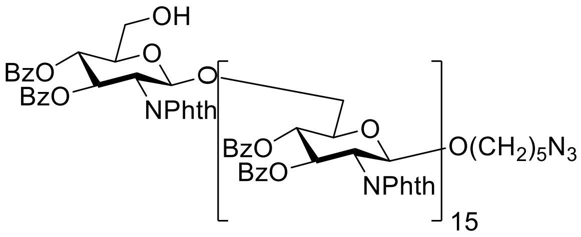

26

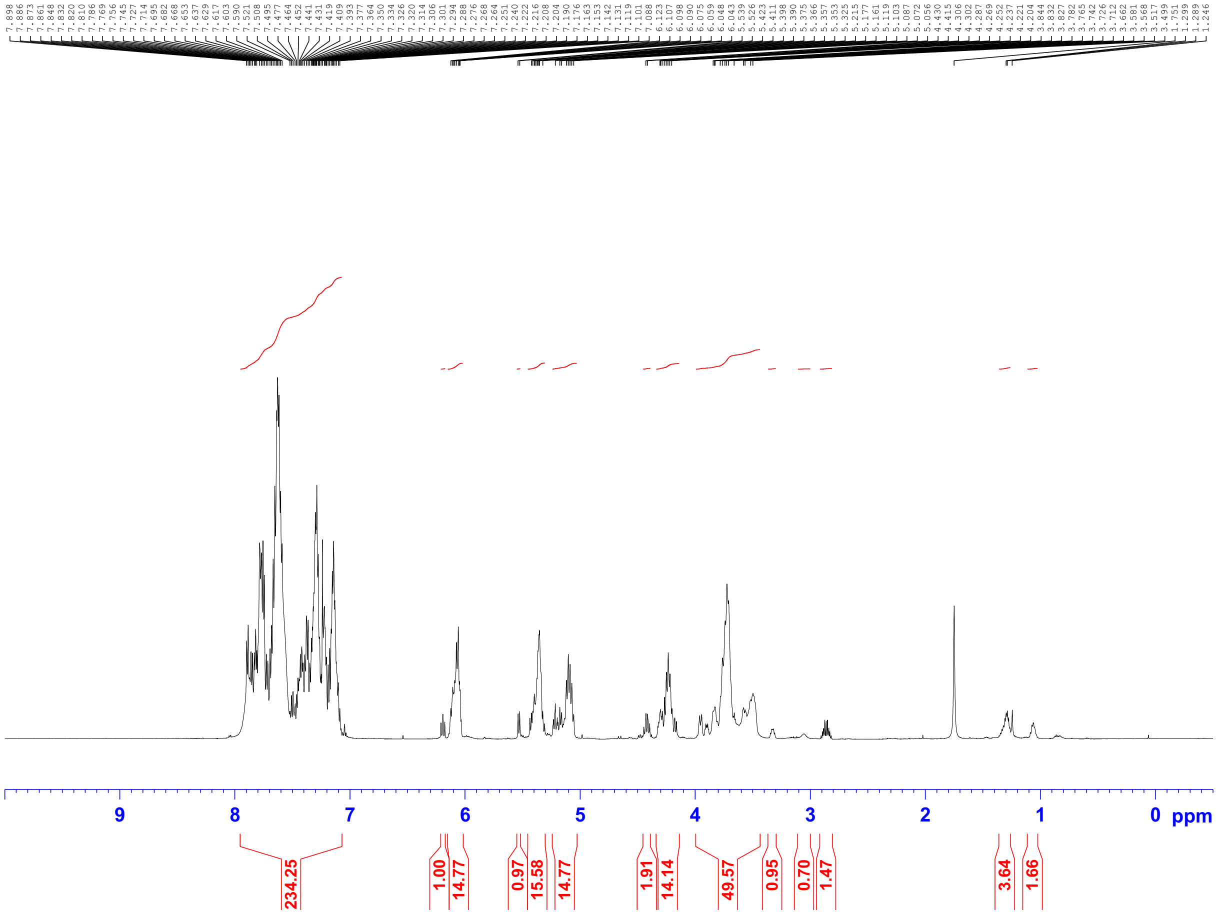

Figure S53. ^{1}H NMR spectrum of Compound 26

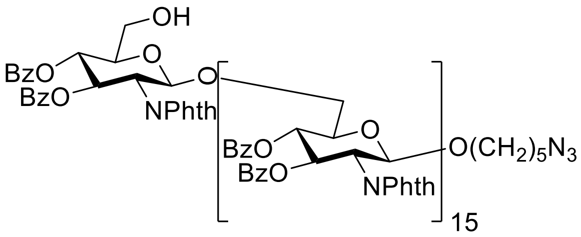

26

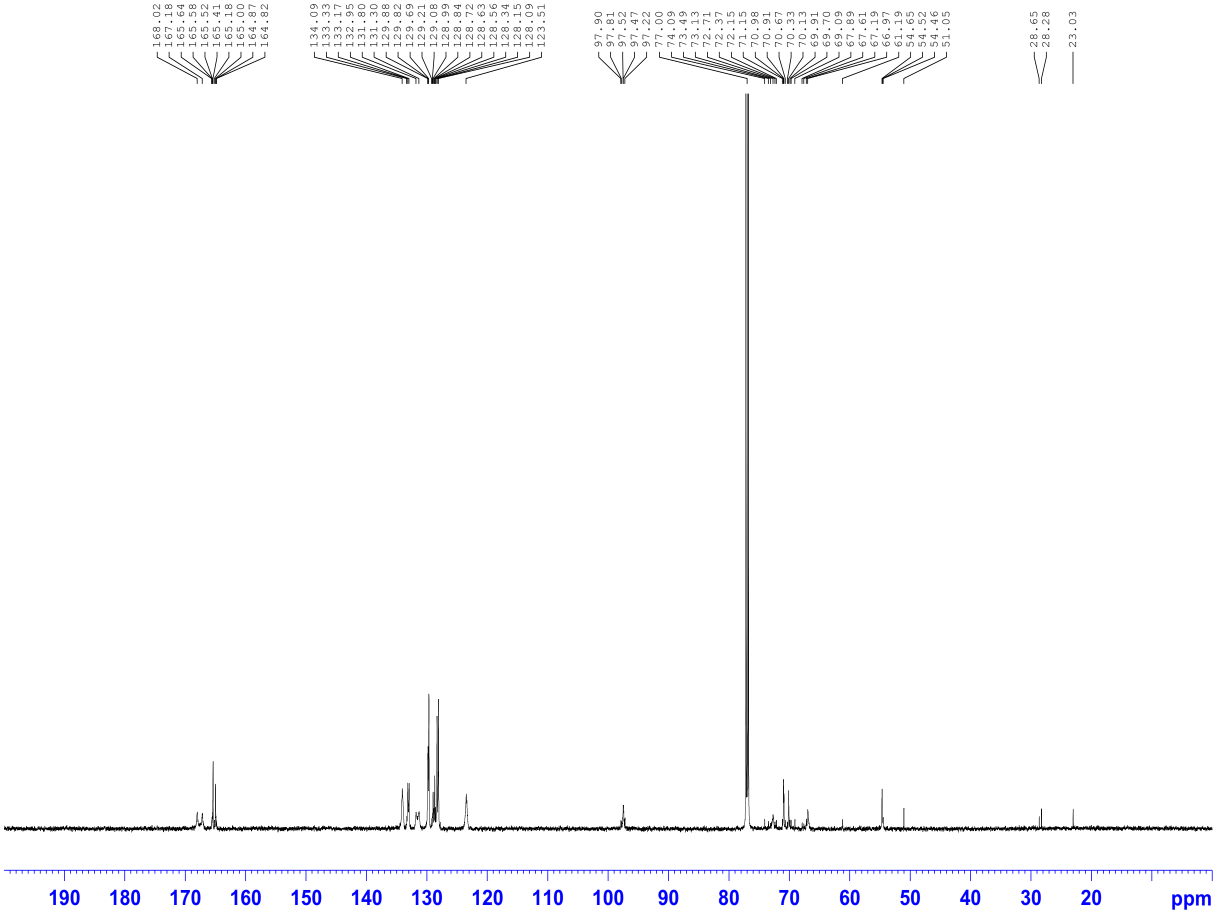

Figure S54. ^{13}C NMR spectrum of Compound 26

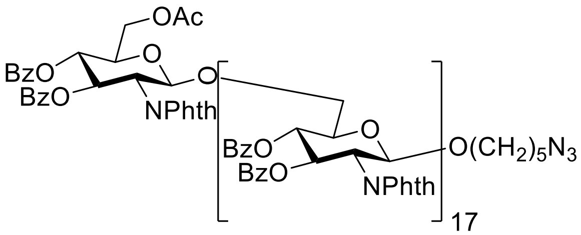

27

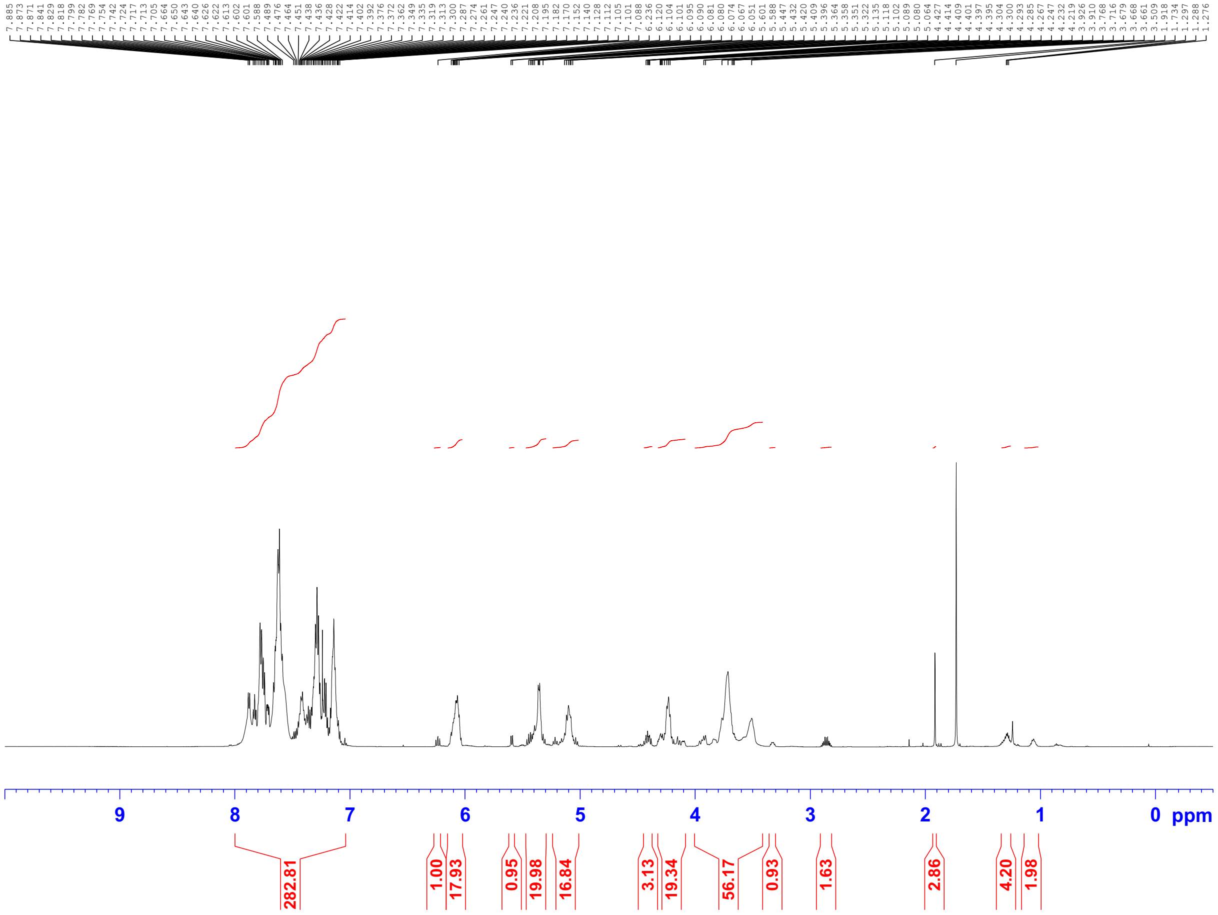

Figure S55. ^{1}H NMR spectrum of Compound 27

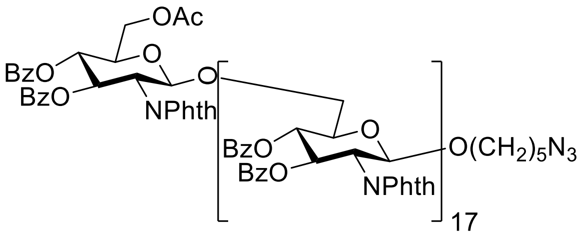

27

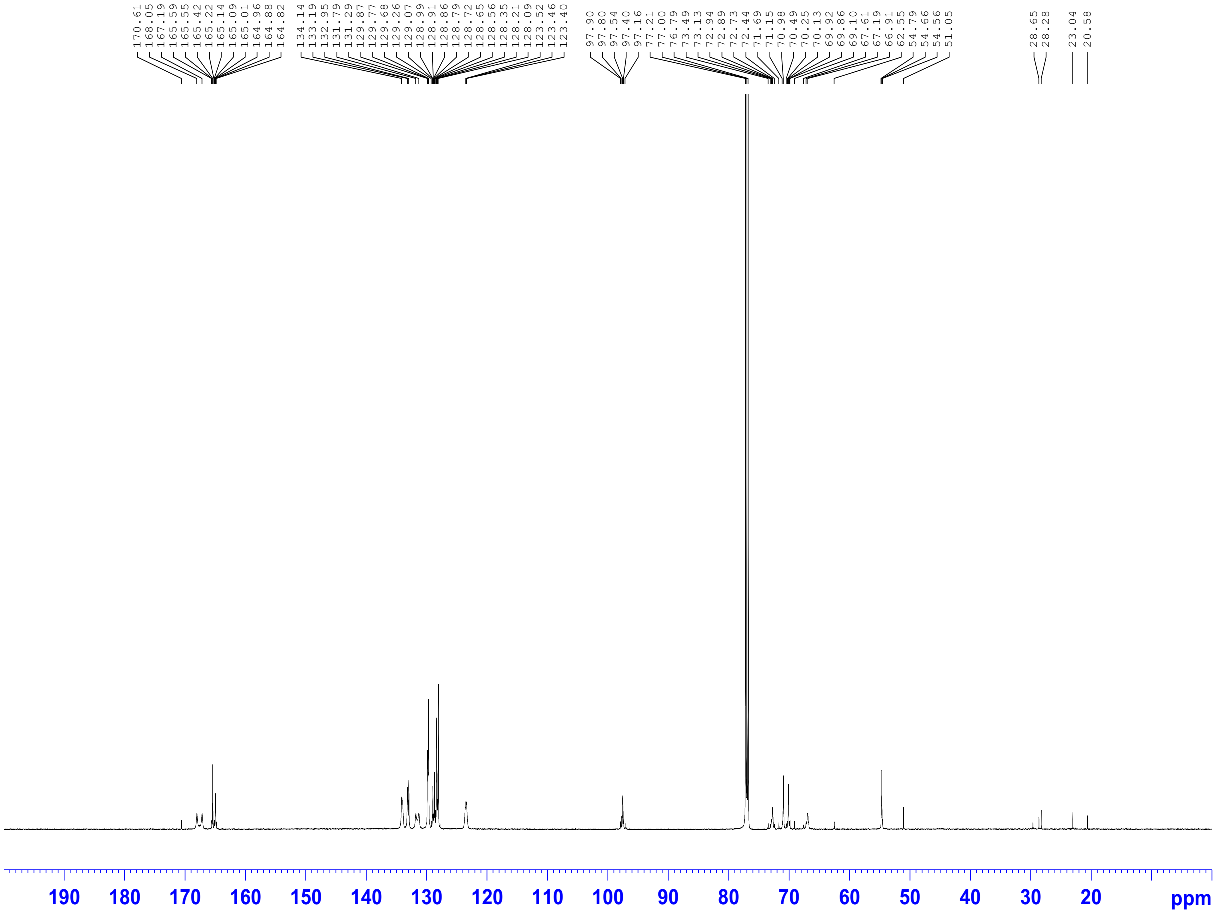

Figure S56. ^{13}**C NMR spectrum of Compound 27**

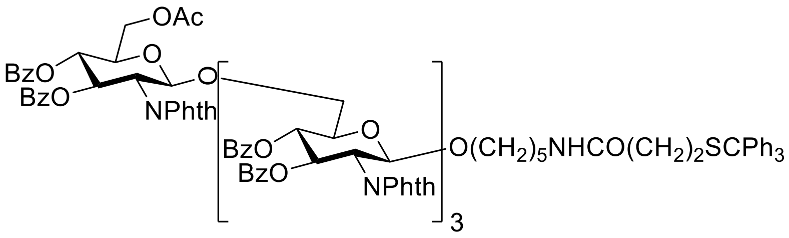

28

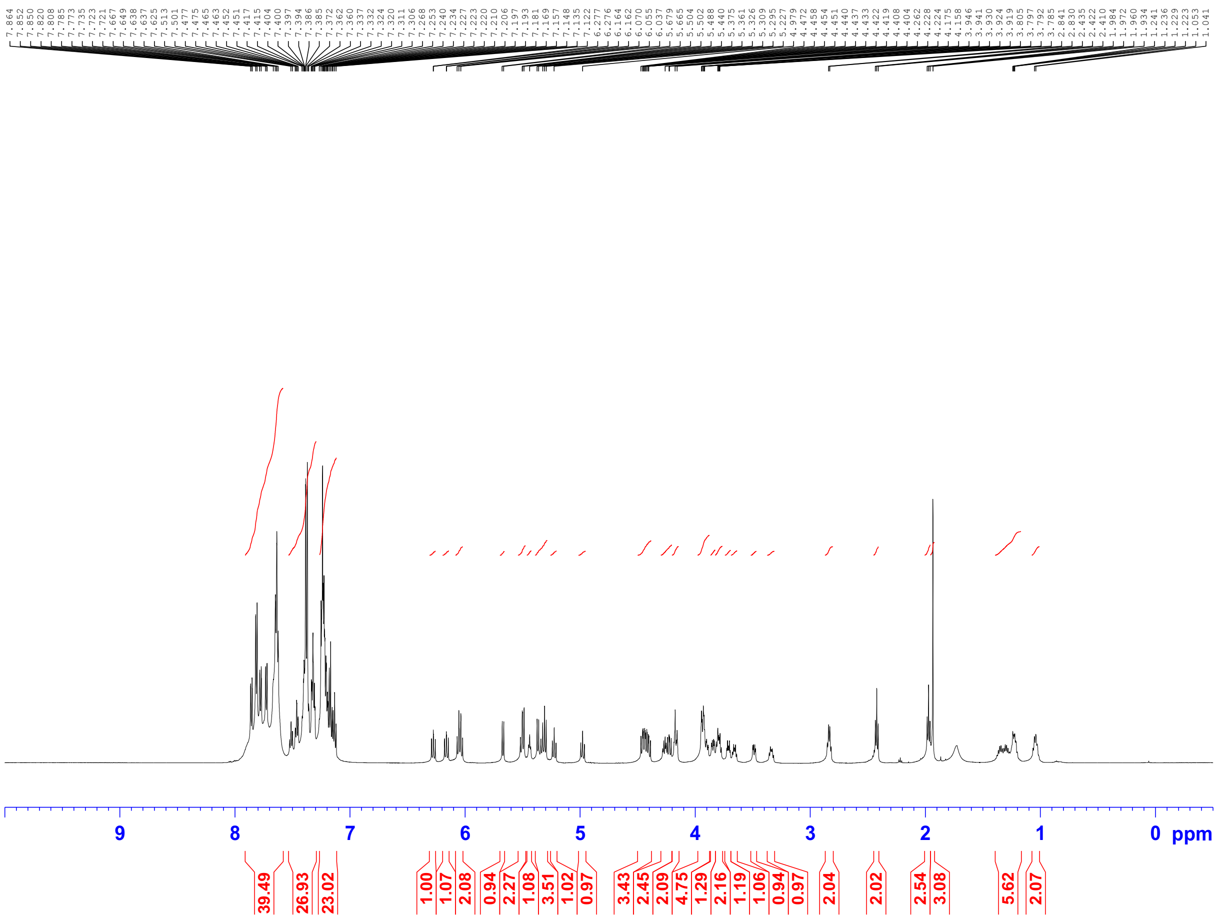

Figure S57. ^{1}H NMR spectrum of Compound 28

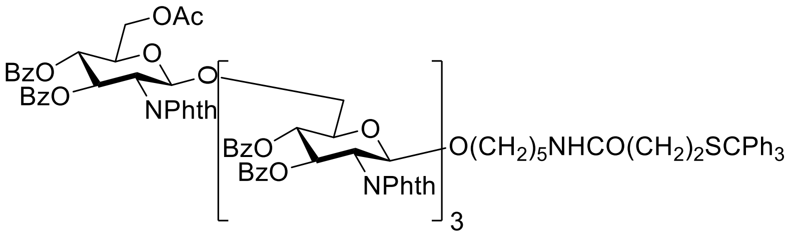

28

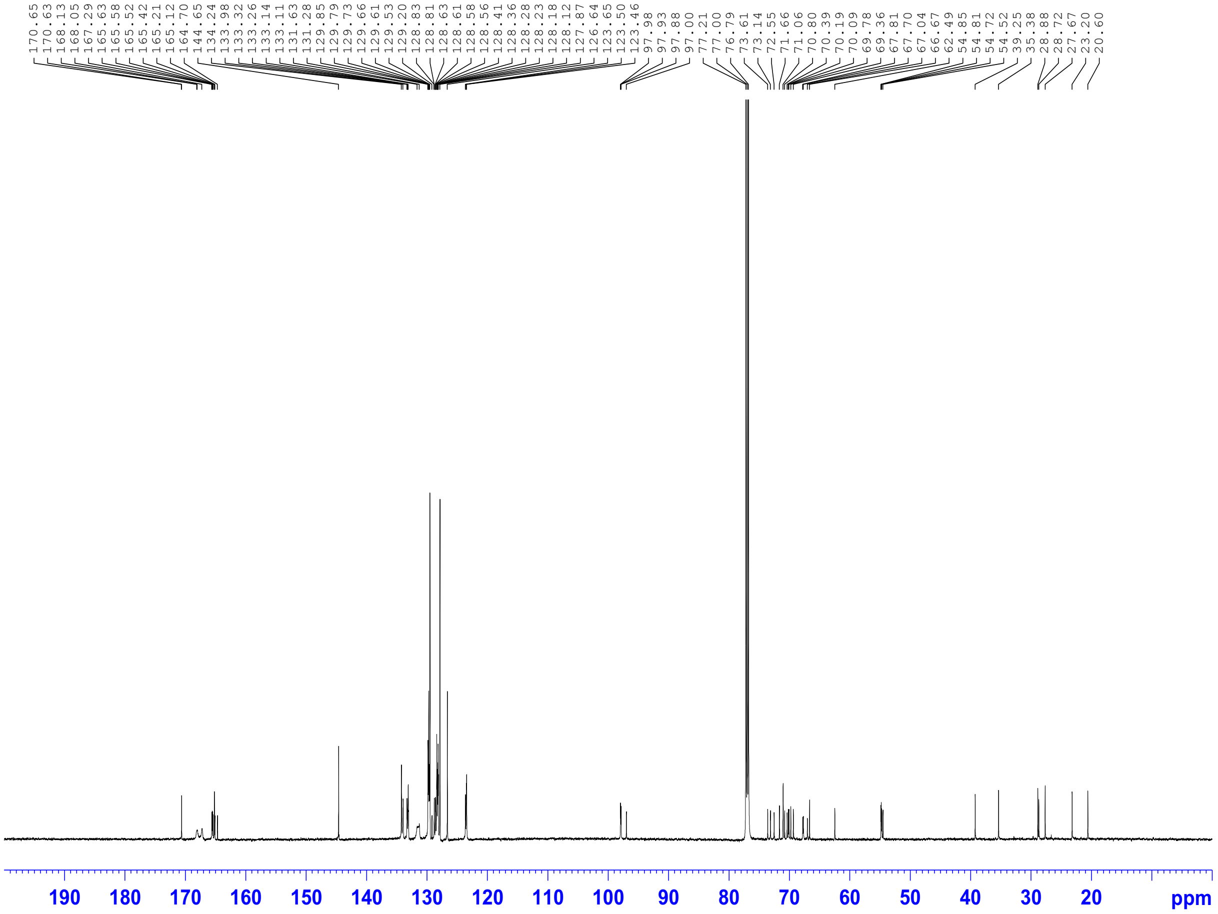

Figure S58. ^{13}C NMR spectrum of Compound 28

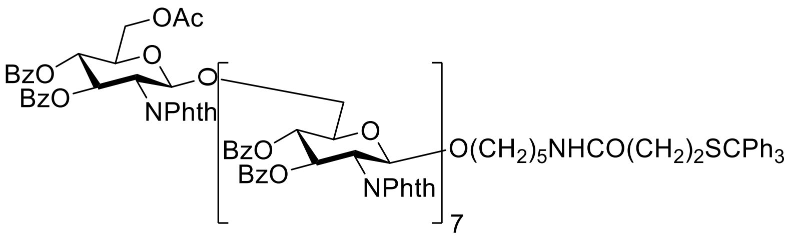

29

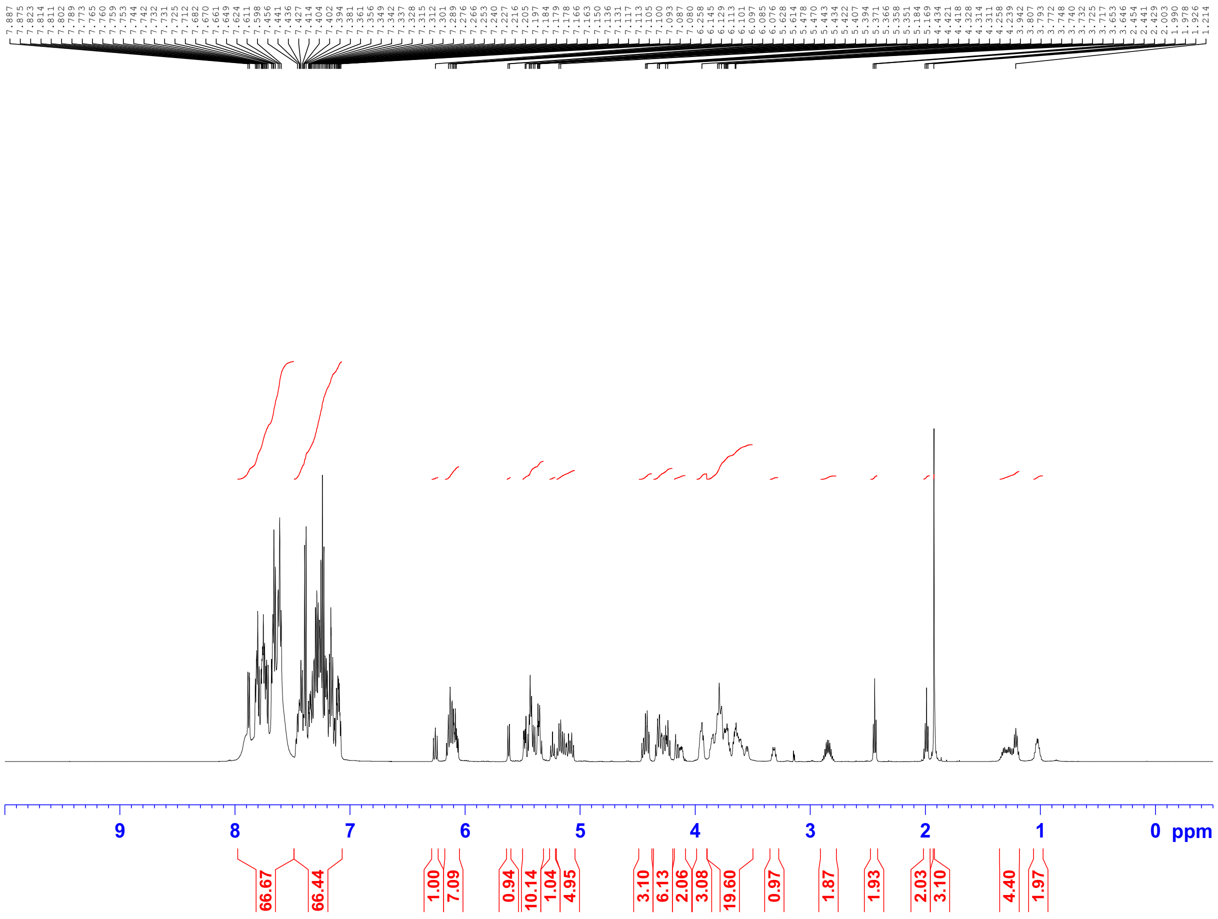

Figure S59. ^{1}H NMR spectrum of Compound 29

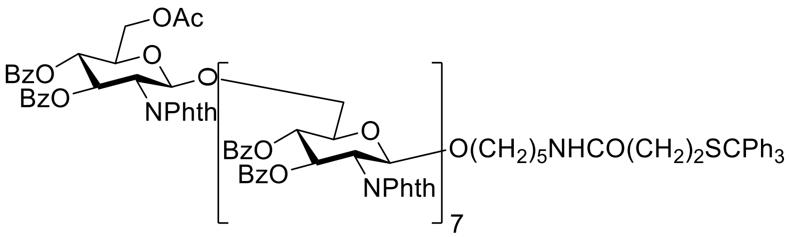

29

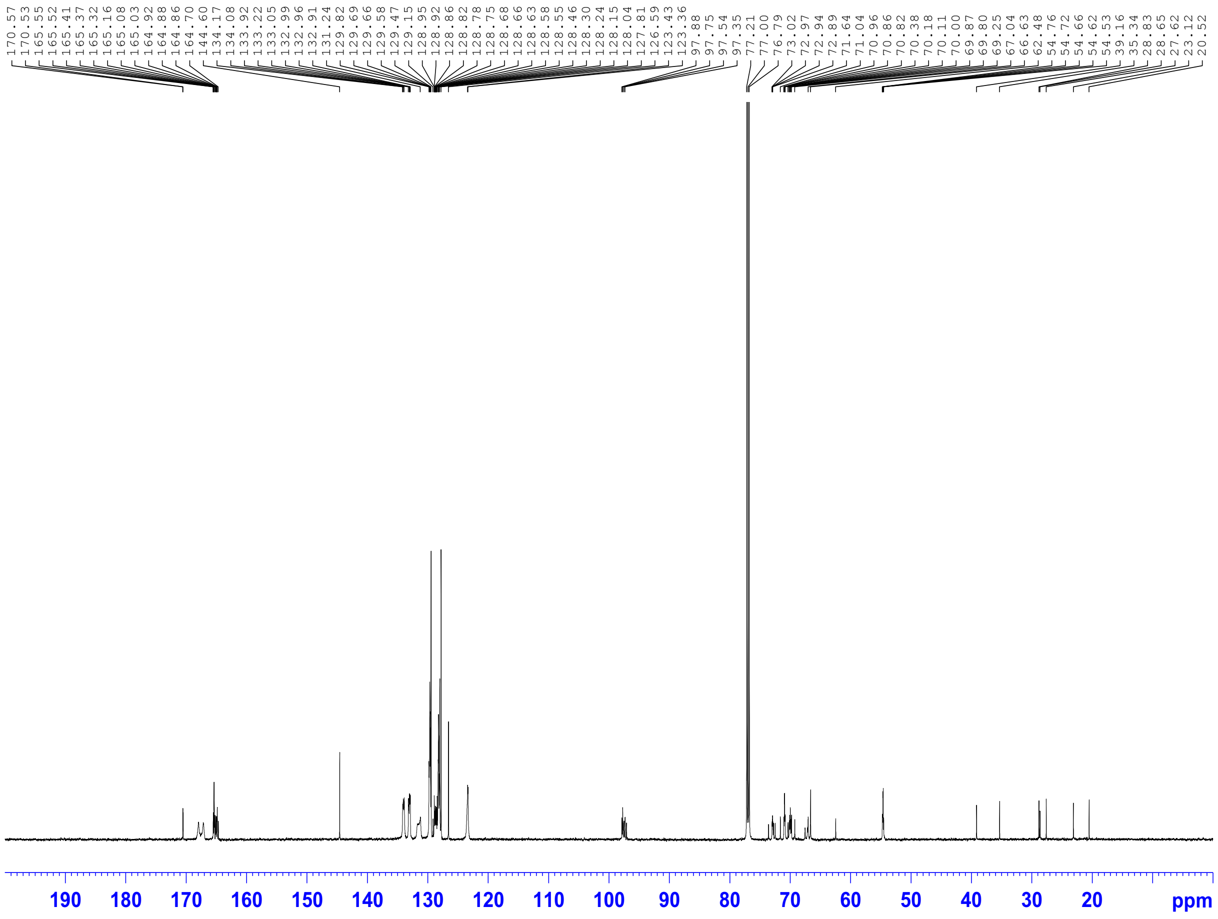

Figure S60. ^{13}C NMR spectrum of Compound 29

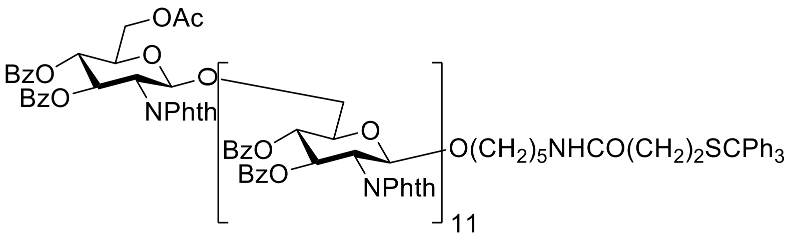

30

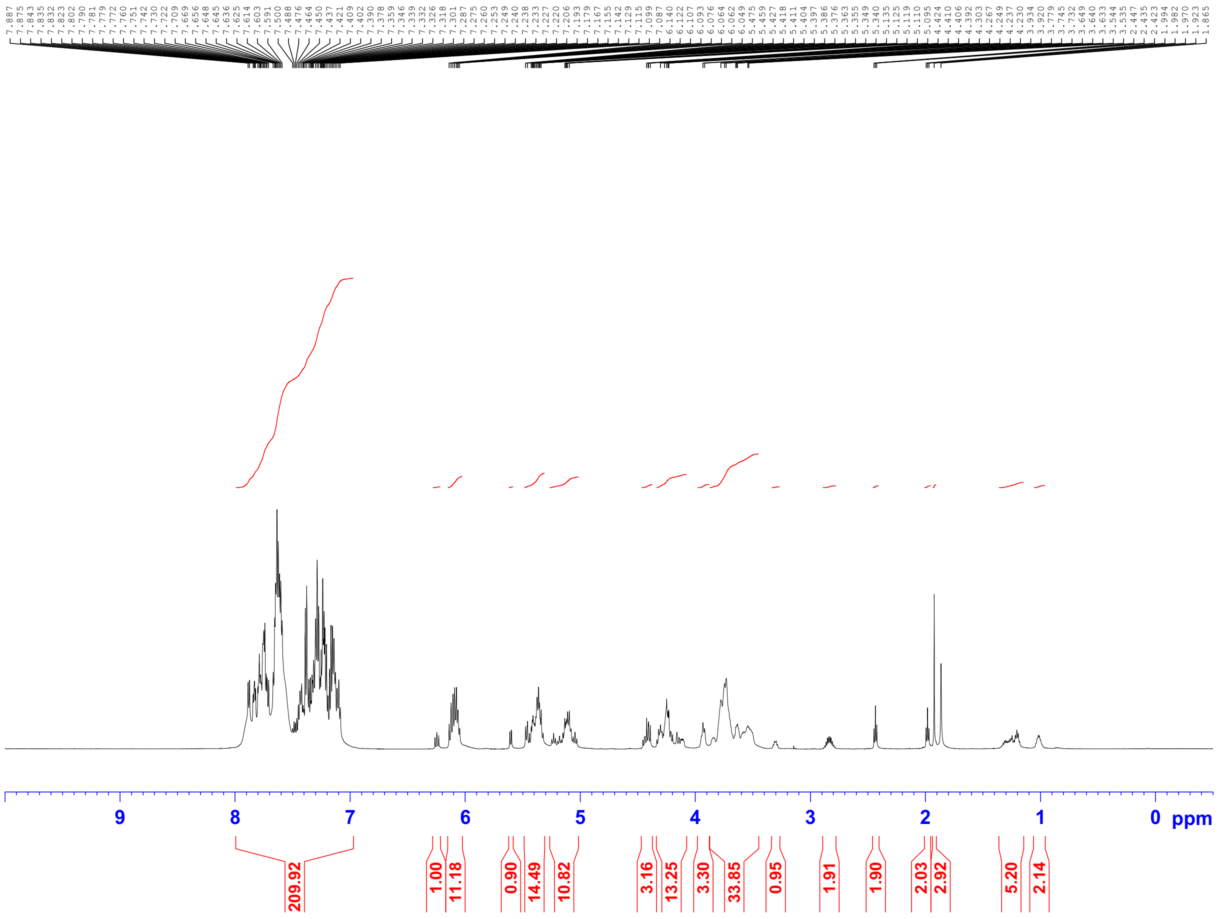

Figure S61. ^{1}H NMR spectrum of Compound 30

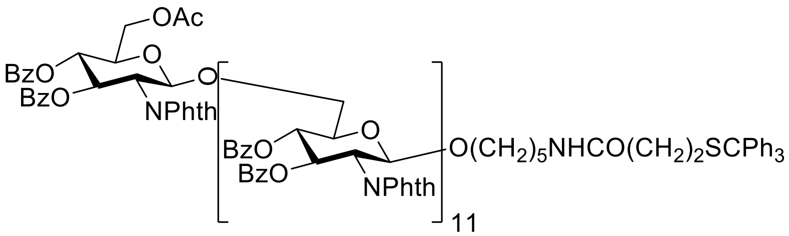

30

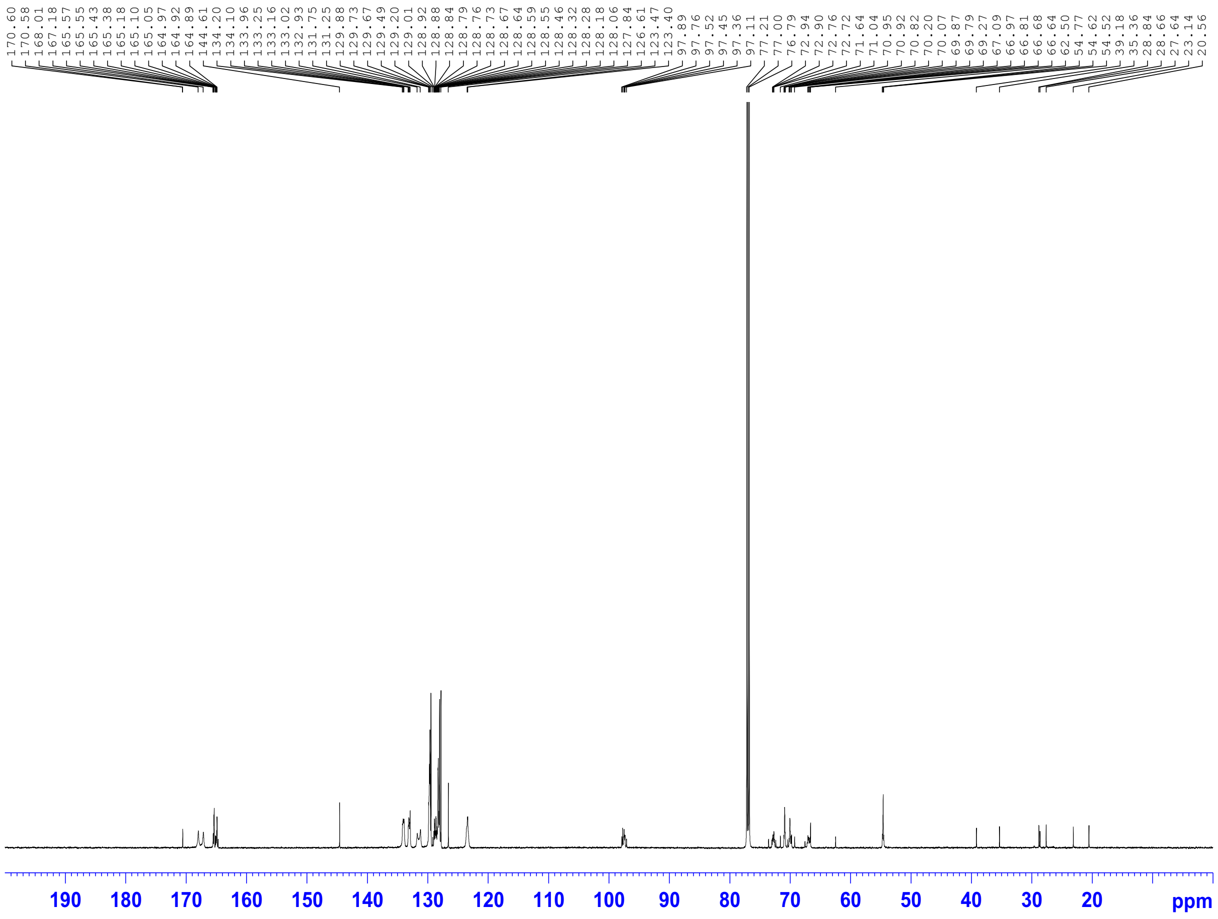

Figure S62. ^{13}**C NMR spectrum of Compound 30**

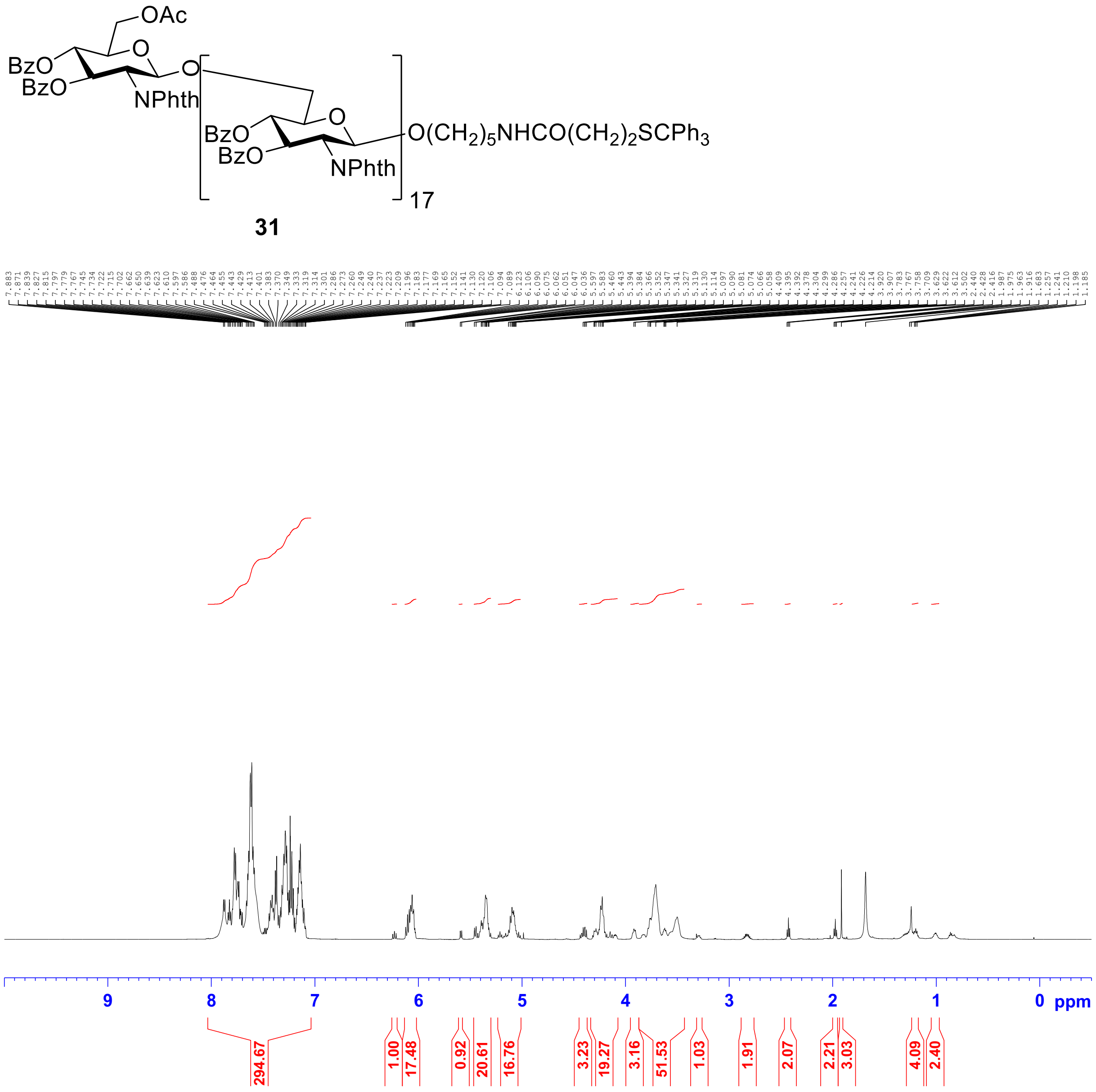

Figure S63. ^{1}**H NMR spectrum of Compound 31**

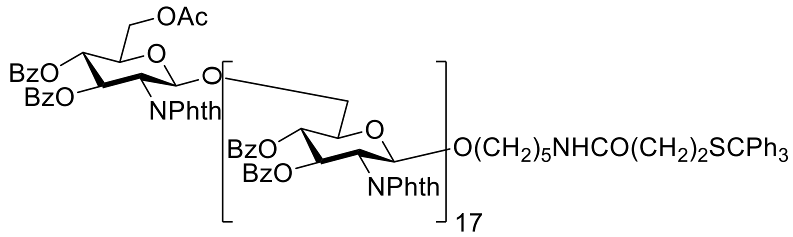

31

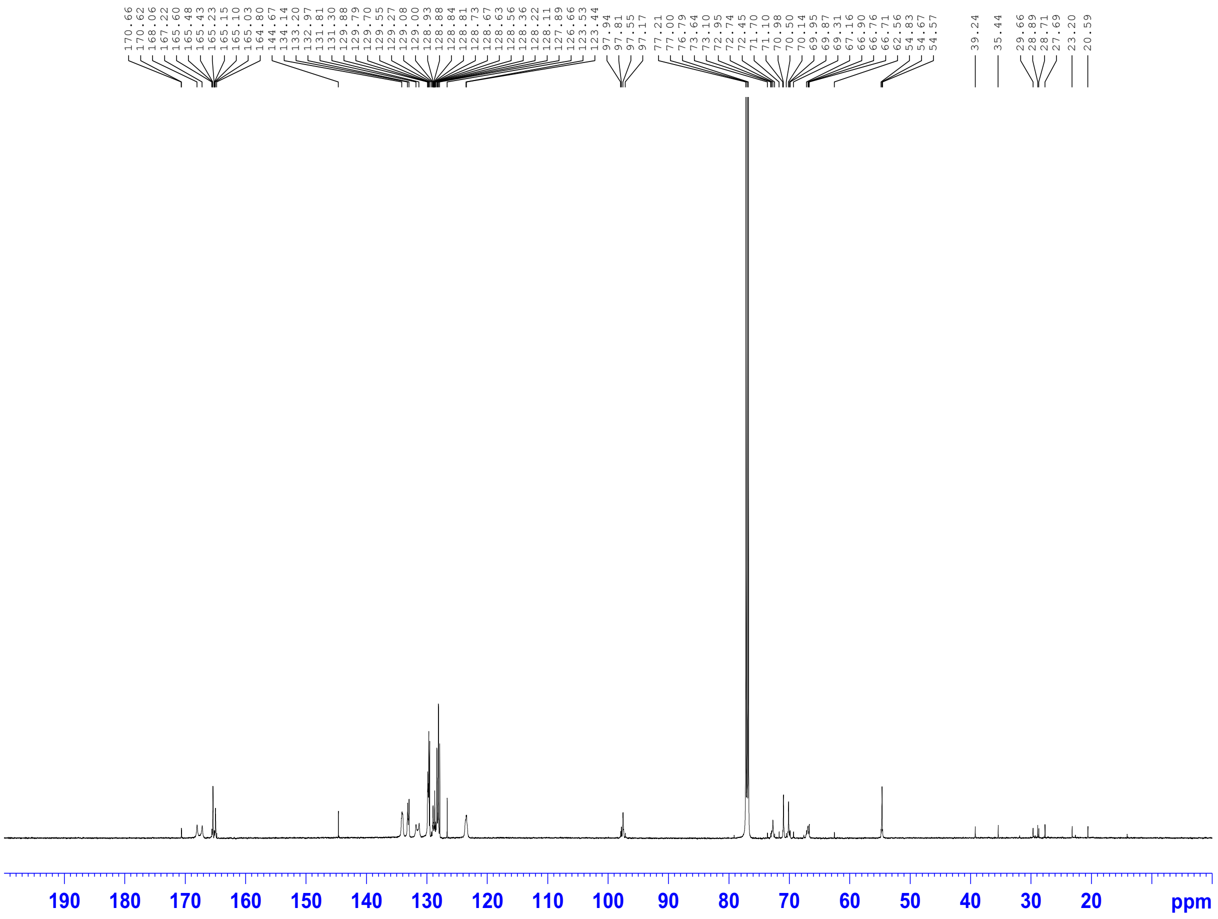

Figure S64. ^{13}C NMR spectrum of Compound 31

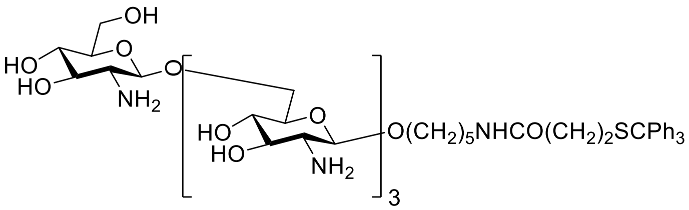

32

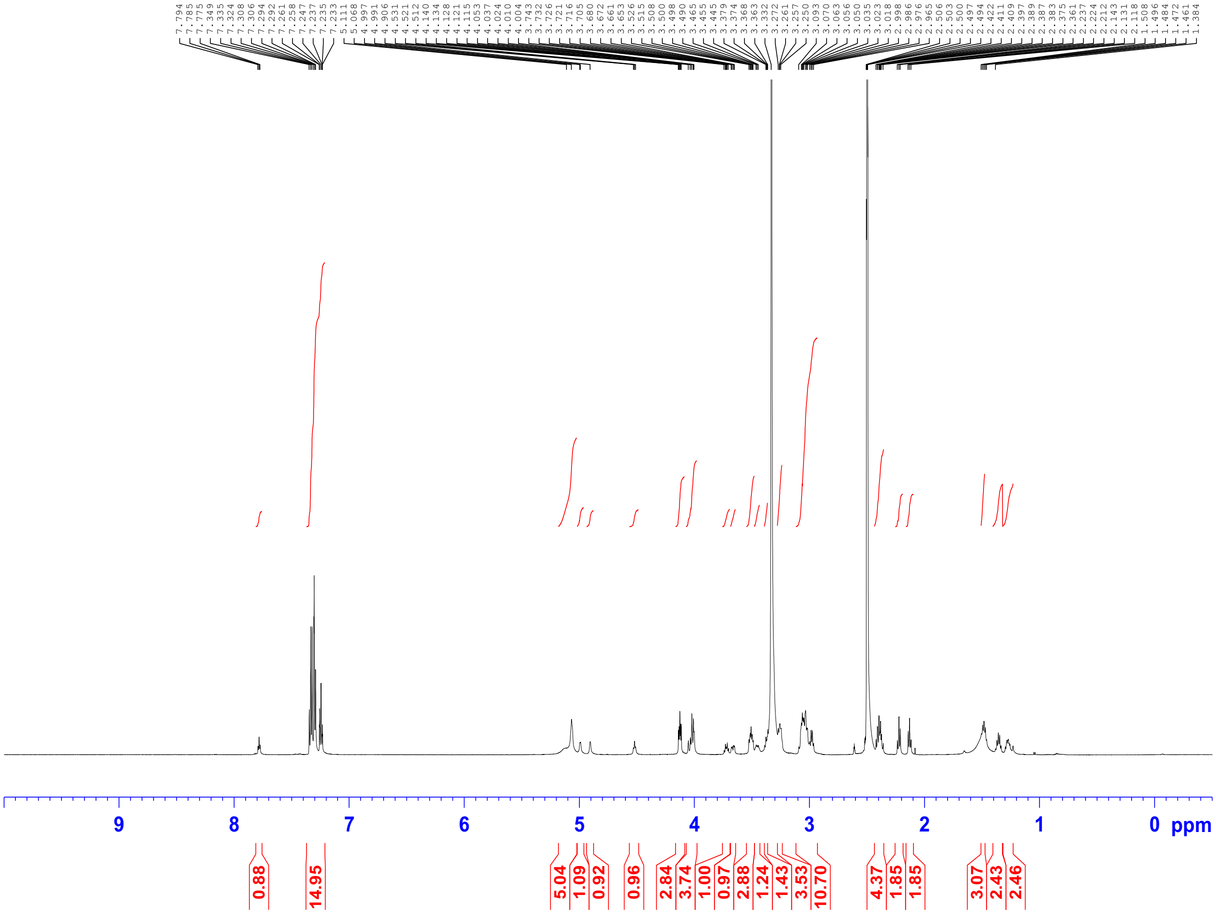

Figure S65. ^{1}H NMR spectrum of Compound 32

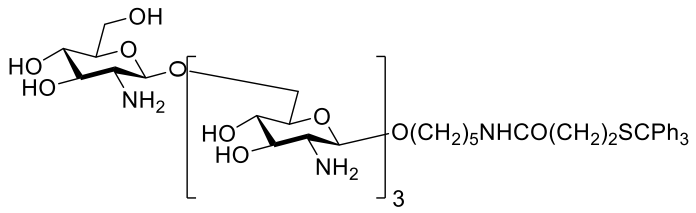

32

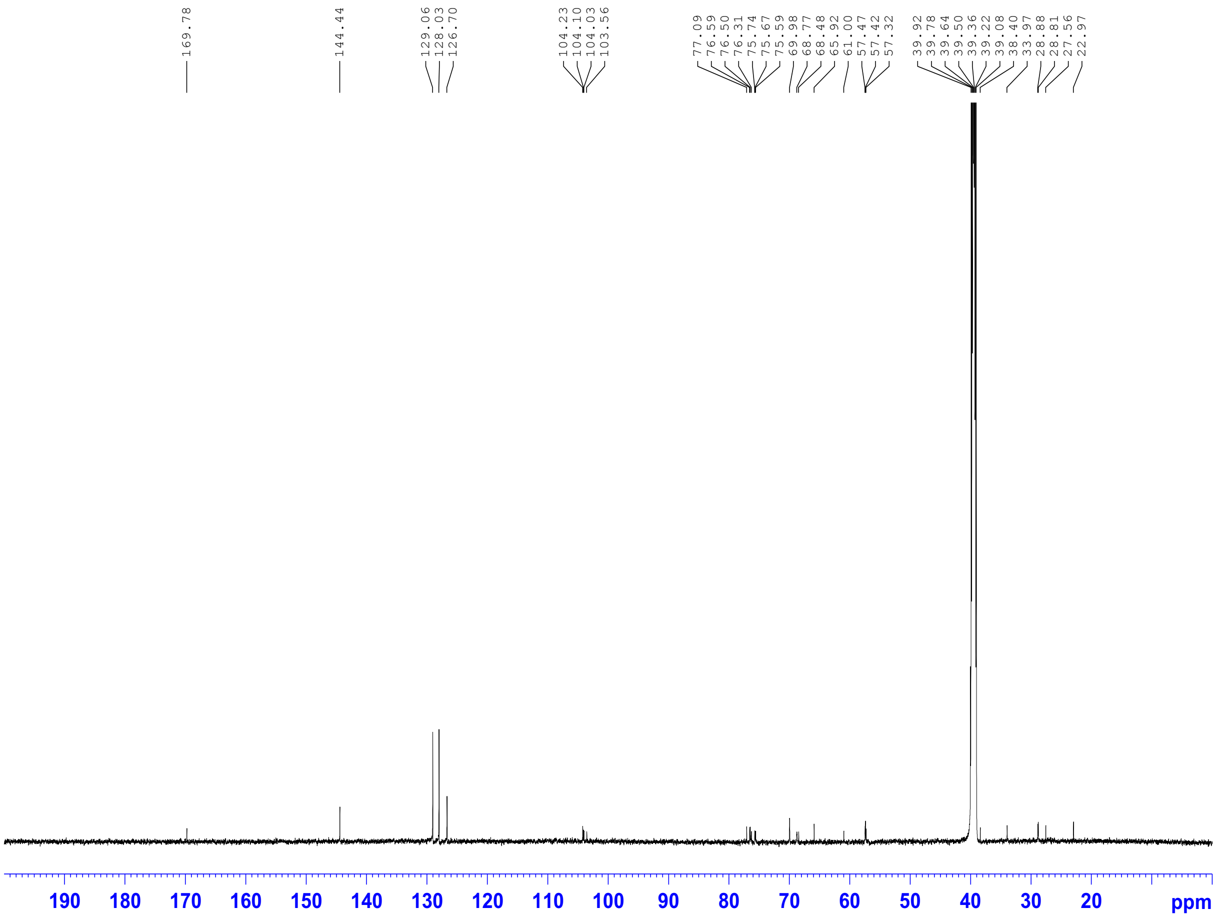

Figure S66. ^{13}C NMR spectrum of Compound 32

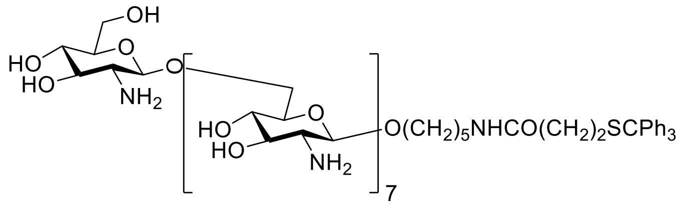

33

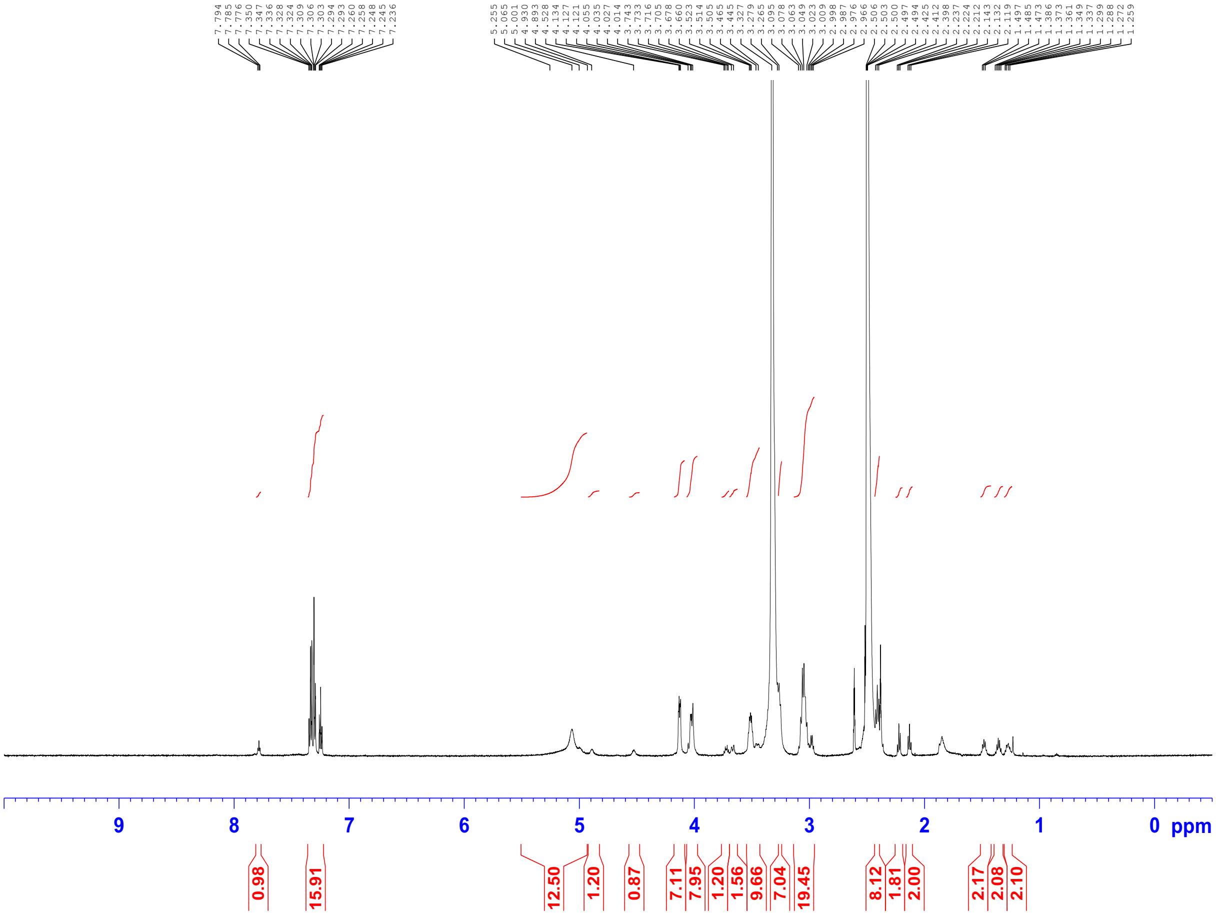

Figure S67. ^{1}H NMR spectrum of Compound 33

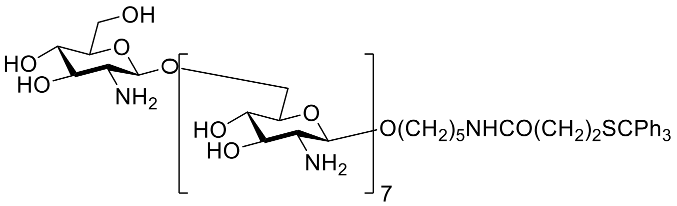

33

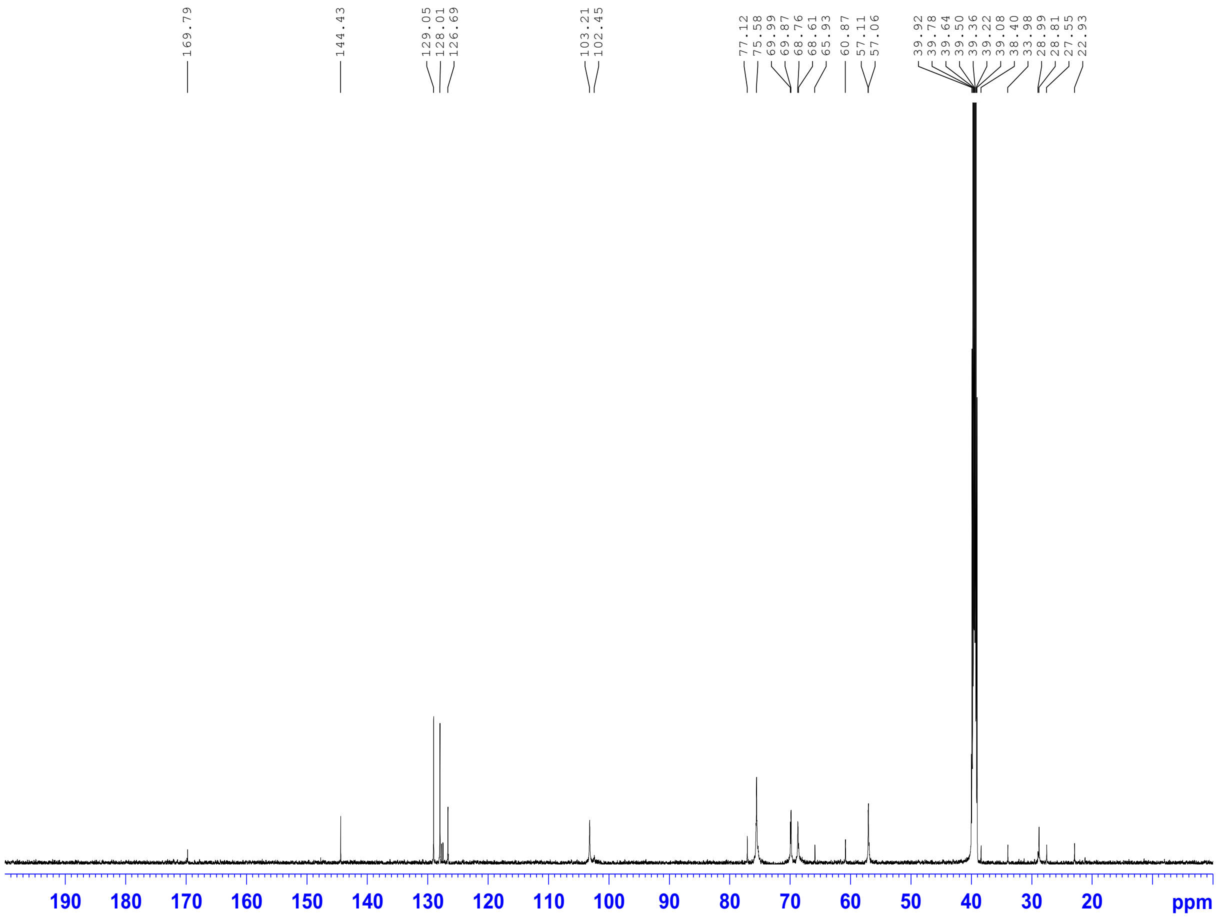

Figure S68. ^{13}C NMR spectrum of Compound 33

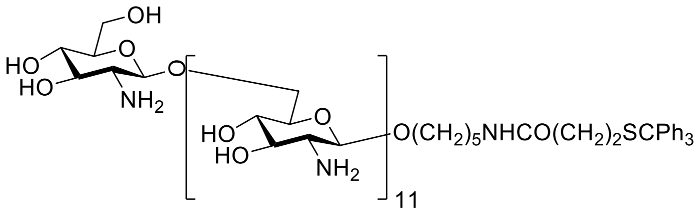

34

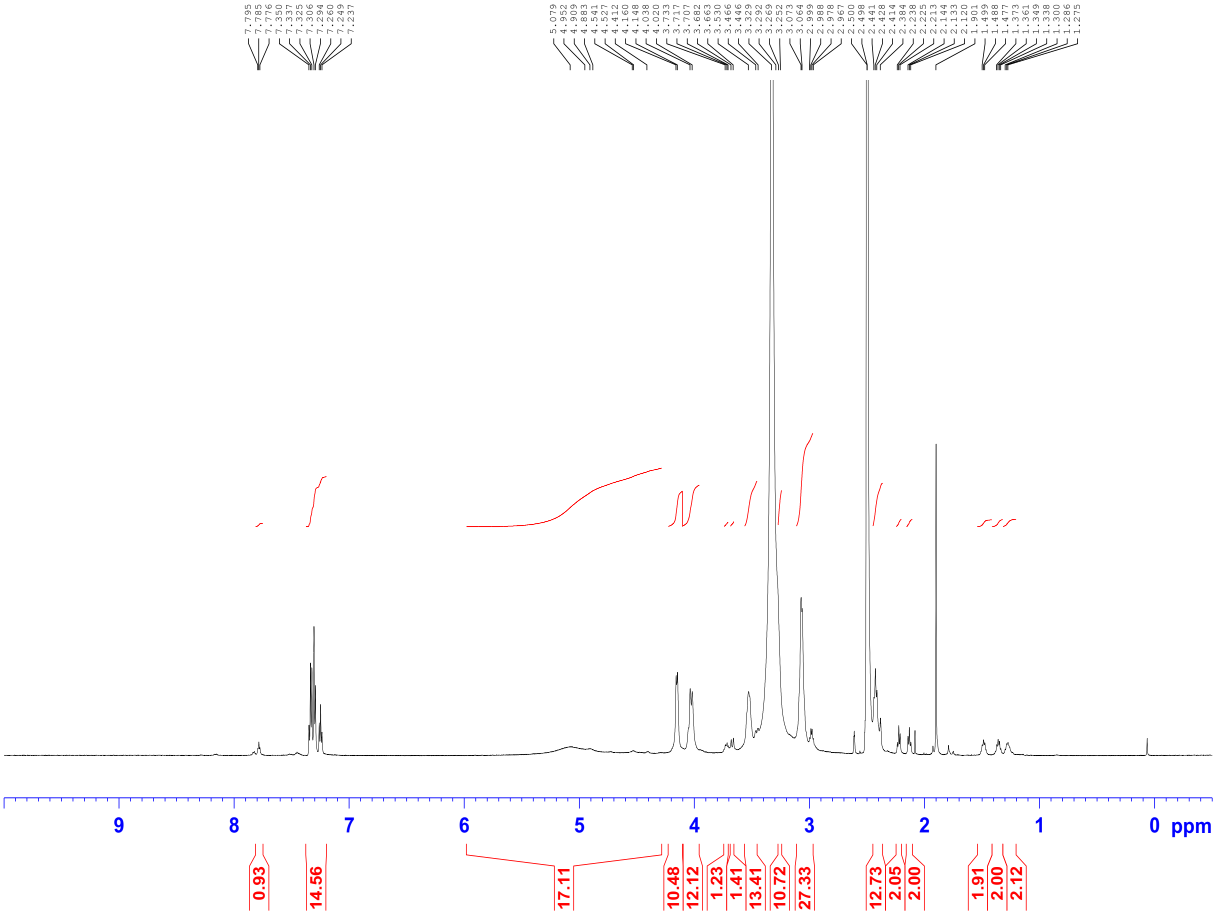

Figure S69. ^{1}H NMR spectrum of Compound 34

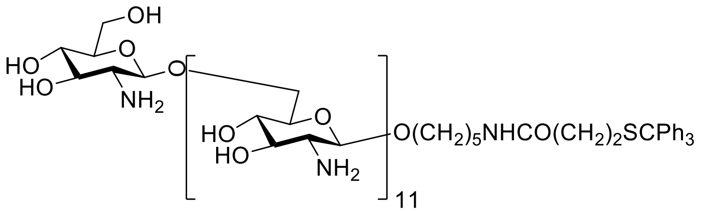

34

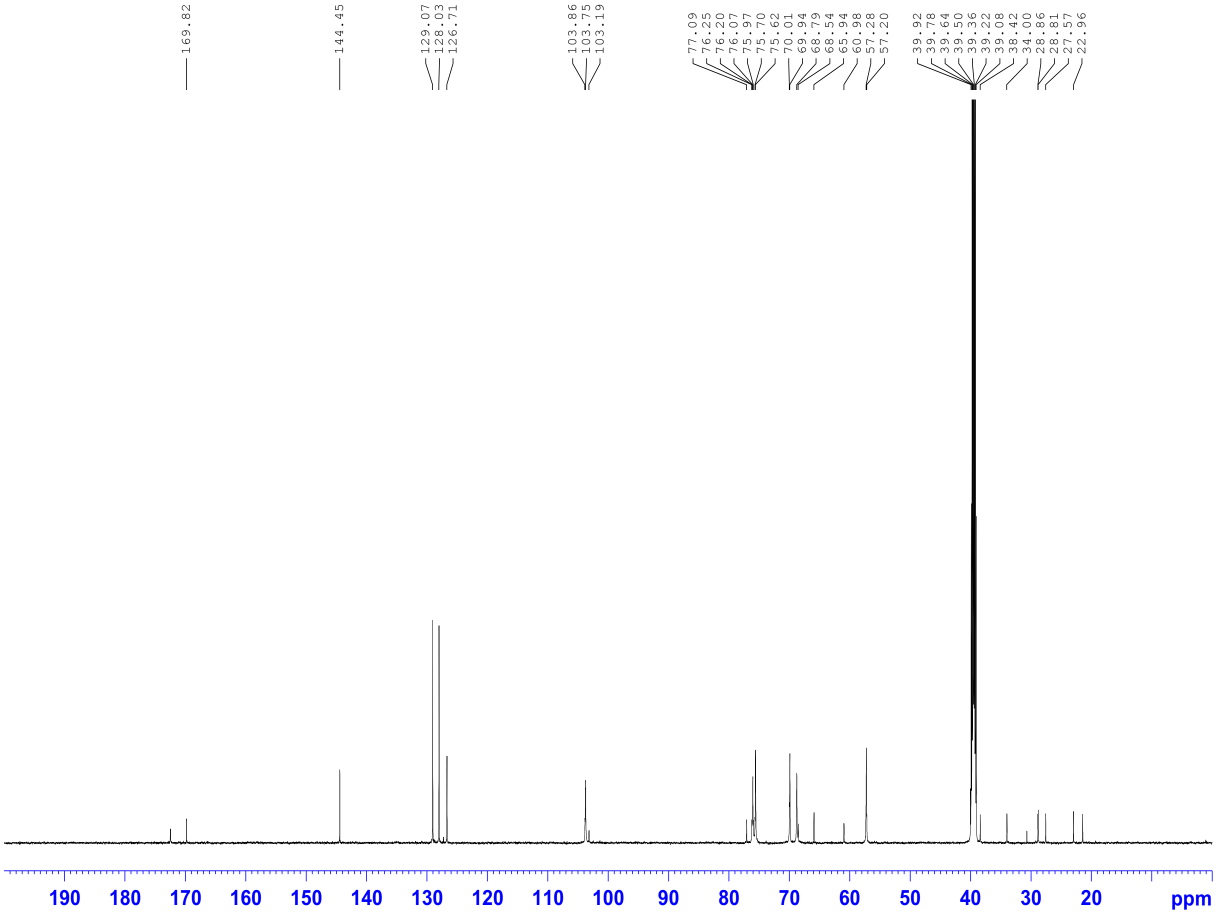

Figure S70. ^{13}C NMR spectrum of Compound 34

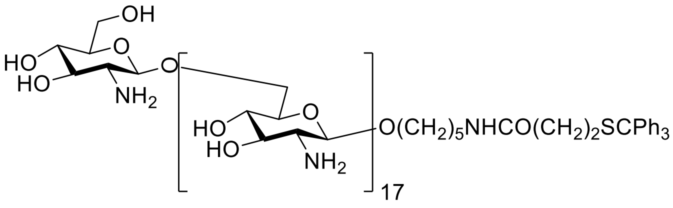

35

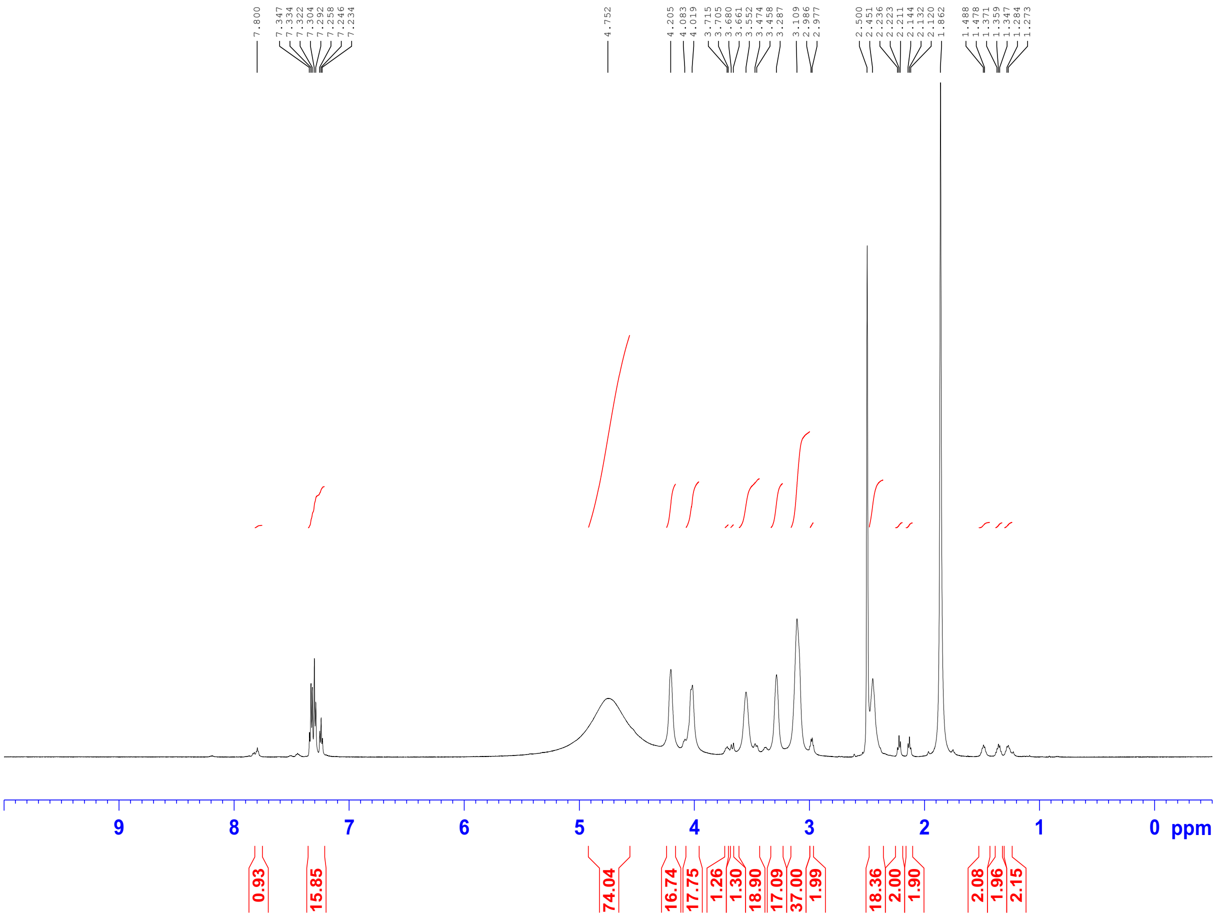

Figure S71. ^{1}H NMR spectrum of Compound 35

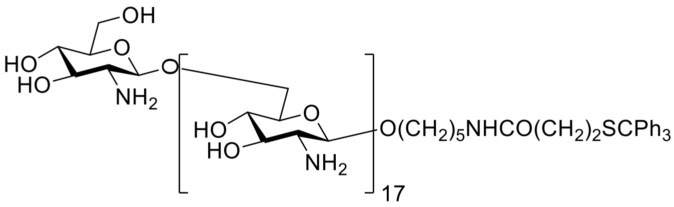

35

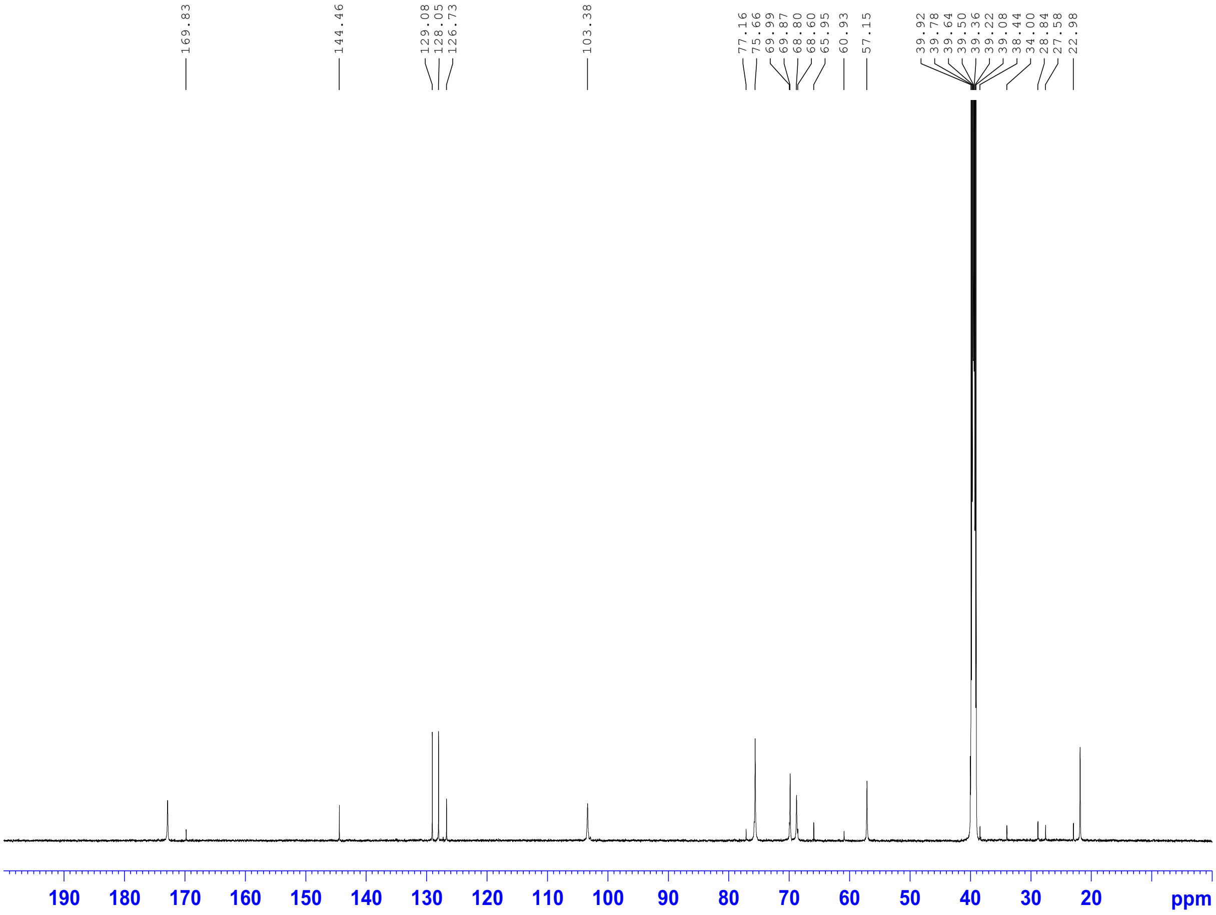

Figure S72. ^{13}C NMR spectrum of Compound 35

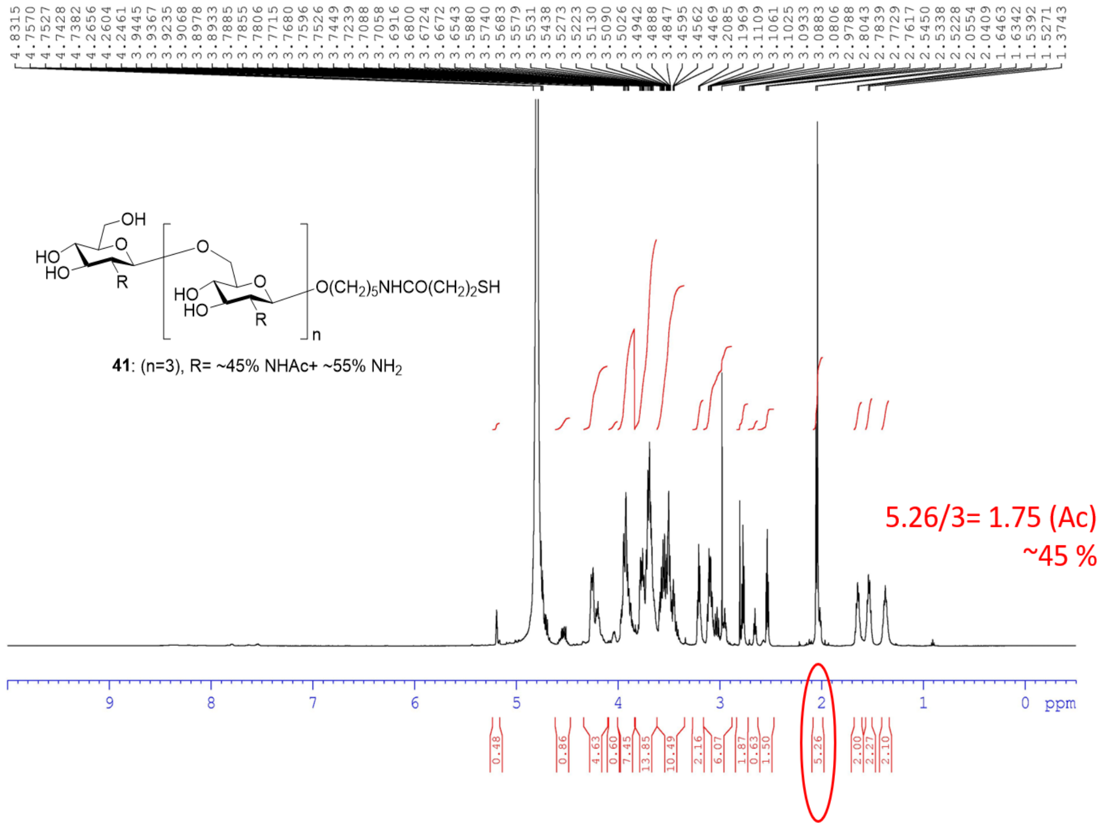

Figure S73. ^{1}H NMR spectrum of Compound 41

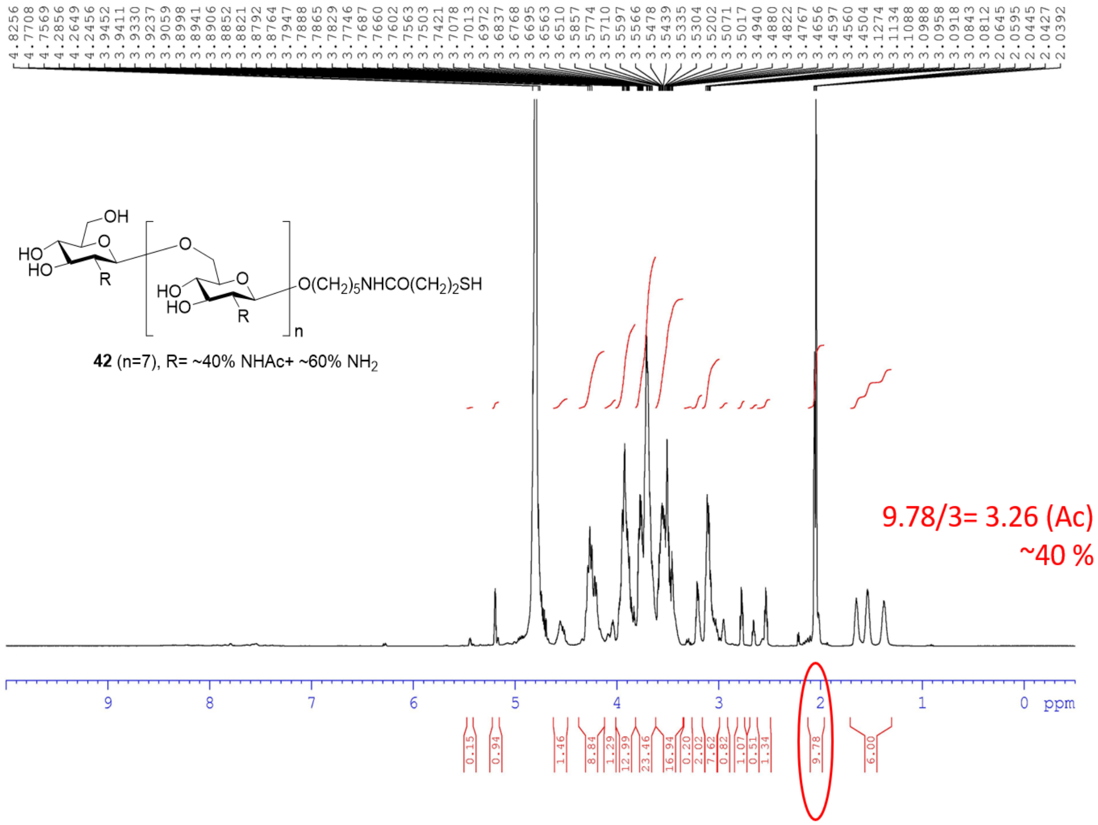

Figure S74. ^{1}H NMR spectrum of Compound 42

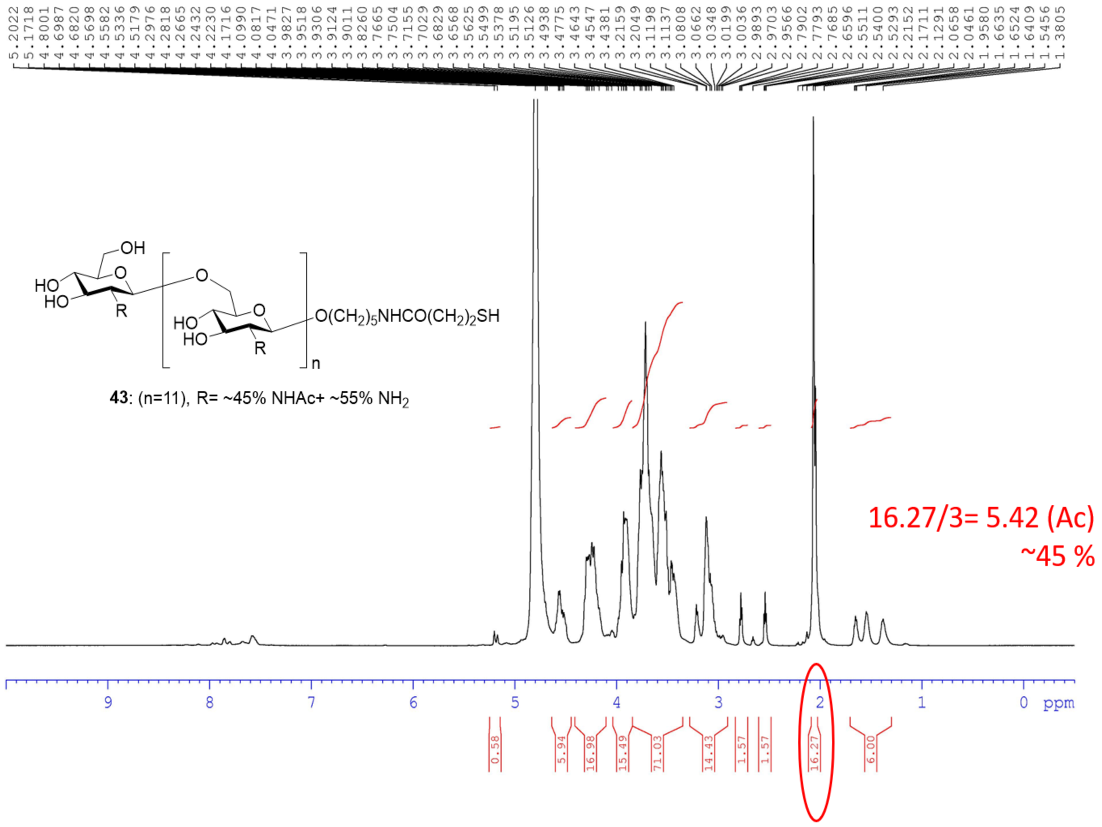

Figure S75. ^{1}H NMR spectrum of Compound 43

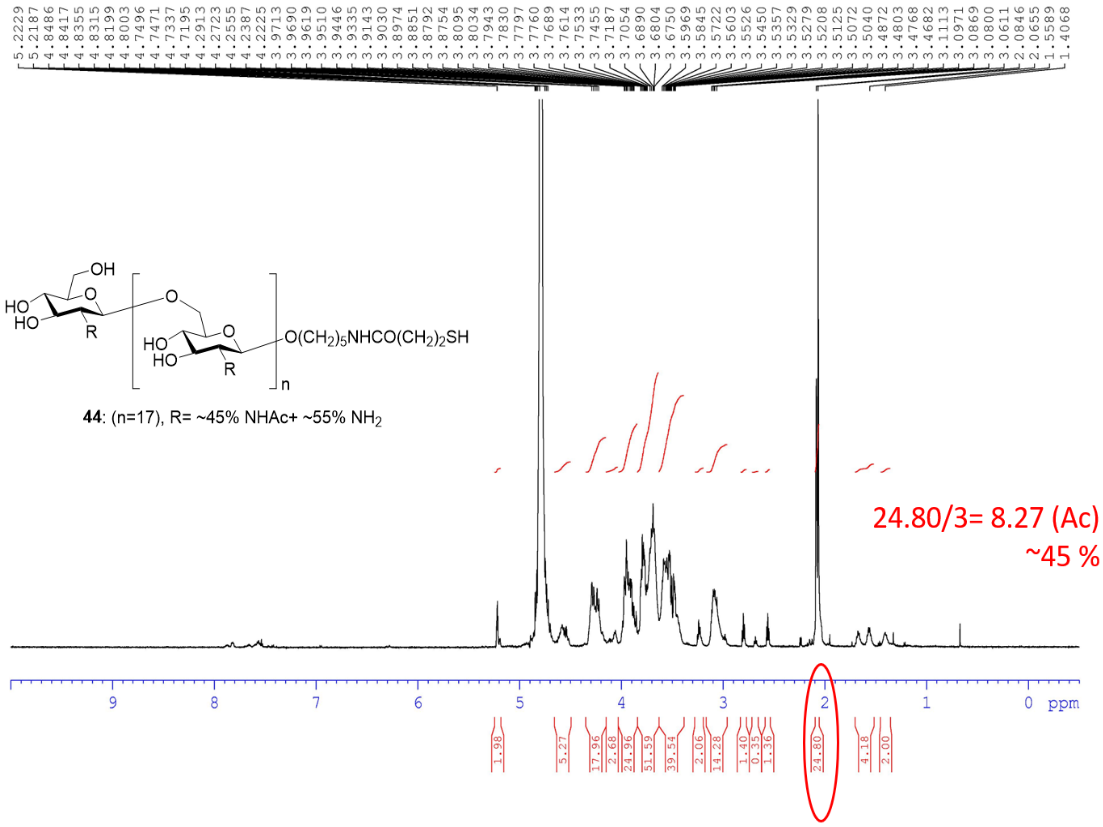

Figure S76. ^{1}H NMR spectrum of Compound 44

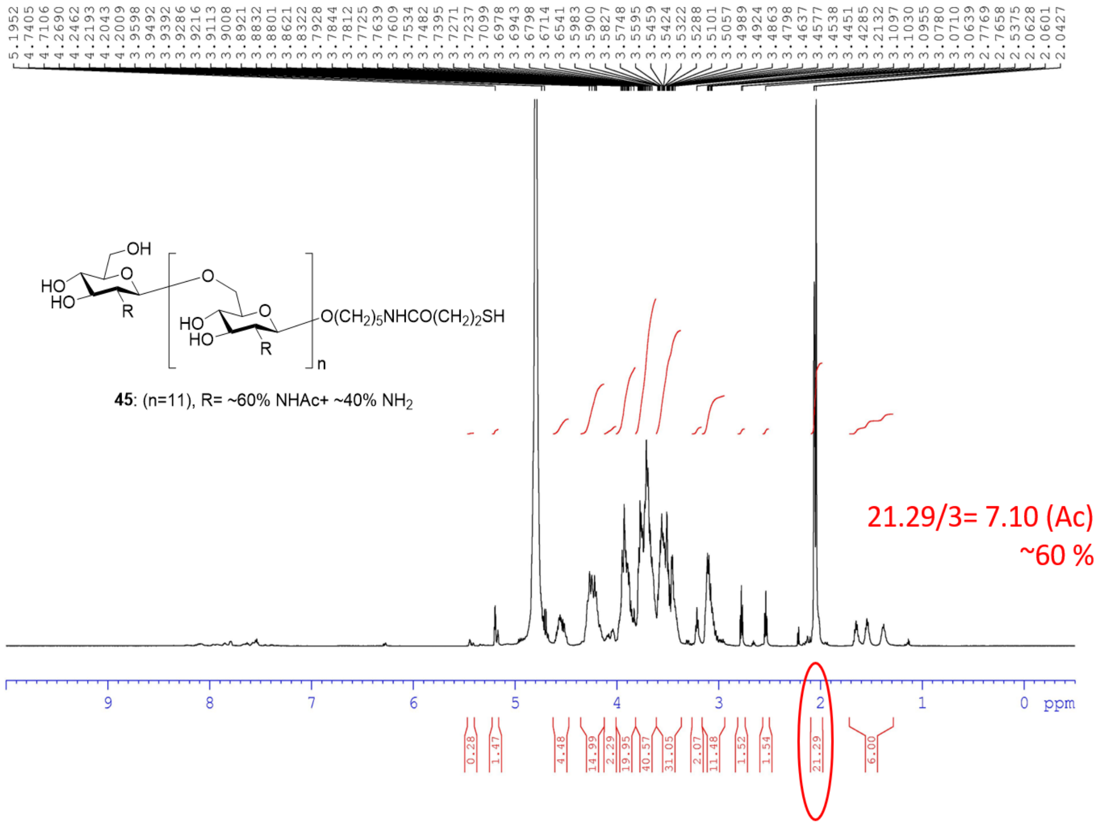

Figure S77. ¹H NMR spectrum of Compound 45

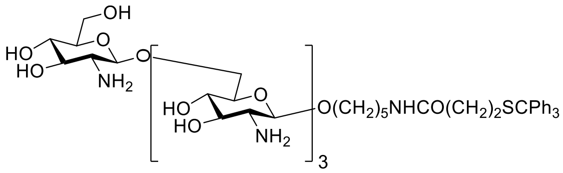

46

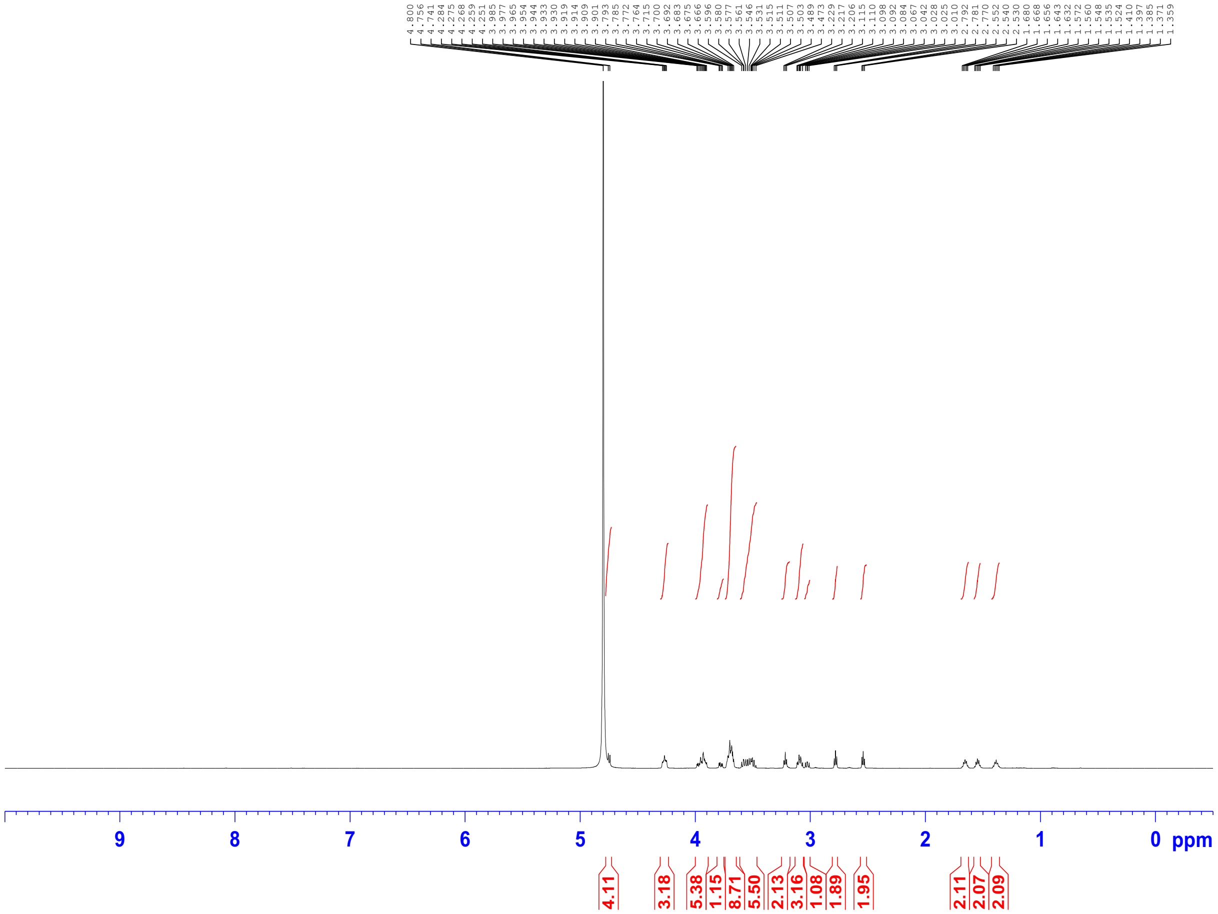

Figure S78. ^{1}H NMR spectrum of Compound 46

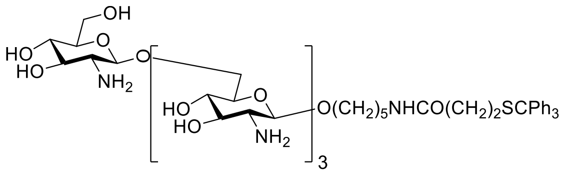

46

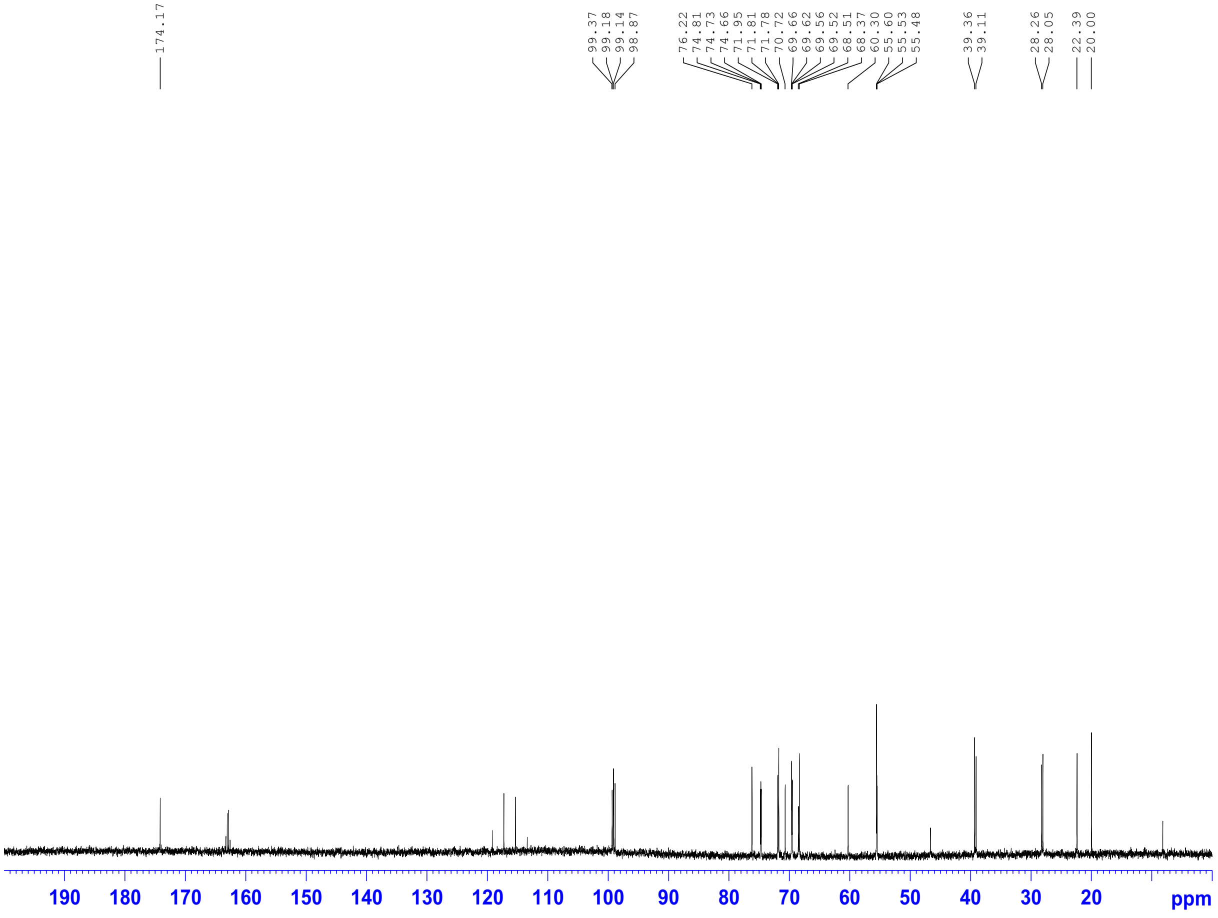

Figure S79. ^{13}C NMR spectrum of Compound 46

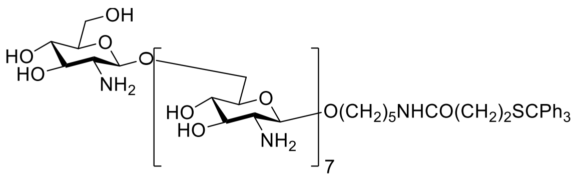

47

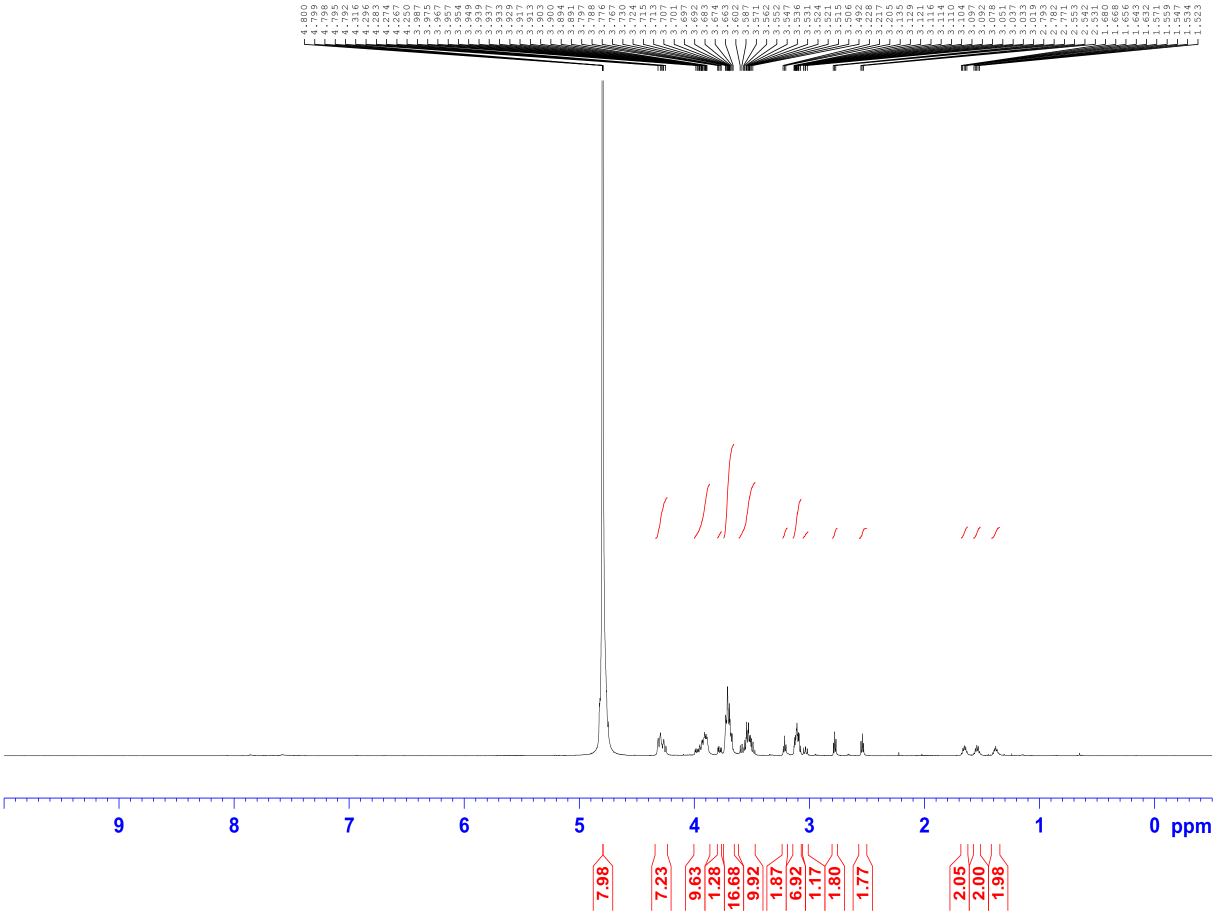

Figure S80. ^{1}H NMR spectrum of Compound 47

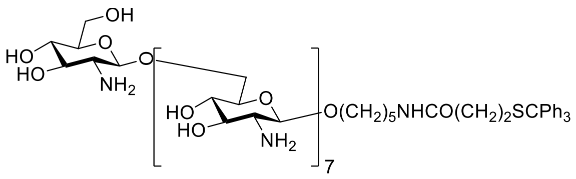

47

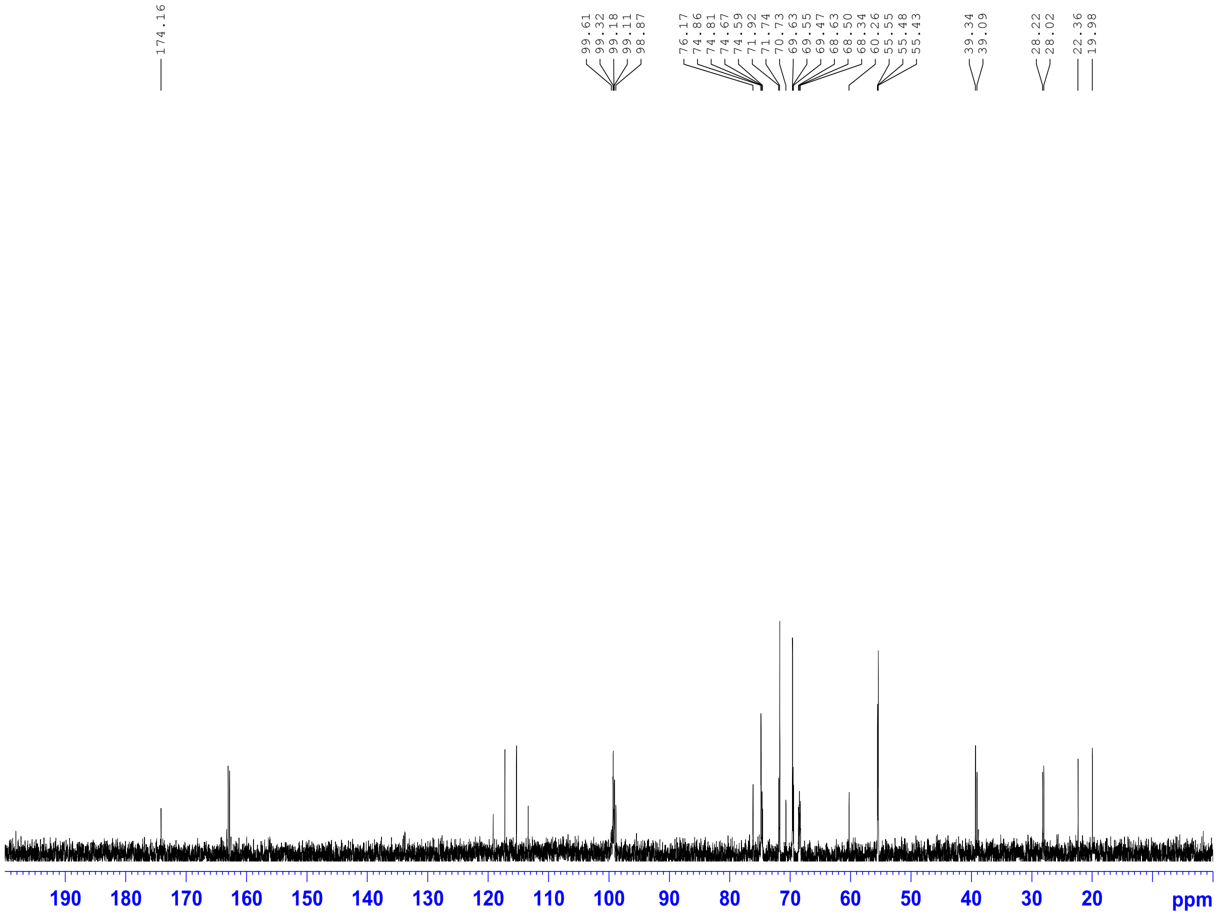

Figure S81. ^{13}C NMR spectrum of Compound 47

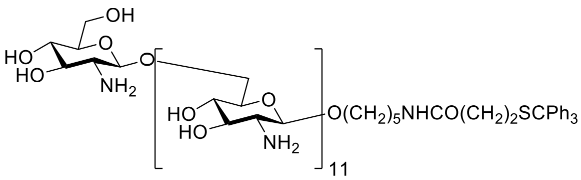

48

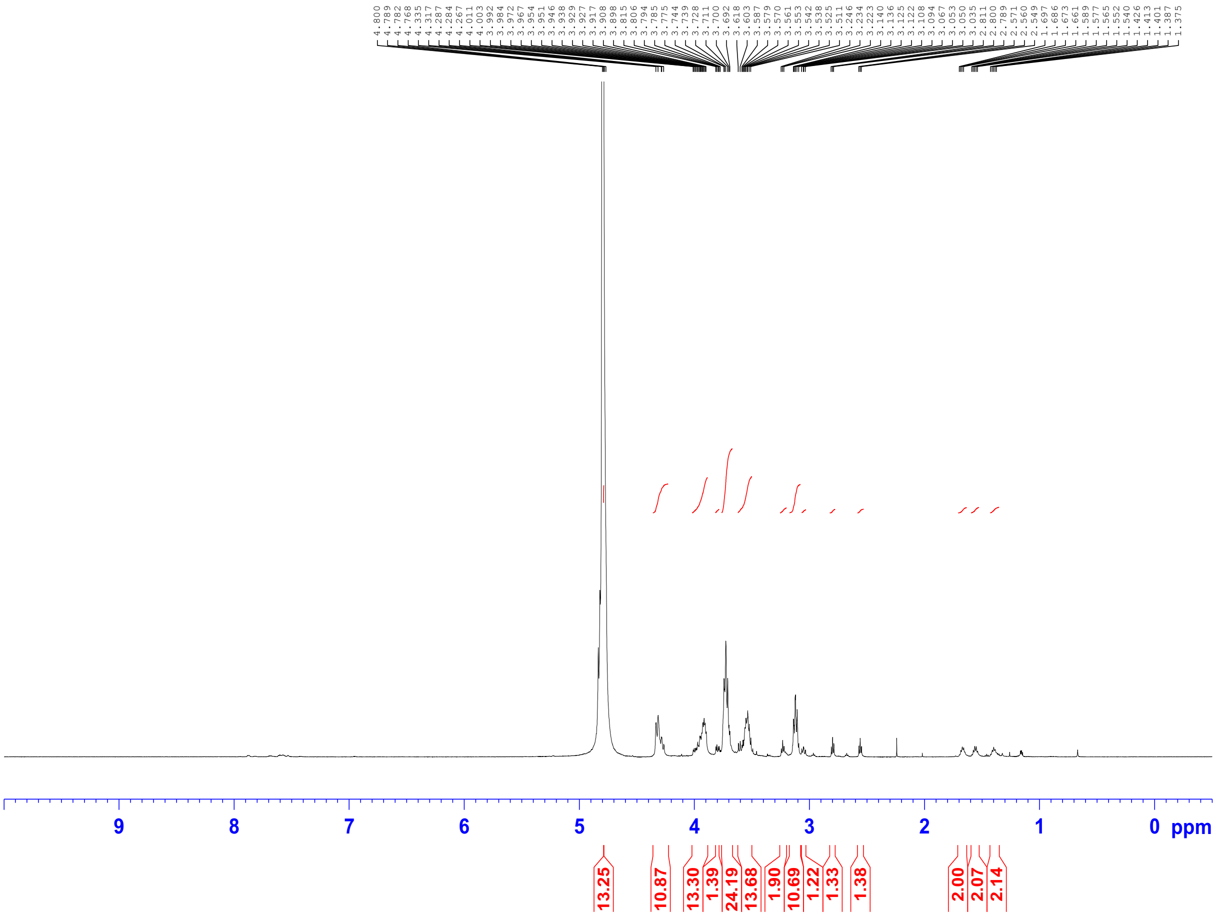

Figure S82. ^{1}H NMR spectrum of Compound 48

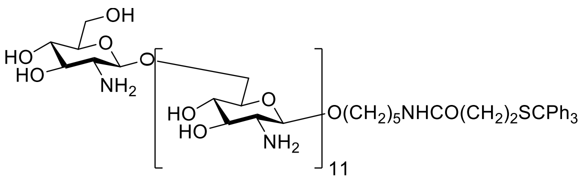

48

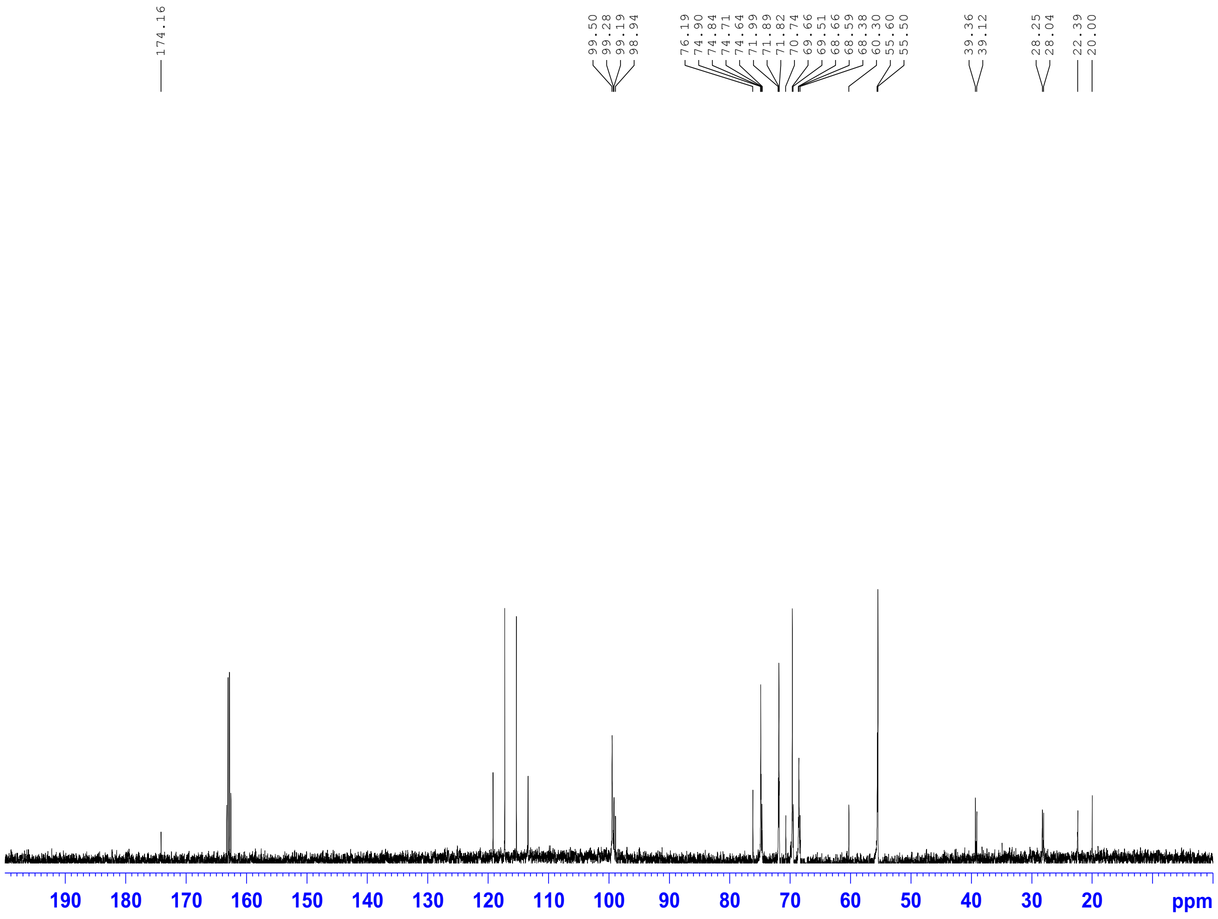

Figure S83. ^{13}C NMR spectrum of Compound 48

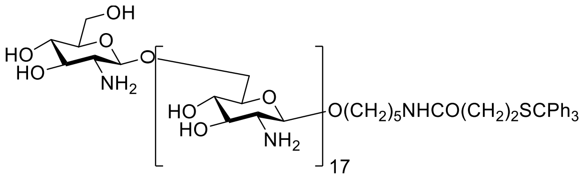

49

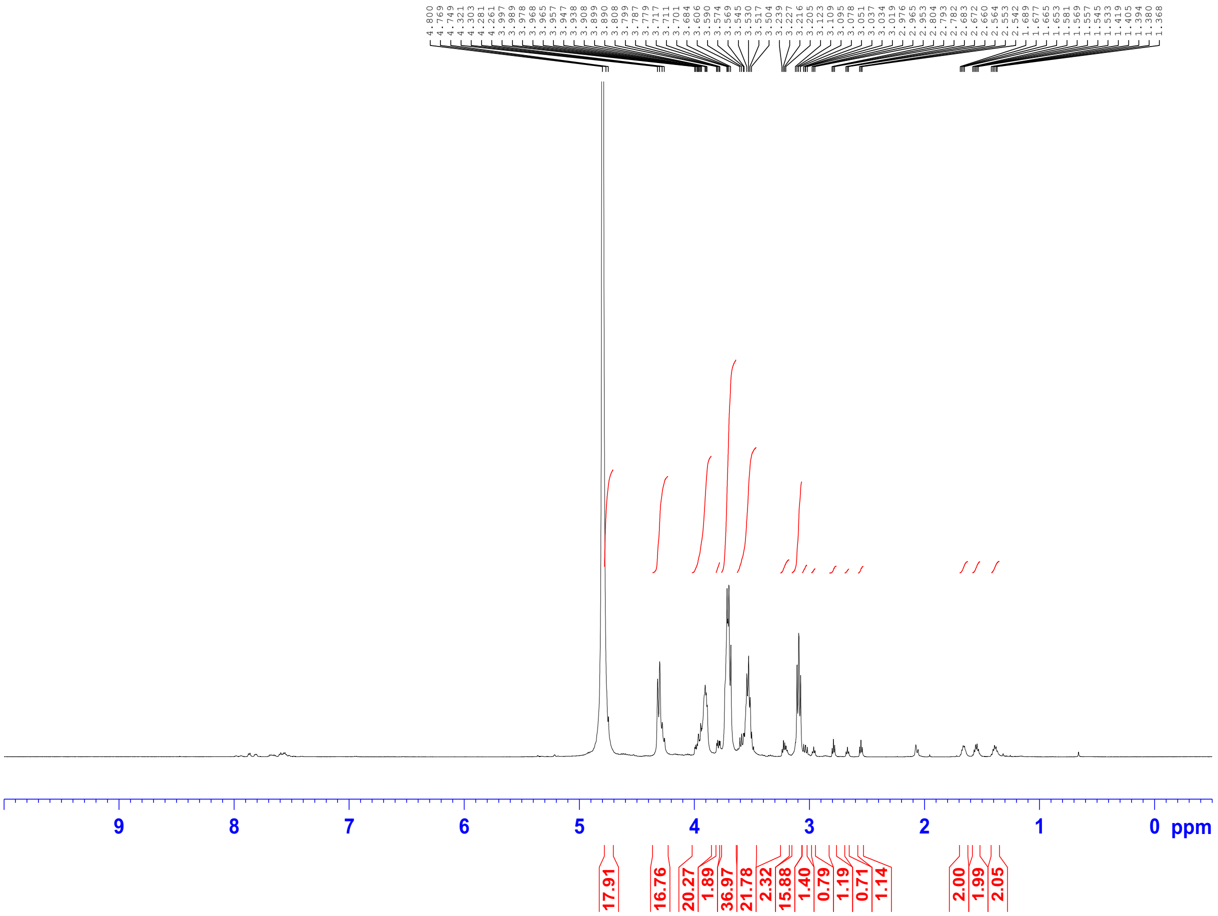

Figure S84. ¹H NMR spectrum of Compound 49

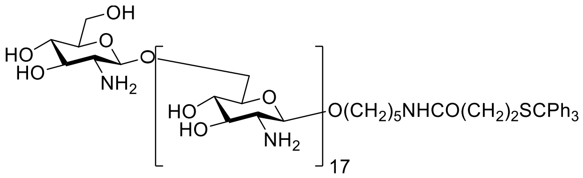

49

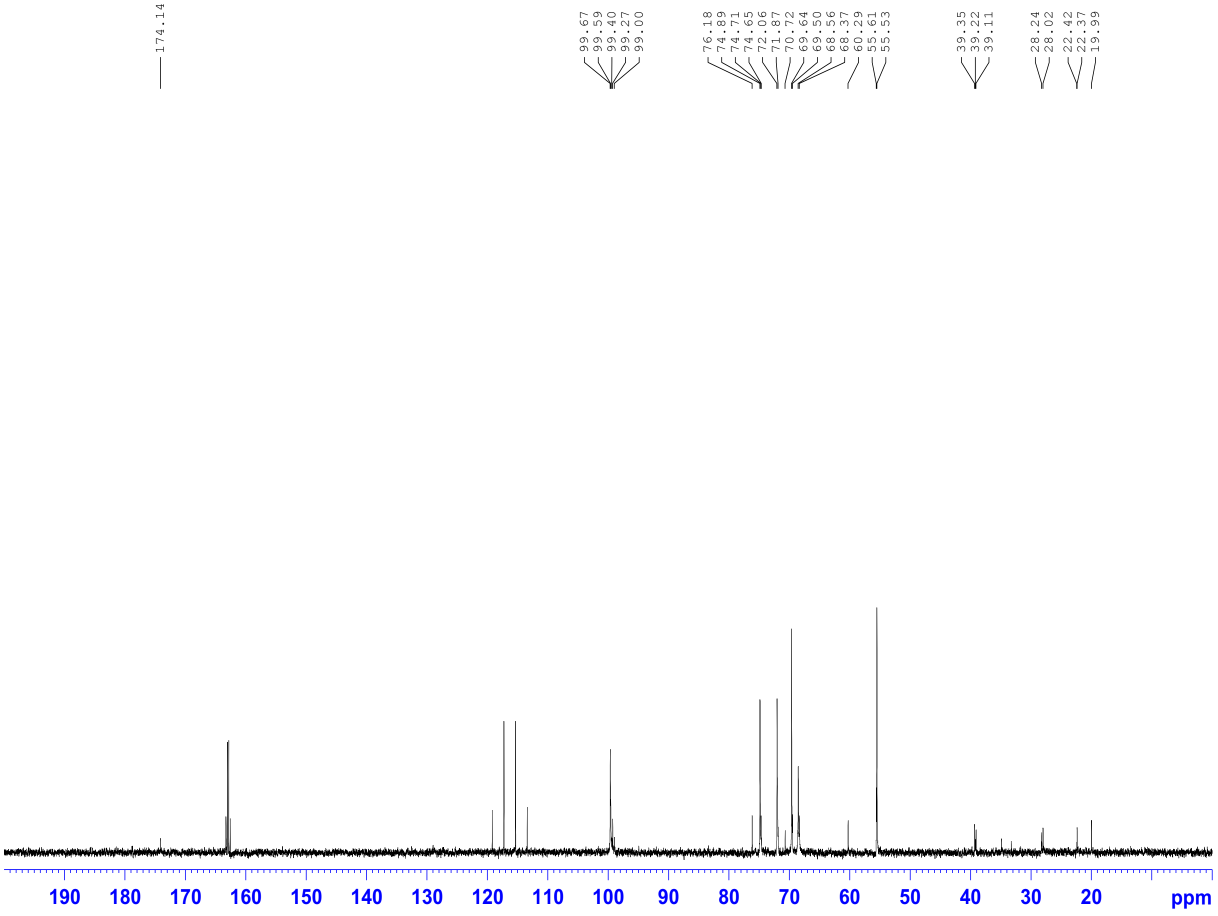

Figure S85. ^{13}C NMR spectrum of Compound 49

Table S2: Statistical significance and P values for dPNAG glycan binding assays with serum antibodies from infected patients

Serum 1_AB		
Tukey's multiple comparisons test	Significance	P Value
DP4_NH_2 vs. DP8_NH_2	****	<0.0001
DP4_NH_2 vs. DP12_NH_2	****	<0.0001
DP4_NH_2 vs. DP18_NH_2	****	<0.0001
DP4_NH_2 vs. DP4_45%NHAc	****	<0.0001
DP4_NH_2 vs. DP8_40%NHAc	****	<0.0001
DP4_NH_2 vs. DP12_45%NHAc	****	<0.0001
DP4_NH_2 vs. DP12_60%NHAc	****	<0.0001
DP4_NH_2 vs. DP18_45%NHAc	****	<0.0001
DP8_NH_2 vs. DP12_NH_2	****	<0.0001
DP8_NH_2 vs. DP18_NH_2	****	<0.0001
DP8_NH_2 vs. DP4_45%NHAc	****	<0.0001
DP8_NH_2 vs. DP8_40%NHAc	ns	0.9994
DP8_NH_2 vs. DP12_45%NHAc	****	<0.0001
DP8_NH_2 vs. DP12_60%NHAc	****	<0.0001
DP8_NH_2 vs. DP18_45%NHAc	****	<0.0001
DP12_NH_2 vs. DP18_NH_2	****	<0.0001
DP12_NH_2 vs. DP4_45%NHAc	****	<0.0001
DP12_NH_2 vs. DP8_40%NHAc	****	<0.0001
DP12_NH_2 vs. DP12_45%NHAc	****	<0.0001
DP12_NH_2 vs. DP12_60%NHAc	****	<0.0001
DP12_NH_2 vs. DP18_45%NHAc	****	<0.0001
DP18_NH_2 vs. DP4_45%NHAc	****	<0.0001
DP18_NH_2 vs. DP8_40%NHAc	****	<0.0001
DP18_NH_2 vs. DP12_45%NHAc	****	<0.0001
DP18_NH_2 vs. DP12_60%NHAc	****	<0.0001
DP18_NH_2 vs. DP18_45%NHAc	****	<0.0001
DP4_45%NHAc vs. DP8_40%NHAc	****	<0.0001
DP4_45%NHAc vs. DP12_45%NHAc	****	<0.0001
DP4_45%NHAc vs. DP12_60%NHAc	****	<0.0001
DP4_45%NHAc vs. DP18_45%NHAc	****	<0.0001
DP8_40%NHAc vs. DP12_45%NHAc	****	<0.0001
DP8_40%NHAc vs. DP12_60%NHAc	****	<0.0001

DP8_40%NHAc vs. DP18_45%NHAc	****	<0.0001
DP12_45%NHAc vs. DP12_60%NHAc	ns	0.1464
DP12_45%NHAc vs. DP18_45%NHAc	****	<0.0001
DP12_60%NHAc vs. DP18_45%NHAc	****	<0.0001

Serum 2_AB

Tukey's multiple comparisons test	Significance	P Value
DP4_NH_2 vs. DP8_NH_2	****	<0.0001
DP4_NH_2 vs. DP12_NH_2	****	<0.0001
DP4_NH_2 vs. DP18_NH_2	****	<0.0001
DP4_NH_2 vs. DP4_45%NHAc	****	<0.0001
DP4_NH_2 vs. DP8_40%NHAc	ns	0.9897
DP4_NH_2 vs. DP12_45%NHAc	****	<0.0001
DP4_NH_2 vs. DP12_60%NHAc	****	<0.0001
DP4_NH_2 vs. DP18_45%NHAc	****	<0.0001
DP8_NH_2 vs. DP12_NH_2	****	<0.0001
DP8_NH_2 vs. DP18_NH_2	****	<0.0001
DP8_NH_2 vs. DP4_45%NHAc	****	<0.0001
DP8_NH_2 vs. DP8_40%NHAc	****	<0.0001
DP8_NH_2 vs. DP12_45%NHAc	****	<0.0001
DP8_NH_2 vs. DP12_60%NHAc	****	<0.0001
DP8_NH_2 vs. DP18_45%NHAc	****	<0.0001
DP12_NH_2 vs. DP18_NH_2	****	<0.0001
DP12_NH_2 vs. DP4_45%NHAc	****	<0.0001
DP12_NH_2 vs. DP8_40%NHAc	****	<0.0001
DP12_NH_2 vs. DP12_45%NHAc	****	<0.0001
DP12_NH_2 vs. DP12_60%NHAc	****	<0.0001
DP12_NH_2 vs. DP18_45%NHAc	****	<0.0001
DP18_NH_2 vs. DP4_45%NHAc	****	<0.0001
DP18_NH_2 vs. DP8_40%NHAc	****	<0.0001
DP18_NH_2 vs. DP12_45%NHAc	****	<0.0001
DP18_NH_2 vs. DP12_60%NHAc	****	<0.0001
DP18_NH_2 vs. DP18_45%NHAc	****	<0.0001
DP4_45%NHAc vs. DP8_40%NHAc	****	<0.0001
DP4_45%NHAc vs. DP12_45%NHAc	****	<0.0001
DP4_45%NHAc vs. DP12_60%NHAc	****	<0.0001
DP4_45%NHAc vs. DP18_45%NHAc	****	<0.0001

DP8_40%NHAc vs. DP12_45%NHAc	****	<0.0001
DP8_40%NHAc vs. DP12_60%NHAc	****	<0.0001
DP8_40%NHAc vs. DP18_45%NHAc	****	<0.0001
DP12_45%NHAc vs. DP12_60%NHAc	****	<0.0001
DP12_45%NHAc vs. DP18_45%NHAc	ns	0.9486
DP12_60%NHAc vs. DP18_45%NHAc	****	<0.0001

Serum 58_19A

Tukey's multiple comparisons test	Significance	P Value
DP4_NH_2 vs. DP8_NH_2	****	<0.0001
DP4_NH_2 vs. DP12_NH_2	****	<0.0001
DP4_NH_2 vs. DP18_NH_2	****	<0.0001
DP4_NH_2 vs. DP4_45%Ac	****	<0.0001
DP4_NH_2 vs. DP8_40%Ac	ns	0.5816
DP4_NH_2 vs. DP12_45%Ac	****	<0.0001
DP4_NH_2 vs. DP12_60%Ac	****	<0.0001
DP4_NH_2 vs. DP18_45%Ac	****	<0.0001
DP8_NH_2 vs. DP12_NH_2	****	<0.0001
DP8_NH_2 vs. DP18_NH_2	****	<0.0001
DP8_NH_2 vs. DP4_45%Ac	****	<0.0001
DP8_NH_2 vs. DP8_40%Ac	****	<0.0001
DP8_NH_2 vs. DP12_45%Ac	****	<0.0001
DP8_NH_2 vs. DP12_60%Ac	****	<0.0001
DP8_NH_2 vs. DP18_45%Ac	****	<0.0001
DP12_NH_2 vs. DP18_NH_2	****	<0.0001
DP12_NH_2 vs. DP4_45%Ac	****	<0.0001
DP12_NH_2 vs. DP8_40%Ac	****	<0.0001
DP12_NH_2 vs. DP12_45%Ac	****	<0.0001
DP12_NH_2 vs. DP12_60%Ac	****	<0.0001
DP12_NH_2 vs. DP18_45%Ac	****	<0.0001
DP18_NH_2 vs. DP4_45%Ac	****	<0.0001
DP18_NH_2 vs. DP8_40%Ac	****	<0.0001
DP18_NH_2 vs. DP12_45%Ac	****	<0.0001
DP18_NH_2 vs. DP12_60%Ac	****	<0.0001
DP18_NH_2 vs. DP18_45%Ac	****	<0.0001
DP4_45%Ac vs. DP8_40%Ac	****	<0.0001
DP4_45%Ac vs. DP12_45%Ac	****	<0.0001

DP4_45%Ac vs. DP12_60%Ac	****	<0.0001
DP4_45%Ac vs. DP18_45%Ac	****	<0.0001
DP8_40%Ac vs. DP12_45%Ac	****	<0.0001
DP8_40%Ac vs. DP12_60%Ac	****	<0.0001
DP8_40%Ac vs. DP18_45%Ac	****	<0.0001
DP12_45%Ac vs. DP12_60%Ac	****	<0.0001
DP12_45%Ac vs. DP18_45%Ac	****	<0.0001
DP12_60%Ac vs. DP18_45%Ac	****	<0.0001

Serum 367_19A

Tukey's multiple comparisons test	Significance	P Value
DP4_NH_2 vs. DP8_NH_2	****	<0.0001
DP4_NH_2 vs. DP12_NH_2	****	<0.0001
DP4_NH_2 vs. DP18_NH_2	****	<0.0001
DP4_NH_2 vs. DP4_45%Ac	****	<0.0001
DP4_NH_2 vs. DP8_40%Ac	****	<0.0001
DP4_NH_2 vs. DP12_45%Ac	****	<0.0001
DP4_NH_2 vs. DP12_60%Ac	****	<0.0001
DP4_NH_2 vs. DP18_45%Ac	****	<0.0001
DP8_NH_2 vs. DP12_NH_2	****	<0.0001
DP8_NH_2 vs. DP18_NH_2	****	<0.0001
DP8_NH_2 vs. DP4_45%Ac	****	<0.0001
DP8_NH_2 vs. DP8_40%Ac	ns	>0.9999
DP8_NH_2 vs. DP12_45%Ac	****	<0.0001
DP8_NH_2 vs. DP12_60%Ac	****	<0.0001
DP8_NH_2 vs. DP18_45%Ac	****	<0.0001
DP12_NH_2 vs. DP18_NH_2	****	<0.0001
DP12_NH_2 vs. DP4_45%Ac	****	<0.0001
DP12_NH_2 vs. DP8_40%Ac	****	<0.0001
DP12_NH_2 vs. DP12_45%Ac	****	<0.0001
DP12_NH_2 vs. DP12_60%Ac	****	<0.0001
DP12_NH_2 vs. DP18_45%Ac	****	<0.0001
DP18_NH_2 vs. DP4_45%Ac	****	<0.0001
DP18_NH_2 vs. DP8_40%Ac	****	<0.0001
DP18_NH_2 vs. DP12_45%Ac	****	<0.0001
DP18_NH_2 vs. DP12_60%Ac	****	<0.0001
DP18_NH_2 vs. DP18_45%Ac	****	<0.0001

DP4_45%Ac vs. DP8_40%Ac	****	<0.0001
DP4_45%Ac vs. DP12_45%Ac	****	<0.0001
DP4_45%Ac vs. DP12_60%Ac	****	<0.0001
DP4_45%Ac vs. DP18_45%Ac	****	<0.0001
DP8_40%Ac vs. DP12_45%Ac	****	<0.0001
DP8_40%Ac vs. DP12_60%Ac	****	<0.0001
DP8_40%Ac vs. DP18_45%Ac	****	<0.0001
DP12_45%Ac vs. DP12_60%Ac	****	<0.0001
DP12_45%Ac vs. DP18_45%Ac	****	<0.0001
DP12_60%Ac vs. DP18_45%Ac	****	<0.0001

Serum 067_19F

Tukey's multiple comparisons test	Significance	P Value
DP4_NH_2 vs. DP8_NH_2	ns	0.6338
DP4_NH_2 vs. DP12_NH_2	****	<0.0001
DP4_NH_2 vs. DP18_NH_2	****	<0.0001
DP4_NH_2 vs. DP4_45%Ac	****	<0.0001
DP4_NH_2 vs. DP8_40%Ac	****	<0.0001
DP4_NH_2 vs. DP12_45%Ac	****	<0.0001
DP4_NH_2 vs. DP12_60%Ac	**	0.0017
DP4_NH_2 vs. DP18_45%Ac	****	<0.0001
DP8_NH_2 vs. DP12_NH_2	***	0.0002
DP8_NH_2 vs. DP18_NH_2	****	<0.0001
DP8_NH_2 vs. DP4_45%Ac	****	<0.0001
DP8_NH_2 vs. DP8_40%Ac	****	<0.0001
DP8_NH_2 vs. DP12_45%Ac	****	<0.0001
DP8_NH_2 vs. DP12_60%Ac	****	<0.0001
DP8_NH_2 vs. DP18_45%Ac	****	<0.0001
DP12_NH_2 vs. DP18_NH_2	ns	0.0757
DP12_NH_2 vs. DP4_45%Ac	****	<0.0001
DP12_NH_2 vs. DP8_40%Ac	ns	0.9997
DP12_NH_2 vs. DP12_45%Ac	****	<0.0001
DP12_NH_2 vs. DP12_60%Ac	****	<0.0001
DP12_NH_2 vs. DP18_45%Ac	****	<0.0001
DP18_NH_2 vs. DP4_45%Ac	**	0.0038
DP18_NH_2 vs. DP8_40%Ac	ns	0.2729
DP18_NH_2 vs. DP12_45%Ac	****	<0.0001

DP18_NH_2 vs. DP12_60%Ac	****	<0.0001
DP18_NH_2 vs. DP18_45%Ac	****	<0.0001
DP4_45%Ac vs. DP8_40%Ac	****	<0.0001
DP4_45%Ac vs. DP12_45%Ac	****	<0.0001
DP4_45%Ac vs. DP12_60%Ac	****	<0.0001
DP4_45%Ac vs. DP18_45%Ac	****	<0.0001
DP8_40%Ac vs. DP12_45%Ac	****	<0.0001
DP8_40%Ac vs. DP12_60%Ac	****	<0.0001
DP8_40%Ac vs. DP18_45%Ac	****	<0.0001
DP12_45%Ac vs. DP12_60%Ac	****	<0.0001
DP12_45%Ac vs. DP18_45%Ac	****	<0.0001
DP12_60%Ac vs. DP18_45%Ac	****	<0.0001

Serum 30_23F

Tukey's multiple comparisons test	Significance	P Value
DP4_NH_2 vs. DP8_NH_2	****	<0.0001
DP4_NH_2 vs. DP12_NH_2	****	<0.0001
DP4_NH_2 vs. DP18_NH_2	****	<0.0001
DP4_NH_2 vs. DP4_45%Ac	****	<0.0001
DP4_NH_2 vs. DP8_40%Ac	ns	0.6697
DP4_NH_2 vs. DP12_45%Ac	****	<0.0001
DP4_NH_2 vs. DP12_60%Ac	****	<0.0001
DP4_NH_2 vs. DP18_45%Ac	****	<0.0001
DP8_NH_2 vs. DP12_NH_2	****	<0.0001
DP8_NH_2 vs. DP18_NH_2	****	<0.0001
DP8_NH_2 vs. DP4_45%Ac	****	<0.0001
DP8_NH_2 vs. DP8_40%Ac	****	<0.0001
DP8_NH_2 vs. DP12_45%Ac	****	<0.0001
DP8_NH_2 vs. DP12_60%Ac	****	<0.0001
DP8_NH_2 vs. DP18_45%Ac	****	<0.0001
DP12_NH_2 vs. DP18_NH_2	***	0.0001
DP12_NH_2 vs. DP4_45%Ac	****	<0.0001
DP12_NH_2 vs. DP8_40%Ac	****	<0.0001
DP12_NH_2 vs. DP12_45%Ac	****	<0.0001
DP12_NH_2 vs. DP12_60%Ac	****	<0.0001
DP12_NH_2 vs. DP18_45%Ac	****	<0.0001
DP18_NH_2 vs. DP4_45%Ac	*	0.044

$DP18\_NH_2$ vs. DP8_40%Ac	****	<0.0001
$DP18\_NH_2$ vs. DP12_45%Ac	****	<0.0001
$DP18\_NH_2$ vs. DP12_60%Ac	****	<0.0001
$DP18\_NH_2$ vs. DP18_45%Ac	****	<0.0001
DP4_45%Ac vs. DP8_40%Ac	****	<0.0001
DP4_45%Ac vs. DP12_45%Ac	****	<0.0001
DP4_45%Ac vs. DP12_60%Ac	****	<0.0001
DP4_45%Ac vs. DP18_45%Ac	****	<0.0001
DP8_40%Ac vs. DP12_45%Ac	****	<0.0001
DP8_40%Ac vs. DP12_60%Ac	****	<0.0001
DP8_40%Ac vs. DP18_45%Ac	****	<0.0001
DP12_45%Ac vs. DP12_60%Ac	****	<0.0001
DP12_45%Ac vs. DP18_45%Ac	****	<0.0001
DP12_60%Ac vs. DP18_45%Ac	****	<0.0001

Serum BK

Tukey's multiple comparisons test	Significance	P Value
$DP4\_NH_2$ vs. $DP8\_NH_2$	ns	>0.9999
$DP4\_NH_2$ vs. $DP12\_NH_2$	ns	>0.9999
$DP4\_NH_2$ vs. $DP18\_NH_2$	ns	>0.9999
$DP4\_NH_2$ vs. DP4_45%Ac	ns	>0.9999
$DP4\_NH_2$ vs. DP8_40%Ac	ns	0.5267
$DP4\_NH_2$ vs. DP12_45%Ac	ns	>0.9999
$DP4\_NH_2$ vs. DP12_60%Ac	ns	>0.9999
$DP4\_NH_2$ vs. DP18_45%Ac	ns	>0.9999
$DP8\_NH_2$ vs. $DP12\_NH_2$	ns	>0.9999
$DP8\_NH_2$ vs. $DP18\_NH_2$	ns	>0.9999
$DP8\_NH_2$ vs. DP4_45%Ac	ns	>0.9999
$DP8\_NH_2$ vs. DP8_40%Ac	ns	0.6919
$DP8\_NH_2$ vs. DP12_45%Ac	ns	>0.9999
$DP8\_NH_2$ vs. DP12_60%Ac	ns	>0.9999
$DP8\_NH_2$ vs. DP18_45%Ac	ns	>0.9999
$DP12\_NH_2$ vs. $DP18\_NH_2$	ns	>0.9999
$DP12\_NH_2$ vs. DP4_45%Ac	ns	>0.9999
$DP12\_NH_2$ vs. DP8_40%Ac	ns	0.5845
$DP12\_NH_2$ vs. DP12_45%Ac	ns	>0.9999
$DP12\_NH_2$ vs. DP12_60%Ac	ns	>0.9999

DP12_NH_2 vs. DP18_45%Ac	ns	>0.9999
DP18_NH_2 vs. DP4_45%Ac	ns	>0.9999
DP18_NH_2 vs. DP8_40%Ac	ns	0.6398
DP18_NH_2 vs. DP12_45%Ac	ns	>0.9999
DP18_NH_2 vs. DP12_60%Ac	ns	>0.9999
DP18_NH_2 vs. DP18_45%Ac	ns	>0.9999
DP4_45%Ac vs. DP8_40%Ac	ns	0.5571
DP4_45%Ac vs. DP12_45%Ac	ns	>0.9999
DP4_45%Ac vs. DP12_60%Ac	ns	>0.9999
DP4_45%Ac vs. DP18_45%Ac	ns	>0.9999
DP8_40%Ac vs. DP12_45%Ac	ns	0.5952
DP8_40%Ac vs. DP12_60%Ac	ns	0.3739
DP8_40%Ac vs. DP18_45%Ac	ns	0.4232
DP12_45%Ac vs. DP12_60%Ac	ns	>0.9999
DP12_45%Ac vs. DP18_45%Ac	ns	>0.9999
DP12_60%Ac vs. DP18_45%Ac	ns	>0.9999

Statistical analysis was performed with one-way ANOVA followed by Tukey's multiple comparison test using GraphPad Prism version 10.4.0 for macOS.

Table S3: Statistical significance and P values for dPNAG glycan binding assays with sera from mice immunized with dPNAG-CRM197 conjugates

Glycan microarray assay with 1000-fold diluted sera		
Tukey's multiple comparisons test	Significance	P Value
DP4_NH_2 glycan binding		
PNAG-4mer-NH_2 vs. PNAG-8mer-NH_2	****	<0.0001
PNAG-4mer-NH_2 vs. PNAG-12mer-NH_2	****	<0.0001
PNAG-4mer-NH_2 vs. PNAG-18mer-NH_2	****	<0.0001
PNAG-4mer-NH_2 vs. BK	****	<0.0001
PNAG-4mer-NH_2 vs. PNAG-4mer-45%Ac	****	<0.0001
PNAG-4mer-NH_2 vs. PNAG-8mer-40%Ac	****	<0.0001
PNAG-4mer-NH_2 vs. PNAG-12mer-45%Ac	****	<0.0001
PNAG-4mer-NH_2 vs. PNAG-12mer-60%Ac	ns	>0.9999
PNAG-4mer-NH_2 vs. PNAG-18mer-45%Ac	****	<0.0001
PNAG-8mer-NH_2 vs. PNAG-12mer-NH_2	****	<0.0001
PNAG-8mer-NH_2 vs. PNAG-18mer-NH_2	****	<0.0001
PNAG-8mer-NH_2 vs. BK	****	<0.0001
PNAG-8mer-NH_2 vs. PNAG-4mer-45%Ac	****	<0.0001
PNAG-8mer-NH_2 vs. PNAG-8mer-40%Ac	****	<0.0001
PNAG-8mer-NH_2 vs. PNAG-12mer-45%Ac	****	<0.0001
PNAG-8mer-NH_2 vs. PNAG-12mer-60%Ac	****	<0.0001
PNAG-8mer-NH_2 vs. PNAG-18mer-45%Ac	****	<0.0001
PNAG-12mer-NH_2 vs. PNAG-18mer-NH_2	****	<0.0001
PNAG-12mer-NH_2 vs. BK	****	<0.0001
PNAG-12mer-NH_2 vs. PNAG-4mer-45%Ac	****	<0.0001
PNAG-12mer-NH_2 vs. PNAG-8mer-40%Ac	****	<0.0001
PNAG-12mer-NH_2 vs. PNAG-12mer-45%Ac	****	<0.0001
PNAG-12mer-NH_2 vs. PNAG-12mer-60%Ac	****	<0.0001
PNAG-12mer-NH_2 vs. PNAG-18mer-45%Ac	****	<0.0001
PNAG-18mer-NH_2 vs. BK	****	<0.0001
PNAG-18mer-NH_2 vs. PNAG-4mer-45%Ac	****	<0.0001
PNAG-18mer-NH_2 vs. PNAG-8mer-40%Ac	****	<0.0001
PNAG-18mer-NH_2 vs. PNAG-12mer-45%Ac	****	<0.0001
PNAG-18mer-NH_2 vs. PNAG-12mer-60%Ac	****	<0.0001
PNAG-18mer-NH_2 vs. PNAG-18mer-45%Ac	****	<0.0001
BK vs. PNAG-4mer-45%Ac	****	<0.0001

BK vs. PNAG-8mer-40%Ac	****	<0.0001
BK vs. PNAG-12mer-45%Ac	****	<0.0001
BK vs. PNAG-12mer-60%Ac	****	<0.0001
BK vs. PNAG-18mer-45%Ac	*	0.0206
PNAG-4mer-45%Ac vs. PNAG-8mer-40%Ac	****	<0.0001
PNAG-4mer-45%Ac vs. PNAG-12mer-45%Ac	****	<0.0001
PNAG-4mer-45%Ac vs. PNAG-12mer-60%Ac	****	<0.0001
PNAG-4mer-45%Ac vs. PNAG-18mer-45%Ac	ns	0.1462
PNAG-8mer-40%Ac vs. PNAG-12mer-45%Ac	ns	0.2903
PNAG-8mer-40%Ac vs. PNAG-12mer-60%Ac	****	<0.0001
PNAG-8mer-40%Ac vs. PNAG-18mer-45%Ac	****	<0.0001
PNAG-12mer-45%Ac vs. PNAG-12mer-60%Ac	****	<0.0001
PNAG-12mer-45%Ac vs. PNAG-18mer-45%Ac	****	<0.0001
PNAG-12mer-60%Ac vs. PNAG-18mer-45%Ac	****	<0.0001
DP8_NH_2 glycan binding		
PNAG-4mer-NH_2 vs. PNAG-8mer-NH_2	****	<0.0001
PNAG-4mer-NH_2 vs. PNAG-12mer-NH_2	****	<0.0001
PNAG-4mer-NH_2 vs. PNAG-18mer-NH_2	****	<0.0001
PNAG-4mer-NH_2 vs. BK	****	<0.0001
PNAG-4mer-NH_2 vs. PNAG-4mer-45%Ac	****	<0.0001
PNAG-4mer-NH_2 vs. PNAG-8mer-40%Ac	ns	0.9967
PNAG-4mer-NH_2 vs. PNAG-12mer-45%Ac	****	<0.0001
PNAG-4mer-NH_2 vs. PNAG-12mer-60%Ac	****	<0.0001
PNAG-4mer-NH_2 vs. PNAG-18mer-45%Ac	****	<0.0001
PNAG-8mer-NH_2 vs. PNAG-12mer-NH_2	****	<0.0001
PNAG-8mer-NH_2 vs. PNAG-18mer-NH_2	****	<0.0001
PNAG-8mer-NH_2 vs. BK	****	<0.0001
PNAG-8mer-NH_2 vs. PNAG-4mer-45%Ac	****	<0.0001
PNAG-8mer-NH_2 vs. PNAG-8mer-40%Ac	****	<0.0001
PNAG-8mer-NH_2 vs. PNAG-12mer-45%Ac	****	<0.0001
PNAG-8mer-NH_2 vs. PNAG-12mer-60%Ac	****	<0.0001
PNAG-8mer-NH_2 vs. PNAG-18mer-45%Ac	****	<0.0001
PNAG-12mer-NH_2 vs. PNAG-18mer-NH_2	****	<0.0001
PNAG-12mer-NH_2 vs. BK	****	<0.0001
PNAG-12mer-NH_2 vs. PNAG-4mer-45%Ac	****	<0.0001
PNAG-12mer-NH_2 vs. PNAG-8mer-40%Ac	****	<0.0001
PNAG-12mer-NH_2 vs. PNAG-12mer-45%Ac	****	<0.0001

PNAG-12mer-NH_2 vs. PNAG-12mer-60%Ac	ns	0.0893
PNAG-12mer-NH_2 vs. PNAG-18mer-45%Ac	****	<0.0001
PNAG-18mer-NH_2 vs. BK	****	<0.0001
PNAG-18mer-NH_2 vs. PNAG-4mer-45%Ac	****	<0.0001
PNAG-18mer-NH_2 vs. PNAG-8mer-40%Ac	****	<0.0001
PNAG-18mer-NH_2 vs. PNAG-12mer-45%Ac	****	<0.0001
PNAG-18mer-NH_2 vs. PNAG-12mer-60%Ac	****	<0.0001
PNAG-18mer-NH_2 vs. PNAG-18mer-45%Ac	****	<0.0001
BK vs. PNAG-4mer-45%Ac	****	<0.0001
BK vs. PNAG-8mer-40%Ac	****	<0.0001
BK vs. PNAG-12mer-45%Ac	****	<0.0001
BK vs. PNAG-12mer-60%Ac	****	<0.0001
BK vs. PNAG-18mer-45%Ac	****	<0.0001
PNAG-4mer-45%Ac vs. PNAG-8mer-40%Ac	****	<0.0001
PNAG-4mer-45%Ac vs. PNAG-12mer-45%Ac	****	<0.0001
PNAG-4mer-45%Ac vs. PNAG-12mer-60%Ac	****	<0.0001
PNAG-4mer-45%Ac vs. PNAG-18mer-45%Ac	ns	0.2772
PNAG-8mer-40%Ac vs. PNAG-12mer-45%Ac	****	<0.0001
PNAG-8mer-40%Ac vs. PNAG-12mer-60%Ac	****	<0.0001
PNAG-8mer-40%Ac vs. PNAG-18mer-45%Ac	****	<0.0001
PNAG-12mer-45%Ac vs. PNAG-12mer-60%Ac	****	<0.0001
PNAG-12mer-45%Ac vs. PNAG-18mer-45%Ac	****	<0.0001
PNAG-12mer-60%Ac vs. PNAG-18mer-45%Ac	****	<0.0001
DP12_NH_2 glycan binding		
PNAG-4mer-NH_2 vs. PNAG-8mer-NH_2	****	<0.0001
PNAG-4mer-NH_2 vs. PNAG-12mer-NH_2	****	<0.0001
PNAG-4mer-NH_2 vs. PNAG-18mer-NH_2	****	<0.0001
PNAG-4mer-NH_2 vs. BK	****	<0.0001
PNAG-4mer-NH_2 vs. PNAG-4mer-45%Ac	****	<0.0001
PNAG-4mer-NH_2 vs. PNAG-8mer-40%Ac	ns	0.538
PNAG-4mer-NH_2 vs. PNAG-12mer-45%Ac	****	<0.0001
PNAG-4mer-NH_2 vs. PNAG-12mer-60%Ac	****	<0.0001
PNAG-4mer-NH_2 vs. PNAG-18mer-45%Ac	****	<0.0001
PNAG-8mer-NH_2 vs. PNAG-12mer-NH_2	****	<0.0001
PNAG-8mer-NH_2 vs. PNAG-18mer-NH_2	****	<0.0001
PNAG-8mer-NH_2 vs. BK	****	<0.0001
PNAG-8mer-NH_2 vs. PNAG-4mer-45%Ac	****	<0.0001

PNAG-8mer-NH_2 vs. PNAG-8mer-40%Ac	****	<0.0001
PNAG-8mer-NH_2 vs. PNAG-12mer-45%Ac	****	<0.0001
PNAG-8mer-NH_2 vs. PNAG-12mer-60%Ac	****	<0.0001
PNAG-8mer-NH_2 vs. PNAG-18mer-45%Ac	****	<0.0001
PNAG-12mer-NH_2 vs. PNAG-18mer-NH_2	****	<0.0001
PNAG-12mer-NH_2 vs. BK	****	<0.0001
PNAG-12mer-NH_2 vs. PNAG-4mer-45%Ac	****	<0.0001
PNAG-12mer-NH_2 vs. PNAG-8mer-40%Ac	****	<0.0001
PNAG-12mer-NH_2 vs. PNAG-12mer-45%Ac	****	<0.0001
PNAG-12mer-NH_2 vs. PNAG-12mer-60%Ac	****	<0.0001
PNAG-12mer-NH_2 vs. PNAG-18mer-45%Ac	****	<0.0001
PNAG-18mer-NH_2 vs. BK	****	<0.0001
PNAG-18mer-NH_2 vs. PNAG-4mer-45%Ac	****	<0.0001
PNAG-18mer-NH_2 vs. PNAG-8mer-40%Ac	****	<0.0001
PNAG-18mer-NH_2 vs. PNAG-12mer-45%Ac	****	<0.0001
PNAG-18mer-NH_2 vs. PNAG-12mer-60%Ac	****	<0.0001
PNAG-18mer-NH_2 vs. PNAG-18mer-45%Ac	****	<0.0001
BK vs. PNAG-4mer-45%Ac	***	0.0004
BK vs. PNAG-8mer-40%Ac	****	<0.0001
BK vs. PNAG-12mer-45%Ac	****	<0.0001
BK vs. PNAG-12mer-60%Ac	****	<0.0001
BK vs. PNAG-18mer-45%Ac	****	<0.0001
PNAG-4mer-45%Ac vs. PNAG-8mer-40%Ac	****	<0.0001
PNAG-4mer-45%Ac vs. PNAG-12mer-45%Ac	****	<0.0001
PNAG-4mer-45%Ac vs. PNAG-12mer-60%Ac	****	<0.0001
PNAG-4mer-45%Ac vs. PNAG-18mer-45%Ac	****	<0.0001
PNAG-8mer-40%Ac vs. PNAG-12mer-45%Ac	****	<0.0001
PNAG-8mer-40%Ac vs. PNAG-12mer-60%Ac	****	<0.0001
PNAG-8mer-40%Ac vs. PNAG-18mer-45%Ac	****	<0.0001
PNAG-12mer-45%Ac vs. PNAG-12mer-60%Ac	****	<0.0001
PNAG-12mer-45%Ac vs. PNAG-18mer-45%Ac	****	<0.0001
PNAG-12mer-60%Ac vs. PNAG-18mer-45%Ac	****	<0.0001
DP18_NH_2 glycan binding		
PNAG-4mer-NH_2 vs. PNAG-8mer-NH_2	****	<0.0001
PNAG-4mer-NH_2 vs. PNAG-12mer-NH_2	****	<0.0001
PNAG-4mer-NH_2 vs. PNAG-18mer-NH_2	****	<0.0001
PNAG-4mer-NH_2 vs. BK	****	<0.0001

PNAG-4mer-NH_2 vs. PNAG-4mer-45%Ac	****	<0.0001
PNAG-4mer-NH_2 vs. PNAG-8mer-40%Ac	ns	0.2134
PNAG-4mer-NH_2 vs. PNAG-12mer-45%Ac	ns	0.1773
PNAG-4mer-NH_2 vs. PNAG-12mer-60%Ac	****	<0.0001
PNAG-4mer-NH_2 vs. PNAG-18mer-45%Ac	****	<0.0001
PNAG-8mer-NH_2 vs. PNAG-12mer-NH_2	****	<0.0001
PNAG-8mer-NH_2 vs. PNAG-18mer-NH_2	****	<0.0001
PNAG-8mer-NH_2 vs. BK	****	<0.0001
PNAG-8mer-NH_2 vs. PNAG-4mer-45%Ac	****	<0.0001
PNAG-8mer-NH_2 vs. PNAG-8mer-40%Ac	****	<0.0001
PNAG-8mer-NH_2 vs. PNAG-12mer-45%Ac	****	<0.0001
PNAG-8mer-NH_2 vs. PNAG-12mer-60%Ac	****	<0.0001
PNAG-8mer-NH_2 vs. PNAG-18mer-45%Ac	****	<0.0001
PNAG-12mer-NH_2 vs. PNAG-18mer-NH_2	****	<0.0001
PNAG-12mer-NH_2 vs. BK	****	<0.0001
PNAG-12mer-NH_2 vs. PNAG-4mer-45%Ac	****	<0.0001
PNAG-12mer-NH_2 vs. PNAG-8mer-40%Ac	ns	0.0835
PNAG-12mer-NH_2 vs. PNAG-12mer-45%Ac	****	<0.0001
PNAG-12mer-NH_2 vs. PNAG-12mer-60%Ac	**	0.0082
PNAG-12mer-NH_2 vs. PNAG-18mer-45%Ac	****	<0.0001
PNAG-18mer-NH_2 vs. BK	****	<0.0001
PNAG-18mer-NH_2 vs. PNAG-4mer-45%Ac	****	<0.0001
PNAG-18mer-NH_2 vs. PNAG-8mer-40%Ac	****	<0.0001
PNAG-18mer-NH_2 vs. PNAG-12mer-45%Ac	****	<0.0001
PNAG-18mer-NH_2 vs. PNAG-12mer-60%Ac	ns	0.6642
PNAG-18mer-NH_2 vs. PNAG-18mer-45%Ac	****	<0.0001
BK vs. PNAG-4mer-45%Ac	ns	0.6488
BK vs. PNAG-8mer-40%Ac	****	<0.0001
BK vs. PNAG-12mer-45%Ac	****	<0.0001
BK vs. PNAG-12mer-60%Ac	****	<0.0001
BK vs. PNAG-18mer-45%Ac	****	<0.0001
PNAG-4mer-45%Ac vs. PNAG-8mer-40%Ac	****	<0.0001
PNAG-4mer-45%Ac vs. PNAG-12mer-45%Ac	****	<0.0001
PNAG-4mer-45%Ac vs. PNAG-12mer-60%Ac	****	<0.0001
PNAG-4mer-45%Ac vs. PNAG-18mer-45%Ac	****	<0.0001
PNAG-8mer-40%Ac vs. PNAG-12mer-45%Ac	****	<0.0001
PNAG-8mer-40%Ac vs. PNAG-12mer-60%Ac	****	<0.0001

PNAG-8mer-40%Ac vs. PNAG-18mer-45%Ac	****	<0.0001
PNAG-12mer-45%Ac vs. PNAG-12mer-60%Ac	****	<0.0001
PNAG-12mer-45%Ac vs. PNAG-18mer-45%Ac	****	<0.0001
PNAG-12mer-60%Ac vs. PNAG-18mer-45%Ac	****	<0.0001
DP4_45%NHAc glycan binding		
PNAG-4mer-NH_2 vs. PNAG-8mer-NH_2	****	<0.0001
PNAG-4mer-NH_2 vs. PNAG-12mer-NH_2	****	<0.0001
PNAG-4mer-NH_2 vs. PNAG-18mer-NH_2	****	<0.0001
PNAG-4mer-NH_2 vs. BK	****	<0.0001
PNAG-4mer-NH_2 vs. PNAG-4mer-45%Ac	****	<0.0001
PNAG-4mer-NH_2 vs. PNAG-8mer-40%Ac	****	<0.0001
PNAG-4mer-NH_2 vs. PNAG-12mer-45%Ac	****	<0.0001
PNAG-4mer-NH_2 vs. PNAG-12mer-60%Ac	****	<0.0001
PNAG-4mer-NH_2 vs. PNAG-18mer-45%Ac	****	<0.0001
PNAG-8mer-NH_2 vs. PNAG-12mer-NH_2	****	<0.0001
PNAG-8mer-NH_2 vs. PNAG-18mer-NH_2	****	<0.0001
PNAG-8mer-NH_2 vs. BK	****	<0.0001
PNAG-8mer-NH_2 vs. PNAG-4mer-45%Ac	****	<0.0001
PNAG-8mer-NH_2 vs. PNAG-8mer-40%Ac	****	<0.0001
PNAG-8mer-NH_2 vs. PNAG-12mer-45%Ac	****	<0.0001
PNAG-8mer-NH_2 vs. PNAG-12mer-60%Ac	****	<0.0001
PNAG-8mer-NH_2 vs. PNAG-18mer-45%Ac	****	<0.0001
PNAG-12mer-NH_2 vs. PNAG-18mer-NH_2	****	<0.0001
PNAG-12mer-NH_2 vs. BK	****	<0.0001
PNAG-12mer-NH_2 vs. PNAG-4mer-45%Ac	****	<0.0001
PNAG-12mer-NH_2 vs. PNAG-8mer-40%Ac	****	<0.0001
PNAG-12mer-NH_2 vs. PNAG-12mer-45%Ac	****	<0.0001
PNAG-12mer-NH_2 vs. PNAG-12mer-60%Ac	****	<0.0001
PNAG-12mer-NH_2 vs. PNAG-18mer-45%Ac	****	<0.0001
PNAG-18mer-NH_2 vs. BK	*	0.0105
PNAG-18mer-NH_2 vs. PNAG-4mer-45%Ac	ns	0.7124
PNAG-18mer-NH_2 vs. PNAG-8mer-40%Ac	****	<0.0001
PNAG-18mer-NH_2 vs. PNAG-12mer-45%Ac	****	<0.0001
PNAG-18mer-NH_2 vs. PNAG-12mer-60%Ac	****	<0.0001
PNAG-18mer-NH_2 vs. PNAG-18mer-45%Ac	ns	0.2247
BK vs. PNAG-4mer-45%Ac	ns	0.6473
BK vs. PNAG-8mer-40%Ac	****	<0.0001

BK vs. PNAG-12mer-45%Ac	****	<0.0001
BK vs. PNAG-12mer-60%Ac	****	<0.0001
BK vs. PNAG-18mer-45%Ac	ns	0.978
PNAG-4mer-45%Ac vs. PNAG-8mer-40%Ac	****	<0.0001
PNAG-4mer-45%Ac vs. PNAG-12mer-45%Ac	****	<0.0001
PNAG-4mer-45%Ac vs. PNAG-12mer-60%Ac	****	<0.0001
PNAG-4mer-45%Ac vs. PNAG-18mer-45%Ac	ns	0.9986
PNAG-8mer-40%Ac vs. PNAG-12mer-45%Ac	****	<0.0001
PNAG-8mer-40%Ac vs. PNAG-12mer-60%Ac	****	<0.0001
PNAG-8mer-40%Ac vs. PNAG-18mer-45%Ac	****	<0.0001
PNAG-12mer-45%Ac vs. PNAG-12mer-60%Ac	ns	0.5917
PNAG-12mer-45%Ac vs. PNAG-18mer-45%Ac	****	<0.0001
PNAG-12mer-60%Ac vs. PNAG-18mer-45%Ac	****	<0.0001
DP8_40%NHAc glycan binding		
PNAG-4mer-NH_2 vs. PNAG-8mer-NH_2	****	<0.0001
PNAG-4mer-NH_2 vs. PNAG-12mer-NH_2	***	0.0006
PNAG-4mer-NH_2 vs. PNAG-18mer-NH_2	****	<0.0001
PNAG-4mer-NH_2 vs. BK	****	<0.0001
PNAG-4mer-NH_2 vs. PNAG-4mer-45%Ac	****	<0.0001
PNAG-4mer-NH_2 vs. PNAG-8mer-40%Ac	****	<0.0001
PNAG-4mer-NH_2 vs. PNAG-12mer-45%Ac	****	<0.0001
PNAG-4mer-NH_2 vs. PNAG-12mer-60%Ac	*	0.0375
PNAG-4mer-NH_2 vs. PNAG-18mer-45%Ac	****	<0.0001
PNAG-8mer-NH_2 vs. PNAG-12mer-NH_2	****	<0.0001
PNAG-8mer-NH_2 vs. PNAG-18mer-NH_2	****	<0.0001
PNAG-8mer-NH_2 vs. BK	****	<0.0001
PNAG-8mer-NH_2 vs. PNAG-4mer-45%Ac	****	<0.0001
PNAG-8mer-NH_2 vs. PNAG-8mer-40%Ac	ns	0.7488
PNAG-8mer-NH_2 vs. PNAG-12mer-45%Ac	****	<0.0001
PNAG-8mer-NH_2 vs. PNAG-12mer-60%Ac	****	<0.0001
PNAG-8mer-NH_2 vs. PNAG-18mer-45%Ac	****	<0.0001
PNAG-12mer-NH_2 vs. PNAG-18mer-NH_2	****	<0.0001
PNAG-12mer-NH_2 vs. BK	****	<0.0001
PNAG-12mer-NH_2 vs. PNAG-4mer-45%Ac	****	<0.0001
PNAG-12mer-NH_2 vs. PNAG-8mer-40%Ac	****	<0.0001
PNAG-12mer-NH_2 vs. PNAG-12mer-45%Ac	****	<0.0001
PNAG-12mer-NH_2 vs. PNAG-12mer-60%Ac	****	<0.0001

PNAG-12mer-NH_2 vs. PNAG-18mer-45%Ac	****	<0.0001
PNAG-18mer-NH_2 vs. BK	****	<0.0001
PNAG-18mer-NH_2 vs. PNAG-4mer-45%Ac	****	<0.0001
PNAG-18mer-NH_2 vs. PNAG-8mer-40%Ac	****	<0.0001
PNAG-18mer-NH_2 vs. PNAG-12mer-45%Ac	****	<0.0001
PNAG-18mer-NH_2 vs. PNAG-12mer-60%Ac	****	<0.0001
PNAG-18mer-NH_2 vs. PNAG-18mer-45%Ac	****	<0.0001
BK vs. PNAG-4mer-45%Ac	ns	0.1305
BK vs. PNAG-8mer-40%Ac	****	<0.0001
BK vs. PNAG-12mer-45%Ac	****	<0.0001
BK vs. PNAG-12mer-60%Ac	****	<0.0001
BK vs. PNAG-18mer-45%Ac	****	<0.0001
PNAG-4mer-45%Ac vs. PNAG-8mer-40%Ac	****	<0.0001
PNAG-4mer-45%Ac vs. PNAG-12mer-45%Ac	****	<0.0001
PNAG-4mer-45%Ac vs. PNAG-12mer-60%Ac	****	<0.0001
PNAG-4mer-45%Ac vs. PNAG-18mer-45%Ac	ns	0.1222
PNAG-8mer-40%Ac vs. PNAG-12mer-45%Ac	****	<0.0001
PNAG-8mer-40%Ac vs. PNAG-12mer-60%Ac	****	<0.0001
PNAG-8mer-40%Ac vs. PNAG-18mer-45%Ac	****	<0.0001
PNAG-12mer-45%Ac vs. PNAG-12mer-60%Ac	****	<0.0001
PNAG-12mer-45%Ac vs. PNAG-18mer-45%Ac	****	<0.0001
PNAG-12mer-60%Ac vs. PNAG-18mer-45%Ac	****	<0.0001
DP12_45%NHAc glycan binding		
PNAG-4mer-NH_2 vs. PNAG-8mer-NH_2	****	<0.0001
PNAG-4mer-NH_2 vs. PNAG-12mer-NH_2	****	<0.0001
PNAG-4mer-NH_2 vs. PNAG-18mer-NH_2	****	<0.0001
PNAG-4mer-NH_2 vs. BK	****	<0.0001
PNAG-4mer-NH_2 vs. PNAG-4mer-45%Ac	****	<0.0001
PNAG-4mer-NH_2 vs. PNAG-8mer-40%Ac	****	<0.0001
PNAG-4mer-NH_2 vs. PNAG-12mer-45%Ac	****	<0.0001
PNAG-4mer-NH_2 vs. PNAG-12mer-60%Ac	****	<0.0001
PNAG-4mer-NH_2 vs. PNAG-18mer-45%Ac	****	<0.0001
PNAG-8mer-NH_2 vs. PNAG-12mer-NH_2	****	<0.0001
PNAG-8mer-NH_2 vs. PNAG-18mer-NH_2	****	<0.0001
PNAG-8mer-NH_2 vs. BK	****	<0.0001
PNAG-8mer-NH_2 vs. PNAG-4mer-45%Ac	****	<0.0001
PNAG-8mer-NH_2 vs. PNAG-8mer-40%Ac	****	<0.0001

PNAG-8mer-NH_2 vs. PNAG-12mer-45%Ac	****	<0.0001
PNAG-8mer-NH_2 vs. PNAG-12mer-60%Ac	****	<0.0001
PNAG-8mer-NH_2 vs. PNAG-18mer-45%Ac	****	<0.0001
PNAG-12mer-NH_2 vs. PNAG-18mer-NH_2	****	<0.0001
PNAG-12mer-NH_2 vs. BK	****	<0.0001
PNAG-12mer-NH_2 vs. PNAG-4mer-45%Ac	****	<0.0001
PNAG-12mer-NH_2 vs. PNAG-8mer-40%Ac	****	<0.0001
PNAG-12mer-NH_2 vs. PNAG-12mer-45%Ac	****	<0.0001
PNAG-12mer-NH_2 vs. PNAG-12mer-60%Ac	****	<0.0001
PNAG-12mer-NH_2 vs. PNAG-18mer-45%Ac	****	<0.0001
PNAG-18mer-NH_2 vs. BK	****	<0.0001
PNAG-18mer-NH_2 vs. PNAG-4mer-45%Ac	****	<0.0001
PNAG-18mer-NH_2 vs. PNAG-8mer-40%Ac	****	<0.0001
PNAG-18mer-NH_2 vs. PNAG-12mer-45%Ac	****	<0.0001
PNAG-18mer-NH_2 vs. PNAG-12mer-60%Ac	****	<0.0001
PNAG-18mer-NH_2 vs. PNAG-18mer-45%Ac	****	<0.0001
BK vs. PNAG-4mer-45%Ac	***	0.0002
BK vs. PNAG-8mer-40%Ac	****	<0.0001
BK vs. PNAG-12mer-45%Ac	****	<0.0001
BK vs. PNAG-12mer-60%Ac	****	<0.0001
BK vs. PNAG-18mer-45%Ac	****	<0.0001
PNAG-4mer-45%Ac vs. PNAG-8mer-40%Ac	****	<0.0001
PNAG-4mer-45%Ac vs. PNAG-12mer-45%Ac	****	<0.0001
PNAG-4mer-45%Ac vs. PNAG-12mer-60%Ac	****	<0.0001
PNAG-4mer-45%Ac vs. PNAG-18mer-45%Ac	****	<0.0001
PNAG-8mer-40%Ac vs. PNAG-12mer-45%Ac	****	<0.0001
PNAG-8mer-40%Ac vs. PNAG-12mer-60%Ac	****	<0.0001
PNAG-8mer-40%Ac vs. PNAG-18mer-45%Ac	****	<0.0001
PNAG-12mer-45%Ac vs. PNAG-12mer-60%Ac	****	<0.0001
PNAG-12mer-45%Ac vs. PNAG-18mer-45%Ac	****	<0.0001
PNAG-12mer-60%Ac vs. PNAG-18mer-45%Ac	****	<0.0001
DP12_60%NHAc glycan binding		
PNAG-4mer-NH_2 vs. PNAG-8mer-NH_2	****	<0.0001
PNAG-4mer-NH_2 vs. PNAG-12mer-NH_2	****	<0.0001
PNAG-4mer-NH_2 vs. PNAG-18mer-NH_2	****	<0.0001
PNAG-4mer-NH_2 vs. BK	****	<0.0001
PNAG-4mer-NH_2 vs. PNAG-4mer-45%Ac	****	<0.0001

PNAG-4mer-NH_2 vs. PNAG-8mer-40%Ac	****	<0.0001
PNAG-4mer-NH_2 vs. PNAG-12mer-45%Ac	****	<0.0001
PNAG-4mer-NH_2 vs. PNAG-12mer-60%Ac	****	<0.0001
PNAG-4mer-NH_2 vs. PNAG-18mer-45%Ac	****	<0.0001
PNAG-8mer-NH_2 vs. PNAG-12mer-NH_2	****	<0.0001
PNAG-8mer-NH_2 vs. PNAG-18mer-NH_2	****	<0.0001
PNAG-8mer-NH_2 vs. BK	****	<0.0001
PNAG-8mer-NH_2 vs. PNAG-4mer-45%Ac	****	<0.0001
PNAG-8mer-NH_2 vs. PNAG-8mer-40%Ac	****	<0.0001
PNAG-8mer-NH_2 vs. PNAG-12mer-45%Ac	ns	0.9091
PNAG-8mer-NH_2 vs. PNAG-12mer-60%Ac	****	<0.0001
PNAG-8mer-NH_2 vs. PNAG-18mer-45%Ac	****	<0.0001
PNAG-12mer-NH_2 vs. PNAG-18mer-NH_2	****	<0.0001
PNAG-12mer-NH_2 vs. BK	****	<0.0001
PNAG-12mer-NH_2 vs. PNAG-4mer-45%Ac	****	<0.0001
PNAG-12mer-NH_2 vs. PNAG-8mer-40%Ac	****	<0.0001
PNAG-12mer-NH_2 vs. PNAG-12mer-45%Ac	****	<0.0001
PNAG-12mer-NH_2 vs. PNAG-12mer-60%Ac	****	<0.0001
PNAG-12mer-NH_2 vs. PNAG-18mer-45%Ac	****	<0.0001
PNAG-18mer-NH_2 vs. BK	****	<0.0001
PNAG-18mer-NH_2 vs. PNAG-4mer-45%Ac	****	<0.0001
PNAG-18mer-NH_2 vs. PNAG-8mer-40%Ac	****	<0.0001
PNAG-18mer-NH_2 vs. PNAG-12mer-45%Ac	****	<0.0001
PNAG-18mer-NH_2 vs. PNAG-12mer-60%Ac	****	<0.0001
PNAG-18mer-NH_2 vs. PNAG-18mer-45%Ac	**	0.0094
BK vs. PNAG-4mer-45%Ac	****	<0.0001
BK vs. PNAG-8mer-40%Ac	****	<0.0001
BK vs. PNAG-12mer-45%Ac	****	<0.0001
BK vs. PNAG-12mer-60%Ac	****	<0.0001
BK vs. PNAG-18mer-45%Ac	****	<0.0001
PNAG-4mer-45%Ac vs. PNAG-8mer-40%Ac	****	<0.0001
PNAG-4mer-45%Ac vs. PNAG-12mer-45%Ac	****	<0.0001
PNAG-4mer-45%Ac vs. PNAG-12mer-60%Ac	****	<0.0001
PNAG-4mer-45%Ac vs. PNAG-18mer-45%Ac	****	<0.0001
PNAG-8mer-40%Ac vs. PNAG-12mer-45%Ac	****	<0.0001
PNAG-8mer-40%Ac vs. PNAG-12mer-60%Ac	****	<0.0001
PNAG-8mer-40%Ac vs. PNAG-18mer-45%Ac	****	<0.0001

PNAG-12mer-45%Ac vs. PNAG-12mer-60%Ac	****	<0.0001
PNAG-12mer-45%Ac vs. PNAG-18mer-45%Ac	****	<0.0001
PNAG-12mer-60%Ac vs. PNAG-18mer-45%Ac	****	<0.0001
DP18_45%NHAc glycan binding		
PNAG-4mer-NH_2 vs. PNAG-8mer-NH_2	****	<0.0001
PNAG-4mer-NH_2 vs. PNAG-12mer-NH_2	****	<0.0001
PNAG-4mer-NH_2 vs. PNAG-18mer-NH_2	****	<0.0001
PNAG-4mer-NH_2 vs. BK	****	<0.0001
PNAG-4mer-NH_2 vs. PNAG-4mer-45%Ac	****	<0.0001
PNAG-4mer-NH_2 vs. PNAG-8mer-40%Ac	****	<0.0001
PNAG-4mer-NH_2 vs. PNAG-12mer-45%Ac	****	<0.0001
PNAG-4mer-NH_2 vs. PNAG-12mer-60%Ac	****	<0.0001
PNAG-4mer-NH_2 vs. PNAG-18mer-45%Ac	****	<0.0001
PNAG-8mer-NH_2 vs. PNAG-12mer-NH_2	****	<0.0001
PNAG-8mer-NH_2 vs. PNAG-18mer-NH_2	****	<0.0001
PNAG-8mer-NH_2 vs. BK	****	<0.0001
PNAG-8mer-NH_2 vs. PNAG-4mer-45%Ac	****	<0.0001
PNAG-8mer-NH_2 vs. PNAG-8mer-40%Ac	****	<0.0001
PNAG-8mer-NH_2 vs. PNAG-12mer-45%Ac	****	<0.0001
PNAG-8mer-NH_2 vs. PNAG-12mer-60%Ac	****	<0.0001
PNAG-8mer-NH_2 vs. PNAG-18mer-45%Ac	****	<0.0001
PNAG-12mer-NH_2 vs. PNAG-18mer-NH_2	****	<0.0001
PNAG-12mer-NH_2 vs. BK	****	<0.0001
PNAG-12mer-NH_2 vs. PNAG-4mer-45%Ac	****	<0.0001
PNAG-12mer-NH_2 vs. PNAG-8mer-40%Ac	****	<0.0001
PNAG-12mer-NH_2 vs. PNAG-12mer-45%Ac	****	<0.0001
PNAG-12mer-NH_2 vs. PNAG-12mer-60%Ac	****	<0.0001
PNAG-12mer-NH_2 vs. PNAG-18mer-45%Ac	****	<0.0001
PNAG-18mer-NH_2 vs. BK	****	<0.0001
PNAG-18mer-NH_2 vs. PNAG-4mer-45%Ac	****	<0.0001
PNAG-18mer-NH_2 vs. PNAG-8mer-40%Ac	****	<0.0001
PNAG-18mer-NH_2 vs. PNAG-12mer-45%Ac	****	<0.0001
PNAG-18mer-NH_2 vs. PNAG-12mer-60%Ac	****	<0.0001
PNAG-18mer-NH_2 vs. PNAG-18mer-45%Ac	****	<0.0001
BK vs. PNAG-4mer-45%Ac	****	<0.0001
BK vs. PNAG-8mer-40%Ac	****	<0.0001
BK vs. PNAG-12mer-45%Ac	****	<0.0001

BK vs. PNAG-12mer-60%Ac	****	<0.0001
BK vs. PNAG-18mer-45%Ac	****	<0.0001
PNAG-4mer-45%Ac vs. PNAG-8mer-40%Ac	****	<0.0001
PNAG-4mer-45%Ac vs. PNAG-12mer-45%Ac	****	<0.0001
PNAG-4mer-45%Ac vs. PNAG-12mer-60%Ac	****	<0.0001
PNAG-4mer-45%Ac vs. PNAG-18mer-45%Ac	****	<0.0001
PNAG-8mer-40%Ac vs. PNAG-12mer-45%Ac	****	<0.0001
PNAG-8mer-40%Ac vs. PNAG-12mer-60%Ac	****	<0.0001
PNAG-8mer-40%Ac vs. PNAG-18mer-45%Ac	****	<0.0001
PNAG-12mer-45%Ac vs. PNAG-12mer-60%Ac	****	<0.0001
PNAG-12mer-45%Ac vs. PNAG-18mer-45%Ac	****	<0.0001
PNAG-12mer-60%Ac vs. PNAG-18mer-45%Ac	****	<0.0001

Statistical analysis was performed with one-way ANOVA followed by Tukey's multiple comparison test using GraphPad Prism version 10.4.0 for macOS.

Table S4: Statistical significance and P values for the opsonophagocytic killing assay with *Staphylococcus aureus* (Newman)

Staphylococcus aureus (Newman)							
PNAG-oligomer-NH_2							
	Serum dilution						
Tukey's multiple comparisons test	1:10	1:20	1:40	1:80	1:160	1:320	1:640
PNAG-8mer-NH_2 vs. PNAG-12mer-NH2	****	**	ns	ns	ns	ns	ns
PNAG-8mer-NH_2 vs. PNAG-18mer-NH_2	ns	ns	ns	ns	ns	ns	ns
PNAG-8mer-NH_2 vs. PBS control	****	****	****	****	****	****	****
PNAG-8mer-NH_2 vs. PNAG-4mer-NH_2	****	***	**	**	****	****	****
PNAG-12mer-NH_2 vs. PNAG-18mer-NH_2	****	*	ns	ns	ns	ns	ns
PNAG-12mer-NH_2 vs. PBS control	****	**	**	****	****	****	****
PNAG-12mer-NH_2 vs. PNAG-4mer-NH_2	ns	ns	ns	ns	****	****	***
PNAG-18mer-NH_2 vs. PBS control	****	****	****	****	****	****	****
PNAG-18mer-NH_2 vs. PNAG-4mer-NH_2	****	**	**	ns	***	****	***
PBS control vs. PNAG-4mer-NH_2	**	ns	ns	**	**	**	*
PNAG-oligomer-NHAc							
	Serum dilution						
Tukey's multiple comparisons test	1:10	1:20	1:40	1:80	1:160	1:320	1:640
PNAG-8mer 40% NHAc vs. PNAG-12mer 45% NHAc	ns	ns	ns	ns	ns	ns	ns
PNAG-8mer 40% NHAc vs. PNAG-12mer 60% NHAc	ns	ns	ns	ns	ns	ns	ns
PNAG-8mer 40% NHAc vs. PNAG-18mer 40% NHAc	ns	ns	ns	ns	ns	ns	ns
PNAG-8mer 40% NHAc vs. PBS control	****	****	****	***	****	****	****
PNAG-8mer 40% NHAc vs. PNAG-4mer 45% NHAc	****	****	**	ns	ns	*	ns
PNAG-12mer 45% NHAc vs. PNAG-12mer 60% NHAc	ns	ns	ns	ns	ns	ns	ns
PNAG-12mer 45% NHAc vs. PNAG-18mer 40% NHAc	ns	ns	ns	ns	ns	ns	ns
PNAG-12mer 45% NHAc vs. PBS control	****	****	****	****	****	****	****

PNAG-12mer 45% NHAc vs. PNAG-4mer 45% NHAc	****	****	**	ns	*	*	ns
PNAG-12mer 60% NHAc vs. PNAG-18mer 40% NHAc	ns	ns	ns	ns	ns	ns	ns
PNAG-12mer 60% NHAc vs. PBS control	****	****	****	**	****	****	****
PNAG-12mer 60% NHAc vs. PNAG-4mer 45% NHAc	**	***	*	ns	ns	ns	ns
PNAG-18mer 40% NHAc vs. PBS control	****	****	****	****	****	****	****
PNAG-18mer 40% NHAc vs. PNAG-4mer 45% NHAc	**	***	**	ns	*	**	ns
PBS control vs. PNAG-4mer 45% NHAc	ns	*	ns	*	****	****	**

Statistical analysis was performed with one-way ANOVA followed by Tukey's multiple comparison test using GraphPad Prism version 10.4.0 for macOS. ****: P < 0.0001; ***: P< 0.001; **: P < 0.01; P < 0.05; ns: not significant

Table S5: Statistical significance and P values for the opsonophagocytic killing assay with *Strepcoccus pneumoniaea* (19A)

Strepcoccus pneumoniaea (19A)							
PNAG-oligomer-NH_2							
	Serum dilution						
Tukey's multiple comparisons test	1:10	1:20	1:40	1:80	1:160	1:320	1:640
PNAG-4mer-NH_2 vs. PNAG-8mer-NH_2	**	***	**	**	ns	ns	ns
PNAG-4mer-NH_2 vs. PNAG-12mer-NH_2	ns	ns	ns	ns	ns	ns	ns
PNAG-4mer-NH_2 vs. PNAG-18mer-NH_2	*	**	*	ns	ns	*	**
PNAG-4mer-NH_2 vs. PBS	*	*	***	****	****	****	****
PNAG-8mer-NH_2 vs. PNAG-12mer-NH_2	*	*	*	**	ns	ns	ns
PNAG-8mer-NH_2 vs. PNAG-18mer-NH_2	ns	ns	ns	ns	ns	ns	ns
PNAG-8mer-NH_2 vs. PBS	****	****	****	****	****	****	****
PNAG-12mer-NH_2 vs. PNAG-18mer-NH_2	*	*	ns	ns	ns	ns	ns
PNAG-12mer-NH_2 vs. PBS	*	**	****	****	****	****	****
PNAG-18mer-NH_2 vs. PBS	****	****	****	****	****	****	****
PNAG-oligomer-NHAc							
	Serum dilution						
Tukey's multiple comparisons test	1:10	1:20	1:40	1:80	1:160	1:320	1:640
PNAG-4mer-45%Ac vs. PNAG-8mer-40%Ac	***	*	*	ns	ns	ns	ns
PNAG-4mer-45%Ac vs. PNAG-12mer-45%Ac	**	ns	ns	ns	ns	ns	ns
PNAG-4mer-45%Ac vs. PNAG-12mer-60%Ac	ns	ns	ns	ns	ns	ns	ns
PNAG-4mer-45%Ac vs. PNAG-18mer-40-45%Ac	ns	ns	ns	***	**	*	**
PNAG-4mer-45%Ac vs. PBS	**	***	****	****	****	****	****
PNAG-8mer-40%Ac vs. PNAG-12mer-45%Ac	ns	ns	ns	ns	ns	ns	ns
PNAG-8mer-40%Ac vs. PNAG-12mer-60%Ac	ns	ns	**	*	**	*	*
PNAG-8mer-40%Ac vs. PNAG-18mer-40-45%Ac	*	**	****	****	****	****	****
PNAG-8mer-40%Ac vs. PBS	****	****	****	****	****	****	****
PNAG-12mer-45%Ac vs. PNAG-12mer-60%Ac	ns	ns	ns	ns	**	*	*
PNAG-12mer-45%Ac vs. PNAG-18mer-40-45%Ac	ns	ns	***	****	****	****	***
PNAG-12mer-45%Ac vs. PBS	****	****	****	****	****	****	****
PNAG-12mer-60%Ac vs. PNAG-18mer-40-45%Ac	ns	ns	ns	*	ns	ns	ns
PNAG-12mer-60%Ac vs. PBS	****	****	****	****	****	***	****
PNAG-18mer-40-45%Ac vs. PBS	****	**	**	ns	*	ns	***

Statistical analysis was performed with one-way ANOVA followed by Tukey’s multiple comparison test using GraphPad Prism version 10.4.0 for macOS. ****: $P < 0.0001$; ***: $P < 0.001$; **: $P < 0.001$; *: $P < 0.05$; ns: not significant

Table S6: Statistical significance and P values for the opsonophagocytic killing assay with *Acinetobacter baumannii* (17978)

Acinetobacter baumannii (17978)							
PNAG-oligomer-NH_2							
	Serum dilution						
Tukey's multiple comparisons test	1:10	1:20	1:40	1:80	1:160	1:320	1:640
PNAG-4mer-NH_2 vs. PNAG-8mer-NH_2	ns	ns	ns	ns	ns	ns	**
PNAG-4mer-NH_2 vs. PNAG-12mer-NH_2	ns	ns	ns	ns	ns	ns	ns
PNAG-4mer-NH_2 vs. PNAG-18mer-NH_2	ns	ns	ns	*	*	*	ns
PNAG-4mer-NH_2 vs. PBS	****	****	****	****	****	****	****
PNAG-8mer-NH_2 vs. PNAG-12mer-NH_2	ns	ns	ns	ns	ns	ns	ns
PNAG-8mer-NH_2 vs. PNAG-18mer-NH_2	ns	ns	ns	*	ns	ns	ns
PNAG-8mer-NH_2 vs. PBS	****	****	****	****	****	*	***
PNAG-12mer-NH_2 vs. PNAG-18mer-NH_2	ns	ns	ns	ns	**	ns	ns
PNAG-12mer-NH_2 vs. PBS	****	****	****	****	****	***	****
PNAG-18mer-NH_2 vs. PBS	****	****	****	****	****	ns	****
PNAG-oligomer-NHAc							
	Serum dilution						
Tukey's multiple comparisons test	1:10	1:20	1:40	1:80	1:160	1:320	1:640
PNAG-4mer-45%Ac vs. PNAG-8mer-40%Ac	ns	****	**	**	**	ns	ns
PNAG-4mer-45%Ac vs. PNAG-12mer-45%Ac	****	****	**	****	ns	**	*
PNAG-4mer-45%Ac vs. PNAG-12mer-60%Ac	****	****	****	****	*	**	ns
PNAG-4mer-45%Ac vs. PNAG-18mer-40-45%Ac	****	****	****	****	****	****	***
PNAG-4mer-45%Ac vs. PBS	****	****	****	****	****	****	****
PNAG-8mer-40%Ac vs. PNAG-12mer-45%Ac	****	****	****	****	****	**	*
PNAG-8mer-40%Ac vs. PNAG-12mer-60%Ac	****	****	****	****	****	**	ns
PNAG-8mer-40%Ac vs. PNAG-18mer-40-45%Ac	****	****	****	****	****	****	***
PNAG-8mer-40%Ac vs. PBS	****	****	****	****	****	****	****
PNAG-12mer-45%Ac vs. PNAG-	**	*	ns	ns	ns	ns	ns

12mer-60%Ac							
PNAG-12mer-45%Ac vs. PNAG-18mer-40-45%Ac	****	****	****	****	****	***	ns
PNAG-12mer-45%Ac vs. PBS	****	****	****	****	****	****	****
PNAG-12mer-60%Ac vs. PNAG-18mer-40-45%Ac	****	***	ns	****	****	***	*
PNAG-12mer-60%Ac vs. PBS	****	****	****	****	****	****	****
PNAG-18mer-40-45%Ac vs. PBS	****	****	****	****	****	****	****

Statistical analysis was performed with one-way ANOVA followed by Tukey's multiple comparison test using GraphPad Prism version 10.4.0 for macOS. ****: P < 0.0001; ***: P < 0.001; **: P < 0.001; *: P < 0.05; ns: not significant

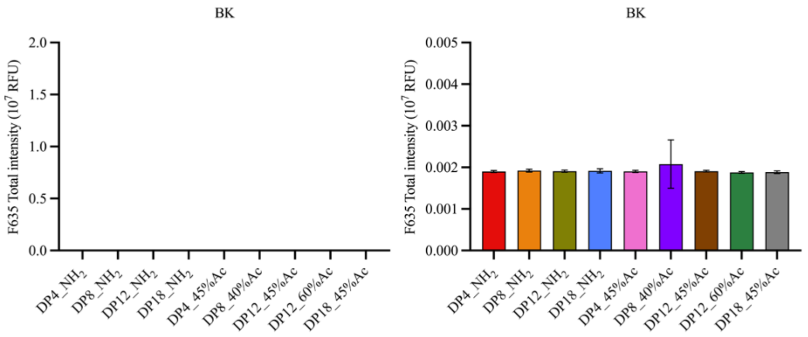

Figure S86. Glycan array analysis of antibodies that bind to dPNAG glycans in the serum from a patient without bacterial infection

A serum from a non-affected patient is considered a negative control (BK). The glycan array analysis is presented with the y-axis adjusted to accommodate its values. The experiment was performed in technical replicates, and error bars represent the standard deviation from the mean of the data point (n = 10). Statistical analysis was performed with one-way ANOVA followed by Tukey's multiple comparison test using GraphPad Prism version 10.4.0 for macOS. Statistic significance and P values are shown in Table S1. ns: not significant

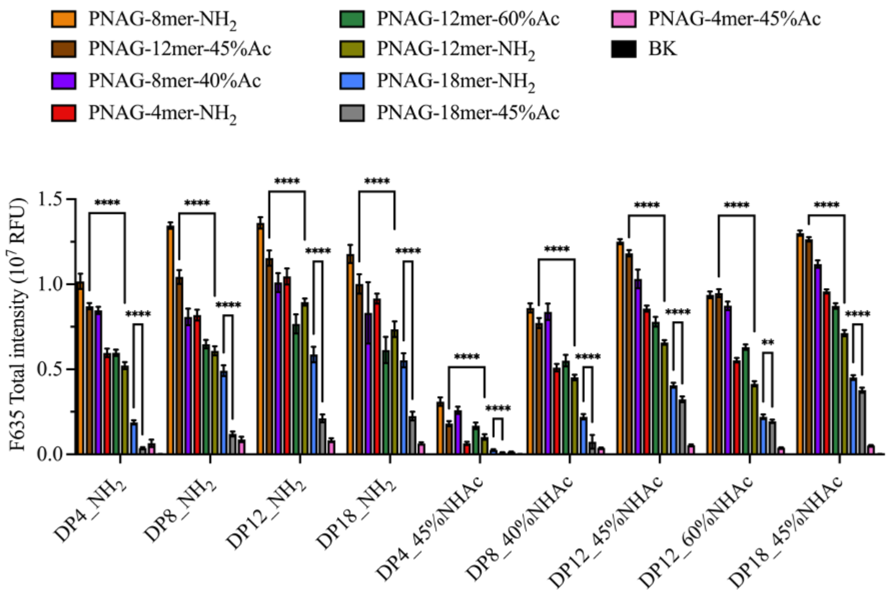

Figure S87. Immunization of mice and evaluation of the immunogenicity of the acetylated and non-acetylated dPNAG-CRM197 conjugates as vaccine candidates.

Glycan microarray binding profiles of antibodies in sera collected from mice immunized with the series of dPNAG-CRM197 conjugates. Mice (n = 5) were immunized i.m. with the same amounts (2 mg) of the different dPNAG-CRM197 conjugates and glycolipid C34. Mouse sera were collected 14 days after the third immunization. The binding profiles were determined with 1000-fold diluted antibodies. The experiment was performed in biological replicates, and error bars represent the standard deviation from the mean of the data point (n = 10). Statistical analysis was performed with one-way ANOVA followed by Tukey's multiple comparison test using GraphPad Prism version 10.4.0 for macOS. **: $p < 0.01$; ****: $p < 0.0001$.

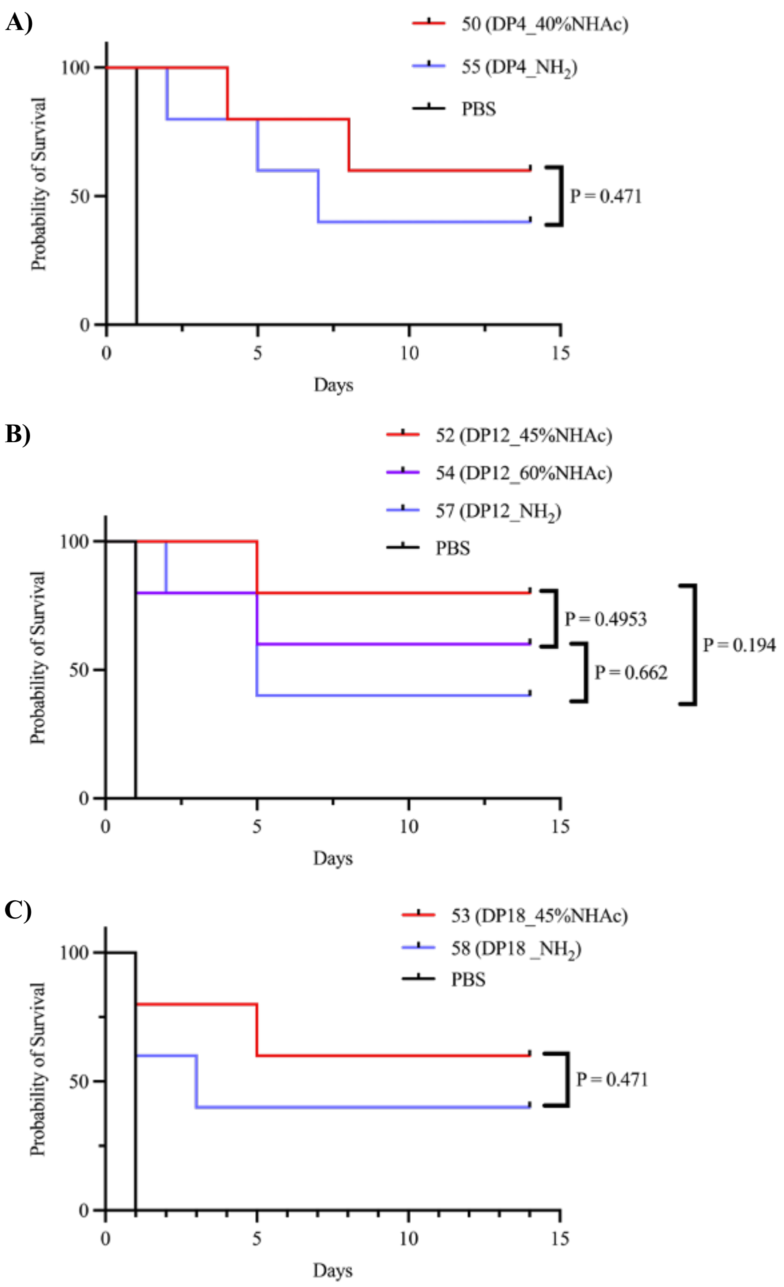

Figure S88. Evaluating *in vivo* efficacy of vaccine candidates for protection against *Staphylococcus aureus* infection.

Each group consisted of 5 mice challenged with a lethal dose. (a) Evaluation of PNAG 4mer (DP4) with 45% acetylation (**50**) and non-acetylated (**55**); (b) evaluation of PNAG 12mer (DP12) with 45% acetylation (**52**), 60% acetylation (**54**), and non-acetylated (**57**); (c); evaluation of PNAG 18mer (DP18) with 45% acetylation (**53**) and non-acetylated (**58**). Statistical analysis was performed using the two-sided Log-rank (Mantel-Cox) test in Kaplan-Meier survival analysis with GraphPad Prism version 10.4.0 for macOS. Comparisons were conducted pairwise; no multiple comparisons were performed.
