# Supplementary material for: Poly-β-D-(1,6)-N-acetyl-glucosamine (PNAG) glycan vaccines with broad spectrum neutralizing activities
Source: Nat Commun. 2025 Jul 4;16:6179. doi: 10.1038/s41467-025-61559-7 (PMC12227562; doi:10.1038/s41467-025-61559-7)
Supplement: Supplementary file 2 — Reporting Summary [file 41467_2025_61559_MOESM2_ESM.pdf]

nature portfolio

Corresponding author(s): Yves S. Y. Hsieh
Last updated by author(s): Jun 14, 2025

Reporting Summary

Nature Portfolio wishes to improve the reproducibility of the work that we publish. This form provides structure for consistency and transparency in reporting. For further information on Nature Portfolio policies, see our Editorial Policies and the Editorial Policy Checklist.

Please do not complete any field with "not applicable" or n/a. Refer to the help text for what text to use if an item is not relevant to your study.
For final submission: please carefully check your responses for accuracy; you will not be able to make changes later.

Statistics

For all statistical analyses, confirm that the following items are present in the figure legend, table legend, main text, or Methods section.

n/a	Confirmed	
☐	☒	The exact sample size (*n*) for each experimental group/condition, given as a discrete number and unit of measurement
☐	☒	A statement on whether measurements were taken from distinct samples or whether the same sample was measured repeatedly
☐	☒	The statistical test(s) used AND whether they are one- or two-sided<br>*Only common tests should be described solely by name; describe more complex techniques in the Methods section.*
☒	☐	A description of all covariates tested
☒	☐	A description of any assumptions or corrections, such as tests of normality and adjustment for multiple comparisons
☐	☒	A full description of the statistical parameters including central tendency (e.g. means) or other basic estimates (e.g. regression coefficient) AND variation (e.g. standard deviation) or associated estimates of uncertainty (e.g. confidence intervals)
☐	☒	For null hypothesis testing, the test statistic (e.g. *F*, *t*, *r*) with confidence intervals, effect sizes, degrees of freedom and *P* value noted<br>*Give P values as exact values whenever suitable.*
☒	☐	For Bayesian analysis, information on the choice of priors and Markov chain Monte Carlo settings
☒	☐	For hierarchical and complex designs, identification of the appropriate level for tests and full reporting of outcomes
☒	☐	Estimates of effect sizes (e.g. Cohen's *d*, Pearson's *r*), indicating how they were calculated

Our web collection on statistics for biologists contains articles on many of the points above.

Software and code

Policy information about availability of computer code

Data collection	No data collection made from commercial or open sources.
Data analysis	NMR spectra were analyzed by Topspin 2.1 software.<br>Microarray Acquisition and Analysis was done by GenePix Pro 7 software.<br>HR MALDI-TOF (LR MALDI-TOF) mass spectra were analyzed by Flexanalysis software.<br>ESI mass spectra recorded on APEX-ultra 9.4 T FTICR-MS were analyzed by Compass™ software suite.<br>Statistical analyses was done by GraphPad Prism version 10.4.0 for macOS

For manuscripts utilizing custom algorithms or software that are central to the research but not yet described in published literature, software must be made available to editors and reviewers. We strongly encourage code deposition in a community repository (e.g. GitHub). See the Nature Portfolio guidelines for submitting code & software for further information.

Data

Policy information about availability of data

All manuscripts must include a data availability statement. This statement should provide the following information, where applicable:

- Accession codes, unique identifiers, or web links for publicly available datasets
- A description of any restrictions on data availability
- For clinical datasets or third party data, please ensure that the statement adheres to our policy

The data that support the findings of this study are available within the article and its Supplementary Information and from the corresponding author(s) upon request. Source data are provided with this paper.

Human research participants

Policy information about studies involving human research participants and Sex and Gender in Research.

Reporting on sex and gender	Four females and two males.
Population characteristics	Total of 6 individuals: Two males age from 4Y8M to 5YM with serotypes of A. baumannii or S. pneumoniae 19F. Four females age from 4Y6M to 6Y2M, with serotypes of A. baumannii, or S. pneumoniae serotype 19A, or S. pneumoniae 23F.
Recruitment	The protocols for obtaining sera from individuals infected with A. baumannii, S. pneumoniae serotype 19A, S. pneumoniae 19F, and S. pneumoniae 23F underwent thorough scrutiny and received approval (IRB Number: 201902027A3D001). Prior to serum collection and the exchange of clinical information, all participants provided informed consent.
Ethics oversight	Institutional Review Board (IRB) of Chang Gung Medical Foundation (IRB Number: 201902027A3D001)

Note that full information on the approval of the study protocol must also be provided in the manuscript.

Field-specific reporting

Please select the one below that is the best fit for your research. If you are not sure, read the appropriate sections before making your selection.

☒ Life sciences ☐ Behavioural & social sciences ☐ Ecological, evolutionary & environmental sciences

For a reference copy of the document with all sections, see nature.com/documents/nr-reporting-summary-flat.pdf

Life sciences study design

All studies must disclose on these points even when the disclosure is negative.

Sample size	Statical sample size calculation was performed prior to the experiments. Sample sizes were determined based on previously published studies involving PNAG glycan conjugates and in vivo immunization models, as well as pilot experiments conducted in our laboratory. These sample sizes have been shown to be sufficient to detect biologically relevant differences in immunogenicity, opsonophagocytic activity, and protection outcomes. In general, 4 to 6 animals per group were used in in vivo studies, which is consistent with established practices in vaccine efficacy testing. All in vitro assays were performed in at least three independent replicates to ensure reproducibility.
Data exclusions	No data exclusion.
Replication	All experimental measurements were performed in triplicate. Error bars represent SD. All attempts of replication were successful and gave similar results.
Randomization	Allocation was not randomized; however, animals were housed and treated under identical conditions to reduce potential confounding factors. In vitro experiments were performed using parallel treatment conditions across groups. Randomization was not deemed necessary for this study, as the primary objective was to assess immunogenicity and functional antibody responses under controlled experimental conditions, rather than to evaluate interventions in a heterogeneous population.
Blinding	The experiments were not blinded.

Reporting for specific materials, systems and methods

We require information from authors about some types of materials, experimental systems and methods used in many studies. Here, indicate whether each material, system or method listed is relevant to your study. If you are not sure if a list item applies to your research, read the appropriate section before selecting a response.

Materials & experimental systems

n/a	Involved in the study
☐	☒ Antibodies
☐	☒ Eukaryotic cell lines
☒	☐ Palaeontology and archaeology
☐	☒ Animals and other organisms
☒	☐ Clinical data
☒	☐ Dual use research of concern

Methods

n/a	Involved in the study
☒	☐ ChIP-seq
☒	☐ Flow cytometry
☒	☐ MRI-based neuroimaging

Antibodies

Antibodies used	fluorescent dye Dylight™ 649 conjugated to donkey anti-human IgG (H+L chains) and AlexaFluor 647-conjugated to goat anti-mouse IgG [H+L] from (Jackson ImmunoResearch Laboratories Inc., West Grove, Pennsylvania).
Validation	All of these are secondary antibodies and therefore not applicable to the reporting of experimental reagents. Variations in their use do not impact the study design or interpretation.

Eukaryotic cell lines

Policy information about cell lines and Sex and Gender in Research

Cell line source(s)	HL Cell lines
Authentication	The HL-60 cell line was sourced from ATCC (catalog number CCL-240), which performs cell line authentication.
Mycoplasma contamination	The cell line was propagated and used immediately upon receipt. Mycoplasma testing was not performed.
Commonly misidentified lines (See ICLAC register)	Not applicable

Animals and other research organisms

Policy information about studies involving animals; ARRIVE guidelines recommended for reporting animal research, and Sex and Gender in Research

Laboratory animals	6 to 8 weeks old female BALB/c mice
Wild animals	Not applicable
Reporting on sex	Female
Field-collected samples	Not applicable
Ethics oversight	Institutional Animal Care & Use Committee (IACUC) of Academia Sinica

Note that full information on the approval of the study protocol must also be provided in the manuscript.

This checklist template is licensed under a Creative Commons Attribution 4.0 International License, which permits use, sharing, adaptation, distribution and reproduction in any medium or format, as long as you give appropriate credit to the original author(s) and the source, provide a link to the Creative Commons license, and indicate if changes were made. The images or other third party material in this article are included in the article's Creative Commons license, unless indicated otherwise in a credit line to the material. If material is not included in the article's Creative Commons license and your intended use is not permitted by statutory regulation or exceeds the permitted use, you will need to obtain permission directly from the copyright holder. To view a copy of this license, visit http://creativecommons.org/licenses/by/4.0/
